# Supplementary material for: Whole-genome sequencing analysis of semi-supercentenarians
Source: eLife. 2021 May 4;10:e57849. doi: 10.7554/eLife.57849 (PMC8096429; doi:10.7554/eLife.57849)
Supplement: Supplementary file 1. — In red are indicated the independent SNPs pruned for LD. In the last column, the p-values of the same analysis performed including PC1 and PC2 as covariates is reported. [file elife-57849-supp1.pdf]

Table 1S Position identified in the comparison between 105+/110+ and CTR with unadjusted pvalue<10-4 (logistic regression adding sex as covariate). In red are indicated the indipendent SNPs pruned for LD. In the last column the p-values of the same anlysis performed including PC1 and PC2 as covariates is reported.

CHR	POSITION	P_UNADJ	Gene Name	Nominal p_val after PC1 - PC2 correction
1	167642992	7.30E-05		3.17E-04
1	214860919	7.68E-05		3.48E-05
1	219582788	3.17E-05		1.69E-05
2	80809763	8.86E-05		5.05E-05
2	173584826	1.95E-05		1.00E-05
2	196515104	8.68E-05	SLC39A10	7.04E-05
2	196825256	8.68E-05	DNAH7	7.04E-05
2	196853313	8.68E-05	DNAH7	7.04E-05
2	196897321	8.68E-05	DNAH7	7.04E-05
2	196915345	8.68E-05	DNAH7	7.04E-05
2	196992016	8.68E-05	DNAH7	7.04E-05
2	223640943	4.21E-05		3.89E-05
3	25986861	9.84E-05		2.81E-05
3	78789217	6.17E-05		6.46E-05
3	119154425	5.59E-05		1.94E-04
5	167204970	6.87E-05		4.11E-05
5	167259833	9.39E-05		3.98E-05
6	125147538	9.38E-05		1.93E-04
7	43637796	9.73E-06	COA1	3.83E-05
7	43638009	9.73E-06	COA1	3.83E-05
7	43643835	9.73E-06	COA1	3.83E-05
7	43650221	9.73E-06	COA1	3.83E-05
7	43651047	9.73E-06	COA1	3.83E-05
7	43655836	1.19E-05	COA1	4.51E-05
7	43660622	1.19E-05	COA1	4.51E-05
7	43670562	1.19E-05	COA1	4.51E-05
7	43674069	1.31E-05	COA1	5.52E-05
7	43678340	1.31E-05	COA1	5.52E-05
7	43696550	1.19E-05	COA1	4.51E-05
7	43698933	1.19E-05	COA1	4.51E-05
7	43702472	1.19E-05	COA1	4.51E-05
7	43703597	1.19E-05	COA1	4.51E-05
7	43706100	1.19E-05	COA1	4.51E-05
7	43708856	1.19E-05	COA1	4.51E-05
7	43713111	1.19E-05	COA1	4.51E-05
7	43714795	1.19E-05	COA1	4.51E-05
7	43716226	1.19E-05	COA1	4.51E-05
7	43718560	1.19E-05	COA1	4.51E-05
7	43720429	1.19E-05	COA1	4.51E-05
7	43731169	1.19E-05	COA1	4.51E-05
7	43732947	1.19E-05	COA1	4.51E-05
7	43747968	1.19E-05	COA1	4.51E-05
7	43748383	1.19E-05	COA1	4.51E-05
7	43749785	1.19E-05	COA1	4.51E-05
7	43753941	1.19E-05	COA1	4.51E-05
7	43756081	1.19E-05	COA1	4.51E-05
7	43760206	1.19E-05	COA1	4.51E-05
7	43760883	1.19E-05	COA1	4.51E-05
7	43761590	1.19E-05	COA1	4.51E-05
7	43767009	1.19E-05	COA1	4.51E-05
7	43769436	1.19E-05	COA1	4.51E-05
7	43770797	1.19E-05	COA1	4.51E-05
7	43773209	1.19E-05	COA1	4.51E-05
7	43774147	1.19E-05	COA1	4.51E-05

CHR	POSITION	P_UNADJ	Gene Name	Nominal p_val after PC1 - PC2 correction
7	43783788	1.19E-05	COA1	4.51E-05
7	43789894	1.19E-05	COA1	4.51E-05
7	43821453	4.18E-05	COA1	1.32E-04
7	43830592	4.18E-05	COA1	1.32E-04
7	43841138	4.18E-05	COA1	1.32E-04
7	43841409	4.18E-05	COA1	1.32E-04
7	43846603	4.18E-05	COA1	1.32E-04
7	43847709	4.18E-05	COA1	1.32E-04
7	43850512	4.71E-05	COA1	1.50E-04
7	43850884	4.71E-05	COA1	1.50E-04
7	43853060	4.71E-05	COA1	1.50E-04
7	43853232	4.71E-05	COA1	1.50E-04
7	43853726	4.71E-05	COA1	1.50E-04
7	43859551	4.18E-05	COA1	1.43E-04
7	65267513	4.02E-05		6.85E-05
7	151364264	8.86E-05	PRKAG2	2.39E-04
7	151365322	3.19E-05	PRKAG2	7.85E-05
7	151365328	3.19E-05	PRKAG2	7.85E-05
7	151367148	6.99E-05	PRKAG2	1.91E-04
7	151376555	7.44E-05	PRKAG2	1.13E-04
9	66252771	7.20E-05		1.84E-04
9	103874937	1.51E-05	LPPR1	3.65E-05
9	103946357	3.52E-05	LPPR1	7.64E-05
9	103955668	5.52E-05	LPPR1	1.01E-04
9	114679493	9.71E-05		2.52E-04
9	114686410	9.71E-05	UGCG	2.52E-04
9	114688103	9.71E-05	UGCG	2.52E-04
9	114689983	9.71E-05	UGCG	2.52E-04
9	114690501	5.88E-05	UGCG	1.59E-04
9	114691177	5.88E-05	UGCG	1.59E-04
9	124293548	9.08E-05		1.23E-04
10	31346063	8.57E-05		1.21E-04
11	18852217	7.14E-05		7.03E-05
11	18853558	7.14E-05		7.03E-05
11	18854200	7.14E-05		7.03E-05
11	18866536	7.14E-05		7.03E-05
11	18873142	2.61E-05		8.59E-05
11	27523186	1.32E-05		1.87E-05
13	49897577	5.33E-05	CAB39L	6.32E-05
13	49897739	7.56E-05	CAB39L	6.32E-05
13	49897815	5.33E-05	CAB39L	6.32E-05
13	49900828	7.56E-05	CAB39L	8.56E-05
13	49901554	7.56E-05	CAB39L	8.56E-05
13	49901738	7.56E-05	CAB39L	8.56E-05
13	49905581	7.56E-05	CAB39L	8.56E-05
18	14531961	4.03E-05		7.33E-05
19	2512795	4.44E-05		4.96E-05
