# Supplementary material for: Whole-genome sequencing analysis of semi-supercentenarians
Source: eLife. 2021 May 4;10:e57849. doi: 10.7554/eLife.57849 (PMC8096429; doi:10.7554/eLife.57849)
Supplement: Supplementary file 2. — Gene name and p-values were reported. [file elife-57849-supp2.pdf]

Table 2S Gene based analysis for common variants using VEGAS (genes with a nominal pvalue< 0.01 were reported). Gene name and pvalues were reported.

GENE_NAME	P-value
LPPR1	7.20E-05
STK17A	8.40E-05
MIR4668	0.000214
PDLIM4	0.000540999
UXS1	0.000575999
PSMA5	0.000681999
SORT1	0.000718999
MAP3K9	0.000832999
UGCG	0.000918999
ISG15	0.00101999
SAT2	0.001059989
P4HA2	0.001299987
KNTC1	0.001469985
MRGPRX1	0.001499985
NACAD	0.001549985
FAM115C	0.001589984
COL11A1	0.001629984
SPEM1	0.001629984
SYPL2	0.001629984
CAB39L	0.001659983
PRAP1	0.001689983
FBLL1	0.001819982
MYBPHL	0.001889981
VWA5B1	0.00202998
BACE1-AS	0.002069979
HIST1H3G	0.002149979
HTR4	0.002159978
FLJ39080	0.002169978
LOC101928992	0.002169978
RNF214	0.002199978
APOC3	0.002209978
HIST1H4H	0.002209978
TMEM39A	0.002209978
SLC39A8	0.002259977
HIST1H2BI	0.002279977
COA1	0.002339977
SORBS3	0.002369976
RGPD1_2	0.002429976
LOC100288846	0.002469975
APOA1	0.002479975
AGRN	0.002509975
APOA4	0.002629974

GENE_NAME	P-value
TMEM178A	0.002729973
RPS10-NUDT3	0.002769972
PREX2	0.002789972
CD80	0.002899971
SERPINB13	0.00295997
SGSM2	0.00302997
DNAH7	0.00304997
LINC01270	0.00304997
TBRG4	0.003079969
HES4	0.003099969
PDLIM2	0.003129969
SERPINB12	0.003159968
LINC01247	0.003279967
NCOR1P1	0.003279967
LYZL1	0.003309967
RPS10	0.003319967
SLC39A6	0.003529965
SHBG	0.003619964
NUDT3	0.003629964
LOC643802	0.003659963
LOC389247	0.003689963
EMP2	0.003729963
BACE1	0.003769962
ZNF493	0.003789962
SLC7A11	0.003849962
TIMMDC1	0.003899961
MTRF1L	0.004099959
CCNYL1	0.004219958
MIR548Q	0.004389956
LINC01272	0.004479955
RETNLB	0.004629954
CAPRIN1	0.004639954
ISG20	0.004659953
LOC102723373	0.004689953
CSF2	0.004739953
MYO1F	0.004739953
LINGO3	0.004809952
SNORA5C	0.004879951
ATP1B2	0.005059949
POGLUT1	0.005249948
SNORA5A	0.005319947
SNORA5B	0.005319947
SPAG6	0.005429946
OSTM1	0.005439946
ZPR1	0.005449946
NFYC	0.005469945
PARP8	0.005509945
HIST1H3F	0.005539945
SAA4	0.005679943

GENE_NAME	P-value
PCSK7	0.005689943
MIR6830	0.005699943
NFYC-AS1	0.005879941
FBXO5	0.00596994
CATIP-AS1	0.00599994
CENPF	0.00602994
CYB561D1	0.006119939
FPR2	0.006229938
CALY	0.006259937
RPL13AP20	0.006269937
HIST1H2BH	0.006299937
HIST1H4G	0.006299937
LZTS1	0.006299937
IGLON5	0.006419936
RARS	0.006439936
RASEF	0.006629934
FAM83H	0.006649934
FAM83H-AS1	0.006709933
MIR5681A	0.006709933
PPARGC1A	0.006719933
SLC22A4	0.006789932
BLVRA	0.006829932
SAA2-SAA4	0.006829932
PIP4K2A	0.006889931
TPSB2	0.006889931
BPIFB2	0.00696993
MIR5681B	0.00701993
KRCC1	0.007059929
YTHDC2	0.007149929
TRPV4	0.007219928
BUD13	0.007229928
ZNF708	0.007239928
ZNF738	0.007279927
FGL1	0.007289927
SLC22A14	0.007339927
PWRN2	0.007379926
ZNF438	0.007449926
SNORA9	0.007459925
FLJ30679	0.007489925
MAPK15	0.007579924
CLCN2	0.007709923
LINC01255	0.007709923
RBP4	0.007709923
LOC101929154	0.007739923
MIR4743	0.007759922
RNF223	0.007759922
MIR4664	0.007769922
AGBL4-IT1	0.007809922
THUMPD2	0.007839922

GENE_NAME	P-value
LOC494141	0.007899921
FAM90A1	0.00798992
TPSG1	0.00803992
SOWAHB	0.00804992
SAA2	0.008129919
SNHG15	0.008169918
ZBTB40	0.008309917
CPNE7	0.008369916
GALNT14	0.008379916
POLR2J	0.008499915
LINC00645	0.008719913
PADI4	0.008749913
MIA2	0.008819912
LINC01093	0.00889911
POLR2H	0.00889911
LRWD1	0.0089991
RHOC	0.0089991
AGBL4	0.00909909
LOC101929584	0.00909909
LOC553103	0.00909909
TMCO3	0.00909909
MIR2052	0.009119909
LINC01258	0.00919908
PRAM1	0.00919908
SUN5	0.00919908
TSGA10IP	0.00919908
MIR4432	0.009259907
GRIN2C	0.00939906
LTB4R	0.009409906
MYRF	0.009469905
CHORDC1	0.00959904
MIR3936	0.00959904
PPP5C	0.00959904
MIR5090	0.00969903
ZNF414	0.00969903
FUOM	0.00979902
BCAM	0.00989901
ING2	0.00989901
PHF14	0.009999
