# Supplementary material for: Whole-genome sequencing analysis of semi-supercentenarians
Source: eLife. 2021 May 4;10:e57849. doi: 10.7554/eLife.57849 (PMC8096429; doi:10.7554/eLife.57849)
Supplement: Supplementary file 3. [file elife-57849-supp3.pdf]

Table3S. GTEx analysis for the 4 SNPs rs10279856, rs3779059, rs849166, rs849175 with credible score > 0 in the Riviera analysis.

Gencode Id	Gene Symbol	Variant Id	SNP	P-Value	Effect Size	Tissue
ENSG00000239556.2	AC004951.5	7_43760206_T_A_b37	rs849166	0.000047	-0.26	Esophagus - Mucosa
ENSG00000106605.6	BLVRA	7_43643835_G_A_b37	rs10279856	0.000015	0.26	Artery - Tibial
ENSG00000106605.6	BLVRA	7_43643835_G_A_b37	rs10279856	0.000000096	0.28	Esophagus - Mucosa
ENSG00000106605.6	BLVRA	7_43643835_G_A_b37	rs10279856	0.0000024	-0.17	Whole Blood
ENSG00000106605.6	BLVRA	7_43720429_G_A_b37	rs3779059	0.000026	-0.25	Artery - Tibial
ENSG00000106605.6	BLVRA	7_43720429_G_A_b37	rs3779059	0.00000011	-0.28	Esophagus - Mucosa
ENSG00000106605.6	BLVRA	7_43720429_G_A_b37	rs3779059	0.0000015	0.17	Whole Blood
ENSG00000106605.6	BLVRA	7_43747968_G_A_b37	rs849175	0.00000057	-0.27	Esophagus - Mucosa
ENSG00000106605.6	BLVRA	7_43747968_G_A_b37	rs849175	0.0000013	0.17	Whole Blood
ENSG00000106605.6	BLVRA	7_43760206_T_A_b37	rs849166	0.000017	-0.26	Artery - Tibial
ENSG00000106605.6	BLVRA	7_43760206_T_A_b37	rs849166	0.00000038	-0.27	Esophagus - Mucosa
ENSG00000106605.6	BLVRA	7_43760206_T_A_b37	rs849166	0.00000087	0.18	Whole Blood
ENSG00000106603.13	COA1	7_43643835_G_A_b37	rs10279856	0.000047	0.21	Adipose - Subcutaneous
ENSG00000106603.13	COA1	7_43643835_G_A_b37	rs10279856	0.0000001	0.28	Artery - Aorta
ENSG00000106603.13	COA1	7_43643835_G_A_b37	rs10279856	0.000000013	0.31	Artery - Tibial
ENSG00000106603.13	COA1	7_43643835_G_A_b37	rs10279856	0.000013	0.25	Esophagus - Mucosa
ENSG00000106603.13	COA1	7_43643835_G_A_b37	rs10279856	0.00000053	0.33	Esophagus - Muscularis
ENSG00000106603.13	COA1	7_43643835_G_A_b37	rs10279856	0.000018	0.27	Nerve - Tibial
ENSG00000106603.13	COA1	7_43643835_G_A_b37	rs10279856	0.000000028	0.28	Skin - Not Sun Exposed (Suprapubic)
ENSG00000106603.13	COA1	7_43643835_G_A_b37	rs10279856	9.2E-12	0.3	Skin - Sun Exposed (Lower leg)
ENSG00000106603.13	COA1	7_43720429_G_A_b37	rs3779059	0.000053	-0.21	Adipose - Subcutaneous
ENSG00000106603.13	COA1	7_43720429_G_A_b37	rs3779059	0.00000015	-0.27	Artery - Aorta
ENSG00000106603.13	COA1	7_43720429_G_A_b37	rs3779059	0.000000037	-0.3	Artery - Tibial
ENSG00000106603.13	COA1	7_43720429_G_A_b37	rs3779059	0.0000018	-0.27	Esophagus - Mucosa
ENSG00000106603.13	COA1	7_43720429_G_A_b37	rs3779059	0.0000012	-0.32	Esophagus - Muscularis
ENSG00000106603.13	COA1	7_43720429_G_A_b37	rs3779059	0.000025	-0.26	Nerve - Tibial
ENSG00000106603.13	COA1	7_43720429_G_A_b37	rs3779059	0.00000004	-0.28	Skin - Not Sun Exposed (Suprapubic)
ENSG00000106603.13	COA1	7_43720429_G_A_b37	rs3779059	4.6E-11	-0.29	Skin - Sun Exposed (Lower leg)

Gencode Id	Gene Symbol	Variant Id	SNP	P-Value	Effect Size	Tissue
ENSG00000106603.13	COA1	7_43747968_G_A_b37	rs849175	0.000000044	-0.28	Artery - Aorta
ENSG00000106603.13	COA1	7_43747968_G_A_b37	rs849175	0.000000021	-0.31	Artery - Tibial
ENSG00000106603.13	COA1	7_43747968_G_A_b37	rs849175	0.00003	-0.24	Esophagus - Mucosa
ENSG00000106603.13	COA1	7_43747968_G_A_b37	rs849175	0.00000046	-0.34	Esophagus - Muscularis
ENSG00000106603.13	COA1	7_43747968_G_A_b37	rs849175	0.00001	-0.27	Nerve - Tibial
ENSG00000106603.13	COA1	7_43747968_G_A_b37	rs849175	0.000000016	-0.29	Skin - Not Sun Exposed (Suprapubic)
ENSG00000106603.13	COA1	7_43747968_G_A_b37	rs849175	1.3E-10	-0.29	Skin - Sun Exposed (Lower leg)
ENSG00000106603.13	COA1	7_43760206_T_A_b37	rs849166	0.000046	-0.21	Adipose - Subcutaneous
ENSG00000106603.13	COA1	7_43760206_T_A_b37	rs849166	0.000000086	-0.28	Artery - Aorta
ENSG00000106603.13	COA1	7_43760206_T_A_b37	rs849166	0.000000015	-0.31	Artery - Tibial
ENSG00000106603.13	COA1	7_43760206_T_A_b37	rs849166	0.0000069	-0.26	Esophagus - Mucosa
ENSG00000106603.13	COA1	7_43760206_T_A_b37	rs849166	0.0000012	-0.33	Esophagus - Muscularis
ENSG00000106603.13	COA1	7_43760206_T_A_b37	rs849166	0.000017	-0.27	Nerve - Tibial
ENSG00000106603.13	COA1	7_43760206_T_A_b37	rs849166	0.000000029	-0.29	Skin - Not Sun Exposed (Suprapubic)
ENSG00000106603.13	COA1	7_43760206_T_A_b37	rs849166	2.2E-10	-0.28	Skin - Sun Exposed (Lower leg)
ENSG00000136206.3	SPDYE1	7_43643835_G_A_b37	rs10279856	0.000045	0.49	Spleen
ENSG00000136206.3	SPDYE1	7_43747968_G_A_b37	rs849175	0.000014	-0.49	Spleen
ENSG00000164543.5	STK17A	7_43643835_G_A_b37	rs10279856	0.000001	-0.5	Heart - Atrial Appendage
ENSG00000164543.5	STK17A	7_43643835_G_A_b37	rs10279856	5.5E-15	-0.48	Heart - Left Ventricle
ENSG00000164543.5	STK17A	7_43643835_G_A_b37	rs10279856	5.7E-14	-0.33	Lung
ENSG00000164543.5	STK17A	7_43643835_G_A_b37	rs10279856	1.8E-10	-0.33	Thyroid
ENSG00000164543.5	STK17A	7_43720429_G_A_b37	rs3779059	0.0000004	0.51	Heart - Atrial Appendage
ENSG00000164543.5	STK17A	7_43720429_G_A_b37	rs3779059	1.7E-14	0.47	Heart - Left Ventricle
ENSG00000164543.5	STK17A	7_43720429_G_A_b37	rs3779059	1.1E-14	0.34	Lung
ENSG00000164543.5	STK17A	7_43720429_G_A_b37	rs3779059	9E-10	0.32	Thyroid
ENSG00000164543.5	STK17A	7_43747968_G_A_b37	rs849175	0.0000057	0.47	Heart - Atrial Appendage
ENSG00000164543.5	STK17A	7_43747968_G_A_b37	rs849175	3.5E-14	0.46	Heart - Left Ventricle
ENSG00000164543.5	STK17A	7_43747968_G_A_b37	rs849175	1.9E-12	0.31	Lung
ENSG00000164543.5	STK17A	7_43747968_G_A_b37	rs849175	0.000097	0.2	Nerve - Tibial
ENSG00000164543.5	STK17A	7_43747968_G_A_b37	rs849175	5.2E-10	0.32	Thyroid
ENSG00000164543.5	STK17A	7_43760206_T_A_b37	rs849166	0.00000012	0.53	Heart - Atrial Appendage
ENSG00000164543.5	STK17A	7_43760206_T_A_b37	rs849166	1.7E-15	0.48	Heart - Left Ventricle

Gencode Id	Gene Symbol	Variant Id	SNP	P-Value	Effect Size	Tissue
ENSG00000164543.5	STK17A	7_43760206_T_A_b37	rs849166	1.5E-14	0.34	Lung
ENSG00000164543.5	STK17A	7_43760206_T_A_b37	rs849166	5.5E-10	0.32	Thyroid
