# Supplementary material for: Whole-genome sequencing analysis of semi-supercentenarians
Source: eLife. 2021 May 4;10:e57849. doi: 10.7554/eLife.57849 (PMC8096429; doi:10.7554/eLife.57849)
Supplement: Supplementary file 4. — The analysis has been performed considering all the annotated common variants in Cohort 1. [file elife-57849-supp4.pdf]

Table 4S. Significant (FDR<0.05) KEGG pathways involved in longevity identified by iGSEA4GWAS software. The analysis has been performed considering all the annotated common variants in Cohort 1.

Pathway	Gene Set Name	P-value	FDR value
KEGG	AXON GUIDANCE	0.001	1.00E-03
KEGG	CALCIUM SIGNALING PATHWAY	0.001	1.00E-03
KEGG	GLYCINE SERINE AND THREONINE METABOLISM	0.001	0.001
KEGG	LONG TERM POTENTIATION	0.001	0.001
KEGG	MELANOGENESIS	0.001	0.001
KEGG	PPAR SIGNALING PATHWAY	0.001	0.001
KEGG	TASTE TRANSDUCTION	0.001	0.001037
KEGG	O GLYCAN BIOSYNTHESIS	0.001	0.00104
KEGG	DILATED CARDIOMYOPATHY	0.001	0.001176
KEGG	MTOR SIGNALING PATHWAY	0.001	0.001383
KEGG	NATURAL KILLER CELL MEDIATED CYTOTOXICITY	0.001	0.001432
KEGG	T CELL RECEPTOR SIGNALING PATHWAY	0.001	0.0015
KEGG	CARDIAC MUSCLE CONTRACTION	0.001	0.001516
KEGG	PURINE METABOLISM	0.001	0.001981
KEGG	LONG TERM DEPRESSION	0.001	0.002048
KEGG	CHEMOKINE SIGNALING PATHWAY	0.001	0.002216
KEGG	HYPERTROPHIC CARDIOMYOPATHY HCM	0.001	0.002672
KEGG	VASOPRESSIN REGULATED WATER REABSORPTION	0.002	0.003628
KEGG	JAK STAT SIGNALING PATHWAY	0.002	0.010869
KEGG	PROGESTERONE MEDIATED OOCYTE MATURATION	0.001	0.014443
KEGG	ARRHYTHMOGENIC RIGHT VENTRICULAR CARDIOMYOPATHY ARVC	0.003	0.022281
KEGG	FOCAL ADHESION	0.009	0.030066
KEGG	ADIPOCYTOKINE SIGNALING PATHWAY	0.011	0.032412
KEGG	FRUCTOSE AND MANNOSE METABOLISM	0.024	0.049648
