# Supplementary material for: Whole-genome sequencing analysis of semi-supercentenarians
Source: eLife. 2021 May 4;10:e57849. doi: 10.7554/eLife.57849 (PMC8096429; doi:10.7554/eLife.57849)
Supplement: Supplementary file 5. — The analysis has been performed considering all the annotated common variants. [file elife-57849-supp5.pdf]

Table 5S. Significant (FDR<0.05) GO pathways involved in longevity identified by iGSEA4GWAS software. The analysis has been performed considering all the annotated common variants in Cohort 1.

Pathway	Gene Set Name	P-value	FDR value
GO	NUCLEOBASENUCLEOSIDENUCLEOTIDE AND NUCLEIC ACID TRANSPORT	0.001	1.00E-03
GO	NEGATIVE REGULATION OF CELL CYCLE	0.001	1.00E-03
GO	POSITIVE REGULATION OF MULTICELLULAR ORGANISMAL PROCESS	0.001	1.00E-03
GO	POSITIVE REGULATION OF DEVELOPMENTAL PROCESS	0.001	1.00E-03
GO	REGULATION OF HEART CONTRACTION	0.001	1.00E-03
GO	COVALENT CHROMATIN MODIFICATION	0.001	1.00E-03
GO	GENERATION OF A SIGNAL INVOLVED IN CELL CELL SIGNALING	0.001	1.00E-03
GO	PROTEIN PROCESSING	0.001	1.00E-03
GO	NEURON DEVELOPMENT	0.001	1.00E-03
GO	CENTRAL NERVOUS SYSTEM DEVELOPMENT	0.001	1.00E-03
GO	NEGATIVE REGULATION OF BIOSYNTHETIC PROCESS	0.001	1.00E-03
GO	LIPID BINDING	0.001	1.00E-03
GO	STRUCTURAL MOLECULE ACTIVITY	0.001	1.00E-03
GO	REGULATION OF SECRETION	0.001	0.001
GO	NEURITE DEVELOPMENT	0.001	0.001
GO	STRIATED MUSCLE DEVELOPMENT	0.001	0.001
GO	LIPID TRANSPORT	0.001	0.001
GO	AXONOGENESIS	0.001	0.001
GO	CELLULAR MORPHOGENESIS DURING DIFFERENTIATION	0.001	0.001
GO	SENSORY PERCEPTION	0.001	0.001
GO	HISTONE MODIFICATION	0.001	0.001
GO	NEURON DIFFERENTIATION	0.001	0.001
GO	NEGATIVE REGULATION OF TRANSLATION	0.001	0.001
GO	REGULATION OF MULTICELLULAR ORGANISMAL PROCESS	0.001	0.001
GO	HEMATOPOIETIN INTERFERON CLASSD200 DOMAIN CYTOKINE RECEPTOR BINDING	0.001	0.001
GO	PROTEIN TYROSINE PHOSPHATASE ACTIVITY	0.001	0.001
GO	PHOSPHORIC MONOESTER HYDROLASE ACTIVITY	0.001	0.001
GO	VOLTAGE GATED CHANNEL ACTIVITY	0.001	0.001
GO	STRUCTURAL CONSTITUENT OF RIBOSOME	0.001	0.001
GO	PHOSPHORIC ESTER HYDROLASE ACTIVITY	0.001	0.001
GO	VOLTAGE GATED CATION CHANNEL ACTIVITY	0.001	0.001
GO	CALCIUM CHANNEL ACTIVITY	0.001	0.001
GO	PHOSPHOPROTEIN PHOSPHATASE ACTIVITY	0.001	0.001
GO	STRUCTURAL CONSTITUENT OF CYTOSKELETON	0.001	0.001
GO	LIPID TRANSPORTER ACTIVITY	0.001	0.001
GO	GROWTH FACTOR ACTIVITY	0.001	0.001
GO	LIPID RAFT	0.001	0.001
GO	TRANSPORT VESICLE	0.001	0.001
GO	NEGATIVE REGULATION OF CELLULAR PROTEIN METABOLIC PROCESS	0.001	0.001032258
GO	REGULATION OF PROTEIN AMINO ACID PHOSPHORYLATION	0.001	0.001033333
GO	MUSCLE DEVELOPMENT	0.001	0.001033898
GO	REGULATION OF CELLULAR PROTEIN METABOLIC PROCESS	0.001	0.001034483
GO	SUBSTRATE SPECIFIC CHANNEL ACTIVITY	0.001	0.001035088
GO	NEGATIVE REGULATION OF CELLULAR BIOSYNTHETIC PROCESS	0.001	0.001035714
GO	SKELETAL DEVELOPMENT	0.001	0.001036364
GO	SKELETAL MUSCLE DEVELOPMENT	0.001	0.001037736
GO	MONOVALENT INORGANIC CATION TRANSPORT	0.001	0.001038462
GO	POSITIVE REGULATION OF CELL PROLIFERATION	0.001	0.001039216
GO	GATED CHANNEL ACTIVITY	0.001	0.001040816
GO	ION CHANNEL ACTIVITY	0.001	0.001041667
GO	NEGATIVE REGULATION OF CELL PROLIFERATION	0.001	0.001044118
GO	PROTEIN HOMODIMERIZATION ACTIVITY	0.001	0.001044776
GO	AUXILIARY TRANSPORT PROTEIN ACTIVITY	0.001	0.001045455
GO	G PROTEIN COUPLED RECEPTOR ACTIVITY	0.001	0.001046154
GO	ESTABLISHMENT AND OR MAINTENANCE OF CHROMATIN ARCHITECTURE	0.001	0.001046875
GO	CHROMATIN BINDING	0.001	0.001047619
GO	CELL CELL ADHESION	0.001	0.001101449
GO	CATION CHANNEL ACTIVITY	0.001	0.00115493
GO	VOLTAGE GATED POTASSIUM CHANNEL ACTIVITY	0.001	0.001178082
GO	POTASSIUM CHANNEL ACTIVITY	0.001	0.001253333
GO	ONE CARBON COMPOUND METABOLIC PROCESS	0.001	0.001289474

Pathway	Gene Set Name	P-value	FDR value
GO	GENERATION OF NEURONS	0.001	0.001333333
GO	LIGAND DEPENDENT NUCLEAR RECEPTOR ACTIVITY	0.001	0.001341772
GO	REGULATION OF CELL CYCLE	0.001	0.001378049
GO	DIGESTION	0.001	0.00139759
GO	CATION TRANSPORT	0.001	0.001404762
GO	CHROMATIN MODIFICATION	0.001	0.001406977
GO	POSITIVE REGULATION OF IMMUNE SYSTEM PROCESS	0.001	0.001411765
GO	POSITIVE REGULATION OF LYMPHOCYTE ACTIVATION	0.001	0.001426966
GO	PHOSPHOLIPID BINDING	0.001	0.001433333
GO	GENERATION OF PRECURSOR METABOLITES AND ENERGY	0.001	0.001436782
GO	POSITIVE REGULATION OF CELLULAR COMPONENT ORGANIZATION AND BIOGENESIS	0.001	0.001461539
GO	METHYLTRANSFERASE ACTIVITY	0.001	0.001489796
GO	PROTEIN SECRETION	0.001	0.001494845
GO	ACTIN CYTOSKELETON	0.001	0.001505263
GO	REGULATION OF PROTEIN METABOLIC PROCESS	0.001	0.001510638
GO	PROTEIN C TERMINUS BINDING	0.001	0.001646465
GO	REGULATION OF PHOSPHORYLATION	0.001	0.00177
GO	CELL MIGRATION	0.001	0.001841584
GO	HUMORAL IMMUNE RESPONSE	0.001	0.001843137
GO	REGULATION OF CELL ADHESION	0.001	0.001980769
GO	NEUROGENESIS	0.001	0.002130841
GO	LEUKOCYTE ACTIVATION	0.001	0.002165138
GO	CELL SURFACE	0.001	0.002166667
GO	ADHERENS JUNCTION	0.001	0.0022
GO	STRUCTURAL CONSTITUENT OF MUSCLE	0.001	0.002232143
GO	SECRETION	0.003	0.002265487
GO	TRANSFERASE ACTIVITY TRANSFERRING ONE CARBON GROUPS	0.001	0.002382609
GO	POTASSIUM ION TRANSPORT	0.001	0.002394737
GO	SUGAR BINDING	0.002	0.002413793
GO	RHODOPSIN LIKE RECEPTOR ACTIVITY	0.001	0.002416667
GO	TRANSMEMBRANE RECEPTOR PROTEIN TYROSINE KINASE SIGNALING PATHWAY	0.001	0.002418803
GO	BRAIN DEVELOPMENT	0.001	0.002420168
GO	CASPASE ACTIVATION	0.001	0.002423729
GO	DEVELOPMENT OF PRIMARY SEXUAL CHARACTERISTICS	0.001	0.002483607
GO	NEGATIVE REGULATION OF DEVELOPMENTAL PROCESS	0.001	0.002487603
GO	INTERPHASE	0.001	0.0025
GO	NEGATIVE REGULATION OF PROTEIN METABOLIC PROCESS	0.003	0.002622047
GO	METAL ION TRANSMEMBRANE TRANSPORTER ACTIVITY	0.001	0.002632
GO	RESPONSE TO NUTRIENT LEVELS	0.001	0.002634921
GO	CATION TRANSMEMBRANE TRANSPORTER ACTIVITY	0.001	0.003261539
GO	LYMPHOCYTE DIFFERENTIATION	0.001	0.003389313
GO	ION TRANSPORT	0.001	0.00344697
GO	METAL ION TRANSPORT	0.001	0.003514925
GO	REGULATION OF LYMPHOCYTE ACTIVATION	0.001	0.003595588
GO	TRANSMEMBRANE RECEPTOR PROTEIN TYROSINE KINASE ACTIVITY	0.001	0.003614815
GO	CELL CYCLE ARREST GO 0007050	0.001	0.00392029
GO	ENZYME ACTIVATOR ACTIVITY	0.001	0.004007194
GO	MOLECULAR ADAPTOR ACTIVITY	0.001	0.004205674
GO	TRANSCRIPTION ACTIVATOR ACTIVITY	0.002	0.004214285
GO	NEGATIVE REGULATION OF CELL DIFFERENTIATION	0.001	0.004267606
GO	ANION TRANSMEMBRANE TRANSPORTER ACTIVITY	0.001	0.004293706
GO	CARBOHYDRATE BINDING	0.001	0.004537931
GO	CYTOPLASMIC VESICLE	0.002	0.005226028
GO	PROTEIN AUTOPROCESSING	0.001	0.005238095
GO	SERINE TYPE ENDOPEPTIDASE ACTIVITY	0.004	0.005858108
GO	REGULATION OF BODY FLUID LEVELS	0.002	0.006120805
GO	POSITIVE REGULATION OF RESPONSE TO STIMULUS	0.001	0.006178808
GO	VESICLE	0.002	0.006193333
GO	REGULATION OF CELL DIFFERENTIATION	0.001	0.006611842
GO	ELECTRON TRANSPORT GO 0006118	0.003	0.007051948
GO	PROTEIN BINDING BRIDGING	0.003	0.007052288
GO	LYMPHOCYTE ACTIVATION	0.002	0.007296775
GO	NEGATIVE REGULATION OF PROGRAMMED CELL DEATH	0.002	0.007358974
GO	SECOND MESSENGER MEDIATED SIGNALING	0.003	0.007745223
GO	NEGATIVE REGULATION OF CATALYTIC ACTIVITY	0.003	0.008189874

Pathway	Gene Set Name	P-value	FDR value
GO	REGULATION OF TRANSLATION	0.004	0.008886793
GO	PROTEIN TYROSINE KINASE ACTIVITY	0.002	0.008993789
GO	TRANSLATION	0.007	0.009246914
GO	NUCLEAR EXPORT	0.002	0.009490798
GO	PROTEIN DOMAIN SPECIFIC BINDING	0.001	0.009789157
GO	PROTEIN DIMERIZATION ACTIVITY	0.002	0.0098
GO	APOPTOTIC PROGRAM	0.004	0.010473054
GO	CYTOPLASMIC MEMBRANE BOUND VESICLE	0.003	0.01083432
GO	POSITIVE REGULATION OF NUCLEOBASENUCLEOSIDENUCLEOTIDE AND NUCLEIC ACID METABOLIC PROCESS	0.002	0.011117647
GO	POSITIVE REGULATION OF CELL DIFFERENTIATION	0.001	0.011768786
GO	NEGATIVE REGULATION OF APOPTOSIS	0.002	0.011770115
GO	REGULATION OF PROTEIN MODIFICATION PROCESS	0.002	0.011795322
GO	REGULATION OF IMMUNE SYSTEM PROCESS	0.003	0.012011299
GO	EXOCYTOSIS	0.003	0.01322472
GO	CAMP MEDIATED SIGNALING	0.006	0.013544444
GO	ORGAN MORPHOGENESIS	0.003	0.013585636
GO	PROTEIN AMINO ACID DEPHOSPHORYLATION	0.002	0.014076923
GO	CYTOSKELETAL PROTEIN BINDING	0.004	0.014081967
GO	PROTEIN COMPLEX ASSEMBLY	0.001	0.014326086
GO	RESPONSE TO EXTRACELLULAR STIMULUS	0.005	0.014424732
GO	SH3 SH2 ADAPTOR ACTIVITY	0.006	0.01502139
GO	TRANSMEMBRANE RECEPTOR PROTEIN KINASE ACTIVITY	0.004	0.015398936
GO	PROTEIN AMINO ACID AUTOPHOSPHORYLATION	0.002	0.015502646
GO	INTERPHASE OF MITOTIC CELL CYCLE	0.003	0.01613158
GO	MAGNESIUM ION BINDING	0.005	0.016193718
GO	PROTEIN OLIGOMERIZATION	0.005	0.016380208
GO	MEMBRANE BOUND VESICLE	0.004	0.016466321
GO	INDUCTION OF APOPTOSIS BY INTRACELLULAR SIGNALS	0.006	0.016726803
GO	CHROMATIN	0.005	0.016866667
GO	I KAPPAB KINASE NF KAPPAB CASCADE	0.004	0.017806122
GO	SERINE HYDROLASE ACTIVITY	0.01	0.01790863
GO	CYTOKINE METABOLIC PROCESS	0.005	0.018530302
GO	T CELL ACTIVATION	0.005	0.01858794
GO	ELECTRON CARRIER ACTIVITY	0.002	0.018760001
GO	POSITIVE REGULATION OF PHOSPHORYLATION	0.003	0.019059703
GO	GTPASE ACTIVATOR ACTIVITY	0.005	0.01920197
GO	SERINE TYPE PEPTIDASE ACTIVITY	0.008	0.01920297
GO	RESPONSE TO WOUNDING	0.004	0.019441176
GO	HOMEOSTATIC PROCESS	0.004	0.020731708
GO	CYTOKINE ACTIVITY	0.007	0.020771844
GO	REGULATION OF T CELL ACTIVATION	0.006	0.021275362
GO	HORMONE ACTIVITY	0.007	0.021932691
GO	HEMOSTASIS	0.009	0.022105262
GO	POSITIVE REGULATION OF TRANSCRIPTION	0.005	0.02235849
GO	TRANSLATION FACTOR ACTIVITY NUCLEIC ACID BINDING	0.012	0.022436019
GO	REGULATION OF CYTOKINE BIOSYNTHETIC PROCESS	0.007	0.022497652
GO	DEPHOSPHORYLATION	0.003	0.022878503
GO	TRANSCRIPTION COFACTOR ACTIVITY	0.007	0.022995347
GO	CHROMOSOME ORGANIZATION AND BIOGENESIS	0.005	0.024709677
GO	CELL ACTIVATION	0.005	0.025192661
GO	SPHINGOLIPID METABOLIC PROCESS	0.003	0.025618182
GO	MICROTUBULE BINDING	0.005	0.025689498
GO	TISSUE DEVELOPMENT	0.006	0.02580543
GO	CYTOKINE PRODUCTION	0.004	0.026536034
GO	ANTI APOPTOSIS	0.008	0.02695964
GO	INFLAMMATORY RESPONSE	0.006	0.027125001
GO	CHROMOSOME SEGREGATION	0.009	0.029568888
GO	RNA POLYMERASE II TRANSCRIPTION FACTOR ACTIVITY	0.004	0.030563878
GO	REGULATION OF MAP KINASE ACTIVITY	0.005	0.030881578
GO	BONE REMODELING	0.01	0.031513043
GO	POSITIVE REGULATION OF CATALYTIC ACTIVITY	0.008	0.032506492
GO	SYNAPSE	0.011	0.032512933
GO	POSITIVE REGULATION OF TRANSFERASE ACTIVITY	0.012	0.033068378
GO	POSITIVE REGULATION OF CASPASE ACTIVITY	0.013	0.033948936
GO	LYTIC VACUOLE	0.006	0.035405062

Pathway	Gene Set Name	P-value	FDR value
GO	LYSOSOME	0.006	0.035405062
GO	GLYCOPROTEIN METABOLIC PROCESS	0.01	0.038836133
GO	RECEPTOR MEDIATED ENDOCYTOSIS	0.01	0.039230127
GO	NEGATIVE REGULATION OF GROWTH	0.012	0.04075
GO	G PROTEIN SIGNALING COUPLED TO CAMP NUCLEOTIDE SECOND MESSENGER	0.016	0.040800832
GO	NEGATIVE REGULATION OF TRANSCRIPTION FROM RNA POLYMERASE II PROMOTER	0.009	0.042355374
GO	ENDOPEPTIDASE ACTIVITY	0.018	0.044094652
GO	BASEMENT MEMBRANE	0.005	0.04452049
GO	EMBRYONIC DEVELOPMENT	0.012	0.044865303
GO	TUBULIN BINDING	0.019	0.045465585
GO	POSITIVE REGULATION OF TRANSCRIPTION FROM RNA POLYMERASE II PROMOTER	0.012	0.046798386
GO	WOUND HEALING	0.016	0.04945783
