# Supplementary material for: Whole-genome sequencing analysis of semi-supercentenarians
Source: eLife. 2021 May 4;10:e57849. doi: 10.7554/eLife.57849 (PMC8096429; doi:10.7554/eLife.57849)
Supplement: Supplementary file 6. — The analysis has been performed considering all the annotated common variants. [file elife-57849-supp6.pdf]

Table 6S. Significant (FDR<0.05) BioCarta pathways involved in longevity identified by iGSEA4GWAS software. The analysis has been performed considering all the annotated common variants in Cohort 1.

**Pathway**	**Gene Set Name**	**P-value**	**FDR value**
BioCarta	GPCR PATHWAY	0.001	0.001
BioCarta	TCR PATHWAY	0.001	0.001021
BioCarta	EDG1 PATHWAY	0.001	0.001022
BioCarta	CXCR4 PATHWAY	0.001	0.001033
BioCarta	FMLP PATHWAY	0.001	0.001143
BioCarta	BCR PATHWAY	0.001	0.001181
BioCarta	NO1 PATHWAY	0.001	0.001325
BioCarta	ECM PATHWAY	0.001	0.001375
BioCarta	VEGF PATHWAY	0.001	0.001467
BioCarta	TPO PATHWAY	0.002	0.002038
BioCarta	MET PATHWAY	0.002	0.00248
BioCarta	HER2 PATHWAY	0.001	0.002969
BioCarta	HDAC PATHWAY	0.001	0.003451
BioCarta	VIP PATHWAY	0.001	0.004299
BioCarta	INFLAM PATHWAY	0.001	0.008987
BioCarta	INTEGRIN PATHWAY	0.003	0.009561
BioCarta	DEATH PATHWAY	0.003	0.011779
BioCarta	GH PATHWAY	0.003	0.011931
BioCarta	NFAT PATHWAY	0.002	0.012062
BioCarta	PYK2 PATHWAY	0.002	0.013408
BioCarta	NTHI PATHWAY	0.005	0.022912
BioCarta	HIVNEF PATHWAY	0.009	0.030978
BioCarta	CARM ER PATHWAY	0.015	0.045028
