# Supplementary material for: Whole-genome sequencing analysis of semi-supercentenarians
Source: eLife. 2021 May 4;10:e57849. doi: 10.7554/eLife.57849 (PMC8096429; doi:10.7554/eLife.57849)
Supplement: Supplementary file 7. — The allele associated with a longer lifespan is reported as ‘Protective allele’. Chromosome, position (GRCH 37/hg19), rs ID, gene name, protective allele, frequency in semi-supercentenarians, frequency in controls and nominal p-values were reported. [file elife-57849-supp7.pdf]

Table 7S. Comparison of allele frequency for a subset of known longevity variants. The allele associated with a longer lifespan is reported as "Protective allele". Chromosome, position (GRCH 37/hg19), rs ID, gene name, protective allele, frequency in semi-supercentenarians, frequency in controls and nominal p-values were reported.

CHR	BP	SNP_ID	GENE	Protective allele	F_105+	F_CTRL	Nominal p-value
6	108908518	rs2802292	FOXO3A	G	0.49	0.53	0.62
6	108999287	rs1935949	FOXO3A	A	0.36	0.39	0.81
7	22766645	rs1800795	IL6	C	0.34	0.31	0.65
9	21998035	rs2811712	CDKN2A	G	0.12	0.13	0.88
10	69643342	rs3758391	SIRT1	T	0.25	0.33	0.15
15	99451976	rs34516635	IGF1R	A	0	0	na
16	57016092	rs5882	CETP	G	0.28	0.4	0.09
17	7579472	rs1042522	TP53	C	0.76	0.6	0.01
