# Supplementary material for: Whole-genome sequencing analysis of semi-supercentenarians
Source: eLife. 2021 May 4;10:e57849. doi: 10.7554/eLife.57849 (PMC8096429; doi:10.7554/eLife.57849)
Supplement: Supplementary file 8. — The table reported six columns with the description of the SNP, chromosome, position in hg19, minor allele, gene name and the trend of allele frequency in different age groups as described in Giuliani et al., 2018b (Class A, B, E, and F, see legend). From columns 7–10 allele frequencies in Cohort 2 is reported as published in Giuliani et al., 2018b. The p-value of the association test performed between 105+/110+ and CTRL (Cohort 1) is reported in the last column. [file elife-57849-supp8.pdf]

**Table 8S.** Comparison between the SNPs described in Giuliani et al 2018 to the present study. The table reported 6 columns with the description of the SNP, chromosome, position in hg19, minor allele, gene name and the trend of allele frequency in different age groups as described in Giuliani et al 2018 (Class A, B, E and F, see legend). From column 7 to 10 allele frequencies in Cohort 2 is reported as published in Giuliani et al 2018. The p-value of the association test performed between 105+/110+ and CTR (Cohort 1) is reported in the last column.

| SNP description | | | | | | Cohort 2 (data from Giuliani et al 2018) | | | | Cohort 1 | |
|---|---|---|---|---|---|---|---|---|---|---|---|
| **CHR** | **Position (hg19)** | **SNP** | **A1** | **GENE_NAME** | **Class paper Giuliani et al 2018 (#)** | **MAF_AGE_MIN50** | **MAF_AGE_MAG50** | **MAF_CENT** | **Fisher_combined_Pval** | **P-value 105+/110+ vs CTR (Garagnani 2020)** | |
| 1 | 217005202 | rs1436897 | G | ESRRG | ClassA_Giuliani2018 | 0.3559 | 0.3942 | 0.3288 | 2.02E-05 | 0.02594 | |
| 2 | 47740566 | rs876937 | G | | ClassB_Giuliani2018 | 0.3913 | 0.3287 | 0.3694 | 2.45E-05 | 0.04623 | |
| 7 | 43864699 | rs623108 | A | | ClassB_Giuliani2018 | 0.3941 | 0.3398 | 0.3979 | 2.96E-04 | 0.0005474 | |
| 11 | 76415209 | rs10899257 | A | GUCY2EP | ClassB_Giuliani2018 | 0.1569 | 0.1351 | 0.1547 | 1.70E-04 | 0.03773 | |
| 12 | 127917085 | rs10444466 | A | | ClassB_Giuliani2018 | 0.2092 | 0.1643 | 0.2312 | 6.79E-06 | 0.006454 | |
| 17 | 54157181 | rs11650687 | C | | ClassB_Giuliani2018 | 0.3133 | 0.2682 | 0.3063 | 6.25E-04 | 0.0287 | |
| 17 | 54164935 | rs9901788 | G | | ClassB_Giuliani2018 | 0.3026 | 0.2612 | 0.3106 | 2.15E-04 | 0.02037 | |
| 3 | 60056853 | rs2630196 | G | FHIT | ClassE_Giuliani2018 | 0.2704 | 0.2869 | 0.2417 | 4.09E-05 | 0.01726 | |
| 3 | 60053331 | rs2630173 | A | FHIT | ClassE_Giuliani2018 | 0.2685 | 0.2869 | 0.2417 | 6.65E-05 | 0.01726 | |
| 20 | 8144719 | rs10485720 | G | PLCB1 | ClassE_Giuliani2018 | 0.25 | 0.2521 | 0.1997 | 1.79E-04 | 0.02917 | |
| 6 | 85417046 | rs13215600 | A | | ClassF_Giuliani2018 | 0.1416 | 0.1671 | 0.2072 | 2.20E-06 | 0.02266 | |
| 6 | 85462640 | rs860844 | G | TBX18 | ClassF_Giuliani2018 | 0.1339 | 0.1634 | 0.1982 | 1.01E-05 | 0.02597 | |
| 6 | 157199844 | rs17266366 | G | ARID1B | ClassF_Giuliani2018 | 0.2717 | 0.2632 | 0.3363 | 3.84E-05 | 0.02188 | |
| 7 | 45122723 | rs61740895 | A | NACAD | ClassF_Giuliani2018 | 0.3367 | 0.3254 | 0.4114 | 7.58E-06 | 0.02228 | |
| 7 | 45124465 | rs3735495 | A | NACAD | ClassF_Giuliani2018 | 0.3393 | 0.3273 | 0.4129 | 5.11E-06 | 0.02228 | |
| 17 | 66670126 | rs11654241 | G | | ClassF_Giuliani2018 | 0.199 | 0.2061 | 0.2432 | 1.16E-04 | 0.03569 | |
| 19 | 9228196 | rs1036224 | A | OR7G1 | ClassF_Giuliani2018 | 0.2609 | 0.2646 | 0.3408 | 4.44E-04 | 0.01891 | |
| 6 | 85462640 | rs860844 | G | TBX18 | All_Giuliani2018 | 0.147 | | 0.1982 | 0.0000693 | 0.02597 | identified in HRS |
| 7 | 45122723 | rs61740895 | A | NACAD | All_Giuliani2019 | 0.329 | | 0.4114 | 0.0000219 | 0.02228 | identified in HRS |
| 7 | 45124286 | rs3735494 | G | NACAD | All_Giuliani2020 | 0.3323 | | 0.4114 | 0.0000238 | 0.02228 | identified in HRS |
| 7 | 45124465 | rs3735495 | A | NACAD | All_Giuliani2021 | 0.3312 | | 0.4129 | 0.0000152 | 0.02228 | identified in HRS |

***(#) Legend***

*Class A: SNPs for which CTR > 50 years old showed higher allele frequencies than CTR < 50 years and centenarians, while similar allele frequencies were observed in centenarians and CTR < 50 years old.*

*Class B: SNPs for which CTR > 50 years old showed lower allele frequencies than CTR < 50 years old and centenarians, while similar allele frequencies in centenarians and CTR < 50 years old were observed.*

*Class E: these variants significantly decreased in frequency in centenarians, while CTR < 50 years old and CTR > 50 years old showed similar frequencies.*

*Class F: these variants significantly increased in frequency in centenarians, while CTR < 50 years old and CTR > 50 years old showed similar frequencies.*
