# Supplementary material for: Whole-genome sequencing analysis of semi-supercentenarians
Source: eLife. 2021 May 4;10:e57849. doi: 10.7554/eLife.57849 (PMC8096429; doi:10.7554/eLife.57849)
Supplement: Supplementary file 9. [file elife-57849-supp9.pdf]

Table 9S. List of 5055 105+/110+ private mutations predicted as damaging in more than 4 (out of 6) database (SIFT Pred, Polyphen2 HVAR Pred, MutationTaster Pred, MutationAssessor Pred, FATHMM Pred, FATHMM MKL Coding Pred)

Chr.Pos	Ref.Alt	Identifier.1	Gene.Names
1:871262	G/A	rs1276148894	SAMD11
1:897330	C/T	rs150391309	KLHL17
1:898153	C/A	rs147703918	KLHL17
1:985937	C/T	rs151104255	AGRN
1:989305	C/T	rs748598591	AGRN
1:1231862	C/T	rs563840123	ACAP3
1:1234016	G/A	rs369561716	ACAP3
1:1267729	T/C	rs370117959	TAS1R3
1:1271553	T/A	rs763319960	DVL1
1:1323407	G/C	rs142455256	CCNL2
1:1372488	G/C	rs1192825439	VWA1
1:1606967	G/A	rs756610571	SLC35E2B
1:1647885	C/T	rs1336369850	CDK11A
1:2235533	G/A	rs530007354	SKI
1:2433629	C/T	rs189336246	PLCH2
1:2522487	G/A	rs143215062	MMEL1,PRXL2B
1:2523053	C/T	rs138709620	MMEL1
1:2524107	C/T	rs146100312	MMEL1
1:2527495	C/T	rs373436929	MMEL1
1:2938369	G/T		ACTRT2
1:2938887	G/A	rs61745278	ACTRT2
1:2939259	C/T	rs753889830	ACTRT2
1:3342734	A/G	rs1356695446	PRDM16
1:3380112	G/A	rs1031473979	ARHGEF16
1:3397137	G/A	rs145970985	ARHGEF16
1:3413317	C/T		MEGF6
1:3418428	G/A	rs200472001	MEGF6
1:3426554	C/T	rs1037487591	MEGF6
1:3427461	C/T	rs760974680	MEGF6
1:3431168	G/A	rs199566066	MEGF6
1:3644322	G/A	rs61737710	TP73
1:3646620	A/G		TP73
1:3649322	C/A		TP73
1:3649420	C/G		TP73
1:3649528	G/A	rs376856688	TP73
1:3765188	G/A	rs201531250	CEP104
1:4772034	C/T	rs149284997	AJAP1
1:5935048	G/A	rs569364202	NPHP4
1:5937230	G/A	rs373603595	NPHP4
1:6100648	C/T	rs1351483053	KCNAB2
1:6311473	G/A	rs764688053	GPR153
1:6313887	C/T	rs143332760	GPR153
1:6488329	G/A	rs367832657	ESPN
1:6504665	C/T	rs747761267	ESPN

1:6512061	G/A	rs121908135	ESPN
1:6530391	C/T	rs200641225	PLEKHG5
1:6533339	G/A	rs376782083	PLEKHG5
1:6688573	G/A		THAP3
1:8384718	C/T		SLC45A1
1:8420615	G/T	rs565894522	RERE
1:9171336	G/A	rs144609953	GPR157
1:9305499	G/A	rs765328943	H6PD
1:9804566	G/A	rs564647610	CLSTN1
1:9932101	C/T	rs113935062	CTNNBIP1
1:10683165	C/A	rs145867351	PEX14
1:10713997	G/A	rs61736955	CASZ1
1:10714261	G/A	rs142418398	CASZ1
1:11139829	C/T	rs748531214	EXOSC10
1:11272478	T/C	rs151082401	MTOR
1:11584058	A/G	rs756533821	DISP3
1:11591015	C/G	rs778972601	DISP3
1:11710576	A/T		FBXO2
1:11887224	G/C	rs775989333	CLCN6
1:12059085	G/A	rs140234726	MFN2
1:12785377	C/T		AADACL3
1:13839944	T/C		LRRC38
1:14068596	G/A		PRDM2
1:15382771	G/A	rs199776092	KAZN
1:15707803	T/C		FHAD1
1:16069648	T/C	rs146908578	TMEM82
1:16070767	C/T	rs148343257	TMEM82
1:16070958	G/A	rs375882436	TMEM82
1:16254768	G/A	rs1355240501	SPEN
1:16357048	G/A	rs772885800	CLCNKA
1:16374499	G/A		CLCNKB
1:16477423	G/C	rs147977279	EPHA2
1:16577668	C/T	rs143311144	FBXO42
1:16577716	T/C	rs1057483349	FBXO42
1:16727361	C/A	rs141634535	SPATA21
1:17256422	C/T	rs545352105	CROCC
1:17257060	C/T	rs572385634	CROCC
1:17296904	C/T	rs774166280	CROCC
1:17975148	C/T	rs141216141	ARHGEF10L
1:19166151	C/T	rs752505410	TAS1R2
1:19612697	G/C		AKR7A3
1:19630723	C/T	rs765069207	AKR7A2
1:20073668	C/T	rs770828902	TMCO4
1:20416328	G/C		PLA2G5
1:20501587	G/A	rs1204197671	PLA2G2C
1:20972189	G/A	rs200729715	PINK1
1:21050165	C/T	rs764616867	SH2D5
1:21050596	T/C	rs758595773	SH2D5
1:21616691	T/G	rs771219479	ECE1
1:21894605	G/T	rs776117933	ALPL

1:21903983	G/A		ALPL
1:22168831	C/T	rs1185082010	HSPG2
1:22207217	G/C		HSPG2
1:22816668	A/G	rs372233615	ZBTB40
1:23759611	G/A	rs200686258	ASAP3
1:23885841	C/T	rs765414579	ID3
1:24080617	A/C	rs144826294	ELOA
1:24397632	T/C	rs377309347	MYOM3
1:24409160	C/A	rs750814730	MYOM3
1:24424415	C/T	rs139828169	MYOM3
1:24434973	G/A	rs762184862	MYOM3
1:24663192	T/C	rs1186587606	GRHL3
1:25554663	C/T	rs34639798	SYF2
1:25715468	G/A	rs781384610	RHCE
1:26104704	G/A	rs769693980	MAN1C1
1:26299138	A/G	rs199892319	PAFAH2
1:26317303	C/T	rs148837170	PAFAH2
1:26369096	C/T	rs746355195	SLC30A2
1:26387820	G/A	rs199936688	TRIM63
1:26387823	C/T	rs775364417	TRIM63
1:26582316	A/G	rs768286565	CEP85
1:27268299	C/T	rs202085543	NUDC
1:27320701	G/A		TRNP1
1:27427750	G/A	rs140853773	SLC9A1
1:27690857	G/C		MAP3K6
1:28209484	C/T	rs201614111	THEMIS2
1:28275667	T/C		SMPDL3B
1:28282314	A/G		SMPDL3B
1:28290139	C/T	rs147855072	XKR8
1:28786737	A/G	rs571300371	PHACTR4
1:28806968	C/T		PHACTR4
1:28807091	C/T		PHACTR4
1:29139049	A/G	rs111622802	OPRD1
1:29344900	C/G	rs202197419	EPB41
1:29527085	C/A	rs34835902	MECR
1:29585169	T/A		PTPRU
1:31347330	G/C	rs748073439	SDC3
1:31452995	G/A	rs1294868563	PUM1
1:31467949	T/C	rs373459171	PUM1
1:31532214	C/A	rs761956967	PUM1
1:31898217	G/A	rs140666908	SERINC2
1:32140894	C/T	rs746549833	COL16A1
1:32557607	G/A	rs202154275	TMEM39B
1:33560210	C/T	rs140229682	AZIN2
1:33560216	G/A	rs141134478	AZIN2
1:33583634	G/A		AZIN2
1:33960171	T/G	rs35642856	ZSCAN20
1:33998761	C/G		CSMD2
1:34008479	G/C	rs1487701994	CSMD2
1:34034977	G/A	rs143459383	CSMD2

1:34076667	C/T	rs142970674	CSMD2
1:34330008	G/C		CSMD2,HMGB4
1:34330076	G/A		CSMD2,HMGB4
1:35453687	T/C	rs754877635	ZMYM6
1:36214026	A/C		CLSPN
1:36552436	G/A	rs116893490	TEKT2
1:36564663	C/T		COL8A2
1:36644602	G/T		MAP7D1
1:36937121	G/C		CSF3R
1:38027233	G/A	rs771741737	DNALI1
1:38155366	G/T	rs145196420	C1orf109
1:38397417	G/A	rs201779932	INPP5B
1:39734458	G/A	rs906957683	MACF1
1:39797461	C/T	rs140978060	MACF1
1:39797509	G/A	rs370428216	MACF1
1:39893249	A/G		MACF1
1:40105297	T/C	rs201783171	HEYL
1:40131883	A/G		NT5C1A
1:40211072	G/A	rs149451835	PPIE
1:40527407	G/A	rs771719133	CAP1
1:40557772	G/A		PPT1
1:41579092	C/T	rs1385160784	SCMH1
1:41847610	C/G	rs963754449	FOXO6
1:42922999	A/G		PPCS
1:43131694	C/T	rs142436431	PPIH
1:43647252	C/T	rs755893459	CFAP57
1:43777350	G/A	rs56302794	TIE1
1:43814619	G/T	rs780160063	MPL
1:44134806	G/A	rs566945580	KDM4A
1:44402203	G/C	rs762302784	ARTN
1:44422021	C/T	rs147434747	IPO13
1:45140056	G/C	rs1339294324	TMEM53
1:45407273	A/T	rs1164638252	EIF2B3
1:45981416	A/G	rs142590526	PRDX1
1:46035599	C/T	rs145543542	AKR1A1
1:46487731	G/A		MAST2
1:46496783	G/A	rs563202707	MAST2
1:46658206	G/A	rs1571655768	POMGNT1
1:46740268	T/C	rs28363243	RAD54L
1:46879171	C/T		FAAH
1:46976854	G/A	rs372261765	DMBX1
1:47028449	C/T	rs764595768	MKNK1
1:47101587	T/C		ATPAF1
1:47278174	G/A	rs148753850	CYP4B1
1:49224628	T/A	rs747657670	AGBL4,BEND5
1:51787808	G/T	rs765707131	TTC39A
1:52231541	G/A	rs374143753	OSBPL9
1:52747360	C/T	rs142219419	ZFYVE9
1:52851608	G/A	rs3087481	ORC1
1:52941118	G/A	rs369413824	TUT4

1:53072389	G/A	rs750935402	GPX7
1:53416457	A/G	rs138573524	SCP2
1:53559211	C/T	rs142744203	SLC1A7
1:54337063	C/T	rs764033322	YIPF1
1:54694471	G/A		SSBP3
1:55078210	A/G	rs368152719	ACOT11,FAM151A
1:55139471	C/T	rs138838053	MROH7
1:55161125	G/A	rs200130362	MROH7
1:55518374	C/T	rs148195424	PCSK9
1:55587134	A/C	rs758195596	USP24
1:57209853	C/G	rs140047407	FYB2
1:59139321	C/T	rs199719141	MYSM1
1:60019796	G/T	rs142088608	FGGY
1:62234985	C/T	rs536926846	PATJ
1:63021621	A/G	rs35400360	DOCK7
1:63284763	T/G		ATG4C
1:64034086	T/C	rs149252955	EFCAB7
1:65099902	A/G	rs200696750	CACHD1
1:65113598	T/A	rs1243669918	CACHD1
1:65145385	C/T	rs369700188	CACHD1
1:65845158	A/G	rs770344233	DNAJC6
1:67242971	G/A	rs751531763	TCTEX1D1
1:67895733	G/T		SERBP1
1:70502234	A/G	rs761783275	LRRC7
1:70504612	C/G		LRRC7
1:76215192	A/G	rs201375579	ACADM
1:76272729	G/C		MSH4
1:76878036	A/G		ST6GALNAC3
1:77632494	G/A	rs374050300	PIGK
1:78098238	A/G		ZZZ3
1:78408274	T/G	rs199738750	NEXN
1:79387320	A/C	rs185967608	ADGRL4
1:82372825	G/A		ADGRL2
1:82417671	G/A	rs193155723	ADGRL2
1:82417722	A/G		ADGRL2
1:82434977	A/G	rs759301278	ADGRL2
1:82450379	A/G		ADGRL2
1:85555822	C/T	rs149091067	WDR63
1:85648238	C/T	rs762947899	SYDE2
1:86340342	G/A	rs200448368	COL24A1
1:86375680	G/A	rs781239648	COL24A1
1:86437045	G/A	rs768842736	COL24A1
1:86591826	G/C	rs1027427440	COL24A1
1:86905988	A/T	rs145061827	CLCA2
1:87030974	C/A	rs200272807	CLCA4
1:87040284	A/G	rs200785098	CLCA4
1:89414921	T/C	rs527950397	KYAT3
1:89732049	C/A	rs771123564	GBP5
1:89847321	G/A	rs1203252285	GBP6
1:91177958	G/A	rs369760372	BARHL2

1:91973426	T/C	rs769338114	CDC7
1:92149345	C/T	rs1187231599	TGFBR3
1:93160880	A/G	rs143611208	EVI5
1:93163529	T/G	rs200747400	EVI5
1:93651954	A/C	rs771766725	CCDC18
1:94047943	G/A	rs748486479	BCAR3
1:94471055	C/T	rs61750641	ABCA4
1:94543278	G/A	rs138157885	ABCA4
1:94998715	G/C	rs200192517	F3
1:95293119	G/A	rs536412204	SLC44A3
1:95330361	A/G	rs140072535	SLC44A3
1:95357903	G/A		SLC44A3
1:95710231	C/G	rs201731120	RWDD3,TLCD4-RWDD3
1:95712352	G/A	rs369779655	RWDD3
1:100346211	C/T	rs139488862	AGL
1:100551145	C/A		SASS6
1:100575980	A/T		SASS6
1:100843122	C/T	rs754007075	CDC14A
1:103481285	C/T	rs149558726	COL11A1
1:107691435	G/T		NTNG1
1:109289390	A/C		STXBP3
1:109444449	G/C	rs370245661	GPSM2
1:109794316	C/T	rs138919468	CELSR2
1:109813204	G/A	rs184626807	CELSR2
1:109840856	G/A	rs201587393	MYBPHL
1:110146080	T/C	rs745377564	GNAT2
1:110146166	C/T	rs200224608	GNAT2
1:110168026	A/G		AMPD2
1:110200400	A/C	rs745500600	GSTM4
1:110257765	A/G		GSTM5
1:110280921	C/T	rs146952826	GSTM3
1:110754728	G/A		KCNC4
1:110882077	G/T	rs770679512	RBM15
1:112042939	T/A	rs755987513	ADORA3,TMIGD3
1:113153619	C/G	rs140260708	ST7L
1:113231655	T/C	rs752675873	MOV10
1:113239399	A/G	rs747373547	MOV10
1:113254606	C/T	rs377437744	PPM1J
1:114442813	C/T	rs1301678999	AP4B1
1:114515967	G/A	rs368547650	HIPK1
1:114680329	G/T	rs199947720	SYT6
1:115220592	C/T	rs781528554	AMPD1
1:116380899	A/C		NHLH2
1:117564491	G/A	rs200228819	CD101
1:117626760	A/G	rs144713709	TTF2
1:117660805	T/A	rs200213016	TRIM45
1:117663624	C/A		TRIM45
1:117699342	C/G		VTCN1
1:118426243	A/C		GDAP2
1:118430349	A/G	rs986371211	GDAP2

1:119929383	G/A	rs758059487	HAO2
1:120301801	G/A	rs773945291	HMGCS2
1:146736109	C/T	rs587674066	CHD1L
1:146758135	C/A	rs149913688	CHD1L
1:147131530	A/T	rs146298808	ACP6
1:149858147	G/C		H2BC21
1:149920922	C/A	rs192984583	OTUD7B
1:150116954	A/G	rs782644893	VPS45
1:150325417	C/T		PRPF3
1:150483454	G/T	rs781095324	ECM1
1:150808879	C/T	rs774247572	ARNT
1:150935095	A/G	rs587629539	SETDB1
1:150955890	T/C	rs587597900	ANXA9
1:150991126	G/A	rs773618224	PRUNE1
1:151090601	G/A		GABPB2
1:151108993	T/G	rs141765997	SEMA6C
1:151139877	C/T	rs144369082	SCNM1,TNFAIP8L2-SCNM1
1:151491032	C/T	rs780503507	CGN
1:151491054	T/G		CGN
1:151508742	G/A	rs372439240	CGN
1:153652107	C/T	rs145002753	NPR1
1:153659131	G/A	rs61758562	NPR1
1:154186402	T/C		C1orf43
1:154218711	C/T	rs915769840	UBAP2L
1:154437843	T/A	rs765750844	IL6R
1:154461589	G/A	rs1326318861	SHE
1:154999189	G/C	rs144159530	DCST2
1:155005719	A/G	rs139391176	DCST2
1:155160698	A/T	rs35819649	MUC1
1:155165906	T/C	rs756201460	THBS3
1:155165916	C/T		THBS3
1:155174703	G/A	rs748198784	THBS3
1:155252609	G/A		HCN3
1:155255611	C/T	rs146290600	HCN3
1:155291583	G/A	rs766941422	RUSC1
1:155584026	G/A	rs764937683	MSTO1
1:155630428	G/A	rs1206341871	YY1AP1
1:155698891	C/T	rs748394462	DAP3
1:155736274	C/T	rs759286454	GON4L
1:155912200	C/T	rs41264991	RXFP4
1:155935163	G/C		ARHGEF2
1:156127862	T/C	rs149652495	SEMA4A
1:156206143	C/T	rs149553436	PMF1,PMF1-BGLAP
1:156351621	G/C	rs772459487	RHBG
1:156626086	C/T	rs762992077	BCAN
1:156642658	A/G	rs868615713	NES
1:156707560	G/A		MRPL24
1:156830911	T/C	rs766307207	NTRK1
1:157068981	C/T	rs768731373	ETV3L
1:158368716	C/G	rs148758700	OR10T2

1:158549278	G/A	rs747790868	OR10X1
1:158592867	C/T	rs188580757	SPTA1
1:159799703	G/A	rs115535212	SLAMF8
1:159842773	C/T	rs142022446	CFAP45
1:159842782	C/T	rs377234702	CFAP45
1:159889502	G/A	rs140206246	TAGLN2
1:159897648	T/C		IGSF9
1:159898627	C/T	rs760663053	IGSF9
1:160000703	T/C	rs368135991	PIGM
1:160105381	G/C	rs147183887	ATP1A2
1:160171036	C/T	rs147541452	CASQ1
1:160394996	A/C	rs143298058	VANGL2
1:161021368	G/T	rs780211980	ARHGAP30
1:161127986	T/A	rs761045653	UFC1
1:161163164	C/T	rs746213424	ADAMTS4
1:161163466	G/A	rs147127522	ADAMTS4
1:161203095	C/T	rs531442743	NR1I3
1:161293444	C/G	rs1553261768	SDHC
1:161298190	G/T	rs754818119	SDHC
1:161495148	G/A	rs147002100	HSPA6
1:165386436	C/G	rs748751592	RXRG
1:167095777	C/T	rs144860364	DUSP27
1:167096874	C/T	rs141439169	DUSP27
1:167381220	A/C		POU2F1
1:169345927	C/G		BLZF1
1:169454821	T/G	rs760432864	SLC19A2
1:169555582	C/T	rs9332485	F5
1:169586281	C/T	rs72712022	SELP
1:169761758	T/A	rs13375701	METTL18
1:170513863	C/T	rs770077182	GORAB
1:171174762	G/C	rs28369899	FMO2
1:171514775	C/A	rs773951826	PRRC2C
1:171707536	G/A	rs535997142	VAMP4
1:175375804	C/A	rs1031445505	TNR
1:176668328	C/T	rs182517174	PAPPA2
1:177911150	C/T	rs149895580	SEC16B
1:177921119	G/A	rs112199919	SEC16B
1:178425926	C/T	rs779146098	RASAL2
1:178436488	G/A	rs144401857	RASAL2
1:178861391	C/T	rs775899673	RALGPS2
1:179521739	C/T	rs751767084	AXDND1,NPHS2
1:179526191	C/G	rs146906190	NPHS2
1:179975648	C/G	rs146044862	CEP350
1:180155276	C/T	rs189319097	QSOX1
1:180159679	G/A	rs200237514	QSOX1
1:180953863	C/T	rs141108606	STX6
1:181549750	C/A		CACNA1E
1:181765954	G/A	rs200113695	CACNA1E
1:182617421	G/A	rs778638489	RGS8
1:182781313	G/A	rs141892236	NPL

1:182874747	T/C	rs764660226	SHCBP1L
1:183079660	A/C	rs778839175	LAMC1
1:184777291	C/T	rs371190847	NIBAN1
1:185976348	C/T	rs143641249	HMCN1
1:186289484	A/C		TPR
1:192153431	A/G	rs758238355	RGS18
1:196963355	T/G	rs151134004	CFHR5
1:197059119	T/G		ASPM
1:197072538	C/A	rs373196055	ASPM
1:197111898	G/A		ASPM
1:197404390	G/A	rs116246250	CRB1
1:200378773	C/A	rs548362759	ZNF281
1:200613608	G/A	rs753527075	DDX59
1:200801464	T/C	rs377378492	CAMSAP2
1:200959415	G/A	rs763592461	KIF21B
1:201029943	C/T	rs1800559	CACNA1S
1:201029944	G/A	rs80338782	CACNA1S
1:201031099	G/A	rs200224590	CACNA1S
1:201047034	C/T	rs748711395	CACNA1S
1:201047133	C/T	rs150590855	CACNA1S
1:201170895	G/A	rs1209649019	IGFN1
1:202092219	G/A	rs778901003	GPR37L1
1:202126900	C/T	rs149783764	PTPN7
1:202574776	T/C	rs150707131	SYT2
1:202698977	T/C	rs746897616	KDM5B
1:202710517	C/G		KDM5B
1:203669953	G/A	rs759107186	ATP2B4
1:204092324	T/C	rs765882711	SOX13
1:204217972	C/T	rs527478614	PLEKHA6
1:204228558	G/A	rs147420132	PLEKHA6
1:204228665	G/A	rs201387028	PLEKHA6
1:204518499	C/G	rs61754765	MDM4
1:205027192	C/T	rs149564430	CNTN2
1:205027723	G/A	rs768629152	CNTN2
1:205031020	C/T	rs545256650	CNTN2
1:205129291	G/C		DSTYK
1:205180578	C/T	rs200780796	DSTYK
1:205273596	C/G		NUAK2
1:205274405	G/C	rs199839354	NUAK2
1:205632684	G/A	rs753484902	SLC45A3
1:206778778	G/C		EIF2D
1:207755267	C/T	rs534155454	CR1
1:208201458	T/A		PLXNA2
1:208213033	G/A	rs375437589	PLXNA2
1:208216457	C/T	rs777615301	PLXNA2
1:209778897	C/A		CAMK1G
1:209823425	G/A	rs115191959	LAMB3
1:210024625	A/T	rs768614100	UTP25
1:212118289	C/T		INTS7
1:212236261	A/G		DTL

1:212912892	A/T	rs747159160	NSL1
1:212981105	T/C	rs185603499	TATDN3
1:214556797	C/T	rs142432691	PTPN14
1:214794059	C/A	rs141892982	CENPF
1:215848910	G/A	rs111033275	USH2A
1:217793543	C/T	rs950192394	GPATCH2
1:219366452	T/G	rs761056754	LYPLAL1
1:219384901	T/C	rs570275364	LYPLAL1
1:220101509	G/A	rs1357141356	SLC30A10
1:220179510	T/C	rs150364999	EPRS1
1:220179599	T/A	rs747663156	EPRS1
1:220363774	C/T		RAB3GAP2
1:220369731	G/A	rs1455844030	RAB3GAP2
1:220955124	C/T	rs761979205	MTARC2
1:222838879	C/T	rs183067270	MIA3
1:223175886	C/T	rs371163462	DISP1
1:223722731	A/C	rs369121915	CAPN8
1:223943295	C/T	rs761658788	CAPN2
1:223990986	T/C	rs367908768	TP53BP2
1:224621761	C/T	rs964228171	WDR26
1:225288416	C/G		DNAH14
1:225328662	G/A		DNAH14
1:225332283	G/A	rs41307682	DNAH14
1:225702561	G/A	rs760881130	ENAH
1:226041377	G/A	rs75188792	TMEM63A
1:226044368	G/A	rs748261411	TMEM63A
1:227174263	T/A		COQ8A
1:227954731	C/A	rs773745138	SNAP47
1:228362481	A/G	rs770623717	IBA57
1:228464633	C/G	rs201269256	OBSCN
1:228505745	C/T	rs762117147	OBSCN
1:228527749	C/T	rs56065114	OBSCN
1:228528939	C/G	rs755543960	OBSCN
1:229666002	C/T		ABCB10
1:230472997	C/A	rs1046306416	PGBD5
1:230846470	G/A	rs41271499	AGT
1:230927671	G/A	rs140529301	CAPN9
1:231299608	G/C	rs1195378585	TRIM67
1:231935882	C/T	rs367543092	DISC1
1:232568020	T/C		SIPA1L2
1:232568146	A/C	rs201254048	SIPA1L2
1:233121929	G/A	rs778033025	PCNX2
1:233490587	T/A		MAP3K21
1:233511710	G/A	rs374695182	MAP3K21
1:234445050	G/C	rs748493581	SLC35F3
1:234529455	C/T	rs749220682	TARBP1
1:234599651	G/A		TARBP1
1:235316028	C/T	rs372663591	RBM34
1:235397741	T/G	rs763009081	ARID4B
1:235915340	C/T		LYST

1:236572518	C/T	rs762357435	EDARADD
1:236734652	G/A	rs935904378	HEATR1
1:236737979	C/G	rs369436014	HEATR1
1:236751313	C/T	rs1408711550	HEATR1
1:236902708	G/A	rs774046373	ACTN2
1:236917337	G/A	rs146164600	ACTN2
1:237774070	G/A	rs201675951	RYR2
1:238048865	G/C	rs745902324	ZP4
1:238049143	G/A	rs34811980	ZP4
1:238050120	C/T	rs141717126	ZP4
1:238050714	A/G	rs765775115	ZP4
1:241958566	T/C		WDR64
1:242023871	A/T	rs201509012	EXO1
1:242159682	C/A	rs768580624	MAP1LC3C
1:245772756	G/A	rs778508665	KIF26B
1:245775116	C/T	rs1463339956	KIF26B
1:246754962	C/T	rs201330696	CNST
1:247054333	C/T	rs200702767	AHCTF1
1:247695273	C/T	rs200581250	GCSAML,OR2C3
2:1926226	C/T	rs1191064463	MYT1L
2:3392003	C/A	rs199651906	TRAPPC12
2:3425730	G/A	rs550531429	TRAPPC12
2:3504602	A/G	rs201541392	ADI1
2:8919091	C/A		KIDINS220
2:9528649	C/T	rs75701656	ASAP2
2:9621442	T/G	rs151051276	IAH1
2:9991704	C/G		TAF1B
2:10188300	C/T		KLF11
2:10784467	C/G	rs148529864	NOL10
2:10942641	C/T	rs771820742	PDIA6
2:11696820	G/T	rs532741308	GREB1
2:11728938	C/T	rs151098036	GREB1
2:11728953	C/T	rs747899314	GREB1
2:11928520	A/T	rs751535374	LPIN1
2:11959618	T/C	rs749481129	LPIN1
2:15319203	G/A	rs760920114	NBAS
2:15374831	C/T	rs140373332	NBAS
2:15415865	T/A	rs201084909	NBAS
2:15415867	T/G	rs199717686	NBAS
2:15467943	C/T		NBAS
2:21260934	G/A	rs6752026	APOB
2:24344136	C/T	rs764889045	FAM228B,PFN4
2:24888620	C/T	rs769307134	NCOA1
2:25457173	A/T	rs751868166	DNMT3A
2:25536796	C/T	rs781254365	DNMT3A
2:25803650	G/C	rs368967650	DTNB
2:25965205	G/A		ASXL2
2:25965983	G/A	rs200946888	ASXL2
2:26457140	G/A		HADHA
2:26597965	C/T	rs140004348	SELENOI

2:26667184	C/T	rs145305515	DRC1
2:26688621	A/G	rs111033405	OTOF
2:26689977	C/A	rs761359291	OTOF
2:26699169	C/T	rs772312557	OTOF
2:27162984	C/T		DPYSL5
2:27258840	G/C	rs367766864	TMEM214
2:27303763	C/T	rs1221738236	EMILIN1
2:27352481	A/C		ABHD1
2:27375583	G/A	rs757395294	TCF23
2:27454921	G/A	rs370850388	CAD
2:27455356	A/G		CAD
2:27677464	A/C	rs202111577	IFT172
2:27681015	C/T	rs369780709	IFT172
2:27728585	C/T	rs745945801	GCKR
2:27884130	G/A	rs548044329	SUPT7L
2:29297120	C/T	rs1420546201	PCARE
2:29443676	G/A	rs56315533	ALK
2:31400008	C/A		CAPN14
2:31467155	G/C	rs138676775	EHD3
2:31588857	G/A	rs148585342	XDH
2:31590868	T/C		XDH
2:31602791	T/A	rs34929837	XDH
2:31624153	A/C	rs746230682	XDH
2:32361695	A/G		SPAST
2:32640012	G/C		BIRC6
2:32702461	G/A		BIRC6
2:33246017	C/T	rs377502460	LTBP1
2:33360013	C/T	rs754291598	LTBP1
2:33810258	C/T		FAM98A
2:33810451	G/C	rs147172208	FAM98A
2:37455553	G/C		CEBPZ
2:37474742	A/C		NDUFAF7
2:37599595	A/G	rs755896115	QPCT
2:38156650	A/G		RMDN2
2:38298394	C/T	rs79204362	CYP1B1
2:38302350	C/T	rs28936700	CYP1B1
2:38893338	T/C	rs201350001	GALM
2:39053138	T/G		DHX57
2:39233616	C/G	rs369277679	SOS1
2:42671159	T/C	rs1558372434	KCNG3
2:43015716	C/T	rs775376402	HAAO
2:43451577	G/A	rs748600797	ZFP36L2
2:43805716	G/T	rs373077677	THADA
2:43937198	C/T	rs149301613	PLEKHH2
2:44031852	G/A	rs142412169	DYNC2LI1
2:44055202	A/G	rs769667259	ABCG5
2:44078806	G/T		ABCG8
2:44079509	C/T	rs376362072	ABCG8
2:44079828	A/T		ABCG8
2:44079965	G/T	rs749564472	ABCG8

2:44128597	C/T	rs372374729	LRPPRC
2:45233478	G/A	rs372153489	SIX2
2:46583923	C/T		EPAS1
2:46739447	C/T	rs142304043	ATP6V1E2
2:47639682	C/T	rs587781294	MSH2
2:48030646	C/T	rs63750753	MSH6
2:48807816	A/T	rs141632429	STON1,STON1-GTF2A1L
2:48808647	G/A	rs762534739	STON1,STON1-GTF2A1L
2:48808774	G/C	rs143139624	STON1,STON1-GTF2A1L
2:48897029	G/T		GTF2A1L,STON1-GTF2A1L
2:49190602	C/T	rs1181865489	FSHR
2:50280648	G/A		NRXN1
2:54163247	C/G		PSME4
2:55093976	G/A	rs149815344	EML6
2:55119761	G/A		EML6
2:55252261	G/T	rs139414628	RTN4
2:55252334	T/C	rs199592685	RTN4
2:55253014	C/T	rs139542847	RTN4
2:55462625	C/G		RPS27A
2:55467216	G/A	rs79235708	MTIF2
2:55756042	A/C	rs191927946	CFAP36
2:55792169	G/C	rs200351314	PPP4R3B
2:61259062	T/G	rs754783237	PEX13
2:61439033	A/T	rs768733068	USP34
2:61523960	G/A	rs185706364	USP34
2:62063237	C/T	rs868226745	FAM161A
2:62228048	G/T	rs1171291356	COMMD1
2:63206344	C/T	rs143970378	EHBP1
2:64682532	G/A	rs145173493	LGALSL
2:64682792	G/A	rs1351483704	LGALSL
2:64778749	C/A	rs113401509	AFTPH
2:67626390	A/G	rs143819253	ETAA1
2:68273541	A/G		C1D
2:68621249	C/T	rs752019384	PLEK
2:68717350	A/T	rs147962002	APLF
2:69627587	C/A	rs201634470	NFU1
2:70408345	T/C		C2orf42
2:70409065	C/T		C2orf42
2:70502159	G/T		PCYOX1
2:71036947	C/T	rs201804036	CLEC4F
2:71297922	G/A	rs192172897	NAGK
2:71297944	A/T	rs150821125	NAGK
2:71376420	G/T	rs539937334	MPHOSPH10
2:71766298	C/T		DYSF
2:71886099	A/G	rs933715192	DYSF
2:71892323	C/T	rs758206608	DYSF
2:72359481	G/A	rs146011965	CYP26B1
2:72360268	G/A	rs144968323	CYP26B1
2:73114686	G/T	rs888988467	SPR
2:73195640	G/A	rs371438584	SFXN5

2:73487922	G/A	rs1027724234	FBXO41
2:73868577	C/G	rs142272948	NAT8
2:74011533	G/A		C2orf78
2:74141940	C/G	rs1316312229	ACTG2
2:74459656	C/A		SLC4A5
2:74593432	A/G	rs748563840	DCTN1
2:74594192	G/A	rs766338463	DCTN1
2:74641580	A/G	rs372622223	C2orf81
2:74688884	G/A	rs13405869	MOGS
2:74689924	G/A	rs1572920656	MOGS
2:74747111	G/A	rs143679936	DQX1
2:75916303	T/C	rs150597064	GCFC2
2:84756272	A/G	rs1436979374	DNAH6
2:84804515	C/A	rs149725294	DNAH6
2:84846883	A/T	rs367551290	DNAH6
2:85035596	G/A	rs200449901	DNAH6
2:85571210	C/T	rs147993013	RETSAT
2:85626333	G/A	rs11539103	CAPG
2:86297307	C/T	rs753139894	POLR1A
2:86359498	G/A	rs200147987	PTCD3
2:86364656	G/A	rs144366369	PTCD3
2:95540690	C/T	rs781938936	TEKT4
2:95815229	T/C	rs141510791	ZNF514
2:96780663	A/G	rs777661184	ADRA2B
2:96992463	C/T	rs752003922	ITPRIPL1
2:97527138	G/C	rs149174129	SEMA4C
2:98275868	G/A	rs374344795	ACTR1B
2:98430837	T/A	rs200161087	TMEM131
2:98828474	G/A	rs371420742	VWA3B
2:99012502	G/A	rs199837807	CNGA3
2:99012949	G/A	rs760376435	CNGA3
2:99778712	C/G	rs137973334	LIPT1
2:99778974	A/C	rs1462499605	LIPT1
2:100055218	T/C	rs754921264	REV1
2:100055445	C/G		REV1
2:101010185	G/A		CHST10
2:101650032	G/A	rs116402789	TBC1D8
2:101650115	T/A	rs1227890248	TBC1D8
2:102476165	C/G	rs377671536	MAP4K4
2:103340303	G/A	rs138186882	MFSD9
2:105713621	T/C		MRPS9
2:105924731	C/G	rs201941079	TGFBRAP1
2:105984191	G/A	rs140148322	FHL2
2:107029593	G/A	rs368408437	RGPD3
2:108475734	T/C	rs1219411065	RGPD4
2:108477339	C/T	rs1558806397	RGPD4
2:108482781	G/A	rs1348970330	RGPD4
2:108486339	T/C	rs1360897749	RGPD4
2:108487813	T/A	rs542293552	RGPD4
2:108499270	T/G		RGPD4

2:108921036	T/C	rs17036091	SULT1C2
2:109087307	A/G	rs200270126	GCC2
2:109365522	G/A	rs371319181	RANBP2
2:109368398	A/G	rs1329178183	RANBP2
2:110325541	G/A		SEPTIN10
2:110907820	A/G		NPHP1
2:111551731	A/G	rs201529913	ACOXL
2:111556206	C/T	rs371460199	ACOXL
2:112573215	C/T	rs1362086812	ANAPC1
2:112786053	T/C	rs377341255	MERTK
2:112922638	T/A	rs147767836	FBLN7
2:113260499	T/G		TTL
2:113520107	T/C	rs35593767	CKAP2L
2:119739786	G/T	rs147907250	MARCO
2:120020687	G/A	rs768319229	STEAP3
2:120231120	C/T	rs1488194173	SCTR
2:120387461	G/T	rs756941486	CFAP221
2:121712934	G/A	rs202141899	GLI2
2:121747143	G/A		GLI2
2:128044282	G/C		ERCC3
2:128331536	C/T	rs765756611	MYO7B
2:128331564	T/G	rs760261415	MYO7B
2:128394435	A/G	rs986487397	MYO7B
2:128415111	C/T	rs750865991	LIMS2
2:128466278	C/G	rs755836998	WDR33
2:128471199	C/A	rs754393530	WDR33
2:128471245	G/C	rs918581662	WDR33
2:128471416	C/G	rs145331578	WDR33
2:128757996	G/C	rs200102758	SAP130
2:130832866	G/C	rs771177382	POTEF
2:130872528	C/G		POTEF
2:130914202	G/C	rs138211555	SMPD4
2:130949490	C/T	rs766331053	TUBA3E
2:130951867	T/C		TUBA3E
2:131096791	C/T	rs756325067	CCDC115
2:131221280	C/A	rs1406731446	POTEI
2:131904275	G/A	rs1559117985	PLEKHB2
2:132238045	T/G	rs745914780	TUBA3D
2:133542417	C/G	rs377186508	NCKAP5
2:135616903	C/T	rs267598885	ACMSD
2:135744340	C/T	rs137859461	MAP3K19
2:135891531	G/A	rs144231223	RAB3GAP1
2:136073036	G/T	rs769045535	ZRANB3
2:136528277	G/A	rs371075349	UBXN4
2:137814709	C/T	rs138536184	THSD7B
2:138378281	A/G	rs757424980	THSD7B
2:141093334	G/A	rs139868893	LRP1B
2:141299369	C/T	rs150174082	LRP1B
2:144276873	C/T	rs776986852	ARHGAP15
2:144525614	T/C		ARHGAP15

2:144899504	T/C	rs1198491782	GTDC1
2:145147053	C/T	rs750844030	ZEB2
2:148696765	T/C	rs200239981	ORC4
2:149447799	G/A		EPC2
2:152406206	G/A	rs751724804	NEB
2:152466393	T/G	rs199683595	NEB
2:152483522	C/T	rs370053963	NEB
2:152490170	C/G		NEB
2:152496886	A/C	rs1480476721	NEB
2:152521046	G/A	rs373790988	NEB
2:153476095	C/T	rs578243842	FMNL2
2:153504339	G/A	rs773514322	FMNL2
2:158115891	A/G	rs779435698	GALNT5
2:160076262	A/G	rs201218896	TANC1
2:160114308	C/T	rs139910347	WDSUB1
2:160585546	C/T	rs760877357	MARCHF7
2:160621164	G/A	rs779836024	MARCHF7
2:161029145	C/T	rs576531469	ITGB6
2:161223755	C/T	rs753001447	RBMS1
2:162851876	T/C	rs200726335	DPP4
2:163144766	A/T		IFIH1
2:164466072	C/T	rs776699577	FIGN
2:166756324	C/G	rs749920942	TTC21B
2:166797556	T/A	rs149925563	TTC21B
2:166915170	G/A		SCN1A
2:167128911	C/A	rs200817435	SCN9A
2:167128958	A/C	rs200866100	SCN9A
2:167137073	A/T	rs200879772	SCN9A
2:167140988	A/G		SCN9A
2:167266352	T/A	rs766294815	SCN7A
2:168101056	G/A	rs551539824	XIRP2
2:168101294	T/G	rs199992905	XIRP2
2:169404120	C/G	rs146638314	CERS6
2:169684948	T/G		NOSTRIN
2:169833127	T/C	rs147522210	ABCB11
2:170031729	C/A		LRP2
2:170068598	C/T	rs138269726	LRP2
2:170099984	C/T	rs574423999	LRP2
2:170103223	C/G	rs544224269	LRP2
2:170131587	G/A		LRP2
2:170531549	C/A	rs201880958	CCDC173
2:170606007	A/G	rs745997628	KLHL23,PHOSPHO2-KLHL23
2:170662036	T/A		SSB
2:170770618	C/A		UBR3
2:170937099	C/G		UBR3
2:172180809	C/T	rs201005071	METTL8
2:172187117	A/C	rs143561498	METTL8
2:172966996	G/T	rs762068836	DLX2
2:173832165	C/A	rs369456567	RAPGEF4
2:175979420	A/C	rs761534267	ATF2

2:176803109	T/C	rs537138957	LNPK
2:176945076	G/A		EVX2
2:176972297	A/C	rs1193568203	HOXD11
2:176981827	G/A	rs374700658	HOXD10
2:176987959	G/C	rs191379716	HOXD9
2:177054620	A/G		HOXD1
2:178257642	C/T	rs1044647338	AGPS
2:178415856	C/T	rs768606470	TTC30B
2:178482651	A/G	rs535504630	TTC30A
2:178592456	T/C	rs77597060	PDE11A
2:178981107	T/G	rs145599521	RBM45
2:179204432	C/T	rs150008222	OSBPL6
2:179403769	C/G	rs186405108	TTN
2:179417586	C/T	rs1359003253	TTN
2:179419353	C/T	rs111727915	TTN
2:179422669	G/T	rs72648227	TTN
2:179429612	A/G	rs186273940	TTN
2:179430579	T/G	rs778292712	TTN
2:179430596	A/G	rs200181804	TTN
2:179434154	C/G		TTN
2:179440483	G/A	rs776457502	TTN
2:179442010	A/G	rs748860763	TTN
2:179442198	C/G	rs201381085	TTN
2:179446468	G/A	rs779303152	TTN
2:179448393	G/A	rs55948748	TTN
2:179451454	G/A	rs72646859	TTN
2:179454107	T/G	rs750969532	TTN
2:179469611	G/T		TTN
2:179477948	G/A		TTN
2:179554549	G/C	rs200213832	TTN
2:179554579	C/T	rs139790668	TTN
2:179587955	G/T	rs187925021	TTN
2:179597242	C/A	rs72648940	TTN
2:179629535	G/C		TTN
2:179634919	T/A	rs202024134	TTN
2:180835443	C/G	rs143785942	CWC22
2:183584818	G/C	rs756375421	DNAJC10
2:183622423	T/C		DNAJC10
2:189864262	G/C	rs267599125	COL3A1
2:190531277	G/A	rs200125189	ASNSD1
2:190541719	G/T		ANKAR
2:190742065	T/G		PMS1
2:191792077	G/T	rs140284772	GLS
2:192194696	G/A	rs755914659	MYO1B
2:196671483	C/A	rs6708527	DNAH7
2:196749328	T/A	rs376528845	DNAH7
2:196753016	G/A		DNAH7
2:198436794	G/A	rs200681405	RFTN2
2:200213607	C/A		SATB2
2:200828468	G/T	rs755276377	MAIP1

2:201524002	G/A	rs139975106	AOX1
2:202137392	A/G	rs148697064	CASP8
2:202744780	T/G	rs767334097	CDK15
2:202964466	C/T	rs1394388129	KIAA2012
2:204161510	T/A	rs141888883	CYP20A1
2:204320182	T/C	rs35068009	RAPH1
2:205989107	G/A	rs201211082	PARD3B
2:206364664	G/A	rs375059681	PARD3B
2:207006783	G/A	rs1181556411	NDUFS1
2:207008834	G/A	rs1440562589	NDUFS1
2:207460800	T/A		ADAM23
2:207559599	T/C	rs16838593	DYTN
2:207636985	C/T	rs149493338	FASTKD2
2:208994188	G/A	rs746609116	CRYGC
2:209302522	G/T	rs148656389	PTH2R
2:210843307	G/A		UNC80
2:210858960	C/T	rs368594548	UNC80
2:215797431	T/C	rs199846944	ABCA12
2:215818614	C/T	rs138995566	ABCA12
2:216002886	A/G		ABCA12
2:216199644	G/A	rs956941513	ATIC
2:216240047	G/A	rs139452116	FN1
2:216243915	C/T	rs777907579	FN1
2:217347521	G/A		SMARCAL1
2:218682610	C/T	rs765466438	TNS1
2:218682829	C/T	rs141992593	TNS1
2:218713379	G/A	rs758441152	TNS1
2:218745635	T/C	rs781427620	TNS1
2:218758179	C/T	rs149902330	TNS1
2:219137364	A/G	rs988131059	PNKD
2:219141822	C/T		PNKD,TMBIM1
2:219142696	G/A	rs142636664	PNKD,TMBIM1
2:219290494	G/A	rs746568854	VIL1
2:219483526	G/C	rs200863393	PLCD4
2:219492820	T/G	rs200161654	PLCD4
2:219503348	T/A	rs200133906	ZNF142
2:219507889	C/G		ZNF142
2:219757885	C/G	rs759671927	WNT10A
2:220088694	C/T	rs199900351	ATG9A
2:220098443	C/T	rs530025449	ANKZF1
2:220159790	G/T	rs777087069	PTPRN
2:220164086	G/A	rs375009720	PTPRN
2:220349660	G/T	rs1427721693	SPEG
2:220417338	G/A	rs944701875	OBSL1
2:220421379	G/A	rs200449388	OBSL1
2:220428290	C/T	rs770552541	OBSL1
2:220435534	G/A		OBSL1
2:222301252	G/C	rs200282869	EPHA4
2:224463150	C/T	rs778138097	SCG2
2:227660163	T/A	rs183414071	IRS1

2:227872225	G/T	rs778524414	COL4A4
2:227922238	G/A		COL4A4
2:227967507	G/A	rs200817090	COL4A4
2:228167818	G/A		COL4A3
2:228172522	G/A	rs746450762	COL4A3
2:230231598	C/T	rs143853534	DNER
2:230875554	G/A	rs145410117	FBXO36
2:231223704	T/C	rs764760652	SP140L
2:233244289	A/C	rs376639163	ALPP
2:233349576	G/A	rs1297891115	ECEL1
2:233394756	C/T	rs201733876	CHRND
2:233633499	C/T	rs757304681	GIGYF2,KCNJ13
2:234357944	C/T	rs113483143	DGKD
2:234545887	C/T	rs149598882	UGT1A8,UGT1A10
2:234602452	A/G		UGT1A6,UGT1A7,UGT1A8,UGT1A9,UGT1A10
2:234854575	C/T	rs199593973	TRPM8
2:234858626	T/C		TRPM8
2:234869481	G/A	rs267599275	TRPM8
2:234869523	C/T	rs76512385	TRPM8
2:235950405	A/G	rs780844581	SH3BP4
2:235961282	A/G		SH3BP4
2:236653367	T/C	rs758794882	AGAP1
2:238267213	G/T	rs369169235	COL6A3
2:238267718	A/G	rs1482422103	COL6A3
2:238303529	C/T	rs779126378	COL6A3
2:238664781	A/G	rs942848361	LRRFIP1
2:238785955	A/G	rs1044004510	RAMP1
2:238976763	G/C	rs747462994	SCLY
2:239009274	A/C		ESPNL
2:239049516	G/A	rs781523389	KLHL30
2:239164410	C/A	rs537238176	PER2
2:239257976	C/T	rs1300880230	TRAF3IP1
2:239307435	G/A	rs199665200	TRAF3IP1
2:239344384	G/T	rs199985152	ASB1
2:239990284	C/T	rs771662328	HDAC4
2:241463317	G/A	rs34267428	ANKMY1
2:241533413	G/C		CAPN10
2:241621990	C/T	rs202020511	AQP12B
2:241816972	C/T	rs180177290	AGXT
2:241829423	T/G		MAB21L4
2:242035595	G/A	rs376613445	MTERF4
2:242035726	T/C		MTERF4
2:242076639	C/T	rs148298829	PASK
2:242402756	C/T	rs377640265	FARP2
2:242757670	G/T	rs748488398	NEU4
3:3186367	A/G	rs754316396	TRNT1
3:4735407	C/G	rs61757110	ITPR1
3:4767234	C/T		ITPR1
3:5024660	C/G	rs765940624	BHLHE40
3:7348331	C/T	rs755371859	GRM7

3:8671375	C/A		SSUH2
3:9074366	G/A		SRGAP3
3:9406871	T/A		THUMPD3
3:9868894	C/T	rs142298876	ARPC4-TTLL3,TTLL3
3:9962175	G/A		IL17RC
3:9988053	G/A	rs772806301	PRRT3
3:10417250	G/A	rs149899981	ATP2B2
3:11075334	G/A	rs771439149	SLC6A1
3:11468362	G/C	rs768304604	ATG7
3:12195065	G/A	rs144326666	SYN2,TIMP4
3:12611734	T/G	rs757599810	MKRN2
3:12613661	C/T	rs768823128	MKRN2
3:12613834	C/T	rs141069425	MKRN2
3:12623670	T/A	rs201937482	MKRN2
3:12859065	G/T		CAND2
3:12872978	C/G		CAND2
3:12872988	C/T	rs770416028	CAND2
3:12957207	G/A	rs200895339	IQSEC1
3:13360744	G/A	rs749069796	NUP210
3:13383315	G/C	rs142731621	NUP210
3:13417847	C/A	rs1320367521	NUP210
3:13661319	G/A	rs200141144	FBLN2
3:13860896	C/T		WNT7A
3:15084357	G/A	rs761331134	NR2C2
3:16419576	C/T	rs151244908	RFTN1
3:16640068	G/C	rs61730117	DAZL
3:18390955	C/T	rs772952860	SATB1
3:19574904	G/C	rs775662074	KCNH8
3:20043007	C/T	rs201179163	PP2D1
3:23960943	G/A	rs764132899	RPL15
3:27472950	G/C		SLC4A7
3:27761802	A/G		EOMES
3:27763350	G/A	rs200215171	EOMES
3:32804323	C/T	rs374597639	CNOT10
3:33134922	G/A	rs751596344	GLB1,TMPPE
3:33195213	G/C	rs150528777	SUSD5
3:33453171	T/C	rs137944225	UBP1
3:33617723	C/G		CLASP2
3:37574852	C/T	rs199956894	ITGA9
3:38037265	A/T	rs139825675	VILL
3:38167343	C/T	rs151138541	ACAA1
3:38307500	G/A	rs771872547	SLC22A13
3:38592717	G/A	rs752995885	SCN5A
3:38645517	G/T		SCN5A
3:38655290	G/A	rs41276525	SCN5A
3:38674589	A/C	rs199473050	SCN5A
3:38739727	C/T	rs151090729	SCN10A
3:38743402	C/T	rs757916036	SCN10A
3:38760151	A/G	rs139638446	SCN10A
3:38763839	C/G	rs143744796	SCN10A

3:39129687	G/A	rs770608218	WDR48
3:39148995	C/T	rs541137496	GORASP1
3:39167864	A/T	rs140805783	TTC21A
3:39170386	T/C	rs375065952	TTC21A
3:39227755	G/A	rs35795536	XIRP1
3:39230720	C/T	rs149108228	XIRP1
3:40192598	G/A	rs138774955	MYRIP
3:40192666	G/A	rs142760932	MYRIP
3:40442406	G/A	rs766285710	ENTPD3
3:40570867	C/G	rs376456065	ZNF621
3:41746550	G/A	rs201639701	ULK4
3:42739091	G/C	rs61748826	HHATL
3:42794075	C/G	rs778747601	CCDC13
3:42794201	C/T	rs373266481	CCDC13
3:43121663	C/T	rs371278382	POMGNT2
3:43641902	G/A		ANO10
3:44488506	T/C		ZNF445
3:44685159	A/G		ZNF197
3:44905770	C/T	rs199793010	TMEM42
3:45152112	C/T	rs144577185	CDCP1
3:45812898	A/G	rs147760034	SLC6A20
3:46000037	C/A	rs375376263	FYCO1
3:46011195	A/G	rs1559462086	FYCO1
3:46306924	A/G	rs201293544	CCR3
3:46307064	G/A	rs145141172	CCR3
3:46871917	A/C	rs201320305	PRSS42P
3:47043203	C/G	rs534419583	NBEAL2
3:47045825	A/C	rs778613401	NBEAL2
3:47162834	A/G		SETD2
3:47165272	T/C		SETD2
3:47308548	C/T	rs772217849	KIF9
3:47859566	C/T	rs147205504	DHX30
3:47894356	G/A	rs375614055	MAP4
3:47950612	G/C	rs201731116	MAP4
3:48459903	G/A	rs775508033	PLXNB1
3:48475236	G/A	rs199514353	CCDC51
3:48498706	C/T	rs35240314	ATRIP
3:48506327	G/T		ATRIP
3:48607736	C/T	rs369221071	COL7A1
3:48626306	C/T	rs141649838	COL7A1
3:48668676	C/T	rs1468498109	SLC26A6
3:48717560	G/A	rs764660569	NCKIPSD
3:48916811	G/A	rs748394731	SLC25A20
3:49004582	C/T	rs746152958	ARIH2
3:49049226	T/G	rs1576615032	WDR6
3:49049827	G/T		WDR6
3:49062153	G/C		IMPDH2
3:49136795	G/C	rs774819969	QARS1
3:49568854	C/T	rs147149991	DAG1
3:49570006	C/T		DAG1

3:49570044	T/G	rs141697036	DAG1
3:49698316	C/T	rs140696444	BSN
3:49739000	C/T	rs142417750	RNF123
3:49740890	A/G		RNF123
3:49742546	C/T	rs777796703	RNF123
3:49743053	G/A	rs769646708	RNF123
3:49749975	C/T	rs548992767	RNF123
3:49828315	C/T	rs764180294	CDHR4
3:49847982	G/A	rs137907013	UBA7
3:49869488	C/T	rs143310707	TRAIP
3:50211316	G/A	rs985333083	SEMA3F
3:50220875	G/A	rs771028336	SEMA3F
3:50225523	G/A	rs761760550	SEMA3F
3:50369058	C/T	rs148222115	RASSF1
3:50380426	C/T	rs368114874	ZMYND10
3:51673540	C/T	rs777257233	RAD54L2
3:51812829	G/A	rs905439397	IQCF6
3:51929115	T/G	rs201302777	IQCF1
3:51969378	C/A	rs915998759	RRP9
3:51993654	C/T	rs1228914021	PCBP4
3:52022996	C/T	rs148346337	ABHD14A-ACY1,ACY1
3:52282409	G/A	rs187237629	PPM1M
3:52325811	A/G	rs747941622	GLYCTK
3:52397032	A/G	rs1303254929	DNAH1
3:52400878	G/A	rs199740667	DNAH1
3:52427382	C/T	rs759678423	DNAH1
3:52428993	G/A	rs202058333	DNAH1
3:52473728	C/T	rs201671398	SEMA3G
3:52546910	C/T	rs141381639	STAB1
3:52556184	C/G	rs143242234	STAB1
3:52559086	C/T	rs561845399	NT5DC2
3:52584541	G/C		PBRM1
3:52595971	G/A	rs371730711	PBRM1
3:52740108	C/A	rs779115533	SPCS1
3:52823682	T/A	rs1033280071	ITIH1
3:52867429	C/T	rs758008149	MUSTN1,STIMATE-MUSTN1
3:53769508	G/A	rs115066564	CACNA1D
3:53857665	C/T		CHDH
3:56026218	G/A	rs139250346	ERC2
3:56330114	T/C		ERC2
3:56763366	C/T	rs138533417	ARHGEF3
3:57232302	C/T	rs760503914	HESX1
3:57389202	A/G	rs559801349	DNAH12
3:57431001	G/A	rs776905251	DNAH12
3:57431804	A/G	rs138623026	DNAH12
3:57632163	C/A	rs747859024	DENND6A,PDE12
3:57847788	T/G	rs779312973	SLMAP
3:58088049	G/A		FLNB
3:58109300	A/G	rs921714937	FLNB
3:58196498	C/T	rs145888358	DNASE1L3

3:58516307	A/G	rs370811753	ACOX2
3:61989149	A/G	rs372067576	PTPRG
3:64635449	C/T	rs147650040	ADAMTS9
3:64672272	G/C		ADAMTS9
3:67426232	A/G	rs774922481	SUCLG2
3:69230836	G/A	rs780396995	FRMD4B
3:69237038	C/T	rs768015246	FRMD4B
3:72861879	G/A	rs746829352	SHQ1
3:73433538	G/A	rs970562680	PDZRN3
3:78667148	G/A	rs779648884	ROBO1
3:78680312	T/A		ROBO1
3:93624653	C/T		PROS1
3:93733392	T/C	rs774893160	ARL13B,STX19
3:93772073	G/A	rs142510905	ARL13B
3:96706285	C/T	rs202207730	EPHA6
3:96706825	G/C	rs565045900	EPHA6
3:97202879	C/T	rs774266264	EPHA6
3:97655739	G/A	rs370818948	CRYBG3
3:98237745	A/C	rs780302840	CLDND1
3:98251220	G/A	rs189514169	GPR15
3:100364930	T/A	rs751020877	ADGRG7
3:100470398	T/G		ABI3BP
3:101540494	G/A	rs374457110	NXPE3
3:108110755	A/G		MYH15
3:108117545	C/T	rs372228730	MYH15
3:108220603	C/T	rs200749942	MYH15
3:108278711	T/C	rs376519054	CIP2A
3:108409736	A/G	rs751865193	DZIP3
3:108635060	G/A	rs11917716	GUCA1C
3:110853057	G/A	rs143220633	NECTIN3
3:111427232	A/G	rs774448388	PLCXD2
3:111603355	A/T	rs143220265	PHLDB2
3:111672822	C/T	rs79994265	PHLDB2
3:112727049	G/A	rs149448097	NEPRO
3:112998137	G/A	rs766270235	BOC
3:113025094	T/G	rs897389534	CFAP44
3:113218286	C/G	rs759681412	SPICE1
3:113334964	C/T	rs140834549	SIDT1
3:113784292	C/T	rs370355409	QTRT2
3:118943073	T/C		B4GALT4
3:119176909	C/T	rs746922013	TMEM39A
3:120371474	G/T	rs559502955	HGD
3:121100164	G/A	rs200186528	STXBP5L
3:121168167	T/C	rs150364457	POLQ
3:122274792	T/C	rs139882806	PARP9
3:122288809	C/T	rs145773907	DTX3L
3:122354889	T/C	rs752861790	PARP15
3:123419328	C/T	rs761654890	MYLK
3:124053278	A/C		KALRN
3:124180771	G/T		KALRN

3:124385342	A/G	rs139843692	KALRN
3:124431825	C/T	rs371482945	KALRN
3:124689539	G/A	rs546708760	HEG1
3:124716640	G/A	rs200670096	HEG1
3:125828823	T/C	rs201903896	ALDH1L1
3:125843294	C/T	rs141020864	ALDH1L1
3:126142483	C/T	rs765644982	CFAP100
3:126155240	T/C		CFAP100
3:126207086	C/T	rs758929808	UROC1
3:126218211	G/A	rs9871671	UROC1
3:126226811	A/G		UROC1
3:126708364	C/T	rs778885164	PLXNA1
3:127325595	G/C	rs749963918	MCM2
3:127336826	C/G	rs1181663475	MCM2
3:127395143	G/A	rs748869280	ABTB1
3:127983506	C/T	rs142548867	EEFSEC
3:128181772	C/A	rs141309633	DNAJB8
3:128616543	A/G	rs770234633	ACAD9
3:128618236	T/C	rs1388586677	ACAD9
3:128628253	C/T	rs150283105	ACAD9
3:128984527	C/T	rs768991875	COPG1
3:129200437	G/A	rs138223055	IFT122
3:129223244	G/A	rs561698295	IFT122
3:129695893	C/T	rs35004321	TRH
3:130098733	G/C		COL6A5
3:130107816	G/A	rs199835289	COL6A5
3:130110291	G/T	rs144767448	COL6A5
3:130114278	C/T	rs543746446	COL6A5
3:130150452	G/A	rs569432794	COL6A5
3:130285645	A/C	rs11921769	COL6A6
3:130734988	C/T	rs146481344	ASTE1,ATP2C1
3:132224222	A/C	rs202044084	DNAJC13
3:133692572	C/T	rs772154759	SLCO2A1
3:133698375	G/A	rs200244112	SLCO2A1
3:134670252	A/G	rs201772314	EPHB1
3:134920500	A/G	rs753024740	EPHB1
3:134968200	C/T	rs143309901	EPHB1
3:134968219	C/T	rs759415643	EPHB1
3:136012626	C/T	rs374722096	PCCB
3:137822466	C/G		DZIP1L
3:139257634	G/A	rs150001856	RBP1
3:140275475	C/T	rs757739003	CLSTN2
3:140397391	G/A	rs146449412	TRIM42
3:141006223	G/A	rs754179505	PXYLP1
3:141011898	A/G	rs142918405	PXYLP1
3:141499322	A/T	rs1291288066	GRK7
3:142405208	C/T	rs1393424858	PLS1
3:142840409	G/C		CHST2
3:145828135	C/G	rs147440183	PLOD2
3:146246430	A/G		PLSCR1

3:148459747	T/C	rs764229950	AGTR1
3:148597629	G/A	rs192487183	CPA3
3:148741914	C/T	rs201672568	GYG1
3:149563829	G/C		RNF13
3:149683024	A/G	rs537764717	PFN2
3:150387095	T/C	rs149475266	ERICH6
3:151156090	C/T	rs201909176	IGSF10
3:153912438	C/G	rs146072099	ARHGEF26
3:153958213	A/C	rs780783816	ARHGEF26
3:155200309	A/G		PLCH1
3:155200349	G/T	rs772397831	PLCH1
3:158317831	A/T	rs1560111229	MLF1
3:158428529	G/C	rs1464085333	RARRES1
3:158428589	T/A	rs11919919	RARRES1
3:158520101	T/C		MFSD1
3:160132258	A/T		SMC4
3:160803840	G/T		B3GALNT1
3:164754237	C/T	rs542969966	SI
3:164764736	A/G	rs765433197	SI
3:164905876	C/T		SLITRK3
3:165548394	C/T	rs201820739	BCHE
3:167045816	G/A		ZBBX
3:167083741	G/A	rs200250021	ZBBX
3:169514038	C/T		LRRC34
3:169540665	C/G	rs768956499	LRRIQ4
3:170786653	A/G	rs373014554	TNIK
3:170843760	G/A	rs762447384	TNIK
3:172064153	G/A	rs139746767	FNDC3B
3:179069793	G/A	rs1435820962	MFN1
3:179103439	T/A	rs150378003	MFN1
3:179462903	G/A	rs765271688	USP13
3:179529649	A/C	rs141827659	PEX5L
3:180320723	C/T	rs143759891	TTC14
3:180702475	T/C		DNAJC19
3:182810216	T/G	rs148616219	MCCC1
3:183014889	G/C	rs754136583	MCF2L2
3:183823141	G/A	rs115516419	HTR3E
3:183955083	G/A	rs996922341	VWA5B2
3:184009183	G/A	rs752796877	ECE2
3:184035520	G/A		EIF4G1
3:184049571	C/T	rs377408315	EIF4G1
3:184070569	G/A	rs114702742	CLCN2
3:184297306	G/A	rs112385651	EPHB3
3:185198193	C/T	rs544421900	MAP3K13
3:185990109	G/A	rs199698541	DGKG
3:186299190	G/T		DNAJB11
3:186839079	G/A	rs149473749	RPL39L
3:187003786	C/T	rs77189011	MASP1
3:187416740	T/C	rs143072121	RTP2
3:187446313	G/A	rs137878288	BCL6

3:189705368	T/A		P3H2
3:189838136	C/A	rs200731219	P3H2
3:193132500	C/T	rs777360198	ATP13A4
3:193332528	T/A	rs760770105	OPA1
3:194063356	A/T		CPN2
3:194150490	C/G		ATP13A3
3:194175095	C/G		ATP13A3
3:194379780	C/T	rs147988904	LSG1
3:195000131	G/A	rs775919980	ACAP2
3:195477897	A/C	rs201198170	MUC4
3:195490488	A/C		MUC4
3:195594530	C/T	rs202157460	TNK2
3:195794525	G/A	rs368049940	TFRC
3:196234847	C/T	rs116717850	SMCO1
3:196288054	T/C	rs527371926	WDR53
3:196735690	C/G	rs768150503	MELTF
3:196736638	C/T	rs199669757	MELTF
3:196831780	G/A	rs201145407	DLG1
3:197241242	T/G	rs770637426	BDH1
3:197483409	G/C	rs754044260	FYTTD1
3:197581302	A/G	rs1484280668	LRCH3
4:436856	T/C	rs200508844	ZNF721
4:493194	C/T	rs782798787	PIGG
4:517306	C/T	rs755701436	PIGG
4:524499	C/T	rs368126393	PIGG
4:619719	G/A	rs750237172	PDE6B
4:677471	C/T	rs139230183	SLC49A3
4:983358	C/G		IDUA,SLC26A1
4:1164948	G/T		SPON2
4:1343343	C/T	rs574965437	UVSSA
4:1730352	A/T	rs746878628	TACC3
4:3024158	A/C	rs139428941	GRK4
4:3208534	C/T	rs769547244	HTT
4:3257507	A/C	rs199592772	MSANTD1
4:3418737	C/T	rs140454822	RGS12
4:3422402	G/A	rs759683485	RGS12
4:3430406	A/T	rs142314294	RGS12
4:3478212	C/T	rs368891521	DOK7
4:3487310	A/G	rs139133047	DOK7
4:3494771	C/T	rs757019137	DOK7
4:3494938	C/T	rs199606134	DOK7
4:4322411	A/G	rs146575965	ZBTB49
4:5830268	G/A	rs370759908	CRMP1
4:5868474	G/A		CRMP1
4:6303548	C/T	rs201623184	WFS1
4:6303551	G/C		WFS1
4:6863202	C/T	rs751571296	KIAA0232
4:7728561	G/A	rs200680489	SORCS2
4:8062712	C/T	rs549704297	ABLIM2
4:8229252	T/A	rs759249056	SH3TC1

4:8230036	C/G		SH3TC1
4:8418166	G/A	rs556118466	ACOX3
4:8453716	G/T	rs746235503	TRMT44
4:8605742	C/A	rs149934510	CPZ
4:10079513	G/A	rs186889066	WDR1
4:13475952	T/C	rs762836870	RAB28
4:13602102	C/T	rs766131519	BOD1L1
4:13602867	C/T	rs779223268	BOD1L1
4:15601195	C/T	rs756324214	CC2D2A
4:17585181	C/T	rs775885725	LAP3
4:17836144	G/A	rs775240842	NCAPG
4:22389926	G/A	rs371121360	ADGRA3
4:23833353	C/G	rs370930722	PPARGC1A
4:24801667	G/A	rs373731075	SOD3
4:25347214	T/G		ZCCHC4
4:36162083	T/C	rs1157680048	ARAP2
4:37836294	G/A	rs145365671	PGM2
4:38046045	G/T	rs201258145	TBC1D1
4:38776772	A/T	rs1266496565	TLR10
4:38798720	T/G	rs138016555	TLR1
4:39218798	A/G	rs778750936	WDR19
4:39245928	C/T	rs1211391439	WDR19
4:39505589	C/G		UGDH
4:46053514	G/T	rs752448120	GABRG1
4:47644063	G/T	rs748677860	CORIN
4:47695011	C/A	rs751480678	CORIN
4:48165752	G/A	rs191626703	TEC
4:48385675	C/T	rs375370862	SLAIN2
4:48503699	G/C	rs1456691230	FRYL
4:48552695	C/T		FRYL
4:48583583	C/T	rs373033214	FRYL
4:54327623	G/A	rs757870438	LNX1
4:54344781	G/C	rs373637202	LNX1
4:54966564	C/T	rs1451179519	GSX2
4:56345050	C/A		CLOCK
4:56762973	G/T		EXOC1
4:56765976	G/A		EXOC1
4:57342848	C/T	rs375436807	SRP72
4:57798313	G/A		REST
4:66217267	C/G	rs199801676	EPHA5
4:68447185	C/T	rs199787258	STAP1
4:69095170	G/A	rs201912154	TMPRSS11B
4:69962746	T/A		UGT2B7
4:69962882	T/A	rs142000539	UGT2B7
4:70152601	G/T	rs762494511	UGT2B28
4:70160376	C/A	rs756229589	UGT2B28
4:70361099	C/T	rs200640027	UGT2B4
4:70455277	A/G	rs1412453515	UGT2A1,UGT2A2
4:70596250	C/T		SULT1B1
4:72316997	A/T	rs752935590	SLC4A4

4:73013168	A/T	rs150345262	NPFFR2
4:73013288	G/A	rs749148721	NPFFR2
4:74275120	A/T	rs149432908	ALB
4:74283848	C/T	rs776185501	ALB
4:74447572	G/A	rs200606029	RASSF6
4:74607695	G/C		CXCL8
4:75147198	G/T		MTHFD2L
4:76434476	G/A	rs777695754	RCHY1
4:76797634	G/A	rs138339033	PPEF2
4:77662352	G/A	rs757427504	SHROOM3
4:77677978	A/G		SHROOM3
4:79522685	C/T	rs5949	ANXA3
4:80327982	C/T	rs151024112	GK2
4:80328600	G/A	rs201151701	GK2
4:81967620	C/A	rs1270973941	BMP3
4:81974568	C/A	rs758455386	BMP3
4:83372371	C/T	rs1487304047	ENOPH1
4:83803070	T/C	rs139031667	SEC31A
4:84406162	G/A	rs756786001	ABRAXAS1
4:86916213	G/A	rs1274796676	ARHGAP24
4:87655528	C/T	rs766112626	PTPN13
4:87672251	A/G	rs200688335	PTPN13
4:87672268	T/C		PTPN13
4:87692496	G/A	rs200197835	PTPN13
4:88902627	A/T	rs200088575	SPP1
4:88996664	G/A	rs140848008	PKD2
4:89052998	G/T	rs199473672	ABCG2
4:89671007	G/T		FAM13A
4:90169139	G/A	rs551229625	GPRIN3
4:90856092	G/A	rs534739687	MMRN1
4:90874460	T/C	rs201798101	MMRN1
4:93511327	G/A	rs754954663	GRID2
4:94128584	C/A		GRID2
4:94436444	G/A	rs747630568	GRID2
4:98893489	C/T	rs111720937	STPG2
4:101368728	G/T	rs139103613	EMCN
4:101947057	C/G		PPP3CA
4:103531777	A/G	rs368017285	NFKB1
4:108866306	T/C	rs139692261	CYP2U1
4:109010381	A/T		LEF1
4:110615797	C/T	rs775859085	CASP6
4:110667561	T/G	rs61733901	CFI
4:110687722	A/C		CFI
4:111441371	G/C	rs143234247	ENPEP
4:111553561	G/T		PITX2
4:113303592	G/A	rs751762758	ALPK1
4:113347689	C/T	rs761316245	ALPK1
4:113362114	G/T		ALPK1
4:114295962	T/C		ANK2
4:114378512	C/A		CAMK2D

4:115766993	A/T		NDST4
4:118006190	G/T	rs370224857	TRAM1L1
4:119610559	C/T	rs748869746	METTL14
4:119952262	C/T	rs756919263	SYNPO2
4:119952410	C/T	rs777838583	SYNPO2
4:122301571	T/A		QRFPR
4:122604592	A/C	rs747497278	ANXA5
4:122728748	T/G	rs200764818	EXOSC9
4:123663414	T/G		BBS12
4:123848902	G/A	rs758521022	SPATA5
4:123856880	C/T	rs201751275	SPATA5
4:123978383	C/T		SPATA5
4:128627733	A/G	rs140878220	INTU
4:128690018	C/T	rs778830237	SLC25A31
4:128807318	A/G	rs377756201	PLK4
4:128811378	C/T		PLK4
4:128841790	C/T	rs1398979129	MFSD8
4:129100595	A/G	rs149195038	LARP1B
4:129773373	G/C		JADE1
4:130032906	C/T	rs370206168	C4orf33
4:140278653	A/G		NAA15
4:141483476	G/A	rs148598275	UCP1
4:141543579	G/A	rs777765891	TBC1D9
4:146064561	G/A		OTUD4
4:146071731	C/A	rs148857745	OTUD4
4:151199155	G/A	rs140288109	LRBA
4:151771966	C/T	rs144754728	LRBA
4:151773141	T/A	rs149689496	LRBA
4:152096098	G/C	rs371516037	SH3D19
4:152550945	G/A	rs753203625	FAM160A1
4:152559873	A/T	rs185879278	FAM160A1
4:153802203	A/G	rs146568129	ARFIP1
4:154478199	A/G		TMEM131L
4:154624364	T/C	rs1455850790	TLR2
4:154631595	G/A	rs368796891	RNF175
4:155157646	C/T	rs115331928	DCHS2
4:155158010	A/T	rs143512002	DCHS2
4:155225826	A/G	rs143254462	DCHS2
4:155411607	A/G	rs755226601	DCHS2
4:155507493	C/G	rs772887890	FGA
4:155533353	C/T	rs202132393	FGG
4:156864331	T/C	rs764634611	CTSO
4:159091722	T/C	rs199881611	GASK1B
4:159780781	T/C	rs770696633	FNIP2
4:162841615	G/T	rs769789513	FSTL5
4:164393850	G/A		TKTL2
4:169630202	G/A	rs145026144	PALLD
4:169928890	T/A	rs138581722	CBR4
4:177049909	C/T	rs146789582	WDR17
4:177608606	C/A	rs745847895	VEGFC

4:178355591	C/T		AGA
4:183522104	C/G	rs1465147111	TENM3
4:183550007	C/T	rs1457472687	TENM3
4:183664443	C/T	rs768401343	TENM3
4:183714300	C/G		TENM3
4:183721320	G/A		TENM3
4:183815690	C/T	rs777881225	DCTD
4:186067041	G/A	rs764225621	SLC25A4
4:186353232	T/C	rs149697117	C4orf47
4:186425659	G/A	rs200921332	PDLIM3
4:187004789	C/T	rs1248175611	TLR3
4:187527348	G/A	rs184300563	FAT1
4:187628737	T/G	rs774753400	FAT1
5:191910	G/T	rs762440075	LRRC14B
5:226050	A/G		SDHA
5:1087065	T/C	rs201694282	SLC12A7
5:1414926	C/T	rs375720650	SLC6A3
5:5190148	G/A	rs368918485	ADAMTS16
5:5237088	A/G		ADAMTS16
5:5239829	C/T	rs768212348	ADAMTS16
5:6604756	C/T	rs763793795	NSUN2
5:7851073	G/A	rs777647731	C5orf49
5:9197308	G/A	rs764453795	SEMA5A
5:9629567	G/T	rs185412063	TAS2R1
5:10239222	C/T	rs754421049	ATPSCKMT
5:10430101	G/A	rs763034228	MARCHF6
5:10681232	G/A	rs1267746944	DAP
5:13736021	G/A	rs146750552	DNAH5
5:13771059	A/G	rs561495955	DNAH5
5:16666902	C/A		MYO10
5:16694609	C/T		MYO10
5:31551454	A/G		C5orf22
5:32090378	A/G	rs150841463	PDZD2
5:33649069	C/T	rs201968522	ADAMTS12
5:34845659	G/C	rs1399369843	TTC23L
5:34936303	G/C	rs139629564	DNAJC21
5:34945004	G/A	rs142389949	DNAJC21
5:35035343	C/T	rs201803834	AGXT2
5:35692692	T/C		SPEF2
5:35876565	T/C	rs141919625	IL7R
5:35910608	G/A	rs769642485	CAPSL
5:36961643	C/G		NIPBL
5:37183490	G/T	rs147588579	CPLANE1
5:37226584	G/C		CPLANE1
5:37243184	T/C	rs144969169	CPLANE1
5:38427158	G/A	rs967886707	EGFLAM
5:38463035	G/A	rs374237322	EGFLAM
5:39122475	C/T	rs932786216	FYB1
5:41000344	T/C	rs755024109	MROH2B
5:42711350	G/C	rs200851410	GHR

5:43280331	T/A	rs372224603	NIM1K
5:43292628	T/A	rs368814700	HMGCS1
5:43493673	T/C	rs1388371232	C5orf34
5:43494684	G/A	rs200027263	C5orf34
5:43503779	C/A	rs772253133	C5orf34
5:43508686	C/A		C5orf34
5:44305146	C/T	rs1158466327	FGF10
5:50685542	A/T	rs200209474	ISL1
5:52368473	G/A	rs118190231	ITGA2
5:52403014	G/A	rs146074751	MOCS2
5:54941700	G/C		SLC38A9
5:55206398	G/A	rs113369650	IL31RA
5:55259997	A/G	rs773026173	IL6ST
5:55412524	C/T		ANKRD55
5:55422864	C/T	rs1157754314	ANKRD55
5:56152492	G/A	rs1454725137	MAP3K1
5:56778422	C/T	rs765705222	ACTBL2
5:60839821	C/T	rs780375063	ZSWIM6
5:61783670	A/G		IPO11
5:63257152	A/T		HTR1A
5:64511194	G/A	rs149141130	ADAMTS6
5:64521948	C/G	rs373118005	ADAMTS6
5:65364726	A/T	rs547628959	ERBIN
5:65370866	T/G		ERBIN
5:66055555	C/T	rs749776188	MAST4
5:66410006	A/T		MAST4
5:68423916	T/C	rs373005717	SLC30A5
5:68603851	A/C	rs147567034	CCDC125
5:68661527	G/A	rs769223849	AK6,TAF9
5:68805495	A/G	rs201347125	OCLN
5:70809074	G/C	rs201608641	BDP1
5:70888804	G/A	rs752593298	MCCC2
5:72419319	C/T	rs779047093	TMEM171
5:74014629	C/T	rs28942073	HEXB
5:74324988	G/C	rs750910859	GCNT4
5:74646765	A/C	rs191835914	HMGCR
5:75948620	C/T	rs35366349	IQGAP2
5:76703211	A/G	rs765604396	PDE8B
5:78379069	C/T	rs60797063	BHMT2
5:78384363	C/A	rs148183648	BHMT2
5:79366195	G/A	rs138775049	THBS4
5:79817889	C/G	rs775123191	FAM151B
5:82816056	C/A	rs148104757	VCAN
5:86659294	A/G	rs145752649	RASA1
5:89938739	T/C		ADGRV1
5:90671337	A/G		ARRDC3
5:93159963	G/A		FAM172A
5:94830478	G/A	rs557540994	TTC37
5:94860209	G/C	rs777087809	TTC37
5:94994373	A/G		SPATA9

5:96232521	G/A	rs201986447	ERAP2
5:96503204	A/G	rs142914308	RIOK2
5:98128948	G/A	rs61742751	RGMB
5:101755653	G/A	rs200993517	SLCO6A1
5:101774321	C/T		SLCO6A1
5:109864536	C/T	rs6894728	TMEM232
5:110074968	C/A	rs147648476	SLC25A46
5:110440041	A/G	rs118204022	WDR36
5:110835762	C/T	rs146729717	STARD4
5:111500792	G/C	rs200449454	EPB41L4A
5:111616010	T/C	rs199799127	EPB41L4A
5:112177764	C/G	rs587779804	APC
5:112384867	C/T	rs925915589	MCC
5:114606941	G/T		CCDC112
5:118183823	C/T	rs148574499	DTWD2
5:118451943	G/A	rs368455602	DMXL1
5:118485651	A/G	rs775710410	DMXL1
5:118872238	C/T		HSD17B4
5:121758755	G/C	rs773615333	SNCAIP
5:125816383	C/A	rs762549860	GRAMD2B
5:126145905	C/T	rs142016804	LMNB1
5:126158551	G/A	rs750727860	LMNB1
5:126161759	G/A	rs1462823275	LMNB1
5:126206420	G/A		MARCHF3
5:126791225	C/T	rs1394979668	MEGF10
5:127674693	G/A	rs772041342	FBN2
5:131302170	G/A	rs115302912	ACSL6
5:131705698	G/A	rs139203363	SLC22A5
5:131925354	A/G	rs145428112	RAD50
5:132412451	A/G	rs771713880	HSPA4
5:133292647	T/G	rs752638440	C5orf15
5:135207303	A/G	rs747797136	SLC25A48
5:137271589	A/C	rs373884287	PKD2L2
5:137272050	A/G	rs1561712480	PKD2L2
5:137275968	C/G	rs201094212	FAM13B,PKD2L2
5:137354051	C/T	rs148088678	FAM13B
5:137451411	G/A	rs1448308637	NME5
5:137451438	A/G		NME5
5:137502353	G/T		BRD8
5:137893569	C/T	rs768967239	HSPA9
5:138657632	G/C		MATR3
5:138860779	G/T	rs142609349	STING1
5:139060136	G/T		CXXC5
5:139060809	C/T	rs747221615	CXXC5
5:139216541	G/A	rs138380367	PSD2
5:139744061	G/A	rs547845941	SLC4A9
5:139818160	G/A	rs145577675	ANKHD1,ANKHD1-EIF4EBP3
5:139930379	C/A	rs148927164	SRA1
5:140054320	C/T	rs199615869	HARS1
5:140167995	T/G	rs782121889	PCDHA1

5:140168112	G/T	rs377323471	PCDHA1
5:140202322	C/T	rs140652619	PCDHA1,PCDHA2,PCDHA3,PCDHA4,PCDHA5
5:140214280	C/G		PCDHA1,PCDHA2,PCDHA3,PCDHA4,PCDHA5,PCDHA6,PCDHA7
5:140215836	G/T	rs1183967538	PCDHA1,PCDHA2,PCDHA3,PCDHA4,PCDHA5,PCDHA6,PCDHA7
5:140222264	C/T	rs574989982	PCDHA1,PCDHA2,PCDHA3,PCDHA4,PCDHA5,PCDHA6,PCDHA7,PCDH
5:140256112	T/C		PCDHA1,PCDHA2,PCDHA3,PCDHA4,PCDHA5,PCDHA6,PCDHA7,PCDH
5:140256720	G/T		PCDHA1,PCDHA2,PCDHA3,PCDHA4,PCDHA5,PCDHA6,PCDHA7,PCDH
5:140264020	T/G		PCDHA1,PCDHA2,PCDHA3,PCDHA4,PCDHA5,PCDHA6,PCDHA7,PCDH
5:140530418	G/A	rs146982646	PCDHB6
5:140553017	G/T	rs149003370	PCDHB7
5:140581011	A/G	rs782042453	PCDHB11
5:140581283	G/A	rs781914954	PCDHB11
5:140589294	G/T	rs200437596	PCDHB12
5:140603601	A/G	rs200378467	PCDHB14
5:140683228	G/A	rs1356032865	SLC25A2
5:140710970	C/T		PCDHGA1
5:140720211	A/G		PCDHGA1,PCDHGA2
5:140730489	G/A	rs199977912	PCDHGA1,PCDHGA2,PCDHGA3,PCDHGB1
5:140731461	G/C	rs146243220	PCDHGA1,PCDHGA2,PCDHGA3,PCDHGB1
5:140751096	A/G	rs200031435	PCDHGA1,PCDHGA2,PCDHGA3,PCDHGA4,PCDHGA5,PCDHGB1,PCDH
5:140767561	T/C	rs1376366069	PCDHGA1,PCDHGA2,PCDHGA3,PCDHGA4,PCDHGA5,PCDHGA6,PCDH
5:140778086	C/T		PCDHGA1,PCDHGA2,PCDHGA3,PCDHGA4,PCDHGA5,PCDHGA6,PCDH
5:140794288	G/A	rs373261988	PCDHGA1,PCDHGA2,PCDHGA3,PCDHGA4,PCDHGA5,PCDHGA6,PCDH
5:140794750	G/A	rs201831693	PCDHGA1,PCDHGA2,PCDHGA3,PCDHGA4,PCDHGA5,PCDHGA6,PCDH
5:140801516	C/T	rs749189262	PCDHGA1,PCDHGA2,PCDHGA3,PCDHGA4,PCDHGA5,PCDHGA6,PCDH
5:140802002	A/G	rs199795822	PCDHGA1,PCDHGA2,PCDHGA3,PCDHGA4,PCDHGA5,PCDHGA6,PCDH
5:140802386	T/C	rs369004166	PCDHGA1,PCDHGA2,PCDHGA3,PCDHGA4,PCDHGA5,PCDHGA6,PCDH
5:140856347	C/T	rs201463036	PCDHGA1,PCDHGA2,PCDHGA3,PCDHGA4,PCDHGA5,PCDHGA6,PCDH
5:140865930	T/A		PCDHGA1,PCDHGA2,PCDHGA3,PCDHGA4,PCDHGA5,PCDHGA6,PCDH
5:140869229	G/A	rs2233601	PCDHGA1,PCDHGA2,PCDHGA3,PCDHGA4,PCDHGA5,PCDHGA6,PCDH
5:140957903	C/T	rs201545942	DIAPH1
5:141307749	A/G	rs11948907	DELE1
5:141336115	G/T	rs140142979	PCDH12
5:141354386	C/T	rs200103265	RNF14
5:145859441	T/A		TCERG1
5:147593566	T/C	rs1390298258	SPINK6
5:148207053	C/G	rs3729943	ADRB2
5:148627394	G/A	rs373620710	ABLIM3
5:148679125	G/A	rs1185148835	AFAP1L1
5:148702241	C/T	rs114805975	AFAP1L1
5:148748096	C/A	rs376501121	PCYOX1L
5:149247713	A/G	rs144970068	PDE6A
5:149279025	G/C		PDE6A
5:149310665	C/T	rs148938083	PDE6A
5:149431550	C/T	rs1219532512	HMGXB3
5:149771158	G/A		TCOF1
5:150090851	C/T	rs778708391	DCTN4
5:150407037	T/G	rs201249129	GPX3
5:150672988	G/T	rs150561602	SLC36A3
5:150885451	C/T	rs144888662	FAT2

5:150911174	C/A	rs1425887403	FAT2
5:150922468	G/T	rs749135258	FAT2
5:150924696	T/G	rs751382874	FAT2
5:150943074	T/C	rs199601916	FAT2
5:150946865	C/T	rs778707746	FAT2
5:151176883	A/C	rs376309064	G3BP1
5:151202245	G/T	rs146481873	GLRA1
5:151784001	T/C	rs778545109	NMUR2
5:154275665	G/T		GEMIN5
5:154397093	G/A	rs575430308	KIF4B
5:156592768	G/C	rs143562132	FAM71B
5:156770375	G/A	rs34741916	CYFIP2,FNDC9
5:157065428	G/C	rs138390114	SOX30
5:157078188	G/A	rs375931805	SOX30
5:158139320	C/T	rs756448619	EBF1
5:159776735	C/T	rs1181577074	C1QTNF2
5:167841429	C/T	rs575655082	WWC1
5:167937602	C/T	rs762675175	RARS1
5:169291320	C/T	rs199958998	DOCK2,INSYN2B
5:169714980	C/T	rs747190286	LCP2
5:169812358	A/G	rs780940473	KCNIP1,KCNMB1
5:170319453	C/G	rs200291830	RANBP17
5:170336681	C/G	rs139178386	RANBP17
5:170336717	G/A	rs144195437	RANBP17
5:170345819	G/C	rs145822469	RANBP17
5:171509391	C/T	rs143251398	STK10
5:171661282	G/A	rs765867493	UBTD2
5:171789778	C/T	rs374467295	SH3PXD2B
5:172341812	C/T	rs1398482784	ERGIC1
5:172353527	G/A	rs761420337	ERGIC1
5:172573948	G/T	rs144179275	BNIP1
5:173372017	C/T	rs376710749	CPEB4
5:173534363	A/G	rs1457723218	NSG2
5:175388279	G/A	rs547312788	THOC3
5:176002580	C/T	rs144549731	CDHR2
5:176005428	T/G		CDHR2
5:176072868	C/T	rs182336246	EIF4E1B
5:176079750	G/A	rs777541888	TSPAN17
5:176317824	C/G	rs201738990	HK3
5:176765507	G/A	rs372222840	LMAN2
5:176863157	G/A	rs1458423902	GRK6
5:177574635	C/T	rs143335953	RMND5B
5:177580532	C/T	rs79031130	NHP2
5:178421480	C/T	rs1172096502	GRM6
5:178503473	G/A	rs138654012	ZNF354C
5:178700052	C/T	rs763905470	ADAMTS2
5:179192736	C/A	rs778196575	MAML1
5:179193441	C/A	rs200515773	MAML1
5:179318448	G/C	rs773179113	TBC1D9B
5:179318539	C/T	rs201568253	TBC1D9B

5:179743819	C/T	rs193143625	GFPT2
5:180039583	C/T	rs75614493	FLT4
5:180052980	C/T	rs866937901	FLT4
5:180432379	T/A	rs369608700	BTNL3
5:180432694	G/A	rs201822700	BTNL3
5:180582117	C/T	rs749998402	OR2V2
6:2836098	G/C	rs200375056	SERPINB1
6:2948680	G/A	rs376116821	SERPINB6
6:3015843	A/T	rs761367357	NQO2
6:3140676	G/A	rs201590620	BPHL
6:6266932	G/A	rs767046666	F13A1
6:7231117	G/A	rs145478005	RREB1
6:7574952	A/G	rs778350289	DSP
6:7576615	C/T	rs749051278	DSP
6:7576619	G/A	rs142494121	DSP
6:7579928	T/C		DSP
6:7727471	C/T	rs199518216	BMP6
6:7727588	C/G	rs759005904	BMP6
6:10876742	C/T	rs1447940760	GCM2
6:11134642	A/G	rs752733232	SMIM13
6:12122510	G/A	rs2228218	HIVEP1
6:12123018	C/G	rs746771188	HIVEP1
6:12123952	G/C	rs369754956	HIVEP1
6:15496616	G/A	rs146276296	JARID2
6:16130851	G/A	rs149696224	MYLIP
6:16274716	G/A	rs1161919049	GMPR
6:17800230	C/T	rs201527270	KIF13A
6:17987661	C/G	rs367718683	KIF13A
6:18222162	A/C		KDM1B
6:24475437	A/C		GPLD1
6:24503570	G/A	rs369366567	ALDH5A1
6:25653678	G/T	rs146743002	SCGN
6:25811886	C/T	rs200727863	SLC17A1
6:26017836	G/A	rs117409123	H1-1
6:26056361	G/A	rs147569209	H1-2
6:26598784	C/T	rs782187306	ABT1
6:28219404	C/T	rs199868712	ZKSCAN4
6:33543634	C/T	rs775583296	BAK1
6:33638948	A/C	rs202195536	ITPR3
6:33638949	G/T	rs199987334	ITPR3
6:33644564	C/T	rs149129212	ITPR3
6:33647740	G/A	rs368822884	ITPR3
6:35444083	T/A	rs200154661	TEAD3
6:35473555	G/C		TULP1
6:35565058	C/T	rs142925622	FKBP5
6:35803154	G/C		SRPK1
6:36196679	A/G	rs775759948	BRPF3
6:36993632	G/A	rs137992835	FGD2
6:38224215	A/G	rs376734377	BTBD9
6:38560432	A/C		BTBD9

6:38775450	G/A	rs201654193	DNAH8
6:38828403	T/G	rs1301938853	DNAH8
6:38851728	T/C		DNAH8
6:38976675	C/T	rs189023122	DNAH8
6:39158772	G/A	rs770248541	KCNK5
6:39311571	G/A	rs180943940	KIF6
6:39838191	C/T	rs759121825	DAAM2
6:39847075	C/G	rs770538092	DAAM2
6:39869129	G/A	rs751217536	DAAM2
6:39874266	G/A	rs761344256	MOCS1
6:40359760	C/A	rs770037133	LRFN2
6:41565642	A/G		FOXP4
6:41739579	T/C	rs143921336	FRS3
6:41753121	G/A	rs762141132	PRICKLE4
6:42176643	A/C	rs201208600	MRPS10
6:42237177	G/A	rs201817030	TRERF1
6:42936082	G/A		PEX6
6:42977097	A/G		PPP2R5D
6:42993029	G/A		RRP36
6:43012621	A/C	rs61752334	CUL7
6:43019950	C/T	rs369167170	CUL7
6:43019980	G/A	rs754739961	CUL7
6:43096865	G/A	rs1194029113	PTK7
6:43168517	G/A		CUL9
6:43323496	G/A	rs151197319	ZNF318
6:43324886	T/C		ZNF318
6:43336745	C/T	rs1297089974	ZNF318
6:43405713	G/C	rs200874313	ABCC10
6:43412532	G/A	rs142010618	ABCC10
6:43418543	G/A	rs139041633	DLK2
6:43474986	G/A	rs761034084	LRRC73
6:43485053	A/G	rs144054843	POLR1C
6:43485104	C/G	rs186395114	POLR1C
6:43592317	T/G		GTPBP2
6:43643296	A/T	rs148462467	MRPS18A
6:44141001	G/A	rs202150569	CAPN11
6:44273504	C/G	rs759161776	AARS2
6:44988294	G/A	rs373980353	SUPT3H
6:46658846	A/G	rs373230169	TDRD6
6:46979810	G/A	rs749959769	ADGRF1
6:47650064	G/A	rs200911695	ADGRF2
6:49425590	A/C	rs1561959114	MMUT
6:49479741	T/C	rs560399915	GLYATL3
6:49754131	A/G		PGK2
6:51609303	A/G	rs149798764	PKHD1
6:51612752	G/A	rs145141656	PKHD1
6:52147511	G/A	rs748245826	MCM3
6:52147607	C/G	rs754087825	MCM3
6:53516863	A/G		KLHL31
6:54735339	G/A	rs769300499	FAM83B

6:54804992	G/A	rs375673525	FAM83B
6:54806564	G/A	rs200887888	FAM83B
6:55360360	C/T	rs377232502	HMGCLL1
6:55378847	C/T	rs963740838	HMGCLL1
6:55922559	G/A	rs753645403	COL21A1
6:56035586	T/A	rs925947579	COL21A1
6:56357819	C/A	rs750283690	DST
6:56425209	C/T		DST
6:56499281	T/A		DST
6:57013143	C/T	rs575508843	ZNF451
6:64421553	G/A	rs1301247493	PHF3
6:70034878	C/T		ADGRB3
6:70944498	G/A	rs763616322	COL9A1
6:70980028	G/T	rs144009736	COL9A1
6:71185243	T/A	rs769480483	FAM135A
6:71234195	A/C	rs534314794	FAM135A
6:71665861	C/A	rs951776976	B3GAT2
6:73904782	T/G		KCNQ5
6:74201964	G/T		MTO1
6:74490991	C/T	rs1263132820	CD109
6:74517959	G/A	rs770499492	CD109
6:75843623	C/T	rs770194625	COL12A1
6:75898153	T/A	rs116980451	COL12A1
6:76023263	T/C	rs758261361	FILIP1
6:76344476	G/A	rs531966562	SENP6
6:76412455	A/G	rs201057204	SENP6
6:76617323	G/A	rs1562313932	MYO6
6:76712698	G/T	rs138431617	IMPG1
6:79607589	G/T	rs200615035	IRAK1BP1
6:79671522	C/A	rs147526156	PHIP
6:82924283	T/C		IBTK
6:83832662	T/C	rs761268279	DOP1A
6:83848215	T/C	rs139555117	DOP1A
6:83855292	G/A	rs367662678	DOP1A
6:84269884	A/C	rs752107730	SNAP91
6:84574041	A/G		CYB5R4
6:84879058	G/A	rs138206646	CEP162
6:84884465	T/C	rs756319986	CEP162
6:86160088	C/G		NT5E
6:86281905	G/C		SNX14
6:87971314	A/G	rs573895028	ZNF292
6:87994455	C/T	rs745724761	GJB7
6:87994545	A/C	rs144860417	GJB7
6:88123541	G/A	rs965639371	CFAP206
6:89891741	G/A	rs773405441	GABRR1
6:90372576	C/A	rs897000103	MDN1
6:90468546	C/T	rs756897005	MDN1
6:90604576	G/T		GJA10
6:93967903	T/G	rs146203229	EPHA7
6:97051613	G/C	rs147226482	FHL5

6:97246400	C/T	rs751202136	GPR63
6:99951712	G/A	rs375985216	USP45
6:100055023	C/T	rs200657499	PRDM13
6:101054639	C/T	rs751357098	ASCC3
6:102250221	C/T	rs749439536	GRIK2
6:106555347	C/A	rs75859409	PRDM1
6:106968820	T/G	rs777312523	CRYBG1
6:106992504	G/A	rs147164584	CRYBG1
6:108067977	C/T	rs1226255536	SCML4
6:109768306	G/A	rs750203059	MICAL1
6:109769447	C/A		MICAL1
6:109769950	A/G	rs757426218	MICAL1
6:109837666	A/C	rs756917093	AK9
6:111887733	G/A		TRAF3IP2
6:116442377	G/C	rs774396983	COL10A1,NT5DC1
6:116443023	C/T	rs145214720	COL10A1,NT5DC1
6:116833082	A/G		CALHM5,TRAPPC3L
6:116864503	C/T		CALHM4,TRAPPC3L
6:116950775	G/C	rs147003118	RSPH4A
6:117114041	A/G	rs1367464983	GPRC6A
6:117127876	C/T	rs748301096	GPRC6A
6:117232111	G/A	rs188277006	RFX6
6:121602832	T/A	rs201652810	TBC1D32
6:127636021	G/A	rs146146402	ECHDC1
6:127767872	C/T	rs747655022	KIAA0408,SOGA3
6:128326251	G/T		PTPRK
6:131188619	T/C	rs1161168181	EPB41L2
6:131944519	C/T	rs145843297	MED23
6:132014721	G/A	rs200513651	ENPP3
6:132910432	G/A	rs147580046	TAAR5
6:133093338	C/G		SLC18B1
6:135265094	G/A	rs755934699	ALDH8A1
6:136958542	A/G		MAP3K5
6:137143895	G/C		PEX7
6:137245391	G/A	rs1310064908	SLC35D3
6:137245785	A/C	rs144509540	SLC35D3
6:137468878	A/C		IL22RA2
6:137476179	G/T		IL22RA2
6:138745513	C/T		NHSL1
6:138752241	C/T	rs3734305	NHSL1
6:138753221	G/C	rs138276881	NHSL1
6:142468446	C/A	rs137966407	VTA1
6:142691613	A/G	rs199531526	ADGRG6
6:142723795	A/G	rs193295605	ADGRG6
6:143093570	C/A	rs764955376	HIVEP2
6:143093607	G/A	rs372948812	HIVEP2
6:144507985	C/G		STX11
6:146240560	G/A		SHPRH
6:148711294	G/A	rs376657388	SASH1
6:148852710	G/A	rs568821598	SASH1

6:148869611	G/A	rs757432307	SASH1
6:149783078	G/A	rs376716657	ZC3H12D
6:150005117	G/A	rs56348064	LATS1
6:150063580	C/G		NUP43
6:150715260	G/C	rs35430834	IYD
6:151121859	G/A	rs145017897	PLEKHG1
6:152461162	C/T	rs119103248	SYNE1
6:152631566	G/T	rs145899734	SYNE1
6:152668337	G/T	rs755560676	SYNE1
6:152716771	A/G	rs138766629	SYNE1
6:154411134	C/T	rs202022370	OPRM1
6:154412549	G/A	rs199984546	OPRM1
6:155561791	G/A	rs201811882	TIAM2
6:157431669	G/T		ARID1B
6:158066783	T/C		ZDHHC14
6:158534495	G/C	rs765711096	SERAC1
6:158534621	G/C	rs146410859	SERAC1
6:159086543	G/A	rs547797468	SYTL3
6:159086573	G/A	rs139337109	SYTL3
6:159129392	G/T	rs191078004	SYTL3
6:159404812	C/T	rs556783606	RSPH3
6:160197281	C/G	rs776242450	ACAT2
6:160199289	G/A	rs199875812	ACAT2
6:160231070	C/A	rs771275848	PNLDC1
6:160466779	G/C	rs1417183899	IGF2R
6:160645771	G/A	rs754257056	SLC22A2
6:162475167	T/G	rs9456735	PRKN
6:162864388	C/G	rs368134308	PRKN
6:167570672	C/G	rs773661514	GPR31
6:167570835	C/T	rs779831524	GPR31
6:167753855	A/T	rs1355690250	TTLL2
6:168352686	A/G	rs370552931	AFDN
6:168443396	G/A	rs147788292	KIF25
6:168465664	C/T	rs751929953	FRMD1
6:168465691	C/T	rs199740559	FRMD1
6:168720072	T/C		DACT2
6:169648668	G/T		THBS2
6:170592614	C/G	rs202029364	DLL1
6:170598811	C/T	rs1420409055	DLL1
6:170858118	C/T	rs1303286485	PSMB1
7:635796	C/T	rs200069843	PRKAR1B
7:878527	G/A	rs770554143	SUN1
7:905604	G/A	rs373401176	SUN1
7:938580	G/A	rs377385277	ADAP1
7:1028337	T/G	rs201684384	CYP2W1
7:1272814	T/C	rs755736495	UNCX
7:1272831	G/A	rs567702156	UNCX
7:1488283	A/G		MICALL2
7:1588278	C/G		TMEM184A
7:1590603	G/A	rs753257184	TMEM184A

7:1786469	G/A	rs933268572	ELFN1
7:2275160	A/T	rs771823610	MRM2
7:2613102	G/A	rs746212612	IQCE
7:2958127	G/A	rs769192567	CARD11
7:4188947	C/T	rs146602325	SDK1
7:4245622	A/G		SDK1
7:4823038	T/A	rs1436357315	AP5Z1
7:4827898	G/A	rs370116509	AP5Z1
7:4830476	C/T	rs759903205	AP5Z1
7:4855955	G/A	rs370284601	RADIL
7:5415705	G/A	rs773725606	TNRC18
7:5645091	C/T	rs767397734	FSCN1
7:5997566	T/C	rs750499560	RSPH10B
7:6022480	C/T	rs201671325	PMS2
7:6043689	T/C		PMS2
7:6062997	T/C	rs749728733	AIMP2,EIF2AK1
7:6190119	G/T	rs199521734	USP42
7:6464390	G/C	rs757164150	DAGLB
7:6547672	C/T	rs773330756	GRID2IP
7:6808272	T/C	rs1254632956	RSPH10B2
7:7413030	C/T	rs571848025	COL28A1
7:7472168	G/A	rs201056877	COL28A1
7:16918181	G/A		AGR3
7:17379098	T/C	rs141403259	AHR
7:20683103	G/T		ABCB5
7:20698156	G/A	rs200262701	ABCB5
7:21747413	G/T		DNAH11
7:21951369	C/G	rs779644053	CDCA7L
7:22202059	T/C	rs754536871	RAPGEF5
7:23724192	C/T	rs752705562	FAM221A
7:24745898	A/T		GSDME
7:25163375	T/C	rs11548785	CYCS
7:26224805	A/G	rs778855968	NFE2L3
7:27147639	G/T	rs375670849	HOXA3
7:27148297	G/C	rs144351903	HOXA3
7:28858826	G/T		CREB5
7:29126150	A/G	rs143508754	CPVL
7:29605949	G/A	rs752329341	PRR15
7:30951672	G/A	rs200496523	AQP1
7:30951890	C/A	rs762241529	AQP1
7:31746868	G/A	rs147297649	PPP1R17
7:32620433	C/T	rs762532514	AVL9
7:33035786	G/A	rs748874793	FKBP9
7:33044898	G/C		FKBP9
7:34182859	T/C	rs376348056	BMPER
7:35922130	T/C	rs1396052968	SEPTIN7
7:37780300	C/T	rs758564434	GPR141
7:37903980	C/T	rs200863038	NME8
7:37989949	C/T	rs139510957	EPDR1
7:40314236	G/A		SUGCT

7:42006177	C/T	rs1156987879	GLI3
7:44113298	G/A	rs368970693	POLM
7:44801394	C/T		ZMIZ2
7:44806101	G/A		ZMIZ2
7:45016492	G/A	rs199877330	MYO1G
7:45016587	G/T	rs201090665	MYO1G
7:45115412	A/G	rs201559289	CCM2
7:45124860	G/A		NACAD
7:47333408	T/A		TNS3
7:47408880	G/A	rs148721285	TNS3
7:50444468	T/C	rs764847531	IKZF1
7:50514946	A/G	rs200586199	FIGNL1
7:55233129	G/A	rs753315940	EGFR
7:55240780	G/A	rs150423237	EGFR
7:55269438	G/T		EGFR
7:56146137	C/T	rs764443878	SUMF2
7:64292303	G/T	rs138905610	ZNF138
7:65444769	G/A	rs121918181	GUSB
7:65551751	G/A	rs751590073	ASL
7:65554675	C/T	rs778692269	ASL
7:66532275	A/G	rs200301906	TYW1
7:72722780	A/G		NSUN5
7:72734235	G/A	rs374313766	TRIM50
7:72744255	G/C	rs1064197	FKBP6
7:72985664	C/A	rs145601505	TBL2
7:73010572	C/T	rs201875813	MLXIPL
7:73012030	C/A	rs782021958	MLXIPL
7:73152662	C/T		ABHD11
7:73458241	G/A	rs145669576	ELN
7:73500129	A/G		LIMK1
7:73511070	A/G	rs782794386	LIMK1
7:73768231	G/A	rs369942236	CLIP2
7:74005280	T/C		GTF2IRD1
7:75070334	C/T	rs182076484	POM121C
7:75172253	T/C	rs138335070	HIP1
7:75192493	T/C		HIP1
7:75210555	G/A	rs782057576	HIP1
7:75615273	G/A	rs779082897	POR
7:75889515	G/A		SRRM3
7:76144442	A/G	rs778284234	UPK3B
7:76240877	A/T	rs73365247	POMZP3
7:76909810	G/A		CCDC146
7:77247879	G/T	rs751126065	PTPN12
7:80292426	G/A	rs138897347	CD36
7:82451822	C/T	rs370849099	PCLO
7:82579999	T/C	rs201475178	PCLO
7:82595742	T/C	rs28680905	PCLO
7:82997213	C/T		SEMA3E
7:87035723	C/T	rs866301089	ABCB4
7:87047928	G/T		ABCB4

7:87079396	A/G		ABCB4
7:87165817	G/C	rs201744003	ABCB1
7:88956731	A/C		ZNF804B
7:88956739	G/C		ZNF804B
7:90547013	C/T	rs376270298	CDK14
7:91631785	T/C	rs372844164	AKAP9
7:91737915	C/A	rs866733322	AKAP9
7:91936798	G/A	rs759267009	ANKIB1
7:92122443	C/T	rs141650598	PEX1
7:92732725	A/G	rs142611434	SAMD9
7:92763216	C/T	rs147903234	SAMD9L
7:93090257	C/T	rs143764918	CALCR
7:94054532	G/A	rs200331961	COL1A2
7:94178855	A/C		CASD1
7:95221357	T/C		PDK4
7:96653754	G/A	rs765089599	DLX5
7:97484730	T/C		ASNS
7:97498251	C/A	rs773486639	ASNS
7:99022542	C/T	rs139956151	ATP5MF-PTCD1,PTCD1
7:99026740	G/A	rs140372739	ATP5MF-PTCD1,PTCD1
7:99032424	T/G	rs368346878	ATP5MF-PTCD1,PTCD1
7:99247842	T/C	rs755094756	CYP3A5
7:99264638	A/C	rs746469422	CYP3A5
7:99367788	C/T	rs72552799	CYP3A4
7:99516799	G/A	rs1200258795	TRIM4
7:99527000	T/A	rs1220817765	GJC3
7:99695317	C/T	rs777004718	MCM7
7:99711386	A/G	rs750473553	TAF6
7:99753382	G/A	rs1469433483	MAP11
7:99758527	G/A	rs550951408	GAL3ST4
7:99786136	C/T		STAG3
7:100151752	C/T	rs760794616	AGFG2
7:100159897	C/T	rs139318752	AGFG2
7:100201805	T/C		PCOLCE
7:100225917	C/T	rs80338885	TFR2
7:100411629	G/A	rs778356891	EPHB4
7:100486851	C/G		UFSP1
7:100841590	C/G	rs141385847	MOGAT3
7:102106337	C/T		LRWD1
7:102180527	A/G	rs1466748748	POLR2J3
7:102232778	G/A	rs1249861337	RASA4
7:102553634	A/C	rs757122324	FBXL13,LRRC17
7:102572380	C/T	rs376548338	FBXL13,LRRC17
7:102743998	A/C	rs369637374	NAPEPLD
7:103048411	C/T	rs765458806	SLC26A5
7:103151360	G/A	rs202166176	RELN
7:103193936	A/G		RELN
7:103205800	G/A	rs1056128260	RELN
7:103275986	G/A	rs144235468	RELN
7:104377235	C/T	rs919661761	LHFPL3

7:104717411	G/A	rs144013371	KMT2E
7:105205777	C/T	rs187666745	RINT1
7:106508070	A/G		PIK3CG
7:107188772	G/A	rs150351852	COG5
7:107224347	T/A	rs377633207	BCAP29
7:107605082	C/T	rs138019069	LAMB1
7:107832191	C/T	rs139663718	NRCAM
7:107872889	G/A	rs749483134	NRCAM
7:108137199	C/T	rs1340933251	PNPLA8
7:111368336	C/A	rs752915503	DOCK4
7:113518955	T/C		PPP1R3A
7:114294072	C/T	rs139649711	FOXP2
7:114329899	T/C	rs750287608	FOXP2
7:116199267	G/A	rs150368249	CAV1
7:117250609	G/A	rs184724618	CFTR
7:117359623	C/T	rs571557190	CTTNBP2
7:121011451	C/G	rs140177929	FAM3C
7:121636537	G/A		PTPRZ1
7:121659205	G/A		PTPRZ1
7:121680897	C/T	rs138349768	PTPRZ1
7:122338257	A/T	rs777392998	CADPS2,RNF133
7:123097463	G/C		IQUB
7:123143044	G/A	rs764624584	IQUB
7:123332783	G/A	rs751264121	WASL
7:126173032	A/C	rs200861849	GRM8
7:127732090	G/A		SND1
7:128315799	C/T	rs754390092	FAM71F2
7:128413918	C/T	rs1239463905	OPN1SW
7:128446344	C/A	rs756839463	CCDC136
7:128480899	T/C	rs774963796	FLNC
7:128845210	C/T	rs142599757	SMO
7:129125554	G/A	rs760420426	STRIP2
7:129668767	A/T	rs1395644068	ZC3HC1
7:129922020	C/G		CPA2
7:133884008	C/T	rs139500250	LRGUK
7:133884144	C/T	rs188028215	LRGUK
7:133991425	G/C	rs975345763	SLC35B4
7:134632411	C/T	rs141179317	CALD1
7:134717640	G/A	rs901299301	AGBL3
7:134719537	G/A	rs146250942	AGBL3
7:134879726	G/A	rs367680418	WDR91
7:135377092	C/T		SLC13A4
7:135392902	C/A		SLC13A4
7:137293794	C/T	rs747206720	DGKI
7:137593045	G/A	rs759528760	CREB3L2
7:138341230	G/A	rs143732886	SVOPL
7:138579207	C/A		KIAA1549
7:138774434	G/A	rs142788152	ZC3HAV1
7:138968412	T/G	rs201511798	UBN2
7:139257902	G/A	rs1326616785	HIPK2

7:139315972	G/A	rs778794392	HIPK2
7:139657487	G/A	rs141602429	TBXAS1
7:140269483	T/G	rs767880041	DENND2A
7:140710397	G/A	rs745809255	MRPS33
7:141315304	C/G	rs755620097	AGK
7:141424935	G/A	rs979662248	WEE2
7:141730544	C/T	rs545222714	MGAM
7:141756665	C/T	rs115080419	MGAM
7:141954944	G/A	rs749338729	PRSS58
7:142723557	G/C	rs772703280	OR9A2
7:143018463	A/C	rs764015801	CLCN1
7:143573613	A/T	rs896907336	TCAF1
7:143633023	G/A	rs199756492	OR2F2
7:144064347	C/G		ARHGEF5
7:148709270	T/C	rs369248381	PDIA4
7:148909524	G/A	rs149628742	ZNF282
7:149571195	C/T		ATP6V0E2
7:149981873	C/T		ACTR3C
7:150068916	A/G		REPIN1
7:150554147	C/T	rs370771602	AOC1
7:150555982	C/T	rs200258999	AOC1
7:150772536	C/T	rs141479576	SLC4A2
7:150884073	G/A	rs142736544	ASB10
7:151082227	G/A	rs549406540	WDR86
7:151133276	C/T	rs779736230	CRYGN
7:151873293	G/A	rs61730545	KMT2C
7:151878006	A/T	rs769592355	KMT2C
7:154739639	G/A	rs1211359069	PAXIP1
7:156759004	G/A	rs114968937	NOM1
7:158528253	C/T	rs139531934	ESYT2
7:158591753	G/A	rs141870305	ESYT2
7:158705752	C/T		WDR60
7:158902547	G/A	rs542501834	VIPR2
8:442428	G/C	rs115717719	TDRP
8:1871965	G/C		ARHGEF10
8:1950829	C/G		KBTBD11
8:2046805	C/T	rs147766516	MYOM2
8:2065676	C/T	rs186595396	MYOM2
8:2092650	C/G	rs145385079	MYOM2
8:2857494	G/A	rs146539567	CSMD1
8:3216688	A/G	rs759586231	CSMD1
8:3245142	C/T	rs181981898	CSMD1
8:6357446	C/G	rs759545352	ANGPT2,MCPH1
8:10480546	G/A	rs150931842	RP1L1
8:10555244	A/G	rs187767039	C8orf74
8:11142456	C/A	rs143537669	MTMR9
8:11188770	G/T	rs147745991	SLC35G5
8:11281911	T/C		FAM167A
8:11420559	G/A	rs373565757	BLK
8:11421456	C/A	rs776438085	BLK

8:11421534	G/A		BLK
8:12878588	G/A	rs769676844	TRMT9B
8:16035410	C/T	rs143927568	MSR1
8:17230690	C/G	rs753634396	MTMR7
8:17406339	T/C	rs142593316	SLC7A2
8:17447267	C/T	rs373853328	PDGFRL
8:17500131	C/T	rs148981308	PDGFRL
8:17510972	G/C	rs181719146	MTUS1
8:17794873	C/G	rs201776854	PCM1
8:17820699	G/C	rs200495166	PCM1
8:17869244	G/C	rs755606707	PCM1
8:17917141	T/C	rs371154344	ASAH1
8:17924735	G/T	rs199785411	ASAH1
8:18257610	C/T	rs763283305	NAT2
8:19680905	A/G	rs1463234655	INTS10
8:20036635	T/C	rs149553416	SLC18A1
8:20038418	G/A	rs201099277	SLC18A1
8:21767389	A/C		DOK2
8:21955034	C/T	rs190462850	FAM160B2
8:21986332	G/A	rs371390059	HR
8:21996570	T/C	rs1460016218	REEP4
8:22173818	G/A	rs372146015	PIWIL2
8:22418864	G/T	rs368810763	SORBS3
8:22777725	G/A	rs371118048	PEBP4
8:23156435	A/G	rs148111808	LOXL2
8:23190934	G/A	rs754340605	LOXL2
8:23217656	C/T	rs3736019	LOXL2
8:23702513	C/T	rs867772914	STC1
8:24190198	C/T		ADAM28
8:24333949	C/T	rs148539948	ADAM7
8:26721949	C/G	rs780932474	ADRA1A
8:27294942	A/G	rs769031656	PTK2B
8:27373287	T/C		EPHX2
8:27382881	C/T	rs76549048	EPHX2
8:27457512	T/G	rs9331936	CLU
8:27528738	C/T	rs200949728	SCARA3
8:27650267	T/C	rs751032624	ESCO2
8:27680615	T/A	rs62001048	PBK
8:27995239	C/T	rs775947064	ELP3
8:28384971	C/T	rs771937650	FZD3
8:28928068	C/T	rs200053841	KIF13B
8:28932850	C/T	rs767471422	KIF13B
8:29931557	G/A	rs181979095	SARAF
8:29990136	G/C	rs530929110	LEPROTL1,MBOAT4
8:31004938	T/C		WRN
8:31497675	G/C	rs982442612	NRG1
8:32616923	T/C		NRG1
8:32620785	T/A		NRG1
8:32621349	A/G	rs139436076	NRG1
8:33247152	G/C	rs142881205	FUT10

8:33346630	G/A	rs201215286	MAK16
8:37688318	C/T	rs1365947422	ADGRA2
8:37690532	C/A	rs1387770557	ADGRA2
8:37720486	A/G	rs151011205	RAB11FIP1
8:37734837	T/A	rs767178178	RAB11FIP1
8:37756956	A/T		RAB11FIP1
8:37793286	G/C	rs202216080	GOT1L1
8:38091917	G/T	rs141644282	DDHD2
8:38831978	T/C	rs777899358	HTRA4
8:39564310	T/A	rs141828875	ADAM18
8:39627014	C/A		ADAM2
8:39634616	A/G		ADAM2
8:41753901	C/A		ANK1
8:42287661	T/G		SLC20A2
8:42611002	T/C	rs16891583	CHRNA6
8:42729141	T/A		RNF170
8:48586395	T/G		SPIDR
8:48647880	G/C		SPIDR
8:48798507	T/C	rs368553930	PRKDC
8:49642344	G/T	rs1191688008	EFCAB1
8:53570428	G/C	rs780863338	RB1CC1
8:55534713	G/A	rs145691085	RP1
8:57080018	T/C	rs148894075	PLAG1
8:59409220	C/A	rs528732489	CYP7A1
8:62416016	C/T	rs147092046	ASPH
8:62489375	G/A	rs139441811	ASPH
8:66605940	T/C		MTFR1
8:66989035	G/T	rs142615334	DNAJC5B
8:67040594	G/A	rs758190155	TRIM55
8:67790904	G/C		MCMDC2
8:68107721	G/A	rs778119559	CSPP1
8:69104619	C/T	rs61753704	PREX2
8:69129915	C/T	rs752310428	PREX2
8:69445285	G/A	rs561667412	C8orf34
8:70498639	C/G	rs147245253	SULF1
8:72977723	G/A	rs144784837	TRPA1
8:74526080	C/T	rs746341421	STAU2
8:77775852	C/G		ZFHX4
8:81599562	T/G		ZNF704
8:82644982	G/A	rs1563616666	CHMP4C
8:86049759	A/C		LRRCC1
8:86352091	A/G	rs542149751	CA3
8:87125964	G/A	rs762889203	ATP6V0D2
8:87645092	C/T	rs147876778	CNGB3
8:87660067	C/T	rs760502027	CNGB3
8:87666259	C/T	rs778013642	CNGB3
8:94770788	C/T	rs1291015693	TMEM67
8:94821098	C/T	rs1483136796	TMEM67
8:94821387	C/G		TMEM67
8:95172353	C/T		CDH17

8:95189843	C/G	rs762951650	CDH17
8:95746906	C/T	rs147410800	DPY19L4
8:95793376	A/G		DPY19L4
8:95800261	T/C		DPY19L4
8:95854685	C/G	rs370672430	INTS8
8:95952352	G/A	rs779919334	TP53INP1
8:95953060	A/C	rs531048329	TP53INP1
8:97156855	G/A	rs140782427	GDF6
8:97244060	A/T	rs139283183	UQCRB
8:97614719	C/T	rs772069532	SDC2
8:97797538	G/A	rs34088584	CPQ
8:97847287	A/T		CPQ
8:98698953	C/T	rs374847603	MTDH
8:98943526	G/A	rs370342485	MATN2
8:99045883	G/C	rs181639683	MATN2
8:99140662	C/T	rs3824145	POP1
8:99169954	G/C	rs774510819	POP1
8:99961328	C/A	rs1476917342	OSR2
8:100147957	A/G	rs143205296	VPS13B
8:101011612	G/A	rs3133711	RGS22
8:101609014	A/C	rs139602322	SNX31
8:104337497	C/A	rs142694816	FZD6
8:104415406	G/A	rs374025789	SLC25A32
8:104897539	T/C	rs201322998	RIMS2
8:104898074	G/A	rs199608093	RIMS2
8:105105726	G/A		RIMS2
8:105440273	T/C	rs201457190	DPYS
8:105503481	G/A	rs139489607	LRP12
8:106815396	A/T	rs201729935	ZFPM2
8:107773321	C/G		ABRA
8:108264099	C/T	rs377442517	ANGPT1
8:113303799	A/G	rs138666266	CSMD3
8:114326813	T/C	rs144647346	CSMD3
8:116617037	G/C	rs1563638257	TRPS1
8:118169965	T/C	rs142407509	SLC30A8
8:119945190	C/T		TNFRSF11B
8:120977534	G/A	rs373302616	DEPTOR
8:121015350	G/A	rs772667396	DEPTOR
8:121174707	A/C	rs753217873	COL14A1
8:121463278	T/C	rs1345794370	MTBP
8:123964510	C/T		ZHX2
8:124096557	C/T	rs763893402	TBC1D31
8:124105961	A/G	rs777440242	TBC1D31
8:124109635	G/A	rs142466644	TBC1D31
8:124266332	T/G	rs762770128	ZHX1,ZHX1-C8orf76
8:125072945	G/A	rs200333104	FER1L6
8:125083870	G/A	rs202097932	FER1L6
8:125464024	C/T	rs1050325334	TRMT12
8:125989258	G/A	rs765923254	ZNF572
8:125989558	C/G	rs374083749	ZNF572

8:126052110	T/C	rs1381772866	WASHC5
8:126369957	G/A	rs756476091	NSMCE2
8:128752924	C/T	rs200431478	MYC
8:131792725	C/A	rs1401397666	ADCY8
8:133101781	A/T		HHLA1
8:133107983	C/T	rs1032428923	HHLA1
8:133141822	G/T	rs114095081	KCNQ3
8:133883773	G/A	rs114781869	TG
8:133910471	G/A	rs116119508	TG
8:134060111	C/T		SLA,TG
8:134256618	C/T	rs774885520	NDRG1
8:139263221	G/T		FAM135B
8:139601522	G/A	rs755307992	COL22A1
8:139838972	G/A	rs138591562	COL22A1
8:141294025	C/T	rs147044768	TRAPPC9
8:141756950	C/T	rs182075508	PTK2
8:142229761	C/T	rs61995883	SLC45A4
8:143996464	T/C	rs544064549	CYP11B2
8:144512357	C/A		MAFA
8:144650013	G/C	rs201550753	MROH6
8:144659492	C/T	rs756799130	NAPRT
8:144695921	C/T	rs770039732	TSTA3
8:144696827	G/A	rs774763099	TSTA3
8:144809550	T/A	rs115863410	FAM83H
8:144810594	G/T	rs536088856	FAM83H
8:144873405	T/A	rs200643687	SCRIB
8:144942188	C/T	rs187594766	EPPK1
8:144942362	G/A	rs371322120	EPPK1
8:144945455	G/C		EPPK1
8:144992378	C/T	rs201419047	PLEC
8:144993053	G/A	rs202175941	PLEC
8:144994766	G/A	rs376638828	PLEC
8:144995559	G/C	rs62641758	PLEC
8:144998175	C/G	rs202190540	PLEC
8:144999527	C/T	rs1047641700	PLEC
8:145003969	C/T	rs371122460	PLEC
8:145020559	A/G		PLEC
8:145134940	G/A	rs144241636	EXOSC4
8:145584649	G/A		SLC52A2
8:145620684	G/A	rs782156961	CPSF1
8:145700346	T/A	rs112028242	FOXH1
8:145722584	G/A	rs201175578	PPP1R16A
8:145748143	G/C	rs376131634	LRRC14,LRRC24
8:145806554	C/A		ARHGAP39
8:145947922	T/C	rs368493787	ZNF251
9:117639	A/G	rs753151292	FOXD4
9:173327	C/G		CBWD1
9:396881	A/G	rs140148619	DOCK8
9:712318	G/A		KANK1
9:744512	A/G	rs751377528	KANK1

9:4118589	G/A	rs757448015	GLIS3
9:4697835	G/A		CDC37L1
9:4722574	T/A	rs141767297	AK3
9:4841315	T/C	rs149003954	RCL1
9:5922316	G/A	rs41281757	KIAA2026
9:5969431	C/T	rs200706146	KIAA2026
9:6592208	C/G		GLDC
9:12708979	C/T	rs138272660	TYRP1
9:13119542	C/T	rs202112833	MPDZ
9:13193230	G/C	rs149265684	MPDZ
9:14819361	T/C	rs778240153	FREM1
9:14859421	C/G	rs745637932	FREM1
9:17235770	G/C	rs200513513	CNTLN
9:17342376	A/T	rs200118767	CNTLN
9:18905787	G/A	rs778958555	ADAMTSL1
9:18906784	G/A	rs750373794	ADAMTSL1
9:19080628	A/C		HAUS6
9:19550151	G/C		SLC24A2
9:20866989	C/T		FOCAD
9:20929433	C/A	rs143958174	FOCAD
9:21818036	A/G	rs761662457	MTAP
9:26928218	C/G	rs763290476	PLAA
9:32632376	G/A	rs772629563	TAF1L
9:32633344	T/C	rs1441197926	TAF1L
9:32633900	G/A	rs1407746743	TAF1L
9:33941786	G/A	rs140922028	UBAP2
9:33986766	C/T	rs574338817	UBAP2
9:34371812	A/G	rs770121709	MYORG
9:34489429	C/T	rs116938457	DNAI1
9:34625754	A/G	rs766390398	ARID3C
9:35091687	G/A	rs748093757	PIGO
9:35547680	C/T	rs891776488	RUSC2
9:35560083	G/C		RUSC2
9:35738146	C/T	rs142621039	GBA2
9:35801720	G/A	rs114115939	NPR2
9:35810673	C/T	rs138285220	SPAG8
9:36217409	C/G		GNE
9:36671123	A/T	rs147882939	MELK
9:37429747	G/A	rs200106110	GRHPR
9:37500891	T/C		POLR1E
9:38396346	G/A	rs199527495	ALDH1B1
9:69386021	T/G		ANKRD20A4P
9:71080038	A/G		PGM5
9:71155573	A/G	rs767457213	TMEM252
9:71155588	G/C		TMEM252
9:71851900	G/C	rs142254605	TJP2
9:71992647	C/T	rs775237803	FAM189A2
9:72723349	G/A		MAMDC2
9:72938985	A/T		SMC5
9:73240148	G/C	rs367948487	TRPM3

9:74360185	C/A		CEMIP2
9:75543859	G/A	rs371735738	ALDH1A1
9:77407568	G/C		TRPM6
9:78803562	C/A	rs201930247	PCSK5
9:78804567	C/T	rs202203226	PCSK5
9:79259691	C/A	rs41310045	PRUNE2
9:79635796	T/A		FOXB2
9:80851289	G/C		CEP78
9:80919694	G/A		PSAT1
9:85624574	T/G		RASEF
9:86615956	C/T	rs35819647	RMI1
9:90258337	T/C		DAPK1
9:90265001	G/A	rs370724837	DAPK1
9:90322132	G/C	rs756825105	DAPK1
9:94486693	C/T	rs34431454	ROR2
9:95400370	G/A	rs2277168	IPPK
9:96098219	G/A	rs200912595	C9orf129
9:97321324	C/T	rs763789175	FBP2
9:97346921	C/G		FBP2
9:97844993	T/C	rs150812918	AOPEP
9:98238363	T/C	rs1332335605	PTCH1
9:100364849	G/A	rs149077979	TSTD2
9:100372697	G/A	rs373228155	TSTD2
9:100417198	G/A	rs757052791	NCBP1
9:100850008	T/C	rs1246397297	TRIM14
9:101798485	A/C	rs139815955	COL15A1
9:101831998	C/T	rs147824264	COL15A1
9:102992084	C/G	rs774469922	INVS
9:104184181	G/C	rs78340951	ALDOB
9:104340093	C/T	rs866677056	GRIN3A
9:104432814	A/G	rs758373461	GRIN3A
9:104433336	C/A	rs373415839	GRIN3A
9:106878589	G/T	rs376298987	SMC2
9:107298827	A/G	rs61742435	OR13C3
9:107360910	G/A	rs7852858	OR13C5
9:107583722	C/T	rs763060795	ABCA1
9:108424974	C/T	rs146125593	TAL2
9:108484820	G/T	rs1383543447	TMEM38B
9:111624915	G/A	rs759786635	ACTL7A
9:111625023	G/A	rs139001396	ACTL7A
9:111644016	T/C		ELP1
9:111673437	G/A	rs139703788	ELP1
9:111698617	A/G	rs117239473	ABITRAM
9:111741680	G/A	rs762309755	CTNNAL1
9:112189329	C/G	rs147767465	PTPN3
9:113013706	T/C	rs749648542	TXN
9:113149673	C/T	rs371503132	SVEP1
9:113173587	G/C	rs201520602	SVEP1
9:114195576	G/A		ECPAS
9:114332387	A/G		PTGR1,ZNF483

9:115931853	G/A		FKBP15
9:116116523	A/G	rs202215549	BSPRY
9:116130552	G/A	rs202046262	BSPRY
9:116152771	A/T	rs1057521379	ALAD
9:116770808	C/T	rs531098757	ZNF618
9:117051003	G/C	rs149434700	COL27A1
9:117063983	C/T	rs139690392	COL27A1
9:117103900	T/C	rs2787344	AKNA
9:117188522	G/A	rs56059137	WHRN
9:117827057	T/A		TNC
9:117838320	C/T	rs139800185	TNC
9:121929534	C/T	rs769963438	BRINP1
9:123758537	C/A		C5
9:124971951	G/A	rs367663922	LHX6
9:124976338	G/A	rs935336920	LHX6
9:125154602	C/A	rs145989297	PTGS1
9:125860105	T/C		RABGAP1
9:127549455	A/G	rs200585902	OLFML2A
9:127990237	C/G	rs369057277	RABEPK
9:130111248	G/A	rs769788720	GARNL3
9:130159794	G/T	rs561202364	SLC2A8
9:130509437	G/T		SH2D3C
9:130511737	G/A	rs143231219	SH2D3C
9:131016935	G/A	rs758567328	DNM1
9:131261359	G/A	rs144521735	ODF2
9:131267089	C/G	rs150246404	GLE1
9:131367755	C/T	rs771862017	SPTAN1
9:131388878	A/G	rs144289764	SPTAN1
9:131588317	T/C		SPOUT1
9:131588362	A/T	rs1317353958	SPOUT1
9:131709189	T/C	rs1332281324	DOLK
9:131767741	A/G	rs147294504	NUP188
9:132482980	G/A	rs773930013	PRRX2
9:133569288	C/T		EXOSC2
9:133928248	C/T		LAMC3
9:133928278	C/T	rs142041428	LAMC3
9:133961040	C/A	rs141497885	LAMC3
9:134106048	G/A	rs142328071	NUP214
9:134351496	G/A	rs778936078	PRRC2B
9:135523937	G/A	rs1202807389	DDX31
9:135553407	C/T	rs757337001	GTF3C4
9:135762799	G/A	rs199653353	SPACA9
9:135927470	G/T		GTF3C5
9:135937396	C/T	rs201383133	CEL
9:135942279	G/A	rs776449870	CEL
9:135944565	A/C	rs1040143995	CEL
9:136029137	T/A	rs35403335	GBGT1
9:136029280	C/T	rs143563851	GBGT1
9:136260878	G/A	rs144537926	STKLD1
9:136313842	C/T	rs143568784	ADAMTS13

9:136570106	C/G		SARDH
9:136654461	A/C	rs145444804	VAV2
9:137676914	C/G	rs150539264	COL5A1
9:138642800	G/A	rs748873120	KCNT1
9:138664648	C/T	rs538197009	KCNT1
9:138905065	C/T	rs200168413	NACC2
9:139252487	C/T	rs147843984	GPSM1
9:139417506	T/C		NOTCH1
9:139568274	A/G		AGPAT2
9:139616728	G/A	rs140623977	DIPK1B
9:139753715	G/A	rs755894109	MAMDC4
9:139840128	C/T	rs774819278	C8G
9:139925521	A/G		C9orf139,FUT7
9:140093519	C/T	rs757344465	TPRN
9:140109375	G/A	rs145420455	NDOR1
9:140109640	G/A	rs374525491	NDOR1
9:140127302	T/C	rs371519982	SLC34A3
9:140129115	G/A	rs758354118	SLC34A3
9:140130521	C/T	rs145029982	SLC34A3
9:140130530	G/C	rs149389629	SLC34A3
9:140459409	G/A	rs1385458993	DPH7
9:140685411	G/A	rs777770909	EHMT1
9:140917876	A/T	rs550100275	CACNA1B
9:141015185	G/A	rs199622481	CACNA1B
9:141015991	G/A	rs774017061	CACNA1B
10:3146076	C/T	rs373948435	PFKP
10:4875643	G/T	rs769752726	AKR1E2
10:5009195	A/T	rs1177127624	AKR1C1
10:5037593	A/C	rs782680340	AKR1C2
10:5038045	G/A	rs782231649	AKR1C2
10:5435803	C/T	rs139311920	TUBAL3
10:5435983	C/T	rs41289265	TUBAL3
10:5494334	G/A	rs146200933	NET1
10:5693260	A/G	rs139712171	ASB13
10:6005718	G/A		IL15RA
10:6257261	G/A	rs149555675	PFKFB3
10:7618952	G/A	rs180863175	ITIH5
10:7621784	G/A	rs777190590	ITIH5
10:8100506	C/G	rs143627754	GATA3
10:11543119	T/C	rs61755070	USP6NL
10:11551601	G/A	rs771894885	USP6NL
10:11911663	G/C	rs1280113565	PROSER2
10:13658552	A/G	rs1179720130	PRPF18
10:13699005	T/C		FRMD4A
10:14961757	C/G		DCLRE1C
10:15120975	A/G	rs750500301	ACBD7
10:15146119	A/T		RPP38
10:15256368	C/T	rs143772901	FAM171A1
10:16955908	C/T	rs145799931	CUBN
10:17645565	T/C	rs782784087	HACD1

10:18254445	G/C		SLC39A12
10:18823118	G/A	rs370564617	CACNB2
10:18828427	G/A	rs747168096	CACNB2
10:20106075	G/C	rs150292075	PLXDC2
10:21108412	G/A	rs772963438	NEBL
10:21134212	T/G	rs1346871852	NEBL
10:21804386	C/T	rs200920908	SKIDA1
10:23482808	C/A	rs181911810	PTF1A
10:24833405	C/G	rs542316251	KIAA1217
10:24834859	G/A	rs143473670	KIAA1217
10:24889799	G/C	rs144895449	ARHGAP21
10:25279513	A/G		ENKUR
10:26457719	G/A	rs149468017	MYO3A
10:27459404	A/G	rs769200738	MASTL
10:29777673	G/A	rs200637850	SVIL
10:29818697	C/T	rs144573592	SVIL
10:29821081	G/A	rs143918159	SVIL
10:29824955	C/T	rs372012664	SVIL
10:29843750	C/T	rs771476631	SVIL
10:31799613	C/G		ZEB1
10:31812916	A/G	rs750281534	ZEB1
10:33469180	C/T	rs548175518	NRP1
10:33510724	C/T	rs755346095	NRP1
10:35858012	A/G	rs761914460	CCNY
10:43088671	G/A	rs145842576	ZNF33B
10:43882719	T/C		HNRNPF
10:45430124	G/A	rs201346225	TMEM72
10:45430284	A/T	rs150081288	TMEM72
10:45487381	C/T	rs146647075	RASSF4
10:45487418	G/C	rs767125140	RASSF4
10:49450328	C/T	rs1389065639	FRMPD2
10:49687709	G/C		ARHGAP22
10:49687805	C/A	rs760998236	ARHGAP22
10:50122059	G/A	rs373215473	LRRC18,WDFY4
10:50708599	C/T	rs41549213	ERCC6
10:50952163	A/T		OGDHL
10:55570338	T/C	rs754659921	PCDH15
10:55582900	T/C	rs151309770	PCDH15
10:55587210	G/T		PCDH15
10:55996616	C/G	rs747921916	PCDH15
10:59956406	C/T	rs746974757	IPMK
10:61011383	C/T	rs144555922	FAM13C
10:61830270	G/A	rs777814492	ANK3
10:61840327	T/C	rs139982397	ANK3
10:63959567	G/A	rs373879136	RTKN2
10:64136072	G/C	rs79142251	ZNF365
10:64949190	C/G		JMJD1C
10:64967249	T/A	rs139881253	JMJD1C
10:64974216	T/C	rs771305607	JMJD1C
10:67680240	C/T	rs1467135109	CTNNA3

10:67726423	T/C	rs776814237	CTNNA3
10:67862992	C/T	rs77165728	CTNNA3
10:69667814	A/G		SIRT1
10:69881773	A/G	rs573684358	MYPN
10:69921492	G/A	rs141031460	MYPN
10:69934077	C/T	rs138583865	MYPN
10:69955255	C/T	rs151282801	MYPN
10:70045158	T/C	rs1298029444	PBLD
10:70178844	T/C	rs1301590232	DNA2
10:70218918	G/C	rs751031650	DNA2
10:70641825	C/T	rs748648893	STOX1
10:71020966	A/C	rs770292279	HKDC1
10:71139817	G/A		HK1
10:72026028	C/T	rs201350017	NPFFR1
10:72136238	C/T	rs80070876	LRRC20
10:72294504	G/A	rs760606311	PALD1
10:72360130	G/A	rs201468340	PRF1
10:72517788	G/A		ADAMTS14
10:73044596	C/T	rs141063931	UNC5B
10:73464860	A/G	rs372401651	CDH23
10:73491758	G/A	rs1478583640	C10orf105,CDH23
10:73567275	G/A	rs201076440	CDH23
10:74684311	G/A	rs186007062	OIT3
10:74953294	C/G	rs533399071	FAM149B1
10:75407531	C/T	rs372058702	SYNPO2L
10:75434449	C/T	rs531051699	AGAP5
10:75532416	C/T		FUT11
10:75533410	G/A	rs199728665	FUT11
10:75842226	G/A		VCL
10:75865055	C/A	rs773421444	VCL
10:75871826	G/A	rs199751261	VCL
10:76780900	C/T	rs147643220	KAT6B
10:76788454	A/G		KAT6B
10:76788834	C/T	rs780199206	KAT6B
10:76789813	C/T	rs780866364	KAT6B
10:76797703	G/A	rs765918480	DUPD1
10:79567613	C/G	rs151299676	DLG5
10:82298140	A/C		SH2D4B
10:86273773	C/T	rs886781875	CCSER2
10:88418433	T/C	rs769509506	OPN4
10:90122354	G/A	rs750167470	RNLS
10:91143608	G/A	rs73369750	IFIT1B
10:93220205	G/A		HECTD2
10:93240865	A/G		HECTD2
10:94225474	G/A	rs770839965	IDE
10:94366404	A/G	rs773582910	KIF11
10:94822618	C/T	rs200053509	CYP26C1
10:95072892	G/A	rs199884146	MYOF
10:95085693	C/T	rs565434611	MYOF
10:95092992	G/C	rs202041377	MYOF

10:95159187	C/T	rs373997810	MYOF
10:96014770	C/T	rs180753337	PLCE1
10:96350218	A/G	rs145020994	HELLS
10:96701693	G/A	rs754947384	CYP2C9
10:97174258	G/C	rs370850385	SORBS1
10:97194385	G/A	rs776985431	SORBS1
10:97194411	A/C		SORBS1
10:97194454	C/T	rs747615961	SORBS1
10:97599532	G/A	rs199915134	ENTPD1
10:97816949	C/T	rs201944303	CCNJ
10:98192630	T/C	rs1032846316	TLL2
10:98369513	A/G	rs780606681	PIK3AP1
10:99023341	C/T	rs765513484	ARHGAP19
10:99126613	T/C	rs370734336	RRP12
10:99133424	C/T	rs200477882	RRP12
10:99343346	C/T	rs148239796	ANKRD2
10:99416644	G/A	rs146387356	PI4K2A
10:99639549	G/T		CRTAC1
10:100017794	C/T	rs749714530	LOXL4
10:101451181	G/A	rs767106170	ENTPD7
10:101577180	G/A	rs1216480403	ABCC2
10:101578577	C/T	rs56199535	ABCC2
10:101601836	T/C	rs758070780	ABCC2
10:101606757	G/A	rs748027575	ABCC2
10:102020007	C/T	rs138748193	CWF19L1
10:102054309	C/T	rs202152796	PKD2L1
10:102089040	G/A	rs757832487	PKD2L1
10:102247821	C/T	rs112170994	SEC31B
10:102766359	C/T	rs779310984	LZTS2
10:103384516	G/C		FBXW4
10:103898992	C/T	rs762119001	PPRC1
10:103916792	A/G	rs144588863	NOLC1
10:104128536	G/A		GBF1
10:104139069	C/T	rs1180758541	GBF1
10:104230621	G/A		MFSD13A
10:104679256	C/T		CNNM2
10:105036979	G/A	rs753003968	INA
10:105108678	T/C	rs990570259	PCGF6
10:105108702	C/T		PCGF6
10:105203075	G/C	rs1242710211	PDCD11
10:105204367	A/G	rs141675532	PDCD11
10:105218258	C/T	rs756899682	CALHM1
10:105238660	G/C	rs945300350	CALHM3
10:105331355	C/T		NEURL1
10:105794415	C/T	rs200313424	COL17A1
10:105806506	C/G	rs73329731	COL17A1
10:105812836	G/T		COL17A1
10:105816859	C/A	rs200223042	COL17A1
10:105905262	G/T	rs200783624	CFAP43
10:111885674	C/T		ADD3

10:112540786	C/T	rs977645949	RBM20
10:112541062	G/A	rs61735268	RBM20
10:114045898	C/A		TECTB
10:114177679	C/T	rs1437322930	ACSL5
10:114575060	A/G	rs758848681	VTI1A
10:115345563	C/T	rs141433300	HABP2
10:115388741	C/T	rs200149618	NRAP
10:115485256	G/C	rs201040237	CASP7
10:115612529	C/A	rs201913432	DCLRE1A
10:116203853	A/G		ABLIM1
10:118425181	G/A	rs138140121	C10orf82
10:120354368	C/T	rs758565576	PRLHR
10:121203194	G/A	rs143962659	GRK5
10:121207743	G/A		GRK5
10:121564904	T/C	rs141464998	INPP5F
10:121581986	A/T	rs762586446	INPP5F
10:121692595	G/A	rs763678111	SEC23IP
10:122280559	G/T	rs768298628	PLPP4
10:122646274	G/A	rs200394889	WDR11
10:125506342	G/C		CPXM2
10:125514222	G/T	rs142684083	CPXM2
10:125539759	T/C	rs757948905	CPXM2
10:126715683	C/G	rs112433109	CTBP2
10:127424467	A/T	rs149951107	EDRF1
10:127429151	G/A	rs775580765	EDRF1
10:127456240	T/A	rs763333121	MMP21
10:127525377	C/T		BCCIP,DHX32
10:128821490	A/T	rs529958746	DOCK1
10:129242454	G/A	rs756712008	DOCK1
10:129866448	C/T	rs747243013	PTPRE
10:134010418	G/A	rs776731513	DPYSL4
10:134015517	A/G	rs768524774	DPYSL4
10:134041539	C/T	rs908109999	STK32C
10:134912226	G/A	rs114458014	ADGRA1
10:134941871	A/G	rs145500903	ADGRA1
10:134997433	C/T	rs761767238	KNDC1
10:135098676	A/G	rs759921795	TUBGCP2
10:135180423	G/A	rs751581356	ECHS1
10:135197587	T/C	rs371466427	PAOX
10:135350609	G/A	rs759707874	CYP2E1
11:197689	G/T		ODF3
11:206008	G/A	rs144624940	BET1L
11:298674	G/A	rs374350236	IFITM5
11:396865	C/T	rs146366062	PKP3
11:601580	G/A	rs754418056	PHRF1
11:640561	G/C	rs142719624	DRD4
11:679753	G/A	rs368901164	DEAF1
11:725783	G/T		EPS8L2
11:822403	G/C	rs751072211	PNPLA2
11:831250	T/C	rs199501864	CRACR2B

11:836835	T/C	rs1267651644	CD151
11:1309924	T/C	rs746207071	TOLLIP
11:1857117	T/G	rs140608988	SYT8
11:1955879	A/C	rs1019385765	TNNT3
11:1972199	C/T	rs149034207	MRPL23
11:2189756	A/G	rs147569564	TH
11:2334930	C/A	rs758973942	TSPAN32
11:2424344	C/T	rs200813886	TSSC4
11:2441455	C/T	rs761001440	TRPM5
11:3039154	G/A	rs1380410478	CARS1
11:3039708	G/A	rs769328079	CARS1
11:3115549	G/A	rs151056884	OSBPL5
11:3143637	C/T	rs201350460	OSBPL5
11:3150356	C/T	rs374354189	OSBPL5
11:3700809	A/G	rs138664271	NUP98
11:4144435	C/T	rs141634151	RRM1
11:4148038	A/G	rs1288747323	RRM1
11:4154851	T/C	rs145770432	RRM1
11:4790777	C/A	rs572940532	OR51F1
11:4936401	G/A	rs61747513	OR51G2
11:5221540	G/A	rs145497058	OR51V1
11:5364594	T/G		OR51B5
11:5372925	T/C	rs376039787	OR51B5,OR51B6
11:5373255	C/T	rs551699818	OR51B5,OR51B6
11:5510174	A/C	rs1316125182	OR51B5,OR52D1
11:5510175	C/A		OR51B5,OR52D1
11:5510682	G/A	rs147388113	OR51B5,OR52D1
11:5529987	C/T	rs144033515	UBQLN3
11:5530577	T/C		UBQLN3
11:5565996	A/G		OR52H1
11:5663659	A/G	rs779326424	TRIM6-TRIM34,TRIM34
11:5799453	G/T	rs185237396	OR52N5
11:6190890	T/A	rs757467496	OR52B2
11:6239297	G/A	rs780574233	FAM160A2
11:6244359	G/A	rs753512882	FAM160A2
11:6261008	C/T	rs746858122	CNGA4
11:6292416	G/T	rs200700735	CCKBR
11:6292651	T/A	rs776196223	CCKBR
11:6415553	C/A		SMPD1
11:6423343	G/A	rs200508948	APBB1
11:6477399	C/G	rs775651527	TRIM3
11:6561187	C/T	rs866261363	DNHD1
11:6703590	C/T	rs1463015892	MRPL17
11:6913380	T/C	rs1215770201	OR2D2
11:6942647	T/C		OR2D3
11:7618857	G/C		PPFIBP2
11:7669655	C/T	rs753622546	PPFIBP2
11:8122124	G/T		TUB
11:8246233	T/C	rs201019556	LMO1
11:8645990	G/C		TRIM66

11:8947541	G/C	rs1269749121	C11orf16
11:9048998	G/C	rs141854880	SCUBE2
11:9088340	C/T	rs146308663	SCUBE2
11:9878228	T/C		SBF2
11:10615136	C/T	rs373906826	MRVI1
11:10673607	C/T	rs773082494	MRVI1
11:10715093	G/C		MRVI1
11:10800576	A/T	rs750112805	CTR9
11:12023909	C/T		DKK3
11:12241818	G/A	rs144642828	MICAL2
11:12246240	A/G	rs571399686	MICAL2
11:12246348	C/T	rs140261770	MICAL2
11:14666027	C/T	rs373522381	PDE3B
11:14991595	T/G		CALCA
11:15197525	C/T	rs766025835	INSC
11:17428939	G/A	rs748931549	ABCC8
11:17429956	C/T	rs778384865	ABCC8
11:17742453	A/G	rs199992893	MYOD1
11:17742858	G/A	rs755444576	MYOD1
11:18017358	G/A	rs756628530	SERGEF
11:18305359	A/T	rs181682094	HPS5
11:18359795	G/T		GTF2H1
11:18505533	T/C	rs753626745	TSG101
11:18723282	A/G	rs747819934	TMEM86A
11:18738373	G/A	rs200509378	IGSF22
11:21392425	G/A	rs8176788	NELL1
11:27114730	G/T	rs201115771	BBOX1
11:27390168	G/A	rs369897268	LGR4
11:32421499	T/A		WT1
11:33079565	C/T	rs537737874	TCP11L1
11:33628330	G/C		KIAA1549L
11:34978974	G/A	rs1242053132	PDHX
11:35243218	G/C	rs61755294	CD44
11:36512144	T/G		TRAF6
11:36615705	A/T	rs143415103	RAG2
11:40136986	A/T		LRRC4C
11:43904691	G/C	rs190547700	ALKBH3
11:44148476	G/T	rs747302075	EXT2
11:45939277	A/C		PEX16
11:45945718	C/T	rs144896576	LARGE2
11:46701257	C/T	rs749420115	ARHGAP1
11:46880602	T/C	rs377204138	LRP4
11:46896614	G/T		LRP4
11:47269627	C/T	rs202154881	ACP2
11:47446039	A/T		PSMC3
11:47587489	T/C	rs758900381	PTPMT1
11:47602540	A/G		NDUFS3
11:47830038	A/G	rs1433551328	NUP160
11:48328607	C/A		OR4S1
11:55111386	C/T	rs767128231	OR4A16

11:56128384	T/C	rs201063906	OR8J1
11:57004295	G/A	rs142394789	APLNR
11:57512315	G/A	rs756881265	BTBD18
11:57563057	T/G		CTNND1
11:57886145	T/C	rs144935447	OR9I1,OR9Q1
11:57982427	C/T	rs748941826	OR1S1
11:57982559	T/A	rs146969930	OR1S1
11:58125896	T/C		OR5B17
11:58391757	A/G	rs368989433	CNTF
11:58477315	A/G	rs149668211	GLYAT
11:58477508	A/G	rs751031634	GLYAT
11:58979116	C/T	rs747214046	MPEG1
11:59190317	A/T		OR5A2
11:59480795	A/G	rs775264159	OR10V1
11:59559682	G/A	rs201661485	STX3
11:59856240	T/G	rs140124027	MS4A2
11:60702045	C/A		TMEM132A
11:60703555	C/T	rs376802659	TMEM132A
11:60703876	T/C	rs142571833	TMEM132A
11:61109960	G/A	rs200763152	TKFC
11:61253278	G/T	rs145271355	PPP1R32
11:61724860	A/G	rs748685592	BEST1
11:62286814	C/T		AHNAK
11:62288138	G/C	rs376247409	AHNAK
11:62294772	T/C		AHNAK
11:62295398	T/A	rs141102768	AHNAK
11:62299311	C/T	rs200147726	AHNAK
11:62299943	T/A	rs201303995	AHNAK
11:62494115	C/A	rs750825213	HNRNPUL2
11:62594845	T/A	rs148833107	STX5
11:63719724	T/C		NAA40
11:63974861	G/C		FERMT3
11:64003329	C/T	rs998276317	VEGFB
11:64005012	C/T	rs141977627	VEGFB
11:64005842	G/A	rs572862775	VEGFB
11:64037726	C/G	rs139093260	BAD
11:64055914	C/T	rs752422453	GPR137
11:64067125	G/T		KCNK4
11:64329747	T/C		SLC22A11
11:64360957	T/G	rs759664239	SLC22A12
11:64504329	C/T	rs141060165	RASGRP2
11:64521430	C/T	rs200357590	PYGM
11:64522752	T/C	rs114468011	PYGM
11:64668492	G/A	rs200023496	ATG2A
11:64677270	C/T	rs61746812	ATG2A
11:64678532	C/T		ATG2A
11:64880325	T/C	rs1269532861	TM7SF2
11:64883375	C/A		TM7SF2
11:64954711	C/A	rs192099450	CAPN1
11:65123905	G/A	rs145650667	TIGD3

11:65337966	G/T	rs139666819	ZNRD2
11:65347579	G/A		EHBP1L1
11:65414949	C/T	rs199717374	SIPA1
11:65637694	G/A	rs762712612	EFEMP2
11:65653863	C/T		FIBP
11:65735200	G/A	rs537993475	SART1
11:65767547	C/A		EIF1AD
11:66029303	C/A	rs1462677508	KLC2
11:66062517	G/A	rs1245729165	TMEM151A
11:66101085	C/T	rs116695211	RIN1
11:66252690	A/T	rs201297516	DPP3
11:66454610	G/A	rs150607879	SPTBN2
11:66457287	C/T	rs761002092	SPTBN2
11:66461674	C/T	rs201169791	SPTBN2
11:66475184	C/T	rs143155918	SPTBN2
11:66626068	C/T	rs1049799706	LRFN4,PC
11:66807646	C/G		SYT12
11:67075745	C/G		SSH3
11:67077351	C/T	rs764926762	SSH3
11:67187304	A/G	rs1197852193	CARNS1
11:67200898	G/T		RPS6KB2
11:67209534	G/A	rs1454174653	CORO1B
11:67250697	G/A	rs116940576	AIP
11:67400531	A/G	rs774736258	TBX10
11:67410310	A/G		ACY3
11:67433662	C/T	rs145268144	ALDH3B2
11:67817220	C/T	rs528045019	TCIRG1
11:68453084	C/G		GAL
11:68530157	C/T	rs747835344	CPT1A
11:68575086	G/A	rs61731903	CPT1A
11:68580026	C/A	rs147389938	CPT1A
11:68839416	G/A	rs978081349	TPCN2
11:68846052	A/C	rs765306603	TPCN2
11:70208263	C/T	rs149349390	PPFIA1
11:70263125	C/G	rs139067402	CTTN
11:70666630	G/C	rs781982098	SHANK2
11:70666733	G/A	rs532499001	SHANK2
11:71152334	A/G		DHCR7
11:73020936	C/A	rs1459983916	ARHGEF17
11:73021064	T/C	rs763014747	ARHGEF17
11:73076531	G/A	rs141322412	ARHGEF17
11:73076937	G/T	rs774969674	ARHGEF17
11:73106496	G/A	rs150871389	RELT
11:73141788	G/A	rs201410690	FAM168A
11:73988100	T/G	rs781232004	P4HA3
11:74345739	A/C	rs181857010	POLD3
11:74546882	C/T	rs755293599	RNF169
11:74570320	C/G	rs201462193	XRRA1
11:74716515	C/T	rs201872634	NEU3
11:74876889	C/T	rs148248368	SLCO2B1

11:75282988	C/T	rs764873155	SERPINH1
11:75428971	G/A	rs34643721	MOGAT2
11:75590978	C/G	rs147461545	UVRAG
11:76804776	G/A	rs144453005	CAPN5
11:76825430	C/T	rs1402261316	CAPN5
11:76829337	C/T	rs375065696	CAPN5
11:76868045	C/T	rs373942326	MYO7A
11:76872031	C/T	rs375745526	MYO7A
11:76891451	G/A	rs1052032	MYO7A
11:76944196	G/C	rs371398727	GDPD4
11:77378374	G/A	rs185077706	RSF1
11:77553595	G/C	rs138381967	AAMDC
11:77907879	C/A		USP35
11:77938020	C/A	rs564703513	GAB2
11:78369300	G/A	rs570712669	TENM4
11:78381481	C/T	rs199958891	TENM4
11:78387274	C/T	rs202020330	TENM4
11:78433837	C/T	rs776760211	TENM4
11:82443973	G/C	rs768388699	FAM181B
11:82958985	G/T	rs777721754	ANKRD42
11:85396881	T/C	rs200777690	CCDC89
11:85610055	A/C	rs375388919	CCDC83
11:86662968	C/T	rs148970041	FZD4
11:89935662	T/C	rs758956499	CHORDC1
11:92088564	G/A	rs1444984775	FAT3
11:92258022	C/A		FAT3
11:92523207	G/T	rs201768802	FAT3
11:92577277	G/C		FAT3
11:92714627	G/T	rs772230006	MTNR1B
11:93103266	G/C	rs530435017	DEUP1
11:93430626	C/T	rs116581016	CEP295
11:93494733	G/T		C11orf54
11:93808384	A/G	rs375239799	HEPHL1
11:94180444	C/A	rs376555330	MRE11
11:94341770	G/A	rs140154259	PIWIL4
11:94533347	G/A	rs200480462	AMOTL1
11:94592780	G/A	rs200136415	AMOTL1
11:94906505	C/T	rs139859559	SESN3
11:95826389	G/A	rs201372993	MAML2
11:100999481	G/C	rs147932536	PGR
11:101834514	A/C	rs762199613	CEP126
11:102592236	A/G	rs1395236382	MMP8
11:102649399	T/C	rs371872352	MMP10
11:102710890	G/C		MMP3
11:102815039	G/A	rs142064825	MMP13
11:102824911	T/A		MMP13
11:103025296	G/T	rs201043335	DYNC2H1
11:104763167	A/G		
11:105774670	G/C	rs770665730	GRIA4
11:107375937	C/T	rs532327456	ALKBH8

11:108218039	T/C	rs1591274020	ATM,C11orf65
11:108562630	C/T	rs770527153	DDX10
11:110036098	G/A	rs201741309	ZC3H12C
11:111179155	A/C		COLCA2
11:111593447	C/T	rs748015077	SIK2
11:111613299	G/A	rs566998075	PPP2R1B
11:111711401	C/T	rs782812346	ALG9
11:111845646	C/T	rs782376690	DIXDC1
11:112085561	A/G	rs374509038	BCO2
11:113215004	G/C		TTC12
11:113234602	C/T	rs748308432	TTC12
11:113609036	T/G	rs953805977	ZW10
11:113711324	A/G	rs146991041	USP28
11:114577554	G/T	rs1191928111	NXPE2
11:116692232	G/A	rs374005376	APOA4
11:117062621	G/C	rs368910480	SIDT2
11:117261854	G/A	rs146053426	CEP164
11:117309603	C/T	rs775613586	DSCAML1
11:118110954	C/T	rs145717408	MPZL3
11:118130901	A/G	rs759837097	MPZL2
11:118186226	T/G	rs754411015	CD3E
11:118342648	G/T	rs781870146	KMT2A
11:118344030	G/A	rs1437635121	KMT2A
11:118344650	T/C	rs782261966	KMT2A
11:118534138	A/C		TREH
11:118986834	C/G		C2CD2L
11:119045650	A/T		NLRX1
11:119227996	T/A	rs149898423	USP2
11:120298900	C/T	rs1205771683	ARHGEF12
11:120979932	G/A	rs1364447010	TECTA
11:121037424	G/A	rs770205136	TECTA
11:121174182	G/A	rs927217561	SC5D
11:122945461	C/T	rs528513453	CLMP
11:123900748	G/A	rs79870106	OR10G8
11:123900793	C/G	rs993590258	OR10G8
11:123909479	G/C	rs751096390	OR10G7
11:124440848	C/G	rs150551412	OR8A1
11:124619747	G/A	rs76654665	VSIG2
11:124756674	G/A	rs201688207	ROBO4
11:125765566	C/T	rs200876642	HYLS1,PUS3
11:126147003	A/G	rs7116126	FOXRED1
11:126215285	G/A	rs140377449	DCPS
11:128781827	G/A	rs775915273	KCNJ5
11:128839874	G/A	rs372360136	ARHGAP32
11:129746747	C/T	rs777087266	NFRKB
11:129793184	G/A	rs763250042	PRDM10
11:129979401	G/A	rs761739902	APLP2
11:130007175	C/G		APLP2
11:130067807	G/A	rs745940432	ST14
11:130278387	T/C	rs771991807	ADAMTS8

11:130319205	T/C		ADAMTS15
11:130319647	A/G	rs368298830	ADAMTS15
11:134151947	G/A	rs375343960	GLB1L3
12:304462	T/C	rs1181888178	SLC6A12
12:309921	T/C	rs143648821	SLC6A12
12:311932	G/A	rs751979961	SLC6A12
12:333238	G/A	rs201579126	SLC6A13
12:416243	T/C	rs200449935	KDM5A
12:417051	G/A	rs1450961281	KDM5A
12:1036329	C/T		RAD52
12:1036390	C/T	rs201623936	RAD52
12:1963174	G/A	rs181994120	CACNA2D4
12:2022217	A/G	rs1055255405	CACNA2D4
12:2074725	T/C	rs1013656314	DCP1B
12:2794993	C/T	rs185788586	CACNA1C
12:2800220	A/G	rs201090446	CACNA1C
12:2968448	G/C		FOXM1
12:2973517	C/T	rs147362692	FOXM1
12:2973891	G/A	rs146299415	FOXM1
12:3757762	G/A	rs372821272	CRACR2A
12:4481825	C/T	rs368756067	FGF23
12:5155103	G/A	rs201342234	KCNA5
12:6140654	T/C		VWF
12:6342605	G/A	rs756350694	CD9
12:6463666	T/C	rs758087809	SCNN1A
12:6464545	C/T	rs768438286	SCNN1A
12:6472803	A/G	rs781587925	SCNN1A
12:6623492	C/T	rs202035339	NCAPD2
12:6672928	C/G	rs115447939	NOP2
12:6928020	C/T	rs138218083	CD4
12:7053647	G/A	rs142743155	C12orf57
12:7066829	C/A	rs1177506427	PTPN6
12:7086397	G/A		LPCAT3
12:7260930	C/G	rs201343174	C1RL
12:7351654	C/G	rs751043763	PEX5
12:7354385	C/T	rs144897942	PEX5
12:8200673	C/A		FOXJ2
12:9222385	G/A	rs181129451	A2M
12:9225443	G/C		A2M
12:9305890	A/G	rs778715144	PZP
12:10339078	G/A	rs1357251298	TMEM52B
12:12291316	G/C		LRP6
12:12300323	C/T	rs773594790	LRP6
12:12311775	T/C		LRP6
12:12334197	G/A	rs752144608	LRP6
12:12814551	A/T		GPR19
12:12975665	A/C	rs201398006	DDX47
12:14578192	A/T	rs774003803	ATF7IP
12:14766128	G/A	rs140551603	GUCY2C
12:14774090	G/A	rs144618542	GUCY2C

12:15131004	C/T	rs753392540	PDE6H
12:19427826	G/T		PLEKHA5
12:21453401	A/G		SLCO1A2
12:21608068	A/G	rs1453457664	PYROXD1
12:21639513	G/A	rs150306543	RECQL
12:21711222	G/A	rs776104371	GYS2
12:22637800	G/A	rs748345869	C2CD5
12:27840366	A/T		PPFIBP1
12:29423410	G/A	rs141985647	FAR2
12:31236988	G/A	rs369920569	DDX11
12:31244693	C/T	rs754864339	DDX11
12:31249855	C/T	rs12322122	DDX11
12:32861094	C/T	rs201929226	DNM1L
12:32949101	G/T	rs139734328	PKP2
12:39688263	T/C		KIF21A
12:39701509	C/T		KIF21A
12:40224008	C/T	rs1266079792	SLC2A13
12:40677795	T/C		LRRK2
12:41967174	T/A		PDZRN4
12:42853891	G/A	rs138452760	PRICKLE1
12:42860068	C/T	rs375197568	PRICKLE1
12:42864058	T/C	rs1402955748	PRICKLE1
12:44166753	A/G	rs141209982	IRAK4
12:45173738	G/A	rs778999144	NELL2
12:46320332	C/A	rs759460372	SCAF11
12:46764322	A/G	rs776764111	SLC38A2
12:48141598	T/C	rs146484121	RAPGEF3
12:48380926	G/A	rs140985224	COL2A1
12:48534521	T/C	rs925957626	PFKM
12:48578033	T/C	rs201128716	CCDC184
12:48737049	G/A	rs200502528	ZNF641
12:49221408	C/T	rs151098960	CACNB3
12:49424490	G/A		KMT2D
12:49722810	G/A	rs139479639	TROAP
12:49726930	G/T	rs143197428	C1QL4
12:49878411	G/A	rs61733031	SPATS2
12:50048722	G/A	rs562065768	FMNL3
12:50498475	A/G	rs2232202	GPD1
12:50535840	C/A	rs146787766	CERS5
12:51108272	C/T	rs369371989	DIP2B
12:51584320	C/T		POU6F1
12:52056870	C/T	rs763078635	SCN8A
12:52448216	T/C	rs757668700	NR4A1
12:52627270	C/T	rs138391469	KRT7
12:52714783	C/G	rs777091313	KRT83
12:52844400	C/T	rs71453293	KRT6B
12:52864390	C/T		KRT6C
12:52883773	C/T	rs371739150	KRT6A
12:52883807	G/A	rs368272363	KRT6A
12:52884952	C/T	rs370032292	KRT6A

12:52943819	T/C	rs144642438	KRT71
12:53040719	G/C	rs779620526	KRT2
12:53097083	C/A	rs111504070	KRT77
12:53164964	G/A	rs375239846	KRT76
12:53216981	G/A	rs777338487	KRT79
12:53293648	C/T		KRT8
12:53344641	T/G	rs770760404	KRT18
12:53448166	C/T	rs373949417	TNS2
12:53452590	C/G	rs201486450	TNS2
12:53514632	G/A	rs143680647	SOAT2
12:53676084	C/T	rs757464917	ESPL1
12:53709210	A/G	rs201692749	AAAS
12:53777375	A/C	rs368642435	SP1
12:54367468	C/T	rs773152809	HOXC11
12:54448838	A/T		HOXC4
12:54756641	T/C	rs150704324	GPR84
12:54912710	A/G		NCKAP1L
12:55863885	A/G	rs750858025	OR6C70
12:56096852	A/G		ITGA7
12:56231659	G/A	rs138407292	MMP19
12:56332977	A/C		DGKA
12:56494932	T/C	rs77822103	ERBB3
12:56530568	C/T	rs369273609	ESYT1
12:56549308	G/A	rs778320072	MYL6B
12:56639332	C/T	rs768397824	ANKRD52
12:56642599	C/T	rs201680602	ANKRD52
12:56646022	T/C	rs760754415	ANKRD52
12:57431732	G/C	rs151187460	MYO1A
12:57554788	G/A	rs761803407	LRP1
12:57637600	T/A	rs779483995	STAC3
12:58008563	A/T		ARHGEF25
12:58019236	C/T	rs142736906	SLC26A10
12:58128437	C/T	rs774049850	AGAP2
12:58162893	C/G		METTL1
12:58350480	G/A	rs745670998	ATP23
12:58350629	C/A	rs758248049	ATP23
12:59274659	G/A	rs762474881	LRIG3
12:60169157	A/G	rs143304922	SLC16A7
12:69233268	A/G		MDM2
12:69983377	G/C	rs766622778	CCT2
12:70088218	C/T	rs371263106	BEST3
12:70352261	G/C	rs754427351	MYRFL
12:70953216	G/A	rs541645292	PTPRB
12:71139787	G/A	rs768387199	PTPRR
12:71148080	T/C	rs141302586	PTPRR
12:71977585	A/T	rs751024300	LGR5
12:72013755	G/T	rs372412155	ZFC3H1
12:72026017	T/A	rs774453793	ZFC3H1
12:72050734	G/T	rs561382640	ZFC3H1
12:72680657	C/T	rs201997503	TRHDE

12:75436149	A/T	rs1306301170	KCNC2
12:75445001	C/G	rs752685936	KCNC2
12:75741488	C/G		CAPS2,GLIPR1L1
12:78334201	A/G	rs528385235	NAV3
12:81471942	G/T		ACSS3
12:81777896	C/A	rs762301756	PPFIA2
12:88478367	C/G	rs765514039	CEP290
12:88490716	T/C	rs1400211369	CEP290
12:88512307	T/A	rs369231584	CEP290
12:88513997	A/C		CEP290
12:88520195	A/T	rs774072453	CEP290
12:89861419	A/C	rs765377188	POC1B
12:93139350	T/G		PLEKHG7
12:93251098	C/G	rs150091509	EEA1
12:94697631	T/G	rs773809246	PLXNC1
12:94975936	T/C	rs755958512	TMCC3
12:94975986	T/C	rs530422400	TMCC3
12:95668657	G/A	rs931435629	VEZT
12:95926858	A/G		USP44
12:96356191	G/A	rs144167274	AMDHD1
12:96379705	G/A	rs757249428	HAL
12:99052989	C/T		APAF1
12:100691883	C/T	rs1301290989	SCYL2
12:100934590	A/C	rs149287629	NR1H4
12:101576622	G/A	rs773903392	SLC5A8
12:102155447	T/C		GNPTAB
12:102158531	C/G	rs146460663	GNPTAB
12:102492923	C/T	rs1594394163	NUP37
12:102572410	G/T	rs148483056	PARPBP
12:103245479	C/A	rs5030853	PAH
12:103260383	T/C	rs77554925	PAH
12:104100681	G/C		STAB2
12:104341099	A/G	rs755787354	HSP90B1
12:104697833	C/T	rs377416878	EID3,TXNRD1
12:105428124	C/T	rs140127163	ALDH1L2
12:105538167	A/G		WASHC4
12:105583842	C/T	rs562507472	APPL2
12:105629780	A/C	rs1359650077	APPL2
12:106460681	G/A	rs151011578	NUAK1
12:106460792	G/A	rs777676118	NUAK1
12:106461466	G/A	rs139881229	NUAK1
12:107105260	C/T	rs76703390	RFX4
12:108008884	G/A	rs140496495	BTBD11
12:108086841	C/G	rs780703367	PWP1
12:108913073	T/C	rs148414018	FICD
12:108985672	T/A	rs781222040	TMEM119
12:109637223	G/A	rs1009799722	ACACB
12:109675096	G/A	rs772898022	ACACB
12:109703305	G/A	rs144520667	ACACB
12:109849714	C/T	rs183967344	MYO1H

12:109921415	G/A	rs147402736	UBE3B
12:109928881	G/A	rs747230707	UBE3B
12:110226500	G/A	rs35058636	TRPV4
12:110352346	C/A	rs769910620	TCHP
12:110832983	A/T		ANAPC7
12:111089031	T/G		HVCN1
12:111779599	G/A	rs761841742	CUX2
12:112153654	G/A	rs747866176	ACAD10
12:112321424	A/G	rs755904783	MAPKAPK5
12:112530920	T/G		NAA25
12:113346431	C/G	rs150636851	OAS1
12:113398971	G/C		OAS3
12:113445565	T/C	rs747650665	OAS2
12:113590684	T/A	rs116586136	CFAP73
12:113593218	G/C	rs1026141733	CFAP73
12:113596869	C/T	rs143647024	CFAP73,DDX54
12:113603685	G/A	rs149566389	DDX54
12:113617106	C/T	rs754693454	DDX54
12:113812768	G/A	rs141234639	PLBD2
12:113836621	G/A	rs541907463	SDS
12:114377796	C/T	rs201979395	RBM19
12:114377860	G/A	rs767542752	RBM19
12:115109704	T/C	rs771934403	TBX3
12:117014105	G/A	rs200364642	MAP1LC3B2
12:117465906	C/T	rs191628514	FBXW8
12:117962948	C/T	rs563175641	KSR2
12:118673416	A/C	rs142649312	TAOK3
12:119559885	C/T	rs768328926	SRRM4
12:120152131	G/A	rs138439879	CIT
12:120750369	G/A	rs141365005	SIRT4
12:121613205	A/G	rs1485269129	P2RX7
12:121648031	C/T	rs139621763	P2RX4
12:121648092	A/G		P2RX4
12:121682974	G/A	rs201470961	CAMKK2
12:121747597	C/T	rs138614006	ANAPC5
12:121854065	G/C	rs1479168907	RNF34
12:121877753	T/C	rs369462105	KDM2B
12:122208848	C/T	rs376403880	TMEM120B
12:122248083	C/T	rs61734124	SETD1B
12:122260683	T/C	rs776301135	SETD1B
12:122261544	G/A	rs553963413	SETD1B
12:122618024	C/T		MLXIP
12:122691772	T/G	rs148644965	B3GNT4
12:122845527	G/T		CLIP1
12:123345858	C/T	rs756583740	HIP1R
12:123444415	G/A	rs1240661251	ABCB9
12:123813289	C/T	rs1020639462	SBNO1
12:124093273	C/T	rs368605233	DDX55
12:124197135	A/G		ATP6V0A2
12:124366250	C/T	rs569807517	DNAH10

12:124399051	C/G	rs143987578	DNAH10
12:124419920	A/T	rs762019033	DNAH10
12:124829484	T/C		NCOR2
12:124831135	G/A	rs750708611	NCOR2
12:124831330	C/T	rs372172146	NCOR2
12:124835217	G/A	rs202111746	NCOR2
12:125449019	G/A	rs773733354	DHX37
12:129181906	C/G	rs201749493	TMEM132C
12:129285423	T/A	rs1452110865	SLC15A4
12:129308244	A/C		SLC15A4
12:129559447	G/A	rs201186278	TMEM132D
12:130649096	C/T	rs147350994	FZD10
12:130649097	G/T	rs138051070	FZD10
12:131297541	T/C	rs145834567	STX2
12:132466108	G/A	rs200663976	EP400
12:132505795	C/T	rs1414107776	EP400
12:132625524	G/A		DDX51
12:132636175	G/A	rs148535195	NOC4L
12:132862995	A/G		GALNT9
12:133245077	G/C		POLE
12:133373178	G/A	rs763910236	GOLGA3
12:133378426	T/C	rs145557212	GOLGA3
12:133419664	C/T	rs761464761	CHFR
12:133779756	C/T		ZNF268
13:20012245	A/G		TPTE2
13:21166491	C/T	rs9315740	IFT88
13:21732171	A/G		SKA3
13:21957119	C/T	rs773810335	ZDHHC20
13:23869590	C/T		SGCG
13:23912241	A/T		SACS
13:25075849	G/T	rs979497146	PARP4
13:25425637	G/T		RNF17
13:25478104	T/C	rs141844033	CENPJ
13:25481327	T/C	rs745991478	CENPJ
13:26621187	A/C		SHISA2
13:28498834	G/A	rs754817283	PDX1
13:28592666	T/G		FLT3
13:32953604	G/A	rs59004709	BRCA2
13:32954181	G/A	rs80359171	BRCA2
13:33634837	T/G		KL
13:33686997	C/T		STARD13
13:35619131	G/C		NBEA
13:36049272	A/G		MAB21L1,NBEA
13:36180705	T/C		NBEA
13:37014255	A/G	rs61755282	CCNA1
13:37453435	T/C	rs569499054	SMAD9
13:38161028	T/A		POSTN
13:38320219	A/C		TRPC4
13:39263319	G/A	rs145673360	FREM2
13:39266048	C/T	rs200817424	FREM2

13:39266591	C/A		FREM2
13:39454658	C/T	rs375203975	FREM2
13:39586303	G/T		PROSER1
13:39596501	G/T	rs747366923	PROSER1
13:41827141	T/G		MTRF1
13:41929296	A/G	rs17062054	NAA16
13:41947811	G/A	rs146611323	NAA16
13:43788178	G/A	rs374338452	ENOX1
13:46093140	G/A	rs759431041	COG3
13:46820681	G/A		LRRC63
13:47243248	C/T		LRCH1
13:48942706	G/C	rs1270702085	RB1
13:48953748	C/T	rs759079385	RB1
13:49084865	C/T	rs757285271	RCBTB2
13:49884993	T/C	rs144245337	CAB39L
13:50276526	T/G	rs148675861	KPNA3
13:51397585	G/T		DLEU7
13:51922399	T/A		SERPINE3
13:52534396	T/C	rs767305042	ATP7B
13:52549130	T/C	rs200642204	ATP7B
13:52661583	C/T	rs140291042	NEK5
13:53035193	G/T	rs776001339	CKAP2
13:60384942	C/T	rs775919583	DIAPH3
13:67800580	C/T	rs114529152	PCDH9
13:73320668	G/T		BORA
13:73396092	C/T	rs780265946	PIBF1
13:75880602	T/C	rs779935663	TBC1D4
13:76427520	A/G	rs149304325	LMO7
13:76429413	G/A	rs144929980	LMO7
13:78214897	G/A	rs1411924954	SCEL
13:78327455	A/C	rs148135081	SLAIN1
13:79190746	A/C	rs372028712	OBI1
13:86369589	A/G	rs199516562	SLITRK6
13:88327809	G/A	rs769342000	SLITRK5
13:92101123	C/T	rs142381235	GPC5
13:94958244	G/C	rs748637331	GPC6
13:95860160	T/C		ABCC4
13:96242060	G/A	rs139553238	DZIP1
13:99037976	G/C		FARP1
13:99582438	C/A	rs1263812694	DOCK9
13:99896198	A/G	rs1454490124	UBAC2
13:100617707	G/C	rs774228538	ZIC5
13:101710303	G/A	rs143587652	NALCN
13:103338660	C/G	rs1017895711	METTL21C
13:103513951	A/G	rs4150313	BIVM-ERCC5,ERCC5
13:103528246	A/C	rs201911663	BIVM-ERCC5,ERCC5
13:103701758	G/A	rs146712120	SLC10A2
13:109445884	C/T	rs200186426	MYO16
13:109550364	G/A	rs749977576	MYO16
13:110436449	T/C	rs779239430	IRS2

13:111287067	C/T	rs61734918	NAXD
13:111287888	C/T	rs149297756	NAXD
13:111340076	G/A	rs142034206	CARS2
13:111358316	C/G	rs779385700	CARS2
13:113530221	G/A	rs199958128	ATP11A
13:113980289	G/A	rs141702619	GRTP1
13:114083338	C/G	rs754691717	ADPRHL1
13:114306021	T/C	rs773125482	ATP4B
13:114312413	T/G		ATP4B
13:114782736	T/C	rs562057137	RASA3
14:20215983	T/C		OR4Q3
14:20482590	A/G	rs143121379	OR4K14
14:20711837	A/G		OR11H4
14:20854625	C/T	rs141172994	TEP1
14:20859251	G/A	rs183258806	TEP1
14:20897690	C/G		KLHL33
14:20897736	T/C	rs1349984430	KLHL33
14:21109481	G/A	rs755506112	OR6S1
14:21785941	T/G	rs1486475994	RPGRIP1
14:21871654	A/G	rs1205883889	CHD8
14:21875110	G/A	rs763248373	CHD8
14:23312604	G/A	rs746743029	MMP14
14:23374579	C/T	rs368778639	RBM23
14:23518454	C/T	rs373998017	CDH24
14:23844853	C/T	rs145160878	IL25
14:23858161	C/T	rs533942127	MYH6
14:24041061	C/T	rs201428906	JPH4
14:24551978	G/A	rs1171082162	NRL
14:24573156	C/T	rs201279659	PCK2
14:24613441	C/T	rs138061159	PSME2
14:24614607	G/A		PSME2
14:24619607	A/C	rs753700667	RNF31
14:24653565	A/C	rs768898627	IPO4
14:24655515	G/A	rs367756147	IPO4
14:24684843	C/T	rs760111939	MDP1,NEDD8-MDP1
14:24707865	G/A	rs1163474530	GMPR2
14:24727499	G/A	rs770335603	TGM1
14:24760762	C/T	rs751056286	DHRS1
14:24774224	C/T	rs201242778	NOP9
14:24775626	G/C	rs148872765	CIDEB,NOP9
14:24788961	G/T		ADCY4
14:24791910	C/T	rs138793347	ADCY4
14:24807635	C/G	rs200520478	RIPK3
14:24897992	T/C	rs754765912	CBLN3
14:24898149	G/T		CBLN3
14:24906421	T/C		KHNYN
14:26918084	C/T	rs1177771728	NOVA1
14:30103628	G/A	rs763394987	PRKD1
14:31828100	C/A	rs1482061568	HEATR5A
14:31856439	C/T	rs373714250	HEATR5A

14:31917340	A/T	rs145706913	DTD2
14:33015147	G/T	rs145516811	AKAP6
14:33290888	G/C	rs755618807	AKAP6
14:35593198	C/G	rs753425011	PRORP
14:35872949	G/A	rs779105881	NFKBIA
14:37135732	T/C	rs200733091	PAX9
14:37149866	C/A	rs17104991	SLC25A21
14:38061795	T/C	rs200438155	FOXA1
14:39583663	T/C		GEMIN2
14:39627595	T/A	rs147625412	TRAPPC6B
14:47120525	G/C		RPL10L
14:51221517	T/C	rs750540027	NIN
14:52521026	C/T		NID2
14:52793984	G/A		PTGER2
14:53173926	C/T	rs775248842	PSMC6
14:53360135	G/T	rs199890407	FERMT2
14:54418736	G/T		BMP4
14:54878228	G/C		CDKN3
14:58047982	C/G	rs763647152	SLC35F4
14:59789830	G/T		DAAM1
14:59835392	C/T	rs138713289	DAAM1
14:59939715	C/G		L3HYPDH
14:59950812	T/G	rs775961112	L3HYPDH
14:60582075	G/A	rs140058457	PCNX4
14:60619742	C/T	rs138251248	DHRS7
14:61441898	G/A	rs147405788	TRMT5
14:63246495	G/C	rs143775193	KCNH5
14:63749908	G/A	rs150345688	RHOJ
14:63856444	C/T	rs1446463144	PPP2R5E
14:64457163	T/C	rs755334815	SYNE2
14:65009146	C/T	rs200575249	HSPA2
14:65239642	G/A	rs149727354	SPTB
14:65261324	C/A	rs1280642082	SPTB
14:67787063	C/T	rs750977591	MPP5
14:67854988	A/C		PLEK2
14:68040041	A/G		PLEKHH1
14:68040597	C/T	rs768667777	PLEKHH1
14:68159256	T/A	rs767984929	RDH11
14:69520935	C/T		DCAF5
14:69676192	C/G	rs1459222497	EXD2
14:69676275	C/T	rs1218590339	EXD2
14:69676276	G/C		EXD2
14:70237972	C/T	rs1332582034	SRSF5
14:70634076	C/A	rs141396102	SLC8A3
14:70634632	T/G		SLC8A3
14:72176044	G/A	rs771656202	SIPA1L1
14:73733229	G/T	rs535095570	PAPLN
14:73969624	C/T	rs201974868	HEATR4
14:74042027	G/A	rs949000246	ACOT2
14:74407674	G/T	rs781379472	FAM161B

14:74416817	C/T	rs754225202	COQ6,FAM161B
14:74433689	G/A	rs149673221	ENTPD5
14:74757031	C/G	rs749042883	ABCD4
14:74951269	T/C	rs142075589	NPC2
14:74969614	C/T	rs137854860	LTBP2
14:74975944	C/T	rs748396734	LTBP2
14:75330326	A/G	rs370728105	PROX2
14:75513721	G/C	rs201453923	MLH3
14:76964665	C/T	rs201714970	ESRRB
14:77712312	A/G	rs1325869098	TMEM63C
14:77734810	G/A	rs773475357	NGB
14:77942425	C/T	rs201403223	ISM2
14:78021744	C/T	rs267607090	SPTLC2
14:78182188	T/A	rs199752740	SLIRP
14:78285427	G/A	rs1349175856	ADCK1
14:80327762	C/T	rs183389300	NRXN3
14:80677805	A/T	rs200390046	DIO2
14:81609724	C/T		TSHR
14:81972577	C/T	rs751669409	SEL1L
14:88429849	G/A	rs771203702	GALC
14:88651898	A/G		KCNK10
14:88707116	A/G	rs764070909	KCNK10
14:89087485	G/A	rs373510031	EML5
14:89124636	C/T	rs755046095	EML5
14:89878808	T/C	rs747046500	FOXN3
14:90485680	C/T		TDP1
14:91007716	A/G	rs775950890	TTC7B
14:91413836	C/G	rs7143758	RPS6KA5
14:91739666	C/T	rs201814255	CCDC88C
14:92258809	T/C	rs974985910	TC2N
14:92920379	G/A	rs540116512	SLC24A4
14:93125521	C/G	rs138388973	RIN3
14:93178279	T/G	rs143914416	LGMN
14:93399041	C/T	rs776249486	CHGA
14:93581489	C/T	rs889580600	ITPK1
14:93652981	A/G	rs777915505	TMEM251
14:94046715	G/T	rs1043995250	UNC79
14:94545454	A/C	rs1465318335	DDX24
14:94568276	G/A	rs200925095	IFI27L1
14:94700068	A/C		PPP4R4
14:94845836	C/T	rs367797069	SERPINA1
14:94953823	G/C	rs146053420	SERPINA12
14:95053923	G/A	rs148540452	SERPINA5
14:95670308	C/A	rs376091131	CLMN
14:96707523	C/A		BDKRB2
14:96771963	C/T		ATG2B
14:96797734	G/A		ATG2B
14:96944955	T/C	rs756925348	AK7
14:99640841	G/A		BCL11B
14:99640975	C/T		BCL11B

14:100808759	G/T		WARS1
14:101348671	A/G	rs1282933152	RTL1
14:102674947	T/C		WDR20
14:102717291	G/A	rs151048366	MOK
14:103396640	T/C	rs1232168405	AMN
14:103438476	C/T	rs36001612	CDC42BPB
14:104646009	C/T	rs868085492	KIF26A
14:105190736	C/G	rs1470520650	ADSS1
14:105219581	C/G	rs747059832	SIVA1
14:105268731	C/A	rs1369868185	ZBTB42
14:105349374	C/T	rs559167651	CEP170B
14:105354135	G/A	rs768254237	CEP170B
14:105355876	C/T	rs948318202	CEP170B
14:105395259	C/T	rs745985057	PLD4
14:105411841	G/A	rs185803383	AHNAK2
14:105412015	G/A	rs370304219	AHNAK2
14:105692460	G/C		BRF1
14:105752675	C/T	rs144673138	BRF1
15:28211841	A/G	rs34141095	OCA2
15:28228529	T/C	rs121918170	OCA2
15:28228543	A/T		OCA2
15:28259941	T/C	rs142931246	OCA2
15:28459824	G/A	rs542163867	HERC2
15:28465753	T/C	rs1596266264	HERC2
15:29367193	A/T	rs759790166	APBA2
15:30033639	T/A	rs773283834	TJP1
15:30659691	G/A	rs533316492	CHRFAM7A
15:30927362	G/A	rs143787832	ARHGAP11B
15:31210410	A/G	rs759425884	FAN1
15:31318438	T/C	rs776483887	TRPM1
15:31323297	G/A	rs747440205	TRPM1
15:31355369	C/T	rs751732321	TRPM1
15:32460555	C/T	rs1170628768	CHRNA7
15:32908451	G/T	rs139475473	ARHGAP11A
15:32921841	G/A	rs367930258	ARHGAP11A
15:32925263	G/C		ARHGAP11A
15:32926168	A/G	rs143915480	ARHGAP11A
15:32929483	C/T	rs201625863	ARHGAP11A
15:32976764	A/G	rs1265762656	SCG5
15:32976841	G/C	rs201696326	SCG5
15:34016300	G/A	rs41279210	RYR3
15:34655879	G/T	rs564033666	LPCAT4
15:34825051	G/T	rs202243985	GOLGA8B
15:35152275	G/A	rs201619100	AQR
15:35202418	T/C	rs773309612	AQR
15:35252989	C/G		AQR
15:35674068	T/C		DPH6
15:38756338	A/G	rs200908921	FAM98B
15:38800010	A/G	rs750396457	RASGRP1
15:39885643	T/C		THBS1

15:40057794	T/C	rs779075315	FSIP1
15:40268615	G/A		EIF2AK4
15:40457337	C/T	rs56079734	BUB1B
15:40587472	A/G	rs770325829	PLCB2
15:40764208	T/C	rs377155775	CHST14
15:40865748	G/A	rs149164635	RPUSD2
15:40915075	T/G	rs144939300	KNL1
15:41030724	C/T	rs772297262	RMDN3
15:41146651	G/A	rs780369905	SPINT1
15:41192187	G/A	rs202222195	VPS18
15:41194817	G/A	rs149373237	VPS18
15:41195144	C/T	rs765703231	VPS18
15:41272426	G/A	rs151117314	INO80
15:41275242	C/G	rs141707556	INO80
15:41319846	C/T	rs751912347	INO80
15:41812920	C/T	rs374674439	RPAP1
15:41822151	T/G		RPAP1
15:41865248	G/T		TYRO3
15:41988302	C/G	rs1443024304	MGA
15:42003413	C/T	rs372122562	MGA
15:42034954	C/T	rs377760732	MGA
15:42054491	G/C		MGA
15:42132743	T/G	rs1487227909	JMJD7-PLA2G4B,PLA2G4B
15:42137857	A/G	rs768490358	JMJD7-PLA2G4B,PLA2G4B
15:42149800	T/C	rs759117349	SPTBN5
15:42165746	T/C		SPTBN5
15:42169436	A/G	rs1456204060	SPTBN5
15:42192914	C/T	rs747383299	EHD4
15:42364038	C/T	rs191357380	PLA2G4D
15:42364091	G/C	rs199959919	PLA2G4D
15:42529665	G/A	rs149080105	TMEM87A
15:42631950	C/T	rs144134584	GANC
15:42652017	T/C		CAPN3
15:42686537	T/A	rs774834498	CAPN3
15:42858878	G/A	rs777814162	HAUS2
15:43020434	G/A	rs114779238	CDAN1
15:43038189	C/T	rs200469913	TTBK2
15:43299402	G/A	rs142285781	UBR1
15:43426543	A/C	rs780673040	TMEM62
15:43482569	T/C	rs147781453	CCNDBP1
15:43527092	A/G	rs146901531	TGM5
15:43661963	T/C	rs199823856	ZSCAN29
15:43724612	G/A	rs370172450	TP53BP1
15:43815076	C/T		MAP1A
15:43892839	G/C	rs775159378	STRC
15:43905364	C/T		STRC
15:44630076	C/A		GOLM2
15:45391946	C/T	rs368488511	DUOX2
15:45394035	C/T	rs774192350	DUOX2
15:45399552	A/G	rs774076526	DUOX2

15:45400285	G/A	rs750288898	DUOX2
15:45402093	G/A	rs119472029	DUOX2
15:45412325	C/T	rs1009326616	DUOXA1
15:48446057	G/A	rs370931661	MYEF2
15:48512874	G/T		SLC12A1
15:48591372	G/A	rs199880194	SLC12A1
15:48704816	G/A	rs61746008	FBN1
15:49034217	C/T		CEP152
15:49833979	C/T		FAM227B
15:50519308	G/C		SLC27A2
15:50899447	A/T	rs35648842	TRPM7
15:50906346	A/G	rs200922295	TRPM7
15:51676000	G/T	rs371475015	GLDN
15:51829839	G/A	rs145006217	DMXL2
15:51980501	G/A	rs768697386	SCG3
15:52186062	C/G	rs143809392	TMOD3
15:52567833	A/C	rs748879031	MYO5C
15:52689632	T/C	rs769184092	MYO5A
15:53081840	C/T	rs142641519	ONECUT1
15:55722890	T/G	rs148152687	DNAAF4
15:56719803	G/T	rs201973612	TEX9
15:56735701	C/T	rs111585871	MNS1,TEX9
15:57820987	C/T	rs746164326	CGNL1
15:57921967	C/T	rs200330646	GCOM1,MYZAP
15:58004234	C/G	rs756202023	GCOM1,POLR2M
15:59499797	C/T	rs140321095	LDHAL6B,MYO1E
15:59506459	T/C		MYO1E
15:60641386	G/A	rs776962395	ANXA2
15:61521336	C/G	rs749032388	RORA
15:62228934	C/T		VPS13C
15:62237993	G/C		VPS13C
15:62283995	G/A	rs370789719	VPS13C
15:62994316	G/T	rs369897555	TLN2
15:63569907	C/T	rs1421487967	APH1B
15:63632575	C/T	rs747422711	CA12
15:63916429	C/T	rs372316431	HERC1
15:63966847	C/T	rs766836104	HERC1
15:64658266	G/A	rs373526937	PCLAF
15:64710874	G/C	rs113596176	TRIP4
15:64967447	C/G		ZNF609
15:65108865	G/A	rs150356082	PIF1
15:65114700	C/G	rs139868280	PIF1
15:65370213	T/C	rs1215359382	KBTBD13
15:65370385	C/T	rs372826347	KBTBD13
15:65495722	G/C	rs990898542	CILP
15:65555516	G/A	rs146223049	PARP16
15:65703646	C/T	rs142198652	IGDCC4
15:65937989	C/T		SLC24A1
15:66214724	C/T	rs143395466	MEGF11
15:66845438	T/G	rs150346835	LCTL

15:67713703	G/A	rs147731503	IQCH
15:68486418	G/T		CALML4
15:68606124	A/G	rs769606074	ITGA11
15:69728948	C/T	rs748902179	KIF23
15:72338513	C/T	rs755901127	MYO9A
15:72459382	G/A	rs141536149	GRAMD2A
15:72509803	T/C	rs768357296	PKM
15:73528769	C/T	rs142333707	NEO1
15:73614829	T/C	rs768028811	HCN4
15:74426779	A/G		ISLR2
15:74427127	G/C	rs771165593	ISLR2
15:75043542	G/A	rs746644895	CYP1A2
15:75044195	G/A	rs56276455	CYP1A2
15:75500763	G/A	rs768218420	C15orf39
15:75648970	G/A	rs1485593080	MAN2C1
15:75651087	T/C	rs202052221	MAN2C1
15:75932344	G/A		IMP3
15:75982288	G/A	rs749491820	CSPG4
15:76018498	G/A	rs143716172	ODF3L1
15:76019462	C/T	rs112929195	ODF3L1
15:76152307	C/T	rs751740844	UBE2Q2
15:77224726	C/A	rs144252373	RCN2
15:77471648	C/T	rs556459431	PEAK1
15:77471654	G/T	rs200573840	PEAK1
15:77907357	G/C		LINGO1
15:78778154	G/A	rs1160069811	IREB2
15:78786319	C/T	rs147288797	IREB2
15:78893762	G/A	rs79701466	CHRNA3
15:78922149	G/C	rs148540431	CHRNB4
15:79068582	G/A	rs765813251	ADAMTS7
15:81188340	G/C	rs764946392	CEMIP
15:81230224	C/T	rs376164357	CEMIP
15:81271778	C/T	rs769085988	MESD
15:81271801	T/C	rs146044786	MESD
15:81648870	C/T	rs146506274	TMC3
15:85197490	C/G	rs757624021	WDR73
15:86253813	C/T	rs112971613	AKAP13
15:86286936	G/A	rs140260439	AKAP13
15:86312749	A/G	rs756976281	KLHL25
15:86838537	C/T	rs545196930	AGBL1
15:89008054	G/T		MRPL46
15:89074217	A/C		DET1
15:89402048	G/T	rs185800102	ACAN
15:89422320	G/A	rs138635787	HAPLN3
15:89442723	T/A	rs147286834	MFGE8
15:89858541	G/C		FANCI
15:89872328	C/T	rs146603953	POLG
15:89876442	C/T		POLG
15:90030069	T/C	rs142667714	RHCG
15:90176416	G/A	rs146368430	KIF7

15:90227074	C/T	rs146767422	PEX11A
15:90227105	G/A	rs149426857	PEX11A
15:90446471	C/T	rs35321360	ARPIN,ARPIN-AP3S2
15:90934099	C/T	rs745638782	IQGAP1
15:91292995	C/T	rs896357448	BLM
15:91433444	T/C		FES
15:91475157	G/C	rs758657403	HDDC3,UNC45A
15:91504953	G/C	rs145370091	RCCD1
15:91548307	A/G	rs149121639	VPS33B
15:99491900	G/A	rs368518102	IGF1R
15:99696408	C/T	rs1450063029	TTC23
15:100252630	C/G	rs1056672385	MEF2A
16:231012	C/T	rs559899613	HBQ1
16:312134	G/T	rs768332766	FAM234A
16:313713	G/A		FAM234A
16:332810	A/T		ARHGDIG
16:336860	A/T	rs45585539	PDIA2
16:425386	G/A	rs202054688	PGAP6
16:427765	G/A	rs143766173	PGAP6
16:460365	C/T	rs150670530	DECR2
16:597040	G/A	rs144165814	CAPN15
16:601367	T/G	rs971681518	CAPN15
16:601597	C/T	rs201195272	CAPN15
16:601655	A/G		CAPN15
16:624664	A/C		PIGQ
16:628860	C/T	rs780359107	PIGQ
16:683155	G/C	rs569430619	WFIKKN1
16:684632	C/T	rs142689363	METTL26
16:705827	G/C	rs370376692	WDR90
16:706828	C/T	rs747005220	WDR90
16:716948	G/A		WDR90
16:726826	G/A	rs769183668	RHBDL1
16:734744	T/A	rs368939669	WDR24
16:780942	C/T	rs147538611	CIAO3
16:841917	G/A	rs369587642	CHTF18
16:847913	C/T	rs190105039	CHTF18
16:1245485	G/A	rs1159226662	CACNA1H
16:1250386	C/T	rs978379155	CACNA1H
16:1271955	G/A	rs181691054	TPSG1
16:1396205	G/A	rs765382046	BAIAP3
16:1399946	G/C	rs371843849	TSR3
16:1412115	C/T	rs200741370	GNPTG
16:1412648	G/A		GNPTG
16:1413061	G/A	rs561640998	GNPTG
16:1417818	C/T	rs370955849	UNKL
16:1498758	C/T	rs200917404	CLCN7
16:1510904	T/A		CLCN7
16:1576790	C/T	rs538791217	IFT140
16:1604948	G/A	rs772063146	IFT140,TMEM204
16:1604986	C/A		IFT140,TMEM204

16:1706046	G/A	rs772594351	CRAMP1
16:1717989	G/C	rs560160907	CRAMP1
16:1841557	C/T	rs767507229	IGFALS
16:1961882	G/C	rs369204071	HS3ST6
16:2011840	C/T	rs898194487	NDUFB10
16:2028416	A/T	rs138475784	TBL3
16:2070066	T/C	rs573754213	NPW
16:2140180	G/A	rs115538130	PKD1
16:2147374	C/T	rs146507511	PKD1
16:2147895	G/A	rs142799331	PKD1
16:2150232	T/C		PKD1
16:2152849	C/T	rs150189496	PKD1
16:2156479	G/A	rs539143745	PKD1
16:2185509	G/A	rs886038369	PKD1
16:2239274	G/T		CASKIN1
16:2259421	T/A		BRICD5
16:2293167	G/T	rs756800787	ECI1
16:2521923	G/C	rs571696463	NTN3
16:2548252	T/G	rs753105655	TBC1D24
16:3107123	A/T		MMP25
16:3274259	T/G		ZNF200
16:3293424	T/G		MEFV
16:3336067	G/T	rs143903106	ZNF263
16:3406100	C/T	rs758078126	OR2C1
16:3406511	G/T	rs138530420	OR2C1
16:3576466	C/T	rs137950850	CLUAP1
16:3900353	G/A	rs374499169	CREBBP
16:4164353	G/A	rs145790925	ADCY9
16:4387425	C/T	rs762640275	GLIS2
16:4414844	C/G	rs147491703	CORO7,CORO7-PAM16
16:4933644	G/A	rs766950990	PPL
16:4933690	G/A	rs140094738	PPL
16:4935942	C/T	rs773132799	PPL
16:4940228	G/C	rs370362845	PPL
16:5094473	C/T	rs754662565	C16orf89
16:8851653	C/G	rs541365944	ABAT
16:8890113	G/T	rs202170837	TMEM186
16:8953046	C/T	rs558506791	CARHSP1
16:10788468	C/T	rs768273976	TEKT5
16:10850550	G/A	rs138221572	NUBP1
16:10855689	G/A	rs557472106	NUBP1
16:10861830	C/G	rs148922151	NUBP1,TVP23A
16:11000716	T/C	rs764714532	CIITA
16:11001545	G/T		CIITA
16:11001939	C/T	rs766316610	CIITA
16:14355014	G/A	rs116802282	MRTFB
16:14966155	G/A	rs1271003091	NOMO1
16:15100295	G/A	rs138949435	PDXDC1
16:15711211	C/T	rs202216269	MARF1
16:15842009	G/A	rs148893135	MYH11

16:16244054	G/A	rs63750135	ABCC6
16:16251662	G/C	rs200242428	ABCC6
16:16276369	T/C		ABCC6
16:16302637	G/A	rs72653756	ABCC6
16:18558579	C/T	rs1189580197	NOMO2
16:19656225	A/T		VPS35L
16:19726302	T/G	rs765705295	KNOP1
16:20494394	T/G		ACSM2A
16:20636840	G/A	rs905851561	ACSM1
16:20803579	G/T		ACSM3,ERI2
16:20826344	A/C	rs777348643	REXO5
16:20838418	C/T	rs374659309	REXO5
16:20966345	G/C	rs199783094	DNAH3
16:20976433	T/C	rs1291360148	DNAH3
16:20981303	C/G	rs34223805	DNAH3
16:21042416	T/C	rs768162333	DNAH3
16:21270140	C/T	rs727502945	CRYM
16:22144257	G/A	rs200694202	VWA3A
16:23382634	C/G	rs35728064	SCNN1B
16:23505629	A/T	rs138397318	GGA2
16:23717690	G/A	rs34683474	ERN2
16:23718355	G/C	rs56117885	ERN2
16:24887047	A/T		SLC5A11
16:24920302	G/T		SLC5A11
16:24950751	G/C	rs754276172	ARHGAP17
16:25180449	G/T	rs200832194	LCMT1
16:25255403	G/A	rs748887876	ZKSCAN2
16:27374163	A/G		IL4R
16:27509021	G/A	rs374396728	GTF3C1
16:27763189	G/A	rs185801522	KIAA0556
16:28499044	T/C	rs11552531	CLN3
16:28841268	A/G		ATXN2L
16:28993745	G/A	rs769292422	SPNS1
16:29848154	G/C	rs71389430	MVP
16:29857598	T/C		MVP
16:29916256	G/A	rs375732156	ASPHD1
16:29993188	A/G		TAOK2
16:30078630	A/G	rs929389001	ALDOA
16:30078942	C/T	rs776721459	ALDOA
16:30615733	G/T	rs140674594	ZNF689
16:30666420	C/T	rs749734904	PRR14
16:30667198	G/C		PRR14
16:30676368	C/T	rs774452467	FBRS
16:30731483	C/T	rs889307681	SRCAP
16:30732651	C/T	rs773636301	SRCAP
16:30783422	C/T		RNF40
16:30975605	G/A		SETD1A
16:30997430	C/G		HSD3B7
16:31090182	G/T	rs200280714	ZNF646
16:31202284	G/T		FUS

16:31336688	C/T	rs746481763	ITGAM
16:31422470	T/C	rs373529684	ITGAD
16:31477594	C/G	rs200951744	ARMC5
16:31510727	G/C	rs199577128	RUSF1
16:31765204	A/G	rs755450984	ZNF720
16:46633857	C/T	rs1003144200	SHCBP1
16:46652152	T/C	rs945146766	SHCBP1
16:47162475	C/T	rs147090370	NETO2
16:48145497	C/T	rs61742618	ABCC12
16:48220952	G/A	rs761021001	ABCC11
16:48226549	T/G		ABCC11
16:48303972	A/G	rs1185282942	LONP2
16:49670253	C/T		ZNF423
16:49670325	G/A	rs200585917	ZNF423
16:49671925	G/A	rs985841675	ZNF423
16:50659433	G/A	rs141900711	NKD1
16:50733783	T/G	rs867184583	NOD2
16:50744565	T/G	rs104895423	NOD2
16:50745099	G/A	rs562225614	NOD2
16:53190831	C/G	rs771131924	CHD9
16:53265413	G/A	rs200434598	CHD9
16:53698869	T/C	rs137982921	RPGRIP1L
16:55880681	C/T	rs16955812	CES5A
16:55893497	C/T	rs150415850	CES5A
16:56492469	T/A	rs144257152	OGFOD1
16:56660410	T/A	rs138690474	MT1E
16:56717905	T/A	rs748169079	MT1X
16:56875737	C/T	rs773614710	NUP93
16:56920278	C/T	rs140012781	SLC12A3
16:57149401	A/C	rs1180190070	CPNE2
16:57188265	G/C	rs764328745	PSME3IP1
16:57490512	C/T	rs774168768	COQ9
16:57764972	C/T	rs369743078	DRC7
16:57787392	G/A	rs369120357	KATNB1
16:57954420	T/G	rs774536610	CNGB1
16:58001190	T/A	rs745636376	CNGB1
16:66957616	C/A		RRAD
16:67035339	C/T	rs779593858	CES4A
16:67201759	G/A	rs199773890	HSF4
16:67265587	G/A	rs753143941	FHOD1
16:67354596	C/T	rs780423432	KCTD19
16:67697911	G/A	rs373980533	ENKD1
16:67763231	T/A	rs756625027	RANBP10
16:67865928	T/A	rs146813402	CENPT
16:68156589	C/T	rs765421785	NFATC3
16:68265974	G/A	rs755162828	ESRP2
16:68358715	G/A	rs377261290	PRMT7
16:68373705	A/T		PRMT7
16:69294161	C/G	rs771544005	SNTB2
16:69364008	G/C		COG8,PDF

16:69370482	A/G	rs141717174	COG8
16:69375458	C/A		NIP7
16:70219861	C/T	rs144719711	CLEC18C
16:70285694	C/G	rs781217289	EXOSC6
16:70285785	G/A	rs753994231	EXOSC6
16:70316554	G/A	rs761574997	AARS1
16:70316614	A/C		AARS1
16:70508062	C/T	rs776974813	FCSK
16:70698984	C/G	rs765890015	MTSS2
16:70726752	C/G	rs761901472	VAC14
16:70841632	T/C	rs200322457	HYDIN
16:70867931	C/T	rs201554059	HYDIN
16:70977806	C/G	rs746471423	HYDIN
16:71171073	C/G	rs200435836	HYDIN
16:71319198	T/G	rs759362349	CMTR2
16:71898070	G/A	rs1567407725	ZNF821
16:71961517	G/T	rs765193598	IST1
16:72057084	T/G		DHODH
16:72057511	G/A	rs369778205	DHODH
16:72094679	G/T	rs376612221	HP
16:72174339	C/T	rs781341509	PMFBP1
16:72827613	C/T	rs757947521	ZFHX3
16:74670306	T/C	rs200354694	RFWD3
16:75271212	A/C	rs1369280168	BCAR1
16:75564198	T/G	rs200327821	CHST5
16:75654190	A/G	rs550860402	ADAT1
16:75661858	C/G		KARS1
16:76528921	A/C	rs772732975	CNTNAP4
16:76556060	G/T		CNTNAP4
16:76587285	T/G		CNTNAP4
16:78466521	C/T	rs193001955	WWOX
16:80667130	G/C	rs879863061	CDYL2
16:81078018	G/A	rs765101633	ATMIN
16:81298377	C/T	rs776458742	BCO1
16:84063154	G/C	rs780054215	SLC38A8
16:84256083	C/A	rs144899006	KCNG4
16:84256361	G/A	rs772160103	KCNG4
16:84256578	A/G	rs149163346	KCNG4
16:84472851	A/G	rs377115423	ATP2C2
16:84492940	G/A	rs575721893	ATP2C2
16:84513578	C/G	rs146720539	MEAK7
16:84779243	C/A	rs199654727	USP10
16:85141675	G/T	rs755342432	CIBAR2
16:85699948	A/T	rs749926235	GSE1
16:85832832	A/G		EMC8
16:87717846	C/T	rs772957757	JPH3
16:88744891	G/A	rs150467929	SNAI3
16:88773584	G/C	rs189839353	CTU2
16:88779754	G/C	rs142100748	CTU2
16:88779821	C/T	rs776220494	CTU2

16:88782026	C/G	rs774268616	PIEZO1
16:88782069	T/G	rs1209949243	PIEZO1
16:88793146	T/C	rs928741600	PIEZO1
16:88800834	G/A	rs777039661	PIEZO1
16:88801621	G/A	rs146505418	PIEZO1
16:88902134	G/A	rs775300515	GALNS
16:88967965	G/A	rs201395224	CBFA2T3
16:89187245	G/A	rs559765310	ACSF3
16:89258832	C/A	rs774035764	CDH15
16:89345701	C/T	rs1205729029	ANKRD11
16:89347734	C/T	rs757264535	ANKRD11
16:89350393	T/C	rs779145849	ANKRD11
16:89598340	C/G	rs761518974	SPG7
16:89619411	G/A	rs754771891	SPG7
16:89713646	C/T	rs1064794609	CHMP1A
16:89775352	C/T	rs374177055	VPS9D1
16:89985950	C/T	rs34158934	MC1R
16:89986252	T/G	rs3212366	MC1R
16:90030653	C/T	rs138908537	DEF8
17:465891	T/C		VPS53
17:489559	A/C	rs767364217	VPS53
17:644611	G/A	rs201486568	TLCD3A
17:900483	G/A		TIMM22
17:904263	G/T	rs200687812	TIMM22
17:1381398	C/T	rs541831167	MYO1C
17:1442183	G/C	rs753764282	PITPNA
17:2227825	G/A	rs140102145	SRR,TSR1
17:2237828	C/G	rs766223989	TSR1
17:2595749	C/T		CLUH
17:2598730	G/C	rs200277514	CLUH
17:2604519	G/A	rs200260191	CLUH
17:2923825	C/T	rs549183815	RAP1GAP2
17:2934257	C/T	rs201869060	RAP1GAP2
17:3181326	T/C	rs202113738	OR3A2
17:3195336	A/G	rs61734042	OR3A1
17:3366055	G/A	rs1428510570	SPATA22
17:3427506	C/T	rs200894372	TRPV3
17:3486724	G/A	rs775203776	TRPV1
17:3527384	G/A	rs146284695	SHPK
17:3563159	T/C	rs760798633	CTNS
17:3585257	C/G	rs375878631	P2RX5
17:3593389	G/A	rs143709684	P2RX5
17:3655054	C/T	rs200416853	ITGAE
17:3802253	C/T	rs185009352	P2RX1
17:3848369	G/T	rs1005569748	ATP2A3
17:3854883	C/T	rs371209540	ATP2A3
17:3916824	C/T	rs147838442	ZZEF1
17:3980041	G/C		ZZEF1
17:3989005	G/A	rs148159815	ZZEF1
17:4352571	C/A	rs1284187091	SPNS3

17:4439390	G/A	rs371400985	SPNS2
17:4488030	C/T		SMTNL2
17:4574796	C/G	rs199531847	PELP1
17:4799567	G/C		MINK1
17:4835991	T/C	rs201827537	GP1BA
17:4846171	C/G		RNF167
17:4871034	C/G	rs770443620	SPAG7
17:4876253	C/T		CAMTA2
17:4937834	G/T	rs560354267	SLC52A1
17:5073816	C/T		USP6
17:5276637	T/A		RABEP1
17:5290761	G/C	rs557899492	NUP88
17:5317422	C/T	rs150678594	NUP88
17:5347712	G/A		DHX33
17:6531853	A/G	rs748326391	KIAA0753
17:7080613	G/A	rs140607573	ASGR1
17:7125612	G/A	rs866464446	ACADVL
17:7132319	G/C	rs377201716	DVL2
17:7160257	T/C		ELP5
17:7217464	A/T		GPS2
17:7359929	A/G	rs1429690968	CHRNB1
17:7369208	C/T	rs761184760	ZBTB4
17:7385708	A/C		SLC35G6,ZBTB4
17:7605071	C/T	rs149828392	WRAP53
17:7612856	C/T	rs532773147	EFNB3
17:7674231	C/T	rs772727841	DNAH2
17:7684051	G/A	rs775562825	DNAH2
17:7684069	G/A	rs201098580	DNAH2
17:7684386	G/A	rs372409861	DNAH2
17:7697615	G/A	rs772981855	DNAH2
17:7722256	G/A	rs140819153	DNAH2
17:7803295	C/T	rs780573521	CHD3
17:7811230	G/A	rs149185282	CHD3
17:8013773	T/G	rs142781546	ALOXE3
17:8046123	C/T	rs866940939	PER1
17:8046879	T/C	rs767701385	PER1
17:8133945	G/C		CTC1
17:8158983	C/T	rs139148307	PFAS
17:8222410	T/C		ARHGEF15
17:8644974	G/A	rs146567497	CCDC42
17:9489263	G/A	rs150136894	CFAP52
17:9497517	G/A	rs138755738	CFAP52
17:10258407	C/T	rs201854489	MYH13
17:10297592	C/T	rs553069572	MYH8
17:10302965	G/A	rs34953692	MYH8
17:10304470	C/T	rs201992379	MYH8
17:10348375	T/C	rs75173310	MYH4
17:10352033	G/A	rs768165008	MYH4
17:10352274	C/G		MYH4
17:10367998	G/A	rs563253512	MYH4

17:10397924	G/A	rs1039075314	MYH1
17:10398385	G/C	rs956005247	MYH1
17:10408175	C/G	rs145689977	MYH1
17:10419566	G/T	rs201260086	MYH1
17:10426647	C/A	rs769778269	MYH2
17:10432931	C/G	rs201925793	MYH2
17:10538189	C/T	rs201602783	MYH3
17:10541557	C/T	rs199834077	MYH3
17:10542472	C/T	rs142002449	MYH3
17:10543053	C/T	rs747225470	MYH3
17:10554944	C/G		MYH3
17:11461199	C/T	rs909247684	SHISA6
17:11502123	T/G	rs373784591	DNAH9
17:12898100	G/C		ELAC2
17:14110268	C/T	rs148783821	COX10
17:14248785	A/G	rs1379458535	HS3ST3B1
17:15496778	G/A	rs372307584	CDRT1
17:15532104	G/A	rs146156927	TRIM16
17:15976868	C/T	rs139458532	NCOR1
17:15983926	T/C	rs562260255	NCOR1
17:16332186	G/C		TRPV2
17:16351200	C/G		LRRC75A
17:17119807	G/T	rs1447526883	FLCN
17:17909512	T/C	rs375064299	DRC3
17:18024570	C/G	rs767378045	MYO15A
17:18039039	G/T	rs149813580	MYO15A
17:18044120	C/T	rs369109640	MYO15A
17:18051861	C/T	rs199831544	MYO15A
17:18065941	G/A	rs775028334	MYO15A
17:18140990	G/A	rs139750751	LLGL1
17:18181042	G/A	rs373080718	TOP3A
17:18638394	G/A	rs1218725470	TRIM16L
17:18648014	G/T		FBXW10
17:19316338	C/T	rs770500922	RNF112
17:19559755	A/C	rs1049260316	ALDH3A2
17:19566694	A/G	rs147200808	ALDH3A2
17:19642862	C/T	rs1254877247	ALDH3A1
17:20160812	G/A	rs371442923	SPECC1
17:21075428	C/G	rs371261090	DHRS7B
17:25636171	G/C	rs756219097	WSB1
17:26671405	G/T		TNFAIP1
17:26887207	A/G		PIGS
17:26901526	A/G		ALDOC
17:27225704	C/T	rs377069254	DHRS13
17:27286216	A/C		SEZ6
17:27380597	T/C		PIPOX
17:27383216	G/A	rs375503018	PIPOX
17:27613342	T/G		NUFIP2
17:27613390	G/A	rs775632448	NUFIP2
17:27899563	G/A		TP53I13

17:28644214	G/A		TMIGD1
17:28656532	A/G		TMIGD1
17:28791657	G/A		CPD
17:29203442	C/T	rs145425613	ATAD5
17:30200427	C/G	rs759849700	UTP6
17:33503048	G/A	rs768268417	UNC45B
17:33507673	G/A	rs76329788	UNC45B
17:33510482	C/A	rs745343724	UNC45B
17:33904213	A/C		PEX12
17:34854939	G/A	rs966331392	MYO19,ZNHIT3
17:36454530	G/A	rs775042643	MRPL45
17:36483101	C/G		GPR179
17:36491535	G/A	rs1369783617	GPR179
17:37321335	C/G	rs185189243	ARL5C
17:37347786	G/A	rs759986239	CACNB1
17:37604061	C/T	rs141590722	MED1
17:37819132	C/T	rs151032154	STARD3
17:37821971	G/T	rs375310569	TCAP
17:37822175	G/A	rs576098128	TCAP
17:37826059	G/A		PNMT
17:37842254	T/C	rs141817321	PGAP3
17:38100874	T/G	rs527736517	LRRC3C
17:38856297	G/A	rs16966138	KRT24
17:38856535	G/A	rs759487802	KRT24
17:38907216	A/G		KRT25
17:38926029	C/G	rs78137854	KRT26
17:38936014	C/T	rs200226541	KRT27
17:39274212	C/T	rs187464255	KRTAP4-11
17:39505640	T/C	rs145389769	KRT33A
17:39577641	G/A	rs374934469	KRT37
17:39619250	T/G		KRT32
17:39644596	G/A	rs751430605	KRT36
17:39672184	G/A	rs149918613	KRT15
17:39673065	C/T	rs140616866	KRT15
17:39739667	C/T	rs764013329	KRT14
17:39777128	C/T	rs149778356	KRT17
17:39925295	G/T	rs372145644	JUP
17:40010041	C/T	rs781826045	KLHL11
17:40025798	C/T	rs1488337852	ACLY
17:40040459	A/G		ACLY
17:40068731	T/C	rs781834702	ACLY
17:40174540	G/A	rs1555653307	NKIRAS2
17:40263887	C/G	rs200502586	DHX58
17:40441515	A/C	rs1401556871	STAT5A
17:40695415	G/A	rs377282648	NAGLU
17:40735593	G/A	rs765041342	RETREG3
17:40839738	G/T		CNTNAP1
17:40939483	C/G	rs57737815	WNK4
17:40997352	G/A	rs577504737	AOC2
17:40998087	C/T	rs151168110	AOC2

17:41055964	C/T	rs1801175	G6PC
17:41243891	C/G	rs80356876	BRCA1
17:41365077	C/A	rs142986974	TMEM106A
17:41598119	A/G		DHX8
17:41719288	C/T	rs145290029	MEOX1
17:42232260	C/T	rs776309501	HROB
17:42267983	C/G		TMUB2
17:42336541	A/G	rs150403270	SLC4A1
17:42454411	C/T		ITGA2B
17:42458426	A/G	rs75622274	ITGA2B
17:42818710	T/G	rs201168047	DBF4B
17:42941060	G/T	rs377238998	EFTUD2
17:42982213	A/G	rs780071216	
17:42988616	C/T	rs997766080	GFAP
17:42988811	T/G	rs373961432	GFAP
17:43246907	G/C	rs775048819	HEXIM2
17:43322662	G/A	rs753028987	FMNL1
17:43515255	C/T	rs148339081	PLEKHM1
17:44109447	C/T	rs781056926	KANSL1
17:44627796	C/G		ARL17A,LRRC37A2
17:45387563	G/T	rs771030070	ITGB3
17:45894055	G/A	rs200258761	OSBPL7
17:46670695	G/A	rs150923692	HOXB5
17:46804358	G/A	rs139475791	HOXB13
17:47482458	G/A		PHB
17:48152974	A/T	rs987503159	ITGA3
17:48184214	G/A	rs1210065755	PDK2
17:48193254	C/T	rs963848662	SAMD14
17:48264277	G/A		COL1A1
17:48278871	A/G		COL1A1
17:48653309	C/T	rs745758403	CACNA1G
17:48655555	G/A	rs200203979	CACNA1G
17:48918082	C/T		WFIKKN2
17:48918321	C/T	rs1260918880	WFIKKN2
17:49354621	A/G	rs371731234	UTP18
17:53007554	C/G	rs549780654	TOM1L1
17:55078270	T/C	rs140053634	SCPEP1
17:56324916	C/T	rs764179599	LPO
17:56326531	A/C	rs773510542	LPO
17:56356914	T/C	rs78950939	MPO
17:56400343	G/A	rs142903376	TSPOAP1
17:56435546	G/A	rs1262556898	RNF43
17:56557617	C/T	rs117884653	HSF5
17:56584558	G/A	rs775203610	MTMR4
17:56585610	G/C	rs202018897	MTMR4
17:56598994	A/G	rs143983549	SEPTIN4
17:57139946	C/G	rs752045909	TRIM37
17:57158574	C/T	rs1306969625	TRIM37
17:57917195	T/C		VMP1
17:58144846	C/T	rs76187322	HEATR6

17:58147167	G/A	rs776890276	HEATR6
17:58504084	T/C		C17orf64
17:59481989	C/G	rs200245762	TBX2
17:59485592	A/G		TBX2
17:59543297	A/T	rs147644451	TBX4
17:59557464	G/A	rs572207593	TBX4
17:59945307	C/T		INTS2
17:59947337	C/G	rs563606143	INTS2
17:59988892	T/C	rs201755832	INTS2
17:60033074	T/C	rs778174072	MED13
17:60518006	G/C	rs377157364	METTL2A
17:60769802	A/G	rs191466123	MRC2
17:60782939	A/G		MARCHF10
17:61271384	T/G		TANC2
17:61497517	C/A	rs764219146	TANC2
17:61497830	C/T	rs749905545	TANC2
17:61498178	T/A	rs369696442	TANC2
17:61568688	G/A	rs143507892	ACE
17:61613081	C/T	rs79670910	KCNH6
17:61615793	G/A	rs1310354060	KCNH6
17:61949554	T/C	rs753272366	CSH2
17:61972464	C/G		CSH1
17:62121544	T/C	rs61738538	ERN1
17:62248535	T/C	rs150936754	TEX2
17:63957694	G/A	rs77905043	CEP112
17:64173100	C/T	rs146984975	CEP112
17:64876773	C/T	rs149159754	CACNG5
17:65116707	C/G	rs753206228	HELZ
17:65353513	A/G	rs144951189	PSMD12
17:66429649	C/T	rs375682247	PRKAR1A,WIPI1
17:67013871	C/T		ABCA9
17:67084360	A/C	rs201339605	ABCA6
17:68128332	G/A	rs146940841	KCNJ16
17:70119918	C/G	rs202028563	SOX9
17:71084834	C/A	rs141558950	SLC39A11
17:71193412	A/G		COG1
17:71201749	C/T	rs767006562	COG1
17:71205733	T/C		FAM104A
17:71282359	C/T	rs201116432	CDC42EP4
17:71361498	A/G		SDK2
17:71390400	C/T	rs758597712	SDK2
17:72295986	C/T	rs141079076	DNAI2
17:72306270	C/T	rs61736879	DNAI2
17:72350690	G/T	rs780918742	KIF19
17:72764620	A/T	rs141613848	SLC9A3R1
17:72791167	C/G	rs757827050	TMEM104
17:72832237	G/A	rs768576277	TMEM104
17:72832629	G/A	rs746264120	TMEM104
17:72843020	C/G		GRIN2C
17:73144654	C/T	rs756370285	JPT1

17:73227951	G/T		NUP85
17:73236481	G/A	rs755187544	GGA3
17:73262833	T/A	rs138489085	MIF4GD
17:73282722	G/A	rs753574313	SLC25A19
17:73509819	C/T		CASKIN2
17:73518268	A/C		TSEN54
17:73555418	G/A	rs764296862	LLGL2
17:73560480	G/A	rs373166096	LLGL2
17:73738426	G/A	rs139092703	ITGB4
17:73738746	G/T	rs751621684	ITGB4
17:73738819	A/G		ITGB4
17:73832671	C/T	rs200661413	UNC13D
17:73836640	C/T	rs560990369	UNC13D
17:73910030	C/G	rs773020817	FBF1
17:73916166	C/T	rs760441571	FBF1
17:73922167	G/C	rs201197761	FBF1
17:73946948	G/C		ACOX1
17:74005981	T/C	rs139261978	EVPL
17:74077707	G/C	rs146722649	EXOC7,ZACN
17:74309934	C/T	rs779431619	PRPSAP1
17:74921178	C/T	rs754324770	MGAT5B
17:74922744	C/T	rs140999399	MGAT5B
17:76075557	G/A	rs192860150	TNRC6C
17:76100733	G/A	rs752119878	TNRC6C
17:76133406	T/G	rs753900323	TMC8
17:76526572	T/C	rs201371597	DNAH17
17:76794528	C/G	rs1484906547	USP36
17:76823424	C/T	rs866645554	USP36
17:76989775	C/T	rs145516713	CANT1
17:77044070	G/A	rs113589829	C1QTNF1
17:77078045	G/A	rs539480225	ENGASE
17:77079118	G/A	rs200703027	ENGASE
17:78039371	G/A	rs1449822865	CCDC40
17:78223030	G/A	rs150834163	SLC26A11
17:78310056	G/C	rs116694967	RNF213
17:78319297	G/A	rs375507267	RNF213
17:78320219	C/T	rs202096577	RNF213
17:78343339	T/G	rs1056889653	RNF213
17:78355462	C/T	rs141301945	RNF213
17:78704402	A/G		RPTOR
17:78727933	G/A		RPTOR
17:79409733	G/C	rs201429848	BAHCC1
17:79411578	G/T	rs555714796	BAHCC1
17:79425442	C/T	rs200684776	BAHCC1
17:79504090	C/T	rs752007056	FSCN2
17:79813145	G/A	rs146846893	P4HB
17:79899541	C/T	rs759999357	MYADML2
17:79912135	C/T	rs149932029	NOTUM
17:79983003	G/A	rs779738162	LRRC45
17:80016265	G/A	rs368733115	DUS1L

17:80049410	C/T		FASN
17:80789037	C/G	rs145716788	TBCD,ZNF750
17:81052168	G/A	rs770115888	METRNL
18:677784	T/G		ENOSF1
18:3141984	G/C	rs201104206	MYOM1
18:3164329	C/A		MYOM1
18:3879164	G/A	rs146753741	DLGAP1
18:5197050	C/T		AKAIN1
18:5419732	G/A	rs769159490	EPB41L3
18:5443872	A/C	rs1391830450	EPB41L3
18:6890538	G/T	rs74414891	ARHGAP28
18:8387085	C/T	rs746302735	PTPRM
18:8718560	C/T	rs780521521	MTCL1
18:8784671	G/C	rs749117532	MTCL1
18:8784791	C/T	rs150423289	MTCL1
18:10485583	G/A		APCDD1
18:10680295	G/T		PIEZO2
18:10704486	G/A	rs1005911734	PIEZO2
18:11851557	A/G	rs777911714	CHMP1B,GNAL
18:12697321	C/G	rs74943012	CEP76,PSMG2
18:13681620	A/C		FAM210A
18:19034509	C/T	rs185607659	GREB1L
18:20548813	A/G	rs146649234	RBBP8
18:20548818	C/T	rs373804633	RBBP8
18:20581576	T/C	rs766294871	RBBP8
18:20602138	C/G	rs760235924	RBBP8
18:20602153	G/A	rs140196819	RBBP8
18:21393042	C/T	rs760179701	LAMA3
18:21427457	G/A		LAMA3
18:21898567	T/G	rs749732043	OSBPL1A
18:22805510	G/A	rs910966903	ZNF521
18:23619364	C/T	rs753267000	SS18
18:25565704	G/T	rs775910155	CDH2
18:28576861	G/T	rs1229007764	DSC3
18:28609476	T/C	rs747471270	DSC3
18:28654750	G/A	rs148185335	DSC2
18:28916375	C/T	rs757724113	DSG1
18:28993231	T/G	rs373510855	DSG4
18:29264353	G/T		B4GALT6
18:29339765	T/C	rs140842640	SLC25A52
18:30349947	T/C	rs201012096	KLHL14
18:30350326	C/T	rs913767156	KLHL14
18:31463288	T/G		NOL4
18:31538235	C/T	rs147765916	NOL4
18:33554988	C/G		C18orf21
18:33573139	G/A	rs752379681	RPRD1A
18:33747114	A/G	rs151280482	ELP2
18:34261475	C/T	rs141148037	FHOD3
18:40854263	T/C		SYT4
18:42532513	C/T	rs772971136	SETBP1

18:43418689	C/G	rs200427190	SIGLEC15
18:43490657	T/C	rs200456950	EPG5
18:44435342	C/T	rs1419904526	PIAS2
18:44601681	T/C		KATNAL2
18:44683809	C/G	rs778702869	IER3IP1
18:46284447	C/T	rs148673663	CTIF
18:47402106	A/C	rs201109748	MYO5B
18:47488689	C/G	rs200175136	MYO5B
18:47506839	C/T	rs189027956	MYO5B
18:47778145	A/C	rs1237651268	CFAP53
18:47788424	C/T	rs200378044	CFAP53
18:48333156	T/C		MRO
18:51750538	G/C	rs955088364	MBD2
18:54362409	G/A		WDR7
18:54385255	T/A	rs1448649632	WDR7
18:56651694	A/C	rs200279390	ZNF532
18:56998653	T/C	rs1419731292	LMAN1
18:59815467	A/C	rs1035743375	PIGN
18:61060722	A/G	rs140107443	VPS4B
18:61156603	A/T	rs750045101	SERPINB5
18:61377525	T/C		SERPINB11
18:63511148	C/T	rs142208974	CDH7
18:71825690	A/G		TIMM21
18:71930694	C/T	rs762633432	CYB5A
18:72250887	T/C	rs748012091	CNDP1
18:72913771	T/C	rs146651397	ZADH2
18:74611139	G/T		ZNF236
18:76870408	G/T	rs763076944	ATP9B
18:77475409	C/T	rs1227734595	CTDP1
19:111373	C/G		OR4F17
19:464323	T/C	rs1176166796	ODF3L2
19:629547	G/A	rs1339218084	POLRMT
19:643318	C/T	rs529828160	FGF22
19:863117	G/T		CFD
19:919527	T/G	rs765739273	KISS1R
19:920455	T/C	rs747568732	KISS1R
19:1007961	G/A	rs201937512	GRIN3B
19:1119964	G/A	rs775151184	SBNO2
19:1220596	C/T	rs587782468	STK11
19:1455159	T/C		APC2
19:1461055	G/A	rs565171705	APC2
19:1619427	C/T	rs201296678	TCF3
19:1625670	G/C	rs1167586863	TCF3
19:1632333	G/A	rs368020893	TCF3
19:1787157	A/T	rs200106210	ATP8B3
19:1791770	C/G		ATP8B3
19:1796211	C/T		ATP8B3
19:1825845	C/T	rs148840948	REXO1
19:2110214	T/C	rs762694144	AP3D1
19:2434008	C/T	rs770685785	LMNB2

19:2811698	C/T	rs146226928	THOP1
19:3019741	G/A	rs200839990	TLE2
19:3547545	C/T	rs746832966	MFSD12
19:3638919	G/A	rs760888987	PIP5K1C
19:3740732	C/T	rs144553189	TJP3
19:3751269	C/T	rs201978209	APBA3
19:3885809	T/A		ATCAY
19:3936637	G/A	rs376394284	NMRK2
19:3938675	C/T	rs1005088739	NMRK2
19:4171931	C/T	rs201461744	CREB3L3
19:4446312	C/T	rs572649360	UBXN6
19:4529803	G/A	rs200136383	PLIN5
19:4847825	G/A	rs146789635	PLIN3
19:5218538	C/T	rs767763908	PTPRS
19:5694556	C/T	rs890805355	LONP1
19:5785231	C/G	rs753239315	DUS3L
19:5824343	A/C	rs757355174	NRTN
19:6008200	C/T	rs200258235	RFX2
19:6333561	A/G	rs990325686	ACER1
19:6373009	C/T	rs777018263	ALKBH7
19:6374878	T/C	rs746312399	ALKBH7
19:6466580	G/A	rs771187848	CRB3
19:6495666	G/C		TUBB4A
19:6903880	A/T	rs143245445	ADGRE1
19:7523505	G/A	rs201240706	ARHGEF18
19:7542250	G/T		PEX11G
19:7677714	C/T	rs370407145	CAMSAP3
19:7710122	C/T	rs199924392	STXBP2
19:8140008	C/T	rs142975316	FBN3
19:8146280	C/T	rs774277592	FBN3
19:8151104	G/C	rs372213878	FBN3
19:8174601	T/C	rs200277415	FBN3
19:8176939	C/T	rs770795850	FBN3
19:8196695	G/C		FBN3
19:8206710	C/A	rs201984325	FBN3
19:8209823	C/A	rs554641249	FBN3
19:8326672	G/A	rs141592194	CERS4
19:8381461	C/T	rs1001956315	NDUFA7
19:8386216	C/A	rs897507955	NDUFA7
19:9028283	G/T		MUC16
19:9644620	A/C	rs562808537	ZNF426
19:9801176	A/G	rs765786628	
19:9868501	C/T	rs78118057	ZNF846
19:10084443	C/T	rs534275127	COL5A3
19:10225208	C/T	rs146829743	P2RY11,PPAN-P2RY11
19:10254466	T/C		DNMT1
19:10685662	C/A	rs1395575240	AP1M2
19:11034772	T/C	rs767131387	YIPF2
19:11034784	G/A		YIPF2
19:11036439	C/T	rs747869037	YIPF2

19:11095952	A/G	rs1225673675	SMARCA4
19:11230817	A/G	rs1202258257	LDLR
19:11356363	A/T		DOCK6
19:11447948	G/A	rs112584280	RAB3D
19:11485468	G/C		SWSAP1
19:11516014	C/G	rs201989374	RGL3
19:11598443	C/T	rs148120504	ZNF653
19:11728051	C/T	rs780005956	ZNF627
19:11917922	G/A	rs148440087	ZNF491
19:11917970	A/C	rs116584868	ZNF491
19:11942847	T/C	rs766719383	ZNF440
19:12126866	A/T	rs747038782	ZNF433
19:12501435	A/G	rs8112445	ZNF799
19:12780007	C/A		WDR83,WDR83OS
19:12786932	A/G	rs200308259	DHPS
19:12864100	G/A	rs200640940	BEST2
19:12881860	C/T	rs780475839	HOOK2
19:12902850	C/T		JUNB
19:13008632	G/A	rs121434372	GCDH
19:13008638	C/T	rs121434369	GCDH
19:13010300	C/T	rs121434367	GCDH,SYCE2
19:13035588	C/T	rs762404457	FARSA
19:13067768	C/T	rs1214926366	GADD45GIP1
19:13397745	G/C	rs758178315	CACNA1A
19:13879797	T/A	rs754730419	MRI1
19:13915707	G/T		ZSWIM4
19:14038745	G/T		CC2D1A
19:14046591	G/A	rs761138263	PODNL1
19:14261714	C/T	rs779694675	ADGRL1
19:14273726	C/T	rs201495192	ADGRL1
19:14584927	G/A		PTGER1
19:14629073	C/T	rs758991587	DNAJB1
19:14682757	G/A	rs369947466	NDUFB7
19:14743808	A/C	rs142461083	ADGRE3
19:14877148	G/T	rs61732009	ADGRE2
19:14910074	A/C	rs569136507	OR7C1
19:15083718	C/T	rs201428414	SLC1A6
19:15220607	G/C	rs754569398	SYDE1
19:15233769	G/A	rs776112111	ILVBL
19:15272378	C/T	rs199620476	NOTCH3
19:15285063	G/T	rs141320511	NOTCH3
19:15288693	G/A	rs937871148	NOTCH3
19:15338878	C/T	rs199794526	EPHX3
19:15376295	G/C		BRD4
19:15905115	T/C	rs139928028	OR10H5
19:15990223	G/A	rs142113670	CYP4F2
19:16038111	G/A	rs57519667	CYP4F11
19:16640608	C/G		CHERP
19:17264841	C/T	rs188640121	MYO9B
19:17317090	T/C	rs760233368	MYO9B

19:17366348	C/T	rs372812911	USHBP1
19:17644400	A/G		NIBAN3
19:17768899	C/T	rs200805380	UNC13A
19:17881605	A/G	rs148568413	FCHO1
19:17927854	A/G	rs201125714	INSL3
19:17941341	A/G	rs145260622	JAK3
19:17974011	G/A	rs752823010	RPL18A
19:18119160	A/G	rs553414947	ARRDC2
19:18120471	C/T	rs757352581	ARRDC2
19:18899519	C/T	rs1049944784	COMP
19:18979554	A/G	rs1452075582	CERS1,GDF1
19:19049218	T/G		HOMER3
19:19115400	C/T	rs749309984	SUGP2
19:19243474	T/C	rs1050456995	TMEM161A
19:19258567	C/G	rs768517320	BORCS8-MEF2B,MEF2B
19:19603428	G/C		GATAD2A
19:19609387	A/C	rs201335898	GATAD2A
19:19616246	C/T	rs1262732385	GATAD2A
19:19654895	C/T	rs139539877	CILP2
19:19655156	G/A		CILP2
19:19905753	A/C	rs756416485	ZNF506
19:20727645	C/A	rs782176001	ZNF737
19:24116750	A/G	rs534116402	ZNF726
19:30499943	G/A	rs759629218	URI1
19:32844574	A/G	rs144062945	ZNF507
19:33355167	C/T	rs121908480	SLC7A9
19:33585125	G/T	rs369545403	GPATCH1
19:33706843	T/C	rs774785374	SLC7A10
19:33882259	G/A	rs200183031	PEPD
19:34297907	T/C		KCTD15
19:35617778	G/A	rs145824616	LGI4
19:35757639	C/T		LSR
19:36035845	C/G	rs1022280988	GAPDHS
19:36108237	C/T	rs201575634	HAUS5
19:36230353	G/T		IGFLR1
19:36231381	C/A	rs140154415	IGFLR1
19:36246448	G/T	rs1479469357	HSPB6
19:36297671	G/A	rs148996461	PRODH2
19:36339660	G/T	rs570069789	NPHS1
19:36370111	G/A	rs148071646	APLP1
19:36727578	A/G		ZNF146
19:37853586	G/A	rs139305805	ZNF875
19:37975197	A/G	rs200756675	ZNF570
19:38189389	A/G	rs145573052	ZNF607
19:38190175	C/T	rs1180591093	ZNF607
19:38591782	G/A	rs761856853	SIPA1L3
19:38610014	G/A	rs139903780	SIPA1L3
19:38746819	T/G		PPP1R14A
19:38901994	G/A	rs202093358	RASGRP4
19:38910571	C/G		RASGRP4

19:38910823	C/T	rs757379439	RASGRP4
19:38956856	G/A	rs180714609	RYR1
19:38996550	A/T	rs144777676	RYR1
19:39016027	C/T		RYR1
19:39025421	C/T	rs146361173	RYR1
19:39052029	C/T		RYR1
19:39208572	C/G	rs755318100	ACTN4
19:39230852	C/T	rs62617084	CAPN12
19:39340461	C/G	rs1211277623	HNRNPL
19:39371505	C/T	rs930042447	SIRT2
19:39374297	G/A	rs199646012	SIRT2
19:39516139	T/G	rs201679838	FBXO27
19:39590903	G/C		ACP7
19:39734673	T/C	rs144005418	IFNL3
19:39914077	G/A	rs114741660	PLEKHG2
19:39914237	G/A	rs199844800	PLEKHG2
19:39976915	C/T		TIMM50
19:40382382	C/T	rs1224494493	FCGBP
19:40721017	C/T	rs757496454	MAP3K10
19:40957295	C/T	rs754195224	BLVRB
19:41190390	A/T		NUMBL
19:41239241	C/T	rs146632462	ITPKC
19:41889672	G/A	rs200464270	TMEM91
19:42794922	G/A	rs780133950	CIC
19:42880088	C/T	rs762303782	MEGF8
19:44056954	G/A	rs143917286	XRCC1
19:44273625	G/T	rs76935412	KCNN4
19:44680844	T/C	rs201119111	ZNF226
19:44740552	G/A	rs745389908	ZNF227
19:45004288	C/T	rs147782104	ZNF180
19:45157256	A/G	rs142546426	PVR
19:45175975	G/A		CEACAM19
19:45260458	T/C		BCL3
19:45406334	C/T		TOMM40
19:45494509	A/G	rs140564801	CLPTM1
19:45852084	G/A	rs763647964	KLC3
19:45867721	G/A	rs137910235	ERCC2
19:45868306	G/T		ERCC2
19:45911645	A/T	rs1485530244	CD3EAP,ERCC1
19:45917240	G/A	rs879737645	ERCC1
19:45975908	A/G		FOSB
19:45997556	T/C		RTN2
19:46180366	G/A	rs746846121	GIPR
19:46201866	G/A	rs1211700140	QPCTL
19:46270265	A/G	rs1348543725	SIX5
19:46280721	G/C	rs142936719	DMPK
19:46522877	A/G	rs146282647	PGLYRP1
19:47542783	G/A	rs201519697	NPAS1
19:47646764	C/T	rs778336657	SAE1
19:47646779	G/A	rs199801330	SAE1

19:47729951	G/A	rs1272445133	BBC3
19:47778298	C/T	rs756117594	INAFM1
19:47856979	A/C		DHX34
19:47912716	C/A	rs1232016620	MEIS3
19:47920155	C/T	rs150969429	MEIS3
19:48219888	C/T	rs1293046467	EHD2
19:48239752	A/G	rs138130337	EHD2
19:48259063	C/T	rs115908259	NOP53
19:48337728	C/G	rs139340178	CRX
19:48342749	A/G	rs61748442	CRX
19:48967720	C/G	rs200201274	KCNJ14
19:49116194	C/T	rs1324044474	FAM83E
19:49142833	C/T	rs145539923	CA11
19:49206524	C/T	rs149356814	FUT2
19:49364915	C/A		PLEKHA4
19:49387033	C/T	rs34182743	TULP2
19:49458957	C/G		BAX
19:49564984	G/A	rs1015733961	NTF4
19:49621239	G/A		LIN7B
19:49631677	G/C	rs757661523	PPFIA3
19:49641578	C/A	rs1296984041	PPFIA3
19:49671244	G/A	rs149335121	TRPM4
19:49692275	G/T		TRPM4
19:49971731	G/A	rs766785153	ALDH16A1
19:50172125	T/G	rs199837123	BCL2L12
19:50312016	C/A	rs368721486	FUZ
19:50367585	A/G		PNKP
19:50384713	C/T	rs201954182	TBC1D17
19:50412640	A/G	rs774836417	IL4I1,NUP62
19:50498129	T/C	rs751105355	VRK3
19:50720992	G/A	rs138001307	MYH14
19:50726342	C/T	rs200818171	MYH14
19:50730169	A/G	rs200424400	MYH14
19:50764765	C/T	rs767984672	MYH14
19:50832217	C/A	rs185017345	KCNC3
19:50862765	C/T	rs202120944	NAPSA
19:50912821	G/C	rs144143245	POLD1
19:50917023	G/A	rs145473716	POLD1
19:50939077	G/A	rs25669	MYBPC2
19:50964929	C/T		MYBPC2
19:50983471	C/T	rs770232858	EMC10
19:51378000	C/T	rs61750342	KLK2
19:51446988	A/G		KLK5
19:51501014	G/A	rs774076468	KLK8
19:51528012	C/T	rs146041341	KLK11
19:51581316	G/T	rs776407764	KLK14
19:51607530	C/A	rs780016404	CTU1
19:52130940	A/G	rs200372912	SIGLEC5
19:52887905	G/A	rs774616370	ZNF880
19:53086294	T/C		ZNF701

19:53454244	A/G		ZNF816,ZNF816-ZNF321P
19:53644892	C/T	rs200933013	ZNF347
19:53740802	T/G	rs750611049	ZNF677
19:54313027	G/T	rs146250162	NLRP12
19:54314447	G/A	rs145803984	NLRP12
19:54314489	C/T	rs34330210	NLRP12
19:54611661	C/T	rs753104719	TFPT
19:54677941	C/T	rs745322441	MBOAT7
19:54695243	C/T		TSEN34
19:54778656	C/T	rs1387032357	LILRB2
19:55815477	G/C		BRSK1
19:55967028	C/G	rs771065603	ISOC2
19:55998115	G/T	rs1382978244	NAT14
19:56002423	G/A	rs899272601	SSC5D
19:56047439	G/A	rs201910900	SBK2
19:56133349	G/A		ZNF784
19:56197009	C/T	rs202152406	EPN1
19:56538867	C/T	rs374218026	NLRP5
19:56701539	G/T	rs753726468	ZSCAN5B
19:57089572	C/T	rs780290642	ZNF470
19:57326850	T/C	rs149044578	PEG3,ZIM2
19:58512659	C/A	rs201438785	ZNF606
19:58579278	C/T	rs769186698	ZNF135
19:58773501	A/C	rs1235564626	ZNF544
19:59074080	A/G	rs368446779	MZF1
20:368890	G/C	rs61740295	TRIB3
20:372180	C/A	rs149447454	TRIB3
20:1285832	C/T	rs151237144	SNPH
20:1616005	T/C		SIRPG
20:1961011	C/G		PDYN
20:1961042	C/T	rs76243088	PDYN
20:2315875	G/C		TGM3
20:3102281	T/C	rs749895428	UBOX5
20:3652532	A/C	rs371215440	ADAM33
20:3654474	C/T		ADAM33
20:4680494	G/A	rs74315407	PRNP
20:6064801	C/T	rs373229817	FERMT1
20:6088238	C/G		FERMT1
20:6096551	G/A	rs141690919	FERMT1
20:10626115	T/A	rs367807001	JAG1
20:13279820	G/T	rs546009979	ISM1
20:13747453	T/C	rs199605089	ESF1
20:16359795	T/C		KIF16B
20:17602123	G/A	rs761007374	RRBP1
20:17617260	G/A	rs137981542	RRBP1
20:17936061	A/C		SNX5
20:18491625	C/T	rs151126375	SEC23B
20:18568727	T/C		DTD1
20:19560692	G/A	rs752244122	SLC24A3
20:19937317	C/T	rs372531454	RIN2

20:20079388	C/G	rs369098678	CFAP61
20:20269293	C/T	rs140045830	CFAP61
20:21687694	T/C	rs745309335	PAX1
20:21690047	C/T	rs758771203	PAX1
20:23335085	A/G		NXT1
20:23345727	C/G	rs139046142	GZF1
20:23966584	G/A	rs541165436	GGTLC1
20:23966591	T/A	rs146809011	GGTLC1
20:24944523	C/T	rs151131612	APMAP
20:25004239	G/A	rs137980069	ACSS1
20:25062683	A/G	rs74315436	VSX1
20:25481559	C/T	rs140736402	NINL
20:25656540	A/T	rs183453408	ZNF337
20:30136871	G/A	rs751453148	HM13
20:30432486	G/T	rs148893203	FOXS1
20:30433096	G/A	rs952353034	FOXS1
20:30729441	G/A	rs199931289	TM9SF4
20:31044081	G/A	rs143368888	NOL4L
20:31583439	C/T	rs17123951	SUN5
20:31690809	G/A	rs758458637	BPIFB4
20:32031267	C/G	rs786205848	SNTA1
20:32031315	C/T		SNTA1
20:32664583	G/A	rs374081764	RALY
20:32878442	A/G	rs140810436	AHCY
20:33169370	A/C	rs753336143	PIGU
20:33328946	T/G	rs746386264	NCOA6
20:33575641	A/G	rs75044008	MYH7B
20:33585244	G/A	rs779672127	MYH7B
20:33588858	A/C	rs191392262	MYH7B
20:33703538	G/C	rs772894746	EDEM2
20:33876334	G/A	rs777921694	FAM83C
20:34207531	T/A	rs569605887	SPAG4
20:34278477	C/T	rs141945347	NFS1
20:34319941	C/T	rs757598805	RBM39
20:34763596	G/A	rs772632635	EPB41L1
20:34828371	G/A	rs201316006	AAR2
20:35243673	G/A	rs142111439	SLA2
20:35243730	G/A	rs375445555	SLA2
20:35399342	G/A	rs750211417	DSN1
20:35422399	C/G		SOGA1
20:35425300	C/T	rs774853570	SOGA1
20:35517694	G/A	rs1452135342	TLDC2
20:35632212	G/A	rs149409083	RBL1
20:37580402	C/T	rs61752513	FAM83D
20:40043870	C/T	rs377423955	CHD6
20:42086744	G/A	rs1483956248	SRSF6
20:42694707	A/G		TOX2
20:42825810	G/C		OSER1
20:43251230	G/A	rs559798694	ADA
20:43726359	C/G	rs1362594177	KCNS1

20:43945573	A/C	rs199904334	RBPJL
20:44416558	T/C	rs375796890	WFDC3
20:44528166	G/A	rs772104731	PLTP
20:44538228	G/A	rs377218597	PLTP
20:44592386	T/C	rs770286985	ZNF335
20:44698960	C/T		NCOA5
20:46268423	C/G	rs142951578	NCOA3
20:46288159	G/T	rs1269300022	SULF2
20:46292275	T/C	rs200041967	SULF2
20:46313182	A/G	rs769976195	SULF2
20:49214219	G/A	rs757577863	RIPOR3
20:49221199	G/A	rs369483118	RIPOR3
20:50092091	G/A	rs145327846	NFATC2
20:50704973	G/A	rs1003446473	ZFP64
20:50705130	G/T	rs771070268	ZFP64
20:52198557	T/G		ZNF217
20:52611552	G/A	rs60878965	BCAS1
20:52789512	G/T	rs149806586	CYP24A1
20:55206525	G/T	rs1382089333	TFAP2C
20:55918508	T/C	rs573681690	SPO11
20:55966729	C/G		RBM38
20:56138114	C/T	rs765716997	PCK1
20:56139619	C/T	rs148603002	PCK1
20:57598870	C/G	rs202177647	TUBB1
20:60776035	G/A	rs368976452	MTG2
20:60893539	G/A	rs147913341	LAMA5
20:60897722	G/A	rs201815547	LAMA5
20:60899224	G/A	rs141989486	LAMA5
20:60903022	G/A	rs149169462	LAMA5
20:60928304	A/G	rs150937347	LAMA5
20:61048484	G/A	rs782112827	GATA5
20:61288317	C/T	rs769542474	SLCO4A1
20:61525450	G/C	rs201113903	DIDO1
20:61528337	C/T	rs758044081	DIDO1
20:61869332	G/A	rs766219180	BIRC7
20:61873945	G/A	rs80100531	NKAIN4
20:62172690	T/A		SRMS
20:62174803	C/T	rs147645098	SRMS
20:62187743	C/T	rs138076587	FNDC11
20:62198553	G/C	rs200878475	HELZ2
20:62198783	A/G	rs1390141998	HELZ2
20:62227104	C/T	rs895484137	GMEB2
20:62273477	T/G	rs749257260	STMN3
20:62421567	C/T	rs750696863	ZBTB46
20:62493779	C/T	rs769256438	ABHD16B
20:62729313	A/G		OPRL1
20:62737946	G/C		NPBWR2
21:16337084	A/C	rs61733441	NRIP1
21:18966500	G/A	rs542747898	BTG3
21:27136963	A/G		GABPA

21:30380359	A/G	rs139428854	RWDD2B
21:31587768	C/G	rs757500351	CLDN8
21:31587963	G/A	rs61743791	CLDN8
21:31744383	G/A		KRTAP13-2
21:33719468	C/G	rs1363788374	URB1
21:33723059	T/A	rs147782087	URB1
21:33740773	G/A	rs987800674	URB1
21:35093522	C/T	rs201302056	ITSN1
21:35275936	G/A	rs191960490	ATP5PO
21:37572667	C/T	rs201491075	DOP1B
21:37610969	G/C	rs138787675	DOP1B
21:37618321	G/A	rs189803648	DOP1B
21:37618896	C/A	rs150476018	DOP1B
21:37833630	C/T	rs769599110	CLDN14
21:38444764	C/T	rs148069538	PIGP
21:40650683	G/C	rs775813683	BRWD1
21:42080383	C/G	rs764522059	DSCAM
21:42762528	C/A	rs138600033	MX2
21:42807860	C/G	rs1225918873	MX1
21:43161141	C/T	rs369156558	RIPK4
21:43529782	C/A	rs372915114	UMODL1
21:43796775	G/C		TMPRSS3
21:43802210	C/T	rs181949335	TMPRSS3
21:44492171	G/A	rs201372812	CBS
21:45064211	G/A	rs80027854	HSF2BP
21:45096183	G/A	rs770126824	RRP1B
21:45139194	G/C	rs1252826064	PDXK
21:45499479	C/T	rs145051918	TRAPPC10
21:45679399	C/T	rs147370440	DNMT3L
21:45726581	C/G		PFKL
21:46086542	C/T	rs782611058	KRTAP12-2,TSPEAR
21:47545911	G/A	rs200585528	COL6A2
21:47549283	G/A	rs145838734	COL6A2
21:47549286	C/T	rs370636207	COL6A2
21:47664999	C/T	rs1437791500	MCM3AP
21:47685242	G/T	rs142223686	MCM3AP
21:47766774	C/A		PCNT
21:47848387	C/T	rs143367154	PCNT
21:47958405	C/T	rs1024530625	DIP2A
22:17662794	T/C	rs376785840	ADA2
22:18022111	A/G	rs199565531	CECR2
22:18301432	G/A	rs377121868	MICAL3
22:18923574	A/G		PRODH
22:19028606	G/A		DGCR2
22:19220018	G/A	rs201800229	CLTCL1
22:19222211	C/T	rs199652160	CLTCL1
22:19961730	G/A	rs146960902	ARVCF
22:19964986	G/A	rs199498113	ARVCF
22:20100975	C/G	rs545196562	TRMT2A
22:20307272	G/T		DGCR6L

22:20760157	C/G	rs750614730	ZNF74
22:20760960	A/G	rs1330597377	ZNF74
22:20796623	C/T	rs769050181	KLHL22
22:21134082	C/T	rs930125664	PI4KA,SERPIND1
22:21331055	G/C		AIFM3
22:21348289	C/T	rs762005490	LZTR1
22:21351223	T/C		LZTR1
22:21384598	C/T	rs148642327	SLC7A4
22:21385192	C/T	rs760027253	SLC7A4
22:21570900	G/A	rs765022784	
22:21576460	C/T	rs1362424517	
22:22288467	G/A	rs745928402	PPM1F
22:22318638	T/G	rs768550763	TOP3B
22:22318667	T/C	rs754384547	TOP3B
22:22328770	A/G	rs1601857012	TOP3B
22:22868807	A/G	rs143870620	ZNF280A
22:24095316	C/T	rs1449570113	VPREB3
22:24181174	T/C	rs758393535	DERL3
22:24220011	C/T	rs141517261	SLC2A11
22:24573742	C/T		CABIN1
22:24829703	C/T	rs773743606	ADORA2A
22:24909296	C/T	rs199788632	UPB1
22:24982063	C/T	rs201574647	GGT1,LRRC75B
22:25282688	G/C	rs199841996	SGSM1
22:25586689	A/G	rs1458579490	KIAA1671
22:25755908	G/A	rs200483964	LRP5L
22:25755980	G/A	rs148330254	LRP5L
22:26159253	T/C	rs200574321	MYO18B
22:26164256	G/T	rs1432756622	MYO18B
22:26176101	G/A	rs201070289	MYO18B
22:26194004	C/T	rs371252378	MYO18B
22:26868821	C/T		HPS4
22:27026357	G/A		CRYBA4
22:29130520	C/T	rs141568342	CHEK2
22:29446183	T/C	rs758052664	ZNRF3
22:29533437	C/T	rs61752343	KREMEN1
22:29752563	A/G		AP1B1
22:29879546	G/A	rs565974716	NEFH
22:29885570	G/C	rs200634512	NEFH
22:29904484	T/G		THOC5
22:30189362	G/A	rs1161121853	ASCC2
22:30189598	G/A	rs964205248	ASCC2
22:30421768	C/A	rs773098171	MTMR3
22:30689777	G/A	rs199890944	TBC1D10A
22:30802369	C/T	rs766765779	SEC14L2
22:31022447	G/A		TCN2
22:31492781	C/T	rs34292278	SMTN
22:31530325	C/T	rs185902428	INPP5J
22:31722915	C/T	rs771418683	PATZ1
22:31858938	C/T	rs368717204	EIF4ENIF1

22:32003938	C/T	rs1027884417	SFI1
22:32019800	A/C	rs772273910	PISD
22:32587105	C/T	rs201619870	RFPL2
22:32828366	C/A	rs150852332	BPIFC
22:33673171	G/A	rs1408447253	LARGE1
22:35478570	C/G	rs549935545	ISX
22:35719028	A/G	rs139147613	TOM1
22:35782919	T/C		HMOX1
22:37603379	C/T	rs376489598	SSTR3
22:37904541	C/A		CARD10
22:38161714	C/T	rs201724032	TRIOBP
22:38234593	A/T		ANKRD54
22:38271891	A/G	rs763759372	EIF3L
22:38894492	G/A		DDX17
22:39134681	G/A	rs141013997	SUN2
22:39772080	G/C	rs78665292	SYNGR1
22:39826178	G/A	rs769248693	TAB1
22:39996653	G/A		CACNA1I
22:40391381	C/T		FAM83F
22:40815063	C/T	rs140573764	MRTFA
22:41223177	C/T		ST13
22:41278148	G/C		XPNPEP3
22:41527446	G/A		EP300
22:41564858	C/G		EP300
22:41574152	C/A	rs745528077	EP300
22:41574892	C/G	rs377508897	EP300
22:41623138	G/A	rs780186631	L3MBTL2
22:41974855	C/T	rs765001447	PMM1
22:41980057	G/T	rs138410974	PMM1
22:42177378	C/T	rs200777038	MEI1
22:42524229	G/T	rs142378906	CYP2D6
22:42525058	C/A	rs774943042	CYP2D6
22:42526687	C/G		CYP2D6
22:42608157	A/G		TCF20
22:42908935	C/T	rs143684973	RRP7A
22:43570521	G/A		TTLL12
22:43576914	C/T	rs372418774	TTLL12
22:44004459	C/T		EFCAB6
22:44221961	C/T	rs374057069	SULT4A1
22:45132666	C/T	rs756128125	PRR5,PRR5-ARHGAP8
22:45255629	G/A	rs140056752	ARHGAP8,PRR5-ARHGAP8
22:45726577	G/A	rs372276519	FAM118A
22:45802354	A/G	rs199651348	SMC1B
22:45959078	C/G	rs748995133	FBLN1
22:46656794	C/G		PKDREJ
22:46719132	G/T	rs201709046	GTSE1
22:46773155	C/T	rs200562232	CELSR1
22:46829349	C/A	rs61737817	CELSR1
22:50217167	G/A	rs745892251	BRD1
22:50320944	G/A	rs756156702	CRELD2

22:50471787	T/A		
22:50616764	C/G	rs754138364	PANX2
22:50657765	G/A	rs747283271	TUBGCP6
22:50682439	G/C	rs778256941	TUBGCP6
22:50720039	G/A	rs781014569	PLXNB2
22:50893714	A/G	rs753856952	SBF1
22:50898827	G/A	rs202156491	SBF1
22:50941830	C/T	rs538396256	LMF2
22:50942290	C/T	rs200798239	LMF2
22:51042351	G/A		MAPK8IP2
22:51177754	G/A	rs372139399	ACR
