# Supplementary material for: Whole-genome sequencing analysis of semi-supercentenarians
Source: eLife. 2021 May 4;10:e57849. doi: 10.7554/eLife.57849 (PMC8096429; doi:10.7554/eLife.57849)
Supplement: Supplementary file 10. — Gene name, pvalues and the number of variants is reported. [file elife-57849-supp10.pdf]

Table 10S Genes identified using SKAT-O method in 105+/110+ and CTRL including all rare variants (genes with a nominal pvalue< 0.01 were reported). Gene name, pvalues and the number of variants is reported.

SetID	P.value	N.Marker.Test
NME1,NME1-NME2	6.06E-05	24
LOC728673,LOC101928381	8.93E-05	643
OSBPL1A,IMPACT	0.00011661	41
C1QTNF4	0.000147016	6
ZNF577	0.000214864	40
PRADC1	0.000236251	5
KRT3,KRT4	0.000258985	14
SCAMP5	0.000334175	61
FDX1L	0.000348999	8
CCNE2	0.000369969	23
STK25	0.000433314	40
NONE,MIR3648-1	0.000465948	271
AGXT,C2orf54	0.000481421	9
AMPD1,NRAS	0.000556506	15
LINC01347,CEP170	0.00061041	56
FAM138D	0.000744299	13
ATP1A3,GRIK5	0.000748957	4
FAM174B	0.000786472	126
ZNF225	0.000793821	42
LCE2D,LCE2C	0.000812827	26
ARID3A,WDR18	0.00084743	24
MKRN7P,ZNF334	0.000866243	72
MIR187	0.000872242	4
GYPB,GYPA	0.000901311	327
SNORD114-6,SNORD114-7	0.000913622	8
ADAM29	0.000914888	185
KLHL24,YEATS2	0.000957494	44
C7orf65	0.000959029	22
TMEM42,TGM4	0.001048376	17
IMP4	0.001059991	21
PRSS33	0.00108757	12
NBPF11,NBPF8	0.001281453	4
HMCES,H1FX	0.001315969	12
TBC1D3,TBC1D3	0.001391853	25
CEP68	0.001414424	67
COX11	0.001443342	12
RIBC2	0.001448361	56
GLYATL3	0.001451567	66
TSEN54	0.001531292	23
HSD3B7	0.001536785	15
UBR4,EMC1	0.001591038	5
LRRC66	0.001613207	48
C8orf48,SGCZ	0.001633856	2091

SetID	P.value	N.Marker.Test
RPE65,DEPDC1	0.001648436	42
FAM110C	0.001712347	14
OXCT2P1	0.001722792	3
MYO3A,GAD2	0.001756549	3
LRRC8A	0.001789159	86
CERCAM	0.001791201	3
DBX1,HTATIP2	0.001816325	541
VAV3	0.001845348	921
ERI3,RNF220	0.001918828	110
SKOR2	0.001939935	71
NPIPB15	0.001942985	16
NONE,LOC654342	0.002013564	172
MIR6082	0.002061412	3
MSH4;RABGGTB	0.002061412	3
SIX3-AS1	0.002061412	3
SFMBT2,ITIH5	0.002072334	423
ENDOG	0.002157316	13
ZAR1L	0.002166446	17
PAPPA	0.002174708	552
OR8H1,OR8K3	0.002198446	33
LOC100996671	0.002232897	66
IPO7,LOC644656	0.00227586	20
AQP7P1,LOC102723709	0.0022777	129
LOC100130872	0.002290099	37
CMSS1	0.002349039	112
MS4A6A	0.00236006	33
CFAP61,CRNKL1	0.00237561	3
OR1A1,OR1D4	0.002386144	43
PPP3R1,CNRIP1	0.00238945	59
TAC4	0.002404606	24
ZNF823	0.002407801	36
FAM27E2,FAM27E2	0.002431622	96
NADSYN1,KRTAP5-7	0.002461376	39
ZNF503-AS1,ZNF503	0.002484244	78
NONE,OR11H12	0.002487562	1162
MOCS3	0.002489252	8
LOC643623	0.002492991	95
COG2	0.00249767	104
FAM184B,DCAF16	0.002564715	27
AP4E1,TNFAIP8L3	0.00258464	69
C11orf74,RAG2	0.002601129	4
NRP1,LINC00838	0.002631336	983
POLR2C	0.002673887	20
VWDE,SCIN	0.002722844	436
HOXD9,HOXD8	0.002731043	4
MIR4303	0.002745288	4
ACER2	0.002751047	125
C14orf142	0.002775746	4
ARL2,ARL2-SNX15	0.002812977	4

SetID	P.value	N.Marker.Test
IL17C,CYBA	0.002814792	10
KIAA0232	0.002824026	331
FRG1HP,MIR1299	0.002832255	324
GGT3P	0.002832441	5
DEFB108B,LOC100133315	0.002869905	76
NPIPB8,EIF3C	0.002878952	5
AQP7P1	0.002911483	34
OR5K2	0.002931895	6
MKRN7P	0.002938646	5
JAG2,NUDT14	0.002978905	6
CCT6P1,LOC441242	0.003018899	30
LOC100506476,PTGR2	0.003039711	93
CBWD5,CBWD5	0.00306198	272
SDHC	0.003076433	129
FAM207A	0.003087845	97
TBC1D15,TPH2	0.00309557	17
FOXB1	0.003097158	8
SPATA42	0.00313749	7
CEP170	0.003161922	302
LINC01138	0.003162753	32
CYR61	0.003188428	14
ALS2CL	0.00318951	49
SGCZ,TUSC3	0.003197681	1188
PSG1	0.003262914	67
CERS2,ANXA9	0.003276585	11
C12orf49	0.003289089	48
LOC101927450,TLE4	0.003299662	1044
MSL2	0.003341088	177
HOXB7	0.003361192	6
YWHAZ,FLJ42969	0.003369323	247
SLC9A2,MFSD9	0.003396006	7
DPEP3	0.003397664	5
MIR22HG	0.003427207	11
LOC101928697,ANKRD26P3	0.003480937	208
PCNP,ZBTB11	0.003484406	98
C1QB,EPHB2	0.003517377	117
CHIT1,LINC01353	0.003556021	120
AP2B1	0.003567515	295
ANKRD20A4	0.003596095	26
SAMD14	0.003658939	36
PPP5D1	0.003768276	257
KAT6A	0.003876039	254
CSRP1	0.003882769	78
G0S2	0.003931091	8
FAM131A	0.003943253	30
NHSL1,FLJ46906	0.004019737	234
SMG6	0.004027748	512
ZNF235,ZNF112	0.00404049	32
KCTD3,USH2A	0.004071736	2

SetID	P.value	N.Marker.Test
ZNHIT6,COL24A1	0.004076646	48
MAP1LC3B2,C12orf49	0.004086817	321
NBPF20,NBPF9,PDE4DIP	0.004103285	312
DEFB125,DEFB126	0.004114031	79
LOC440896,FOXD4L6	0.004120858	21
TTC8	0.004126941	109
MUC19,CNTN1	0.004151609	329
FOXD3-AS1	0.004198031	6
CWH43,NONE	0.004260146	1291
ARHGAP10,NR3C2	0.004310063	18
ZNF189,ALDOB	0.004343179	16
GDF3	0.004350777	13
MUC3A	0.004387147	33
VAPB	0.00439797	140
DHRS11	0.004414573	21
ARL17A	0.004491538	18
VMO1	0.004493614	8
LOC101929470,FAM183CP	0.004511874	242
SPATA16	0.004534482	494
LOC81691	0.004558696	66
PTPN3	0.004578556	214
AQP3	0.004598749	21
NTN1,STX8	0.004612539	20
PNMAL2	0.004669159	9
CSN3	0.004675543	14
POLR2A,TNFSF12	0.004703267	78
OR5K4	0.004732118	6
BOD1	0.004734472	31
ETV5,DGKG	0.004739153	69
LINC01231,RFX3	0.004760036	51
LINC01272	0.004779786	37
MIR3976HG	0.004800834	106
PDE8B,WDR41	0.004856827	7
DEFB135,DEFB134	0.004857226	14
PPHLN1	0.004868751	224
DHRS7C	0.004920686	48
FOXD4,CBWD1	0.004965795	3
LOC100506551	0.004980772	24
PPIL6	0.004985914	119
CDRT15	0.005004159	6
SIX3,SIX2	0.005014121	157
MGME1,SNX5	0.005093043	2
SNF8,UBE2Z	0.005099305	2
RRAGD,ANKRD6	0.005128854	40
ARL6	0.005181825	56
EIF5	0.005184762	36
NRN1L,PSKH1	0.005186996	7
LINC01010,LOC101928304	0.005189045	38
MKRN2	0.005227256	60

SetID	P.value	N.Marker.Test
DLX4,DLX3	0.005238446	50
EPB42,TGM5	0.005253495	11
PLCD1,DLEC1	0.005286094	17
SIAH3,ZC3H13	0.005294262	217
CLK2	0.005314429	7
NKX2-4	0.005466254	9
SIGLEC6	0.005500155	29
EME1	0.005576252	13
RYR3	0.005633172	1189
LOC101929124	0.005652871	16
GPR108,MIR6791	0.005657086	4
SLC5A1,AP1B1P1	0.005688048	16
LOC101927651	0.005711227	44
ZMIZ1	0.005722487	548
C12orf49,RNFT2	0.005731297	3
LINC01426,RUNX1	0.005731297	3
OR8G5	0.005731297	3
MFSD14A	0.005734569	128
KPNA2,LINC00674	0.005738033	145
BDKRB1	0.005799942	32
SLC29A4,TNRC18	0.005819875	4
LOC102723373,RBL2	0.005824009	111
LOC284412	0.005866725	13
AVP	0.005874073	10
NOL4L	0.005888209	314
LINC01484	0.005895863	143
FNDC7	0.005899657	56
OR8H1	0.005903	6
KIF17	0.005932021	176
PRSS48	0.005971726	29
NAALADL2-AS2	0.005994187	77
SALRNA1,SIX1	0.006031372	5
ABCA13	0.006086268	881
CHRNA9,RBM47	0.006112259	142
BPIFA2	0.006146352	66
HELZ2,GMEB2	0.006166627	26
YPEL3	0.006210569	14
RNLS	0.006216342	738
KRTAP21-3,KRTAP21-2	0.006332888	40
EHF	0.006381136	103
CFAP44;SPICE1	0.006400835	2
LINC00520,PELI2	0.006403942	713
OR2C1,MTRNR2L4	0.006404271	33
ANKRD7	0.006410771	33
LINC00644	0.00641327	24
AXIN2	0.006417171	90
RECQL	0.006420268	45
NXPH3,SPOP	0.006429869	20
NPPB	0.006455447	7

SetID	P.value	N.Marker.Test
OR8B2	0.006492207	10
ZC3H6,RGPD8	0.006498644	84
DPY30	0.006501672	41
ANKRD34A,NBPF10,NBPF20	0.006514259	2
LINC01586,MIR1179	0.00651534	4
CRY2	0.006521275	74
ARHGAP5-AS1	0.006527511	7
SLITRK3	0.006538963	29
SELO;TRABD	0.006559695	3
NADSYN1	0.006560434	137
ZNF565	0.00657833	90
BCL2L2-PABPN1,SLC22A17	0.006611346	57
FRMD6,GNG2	0.006614766	329
SLC25A21-AS1	0.006632867	11
LOC101928436,RNPC3	0.006643088	2
LRRC36	0.006656023	96
LAMB3	0.006690092	105
ICAM4;ICAM1	0.006710139	2
RGPD4-AS1	0.006731547	2
ALPL,RAP1GAP	0.006772503	50
ZNF705E,DEFB108B	0.006773385	40
ATP5J2	0.006797764	2
TSR1	0.006812635	22
CCDC28B	0.006878048	10
IL18,TEX12	0.006888051	2
LOC101927623	0.006938839	79
TJP2,BANCR	0.0070178	92
TCF15	0.007049223	17
LINC00987	0.007051082	9
LAMC2,NMNAT2	0.007073173	2
JAKMIP1	0.007082505	520
PRR27,ODAM	0.007084226	45
SLC38A8,MBTPS1	0.007102009	35
C11orf24,LRP5	0.007105601	93
TMEM87B,FBLN7	0.007191417	41
SNORD93	0.007216601	4
PIGH	0.00726229	28
NOTCH2	0.007293973	261
MEG9,LINC00524	0.007317989	856
ZKSCAN2	0.007325699	44
TUSC3	0.007388233	669
LINC01046	0.00745296	5
IFITM3,B4GALNT4	0.007454142	157
MMAA	0.007470679	65
MIR3675,NBPF1	0.007471497	23
SDR39U1	0.007494616	6
OR56A3,OR56A5	0.007561131	38
WDR7,LINC-ROR	0.007578003	47
KCNK1	0.007582118	173

SetID	P.value	N.Marker.Test
MGARP	0.00758867	31
DNAL4,NPTXR	0.007591843	39
SLC22A23	0.007623178	528
SIX2	0.007662422	16
ABCC1,ABCC6	0.007674763	20
PCCB	0.007684632	241
LOC101927770	0.007710275	58
OPLAH,EXOSC4	0.007744513	60
CLPB,LINC01537	0.007792791	336
C1orf137	0.00783196	27
POTEKP,LINC01087	0.007848957	65
MIR8060,EPHA6	0.007854998	1242
LOC494141,SAA2-SAA4	0.007882875	24
PRKDC	0.007890985	504
ABTB2,CAT	0.007928721	127
RPS2	0.007951879	17
CSHL1	0.007994642	15
SHC4,SECISBP2L	0.008008609	40
PIK3R5	0.008024173	231
SFRP5,LINC00866	0.008033277	162
TPH1,SAAL1	0.00803741	66
DUXAP10,LINC01296	0.008105578	194
MMRN2,SNCG	0.008127436	5
FAM26D	0.008136174	29
LINC00551,LINC00443	0.008148833	38
C15orf52	0.008149892	18
RPS3A	0.008152658	19
ANO9	0.008189354	72
FMOD	0.008208105	41
C8B	0.008228604	91
UTP23,RAD21	0.008266	225
GPCPD1,C20orf196	0.008273388	363
ZNF806	0.008297492	25
LINC00701,PFKP	0.008320178	1867
SPINK4	0.0083497	24
GNB1L	0.008373077	174
FUZ,MED25	0.008396731	9
LOC100506022	0.00840081	16
CYB561D2	0.008453391	4
HRK,FBXW8	0.00846847	72
COX4I2	0.008473695	31
FREM3,GYPE	0.008506075	747
CELA2B	0.008527112	43
LINC00316,COL18A1	0.008565401	174
PIGW,GGNBP2	0.008577012	9
OR4M1,OR4N2	0.008721886	33
OR5R1,OR5M9	0.008735168	83
MCF2L	0.008787414	316
SOX17	0.008797638	7

SetID	P.value	N.Marker.Test
FAM117A,KAT7	0.008803652	42
BEND3	0.008805474	111
OR5M10,OR5M1	0.008807041	55
RFPL3S	0.008813719	27
GDNF-AS1	0.008867482	13
SGOL1-AS1	0.008884551	19
LOC102467655,PIK3R1	0.008901853	30
PYROXD1	0.008905905	78
WDR38	0.008910527	13
FCGR3B	0.008928819	48
PHLDA1,NAP1L1	0.008936475	31
MIR5684,LINC01207	0.008954931	82
C8orf22,SNTG1	0.008960419	2036
LINC00430	0.009020617	45
IBSP	0.009091568	30
OR5D13,OR5D14	0.009098366	42
VSTM2B,POP4	0.009164047	104
GSTM4,GSTM2	0.009174489	5
TRAF3IP2,FYN	0.009174508	128
ZNF738	0.009176909	102
FLJ23867,LHX4	0.009294499	49
PDK2	0.009338882	59
MIR4267	0.009343128	5
PCSK5,RFK	0.00940006	45
LINC01152	0.009404286	15
LOC613038,SMG1P2	0.00940726	7
SLC35G2	0.009410122	49
KATNAL2	0.00941511	192
AKR1C8P,AKR1C4	0.009462497	22
ALG3	0.009531771	16
SRSF10,MYOM3	0.009543662	49
UBXN8	0.009570274	48
GYPB	0.009574471	99
LOC100506076,LOC100506076	0.009626735	45
PCTP,ANKFN1	0.009637427	806
PPP1R7,ANO7	0.009713996	21
TRIM37,SKA2	0.009717859	3
EREG	0.009723497	88
RBM4	0.009751381	47
LGSN	0.00975842	87
LINC01572	0.009763748	976
SATB2-AS1	0.009764044	9
ABCA7;CNN2	0.009784505	2
OXCT1-AS1	0.009795923	7
OXSR1,SLC22A13	0.00981843	17
PRPSAP2	0.009862886	164
CEACAM4,CEACAM7	0.009873097	94
MTMR7,VPS37A	0.009876612	5
LOC650226,LOC100240728	0.009890553	67

SetID	P.value	N.Marker.Test
KRTAP10-2	0.009981482	3
DHCR7	0.009994269	47
MIR4436A,LOC654342	0.00999737	1211
