# Supplementary material for: Whole-genome sequencing analysis of semi-supercentenarians
Source: eLife. 2021 May 4;10:e57849. doi: 10.7554/eLife.57849 (PMC8096429; doi:10.7554/eLife.57849)
Supplement: Supplementary file 11. — Genes, p-value and the number of variants is reported. [file elife-57849-supp11.pdf]

Table 11S. Genes identified using SKAT-O method in 105+/110+ and CTRL including only rare damaging variants (genes with a nominal pvalue< 0.01 were reported). Genes, p-value and the number of variants is reported.

**SetID**	**P.value**	**N.Marker.Test**
PLEKHG4	0.001110916	5
PPP2R3A	0.001800377	3
DHTKD1	0.002061412	3
ANK2	0.00221473	6
RSPH10B	0.003931106	2
SHC2	0.004067972	2
COL6A6	0.005599718	4
PLB1	0.005731297	3
SCN2A	0.005731297	3
KIF19	0.005731297	3
LIMS2	0.005731297	3
DOCK4	0.006695151	2
GOLGB1	0.006831872	2
ARHGAP17	0.007103907	2
TSPEAR	0.009787176	2
PLXNB1	0.009824466	2
PON3	0.00989568	2
UPP1	0.009946891	2
BEST4	0.010071531	2
C6	0.010071531	2
CATSPER1	0.010071531	2
DHX37	0.010071531	2
EMILIN3	0.010071531	2
GRIK4	0.010071531	2
GSE1	0.010071531	2
GSTK1	0.010071531	2
IPO8	0.010071531	2
LAMA1	0.010071531	2
LMF1	0.010071531	2
MMP9	0.010071531	2
PROCR	0.010071531	2
PYROXD2	0.010071531	2
QSER1	0.010071531	2
SLC3A1	0.010071531	2
SLC4A3	0.010071531	2
TMTC4	0.010071531	2
TNFRSF21	0.010071531	2
USP36	0.010071531	2
USP4	0.010071531	2
GRM2	0.011236843	1
PDCD6IP	0.011323235	1
HSPA4	0.011344528	1
ZNF492	0.011355223	1

SetID	P.value	N.Marker.Test
POLH	0.011398552	1
CUX1	0.011420223	1
SERPINA6	0.011463012	1
NOM1	0.011495616	1
AGAP3	0.011495871	1
MRPL58	0.011734861	1
AQP6	0.011877373	1
STARD10	0.012020778	1
PADI4	0.012121333	3
VWA8	0.01464699	5
ABCB6	0.015195252	5
DNAH10	0.015853355	11
MMUT	0.016169386	4
OBSCN	0.017311945	5
MTUS1	0.018162755	2
DBH	0.018513881	2
TATDN1	0.019494717	1
ABCA9	0.019582711	3
SERPINB13	0.019707154	1
ALG8	0.019744641	1
ASB6	0.019753882	1
CAPZA3	0.019785983	1
GHRL	0.019854165	1
CNTN5	0.019920933	1
GPALPP1	0.020048596	1
C1orf100	0.020146855	1
CERCAM	0.020146855	1
CORO7,CORO7-PA	0.020165985	1
ALS2CL	0.020213926	1
ABHD14A,ABHD14	0.020318509	1
LRRC10	0.020392818	1
MRPL57	0.020396681	1
HPF1	0.020405435	1
SHARPIN	0.020430246	1
ACO2	0.020505845	1
COL16A1	0.020882826	3
GLIS3	0.021505713	2
CCDC9B	0.021573713	2
CNTNAP3	0.021652021	2
FHOD3	0.021765209	2
RAPGEF3	0.022289313	2
BAZ2A	0.022302737	2
PSMA8	0.02284901	2
CAPN13	0.022856099	2
CYB5R3	0.023109817	2
ESRP2	0.023183121	2
THRB	0.023204947	2
AVIL	0.023251015	2
SCN4A	0.023349903	2

SetID	P.value	N.Marker.Test
NSUN4	0.023358506	2
TNFRSF13B	0.023436753	2
GAL3ST4	0.023438168	2
SETD6	0.023454901	2
EPYC	0.023530688	2
KMT2B	0.023613506	2
GJB2	0.023637081	2
KRT40	0.023826711	2
NR2C1	0.023846182	2
PDE6C	0.023852665	2
ATP10D	0.023858759	2
SEC31A	0.023918155	2
GTPBP8	0.024032153	2
B3GNTL1	0.024081955	2
MACC1	0.024128517	2
CGNL1	0.024858764	3
DNAH1	0.024973465	9
KRT20	0.025731114	3
PLA2R1	0.027419297	3
QRSL1	0.027419297	3
HIP1R	0.027419297	3
SIRT2	0.027419297	3
MYH2	0.029175186	6
KRT76	0.029521553	5
AMY2B	0.029747164	2
ARMC3	0.029747164	2
FAHD2A	0.029747164	2
FOCAD	0.029747164	2
GRTP1	0.029747164	2
LDHC	0.029747164	2
LOXHD1	0.029747164	2
MINK1	0.029747164	2
RGPD3	0.029747164	2
SLC27A5	0.029747164	2
STOML3	0.029747164	2
SYNE2	0.029747164	2
GRIN3A	0.030114242	3
IREB2	0.030253526	3
GALNT5	0.0314176	2
MPEG1	0.0315022	2
MYO15A	0.032825732	8
DNAH3	0.032995992	10
SEC23B	0.034577417	5
KCNG4	0.036707316	3
PRUNE2	0.037219812	4
MYH6	0.039237249	5
PKHD1	0.042666432	7
ABCF3	0.043156898	3
LGALS9C	0.043382123	1

SetID	P.value	N.Marker.Test
ERAP2	0.046606511	2
AKR1E2	0.046682711	2
ABCA10	0.047711537	1
ABCC12	0.047711537	1
ABHD12	0.047711537	1
ABHD6	0.047711537	1
ABI1	0.047711537	1
ABL2	0.047711537	1
ACO1	0.047711537	1
ACOT11,FAM151A	0.047711537	1
ACOT6	0.047711537	1
ACSL1	0.047711537	1
ACSL3	0.047711537	1
ACTBL2	0.047711537	1
ADAM19	0.047711537	1
ADAM8	0.047711537	1
ADAMTS10	0.047711537	1
ADAMTSL4	0.047711537	1
ADARB2	0.047711537	1
ADCK1	0.047711537	1
ADCY5	0.047711537	1
ADH4	0.047711537	2
ADH7	0.047711537	1
ADPGK	0.047711537	1
ADRM1	0.047711537	1
AGA	0.047711537	1
38200	0.047711537	1
AGXT2	0.047711537	1
AHSA1	0.047711537	1
AIFM3	0.047711537	1
ALDH7A1	0.047711537	1
ALG10B	0.047711537	1
ALPG	0.047711537	1
ALPK2	0.047711537	1
ALX3	0.047711537	1
AMMECR1L	0.047711537	1
AMN1	0.047711537	1
AMPD3	0.047711537	1
ANGPT4	0.047711537	1
ANGPTL3,DOCK7	0.047711537	1
ANGPTL7,MTOR	0.047711537	1
ANO1	0.047711537	1
APCDD1L	0.047711537	1
APOD	0.047711537	1
ARHGAP22	0.047711537	1
ARHGAP31	0.047711537	1
ARHGAP45	0.047711537	1
ARHGAP9	0.047711537	1
ARID1A	0.047711537	1

SetID	P.value	N.Marker.Test
ARRDC2	0.047711537	1
ASB18	0.047711537	1
ASPN,CENPP	0.047711537	1
ASS1	0.047711537	1
ATP13A5	0.047711537	1
ATP5PB	0.047711537	1
ATP6V1C2	0.047711537	1
AVPR1A	0.047711537	1
B3GALNT2,TBCE	0.047711537	1
B4GALNT3	0.047711537	1
BBS10	0.047711537	1
BBS7	0.047711537	1
BCL7C	0.047711537	1
BIRC2	0.047711537	1
BLM	0.047711537	1
BRD4	0.047711537	1
BRD8	0.047711537	1
BRF1,BTBD6	0.047711537	1
BROX	0.047711537	1
C11orf88	0.047711537	1
C1orf105,PIGC	0.047711537	1
C1orf158	0.047711537	1
C3AR1	0.047711537	1
C3orf20	0.047711537	1
C6orf89	0.047711537	1
C9orf64	0.047711537	1
CA13	0.047711537	1
CACNA2D4,LRTM2	0.047711537	1
CACNB3	0.047711537	1
CALCR	0.047711537	1
CALML4	0.047711537	1
CAPS2	0.047711537	1
CAVIN2	0.047711537	1
CC2D1A	0.047711537	1
CCAR2	0.047711537	1
CCDC134	0.047711537	1
CCDC146	0.047711537	1
CCDC191	0.047711537	1
CCDC59	0.047711537	1
CCDC66	0.047711537	1
CCN1	0.047711537	1
CDC25B	0.047711537	1
CDH19	0.047711537	1
CDH23	0.047711537	1
CDH7	0.047711537	1
CDH9	0.047711537	1
CDHR1	0.047711537	1
CDKL2	0.047711537	1
CELA1	0.047711537	1

SetID	P.value	N.Marker.Test
CENPF	0.047711537	1
CEP112	0.047711537	1
CEP83	0.047711537	1
CEP97	0.047711537	1
CERK	0.047711537	1
CFAP20	0.047711537	1
CFAP65	0.047711537	1
CHAF1B	0.047711537	1
CHI3L2	0.047711537	1
CHMP2B	0.047711537	1
CHRNA10	0.047711537	1
CHRNA2	0.047711537	1
CHRNA5	0.047711537	1
CHSY1	0.047711537	1
CIDEC	0.047711537	1
CKAP4	0.047711537	1
CLASP1	0.047711537	1
CLCA1	0.047711537	1
CLDN10	0.047711537	1
CLDN11	0.047711537	1
CLGN	0.047711537	1
CLMN	0.047711537	1
CLTB	0.047711537	1
CNST	0.047711537	1
CNTN4	0.047711537	1
CNTRL	0.047711537	1
COA8	0.047711537	1
COL18A1	0.047711537	1
COL20A1	0.047711537	1
COL5A1	0.047711537	1
COL9A2	0.047711537	1
CPA1	0.047711537	1
CPD	0.047711537	1
CPO	0.047711537	1
CPSF7	0.047711537	1
CPXM1	0.047711537	1
CREB3L3	0.047711537	1
CRTC3	0.047711537	1
CRYAB	0.047711537	1
CRYBB2	0.047711537	1
CRYGB	0.047711537	1
CSAD	0.047711537	1
CST8	0.047711537	1
CTBS	0.047711537	1
CXCR1	0.047711537	1
CYB5D2	0.047711537	1
CYP24A1	0.047711537	1
DAW1	0.047711537	1
DBN1	0.047711537	1

SetID	P.value	N.Marker.Test
DBT	0.047711537	1
DCDC2B	0.047711537	1
DCHS1	0.047711537	1
DDB2	0.047711537	1
DDIAS	0.047711537	1
DDX42	0.047711537	1
DENND3	0.047711537	1
DEPDC5	0.047711537	1
DEPDC7	0.047711537	1
DGKQ	0.047711537	1
DGLUCY	0.047711537	1
DHX34	0.047711537	1
DHX35	0.047711537	1
DICER1	0.047711537	1
DIP2C	0.047711537	1
DKK1	0.047711537	1
DLAT	0.047711537	1
DLL1	0.047711537	1
DLL3	0.047711537	1
DMRTB1	0.047711537	1
DMWD	0.047711537	1
DNALI1	0.047711537	1
DNM2	0.047711537	1
DOK4	0.047711537	1
DPEP3	0.047711537	1
DPYD	0.047711537	1
DTX2	0.047711537	1
DYNC2H1	0.047711537	1
DZANK1	0.047711537	1
EDEM3	0.047711537	1
EFCAB6	0.047711537	1
EFR3B	0.047711537	1
EIF3A	0.047711537	1
EIF4E1B	0.047711537	1
EIF4G1	0.047711537	1
EIF5B	0.047711537	1
ELMO3	0.047711537	1
ELMOD2	0.047711537	1
ELMOD3	0.047711537	1
ELOVL7	0.047711537	1
EME1	0.047711537	1
EMP1	0.047711537	1
ENAM	0.047711537	1
ENTPD6	0.047711537	1
EPB41L2	0.047711537	1
EPB41L4B	0.047711537	1
EPHX4	0.047711537	1
ESYT1	0.047711537	1
ETFB	0.047711537	1

SetID	P.value	N.Marker.Test
ETFDH	0.047711537	1
EVA1B	0.047711537	1
EXOG	0.047711537	1
EXOSC1	0.047711537	1
EXOSC7	0.047711537	1
EXOSC8	0.047711537	1
EYA2	0.047711537	1
EYS	0.047711537	1
FADS3	0.047711537	1
FAM114A2	0.047711537	1
FAM214A	0.047711537	1
FAM219B	0.047711537	1
FAM53A	0.047711537	1
FAM76B	0.047711537	1
FAM83A	0.047711537	1
FAN1,MTMR10	0.047711537	1
FANCA	0.047711537	1
FANCL	0.047711537	1
FAR2	0.047711537	1
FASLG	0.047711537	1
FBN1	0.047711537	1
FBXL15	0.047711537	1
FBXW10	0.047711537	1
FGFR1	0.047711537	1
FGFR3	0.047711537	1
FOXD3	0.047711537	1
FOXE3	0.047711537	1
FOXO3	0.047711537	1
FOXRED1	0.047711537	1
FPGT-TNNI3K,TNN	0.047711537	1
FRMD3	0.047711537	1
FURIN	0.047711537	1
FUT10	0.047711537	1
FZD5	0.047711537	1
GADD45GIP1	0.047711537	1
GALK1	0.047711537	1
GALNT12	0.047711537	1
GALNT8	0.047711537	1
GATD1	0.047711537	1
GBP7	0.047711537	1
GDAP1L1	0.047711537	1
GDNF	0.047711537	1
GFRA2	0.047711537	1
GLB1L2	0.047711537	1
GPD2	0.047711537	1
GPR152	0.047711537	1
GPR83	0.047711537	1
GPS1	0.047711537	1
GRB14	0.047711537	1

SetID	P.value	N.Marker.Test
GRM4	0.047711537	1
GSDMD	0.047711537	1
GTF3C6	0.047711537	1
GUCY2D	0.047711537	1
HDDC2	0.047711537	1
HGF	0.047711537	1
HK2	0.047711537	1
HKDC1	0.047711537	1
HLCS	0.047711537	1
HMG20A	0.047711537	1
HOPX	0.047711537	1
HOXA10	0.047711537	1
HSPA4L	0.047711537	1
HTR1B	0.047711537	1
HTR3A	0.047711537	1
HTR3B	0.047711537	1
HTR4	0.047711537	1
HUS1	0.047711537	1
HYAL3	0.047711537	1
ICA1	0.047711537	1
ICAM2	0.047711537	1
ID1	0.047711537	1
IDE	0.047711537	1
IDO2	0.047711537	1
IDUA	0.047711537	1
IFFO1	0.047711537	1
IFIT2	0.047711537	1
IFT52	0.047711537	1
IGSF21	0.047711537	1
IL36RN	0.047711537	1
INA	0.047711537	1
INSC	0.047711537	1
INSRR,NTRK1	0.047711537	1
ITGA2	0.047711537	1
ITGB5	0.047711537	1
ITPR2	0.047711537	1
ITPRID2	0.047711537	1
ITPRIP	0.047711537	1
JAK1	0.047711537	1
JARID2	0.047711537	1
JMJD4,SNAP47	0.047711537	1
JPH1	0.047711537	1
JUN	0.047711537	1
KCNAB3	0.047711537	1
KCNB1	0.047711537	1
KCNJ1	0.047711537	1
KCNQ4	0.047711537	1
KIF14	0.047711537	1
KIF22	0.047711537	1

SetID	P.value	N.Marker.Test
KIF9	0.047711537	1
KLHDC8B	0.047711537	1
KLHL36	0.047711537	1
KLHL6	0.047711537	1
KPNA7	0.047711537	1
KRT27	0.047711537	1
KRT32	0.047711537	1
KRT4	0.047711537	1
KYAT1	0.047711537	1
LANCL2	0.047711537	1
LDB3	0.047711537	1
LDLRAD4	0.047711537	1
LFNG	0.047711537	1
LHPP	0.047711537	1
LHX5	0.047711537	1
LIME1,ZGPAT	0.047711537	1
LIPI	0.047711537	1
LMNA	0.047711537	1
LONRF2	0.047711537	1
LPAR3	0.047711537	1
LPIN2	0.047711537	1
LPXN	0.047711537	1
LRP3	0.047711537	1
LRP5	0.047711537	1
LRRFIP2	0.047711537	1
LTC4S	0.047711537	1
LTK	0.047711537	1
LYSMD4	0.047711537	1
MAN2B1	0.047711537	1
MAP2K3	0.047711537	1
MAPK9	0.047711537	1
MBLAC2	0.047711537	1
MFN2	0.047711537	1
MFSD2B	0.047711537	1
MGAT4A	0.047711537	1
MGME1	0.047711537	1
MIEF1	0.047711537	1
MIER2	0.047711537	1
MITF	0.047711537	1
MMACHC	0.047711537	1
MND1	0.047711537	1
MOGAT1	0.047711537	1
MON2	0.047711537	1
MSS51	0.047711537	1
MTOR	0.047711537	1
MYF5	0.047711537	1
MYF6	0.047711537	1
MYO6	0.047711537	1
NAGA	0.047711537	1

SetID	P.value	N.Marker.Test
NAT1	0.047711537	1
NECAB3	0.047711537	1
NEFM	0.047711537	1
NEURL1	0.047711537	1
NFKBID	0.047711537	1
NGLY1	0.047711537	1
NHLRC3	0.047711537	1
NID1	0.047711537	1
NIPAL1	0.047711537	1
NLRP2	0.047711537	1
NMUR1	0.047711537	1
NOP14	0.047711537	1
NOP9	0.047711537	1
NOTUM	0.047711537	1
NOXO1	0.047711537	1
NPM2	0.047711537	1
NR1H2	0.047711537	1
NRP2	0.047711537	1
NT5M	0.047711537	1
NUP153	0.047711537	1
OGFOD2	0.047711537	1
OGFR	0.047711537	1
OR10K1	0.047711537	1
OR1L6	0.047711537	1
OR2W3	0.047711537	1
OR51L1	0.047711537	1
OR52A1	0.047711537	1
OR52K2	0.047711537	1
OR52L1	0.047711537	1
OR5M1	0.047711537	1
OR8B8	0.047711537	1
OTOL1	0.047711537	1
OTUD3	0.047711537	1
OTX2	0.047711537	1
PACRG	0.047711537	1
PAK1IP1	0.047711537	1
PALM	0.047711537	1
PAM	0.047711537	1
PANX1	0.047711537	1
PAQR8	0.047711537	1
PARP3	0.047711537	1
PARP9	0.047711537	1
PARVG	0.047711537	1
PCDHA1,PCDHA2	0.047711537	1
PER3	0.047711537	1
PEX11G	0.047711537	1
PGAP6	0.047711537	1
PHLPP2	0.047711537	1
PIK3R2	0.047711537	1

SetID	P.value	N.Marker.Test
PIK3R3	0.047711537	1
PIK3R5	0.047711537	1
PIWIL3	0.047711537	1
PKNOX1	0.047711537	1
PKP1	0.047711537	1
PLA2G15	0.047711537	1
PLAT	0.047711537	1
PLCG1	0.047711537	1
PLEKHF1	0.047711537	1
PLEKHG1	0.047711537	1
POLE2	0.047711537	1
POLR1B	0.047711537	1
POMT1	0.047711537	1
POT1	0.047711537	1
POU2F2	0.047711537	1
PPHLN1	0.047711537	1
PPP1R15B	0.047711537	1
PRKCE	0.047711537	1
PRLR	0.047711537	1
PRPS1L1	0.047711537	1
PRSS35	0.047711537	1
PRSS48	0.047711537	1
PSMB11	0.047711537	1
PTCH1	0.047711537	1
PTCH2	0.047711537	1
PTP4A2	0.047711537	1
PTPN21	0.047711537	1
PTPN6	0.047711537	1
PTPRG	0.047711537	1
PTPRO	0.047711537	1
PUM3	0.047711537	1
PYCR3	0.047711537	1
QPCTL	0.047711537	1
RABL2A	0.047711537	1
RAD9B	0.047711537	1
RASD1	0.047711537	1
RASSF9	0.047711537	1
RAVER1	0.047711537	1
RB1CC1	0.047711537	1
RBM22	0.047711537	1
RELT	0.047711537	1
RFESD	0.047711537	1
RFLNA,ZNF664-RFI	0.047711537	1
RHBDF1	0.047711537	1
RHOBTB3	0.047711537	1
RNFT1	0.047711537	1
RPA1	0.047711537	1
RPL24	0.047711537	1
RPRM	0.047711537	1

SetID	P.value	N.Marker.Test
RREB1	0.047711537	1
SAG	0.047711537	1
SAMD7	0.047711537	1
SAP18	0.047711537	1
SART3	0.047711537	1
SCAMP2	0.047711537	1
SCAP	0.047711537	1
SCN1A	0.047711537	1
SCN3A	0.047711537	1
SDCBP2	0.047711537	1
SEC14L4	0.047711537	1
SEC14L5	0.047711537	1
SEC23A	0.047711537	1
SEC23IP	0.047711537	2
SELENON	0.047711537	1
SERF2,SERINC4	0.047711537	1
SERPINC1	0.047711537	1
SERPINF1	0.047711537	1
SH2D3A	0.047711537	1
SH3GL3	0.047711537	1
SKOR1	0.047711537	1
SLC15A1	0.047711537	1
SLC17A8	0.047711537	1
SLC19A1	0.047711537	1
SLC25A3	0.047711537	1
SLC25A34	0.047711537	1
SLC25A40	0.047711537	1
SLC29A3	0.047711537	1
SLC29A4	0.047711537	1
SLC2A14	0.047711537	1
SLC31A1	0.047711537	1
SLC34A1	0.047711537	1
SLC34A2	0.047711537	1
SLC35F5	0.047711537	1
SLC6A11	0.047711537	1
SLC9C1	0.047711537	1
SLCO4C1	0.047711537	1
SLF1	0.047711537	1
SLIT2	0.047711537	1
SMOX	0.047711537	1
SMYD1	0.047711537	1
SNX13	0.047711537	1
SORBS2	0.047711537	1
SOX14	0.047711537	1
SOX17	0.047711537	1
SOX6	0.047711537	1
SPDEF	0.047711537	1
SPEG	0.047711537	1
SPRED3	0.047711537	1

SetID	P.value	N.Marker.Test
SQSTM1	0.047711537	1
SRGAP1	0.047711537	1
SRPK1	0.047711537	1
ST3GAL6	0.047711537	1
STARD13	0.047711537	1
STAT2	0.047711537	1
STAU1	0.047711537	1
SUCO	0.047711537	1
SULT2A1	0.047711537	1
SURF1	0.047711537	1
SUSD2	0.047711537	1
SV2C	0.047711537	1
SYNC	0.047711537	1
SYTL2	0.047711537	1
TAF1A	0.047711537	1
TAGAP	0.047711537	1
TBC1D23	0.047711537	1
TBC1D24	0.047711537	1
TBC1D30	0.047711537	1
TCFL5	0.047711537	1
TDRD9	0.047711537	1
TEKT3	0.047711537	1
TERT	0.047711537	1
TGM6	0.047711537	1
THBD	0.047711537	1
THNSL2	0.047711537	1
TICAM1	0.047711537	1
TIE1	0.047711537	1
TLN1	0.047711537	1
TMBIM6	0.047711537	1
TMEM30A	0.047711537	1
TMEM59L	0.047711537	1
TMEM8B	0.047711537	1
TMPRSS11A	0.047711537	1
TNFRSF11A	0.047711537	1
TNFRSF8	0.047711537	1
TNIP1	0.047711537	1
TNKS	0.047711537	1
TNN	0.047711537	1
TOP1MT	0.047711537	1
TPO	0.047711537	1
TPX2	0.047711537	1
TRAPPC11	0.047711537	1
TRARG1	0.047711537	1
TRIM56	0.047711537	1
TRIM72	0.047711537	1
TRIML1	0.047711537	1
TRMO	0.047711537	1
TRPV3	0.047711537	1

SetID	P.value	N.Marker.Test
TRRAP	0.047711537	1
TSC22D2	0.047711537	1
TSEN2	0.047711537	1
TTC14	0.047711537	1
TTC27	0.047711537	1
TTC38	0.047711537	1
TUBG1	0.047711537	1
TVP23A	0.047711537	1
TYW5	0.047711537	1
UBE3D	0.047711537	1
UBQLN4	0.047711537	1
UCP2	0.047711537	1
UFSP2	0.047711537	1
UGT2B7	0.047711537	1
UHRF2	0.047711537	1
UIMC1	0.047711537	1
ULK1	0.047711537	1
UNC45A	0.047711537	1
UNC5C	0.047711537	1
UNC79	0.047711537	1
UPP2	0.047711537	1
USP25	0.047711537	1
VMO1	0.047711537	1
VPS41	0.047711537	1
VSIG10L	0.047711537	1
VSX1	0.047711537	1
WDR35	0.047711537	1
WHRN	0.047711537	1
WNK2	0.047711537	1
XPC	0.047711537	1
YAE1	0.047711537	1
YJU2	0.047711537	1
YKT6	0.047711537	1
ZC3H7B	0.047711537	1
ZCCHC7	0.047711537	1
ZDHHC5	0.047711537	1
ZKSCAN7	0.047711537	1
ZNF143	0.047711537	1
ZNF682	0.047711537	1
ZNF765	0.047711537	1
ZNF775	0.047711537	1
ZNHIT2	0.047711537	1
ZSCAN30	0.047711537	1
MDN1	0.04929098	3
SBF1	0.049748317	3
PITRM1	0.052769071	5
TSTD2	0.05547977	4
COL4A3	0.059774935	4
MYH7	0.060380396	3

SetID	P.value	N.Marker.Test
FGG	0.064661391	2
CCM2	0.07156238	3
CD109	0.07156238	3
DDX59	0.07156238	3
DNER	0.07156238	3
HAL	0.07156238	3
KIF17	0.07156238	3
RB1	0.07156238	3
ADCY7	0.07156238	3
NRXN1	0.07156238	3
CSMD1	0.07156238	3
PTPRB	0.074760931	3
SARDH	0.075352028	4
COL5A3	0.075356659	4
TRIP11	0.076234122	3
ACADVL	0.086007844	3
SNCAIP	0.086007844	3
NPC1L1	0.086007844	3
TBCK	0.086007844	3
CD36	0.086007844	3
MFSD12	0.086007844	3
MYH1	0.086213677	4
LCTL	0.088392561	2
TP73	0.09082538	6
SAMD9L	0.090831167	2
COL6A3	0.091976703	10
CUBN	0.09968271	4
PKD1	0.103090571	7
DOCK8	0.104772591	4
ITIH2	0.104986341	3
IMP4	0.109004418	2
NR1I2	0.109056181	2
KRT75	0.10933707	2
SCIN	0.109545095	2
NOTCH1	0.109830721	3
SCRN2	0.109901154	2
GPR151	0.110147368	2
EPX	0.11039219	2
SACS	0.110646502	6
MYCBPAP	0.110771064	2
HMCN1	0.118291868	5
PCDH15	0.118772935	5
ADAMTS15	0.121082963	4
ATP10B	0.121082963	4
IQUB	0.121082963	4
SPTAN1	0.121082963	4
CYP4F3	0.129013237	3
MFSD9	0.129841001	2
CAPN15	0.132030661	4

SetID	P.value	N.Marker.Test
ABAT	0.139599054	2
ASCC3	0.139599054	2
ASNSD1	0.139599054	2
BIRC6	0.139599054	2
COG3	0.139599054	2
COL1A1	0.139599054	2
EDRF1	0.139599054	2
FARSA	0.139599054	2
FNDC1	0.139599054	2
GUCA1C	0.139599054	2
ITPR3	0.139599054	2
KMT2E	0.139599054	2
MICAL3	0.139599054	2
RFX4	0.139599054	2
TELO2	0.139599054	2
UBAP2	0.139599054	2
ACAD11	0.139599054	2
ARHGEF12	0.139599054	2
BHMT	0.139599054	2
CBLB	0.139599054	2
ELFN2	0.139599054	2
ERCC1	0.139599054	2
FCHSD1	0.139599054	2
HFM1	0.139599054	2
LHFPL5	0.139599054	2
LRP12	0.139599054	2
LRP5L	0.139599054	2
MGAT5B	0.139599054	2
MMP10	0.139599054	2
MRPS27	0.139599054	2
MRTFB	0.139599054	2
NEGR1	0.139599054	2
NOS3	0.139599054	2
PCDHA1,PCDHA2,[illegible]	0.139599054	2
PDHX	0.139599054	2
PGM5	0.139599054	2
POLM	0.139599054	2
SEPTIN10	0.139599054	2
SERPINA3	0.139599054	2
SLC25A47	0.139599054	2
SLCO1C1	0.139599054	2
TBC1D13	0.139599054	2
ZBTB46	0.139599054	2
ACSL5	0.139599054	2
ADAT1	0.139599054	2
DHX33	0.147323146	4
FBN2	0.150078676	4
ABCC6	0.156302052	5
ARHGAP11A	0.158421321	6

SetID	P.value	N.Marker.Test
CDC42BPG	0.159121281	4
COL6A5	0.176193517	7
PGK2	0.177363087	3
LAMC1	0.177781946	3
EPB41L4A	0.177782123	3
NT5DC2	0.178154992	3
LAMA3	0.178174803	3
TTC30B	0.178275831	3
SPTBN2	0.178292895	5
HERC2	0.178494044	3
SPG11	0.178558785	3
CEMIP2	0.17858259	3
FLNC	0.178747674	4
LRRD1	0.178761411	3
KCNT1	0.178816092	3
SEMA5A	0.178850721	3
CNGA3	0.179019199	3
GLI3	0.179080135	3
COL2A1	0.179152982	3
PDCD11	0.179344172	3
UVSSA	0.179400809	3
DOP1B	0.179610118	3
DAGLB	0.181438441	4
ABCA7	0.182859182	1
ABCC8	0.182859182	1
ABHD12B	0.182859182	1
ACBD3	0.182859182	1
ACRV1	0.182859182	1
ACTN1	0.182859182	1
ACVR2B	0.182859182	1
ADAM22	0.182859182	1
ADAMTS18	0.182859182	1
ADAMTS2	0.182859182	1
ADAR	0.182859182	1
ADCY1	0.182859182	1
ADGRG5	0.182859182	1
ADIPOR2	0.182859182	1
AKAP2,PALM2AKA	0.182859182	1
ALDH9A1	0.182859182	1
ANAPC4	0.182859182	1
ANLN	0.182859182	1
AP3M1	0.182859182	1
APBB3	0.182859182	1
ARAP1	0.182859182	1
ARFGEF3	0.182859182	1
ARHGAP20	0.182859182	1
ARHGEF10L	0.182859182	1
ARHGEF38	0.182859182	1
ARHGEF4	0.182859182	1

SetID	P.value	N.Marker.Test
ARID2	0.182859182	1
ASPG	0.182859182	1
ASPHD2	0.182859182	1
ATG16L2	0.182859182	1
ATOH1	0.182859182	1
BBS1	0.182859182	1
BCAR3	0.182859182	1
BCKDHB	0.182859182	1
BRIP1	0.182859182	1
BSCL2	0.182859182	1
C11orf54	0.182859182	1
C16orf70	0.182859182	1
C19orf71	0.182859182	1
C1orf94	0.182859182	1
C1R	0.182859182	1
C7	0.182859182	1
CACNA2D2	0.182859182	1
CAPN8	0.182859182	1
CAPNS2,LPCAT2	0.182859182	1
CARMIL1	0.182859182	1
CASC1	0.182859182	1
CBWD1	0.182859182	1
CCDC113	0.182859182	1
CCDC121	0.182859182	1
CDC42BPB	0.182859182	1
CDC7	0.182859182	1
CDCA5	0.182859182	1
CDCP2	0.182859182	1
CDT1	0.182859182	1
CELA2A	0.182859182	1
CEMIP	0.182859182	1
CHIT1	0.182859182	1
CKM	0.182859182	1
CKMT2	0.182859182	1
CLEC18B	0.182859182	1
CLN6	0.182859182	1
CMAS	0.182859182	1
COL6A1	0.182859182	1
COLEC12	0.182859182	1
COQ4	0.182859182	1
COX4I2	0.182859182	1
CPA5	0.182859182	1
CPSF4L	0.182859182	1
CREM	0.182859182	1
CRISP3	0.182859182	1
CRTAC1	0.182859182	1
CRYGA	0.182859182	1
CTRL	0.182859182	1
DCAF11	0.182859182	1

SetID	P.value	N.Marker.Test
DCAF4L2	0.182859182	1
DDX10	0.182859182	1
DERA	0.182859182	1
DEUP1	0.182859182	1
DGKA	0.182859182	1
DHDH	0.182859182	2
DLGAP4	0.182859182	1
DMGDH	0.182859182	1
DPH2	0.182859182	1
DSG4	0.182859182	1
DUSP13	0.182859182	1
EIF3B	0.182859182	1
EIF4ENIF1	0.182859182	1
EIF5AL1	0.182859182	1
ELFN1	0.182859182	1
ELL2	0.182859182	1
EMP2	0.182859182	1
ESS2	0.182859182	1
EYA3	0.182859182	1
F7	0.182859182	1
FAM126B	0.182859182	1
FAM174B	0.182859182	1
FAM83H	0.182859182	1
FBRSL1	0.182859182	1
FCHO1	0.182859182	1
FDFT1	0.182859182	1
FRRS1	0.182859182	1
GALNT10	0.182859182	1
GALR2	0.182859182	1
GATA4	0.182859182	1
GDI2	0.182859182	1
GDPGP1	0.182859182	1
GGTLC1	0.182859182	1
GJA8	0.182859182	1
GLYR1	0.182859182	1
GNRHR	0.182859182	1
GPATCH11	0.182859182	1
GPATCH4	0.182859182	1
GPR142	0.182859182	1
GPR158	0.182859182	1
GPR162	0.182859182	1
GRB10	0.182859182	1
GRID1	0.182859182	1
GUCY1A2	0.182859182	1
GZMH	0.182859182	1
HCAR3	0.182859182	1
HDAC10	0.182859182	1
HIVEP3	0.182859182	1
HOXC10	0.182859182	1

SetID	P.value	N.Marker.Test
HS1BP3	0.182859182	1
HSD17B1	0.182859182	1
HSF4	0.182859182	1
IL1RL1	0.182859182	1
INSYN1	0.182859182	1
IP6K3	0.182859182	1
ISLR	0.182859182	1
ITGB4	0.182859182	1
ITPKA	0.182859182	1
JAG1	0.182859182	1
KCNQ1	0.182859182	1
KCNS3	0.182859182	1
KCTD13	0.182859182	1
KIF20A	0.182859182	1
KLF17	0.182859182	1
KLHDC1	0.182859182	1
KLHL3	0.182859182	1
KLK12	0.182859182	1
KRTAP4-4	0.182859182	1
LAMA4	0.182859182	1
LAMB1	0.182859182	1
LEMD2	0.182859182	1
LGALS1	0.182859182	1
LHX3	0.182859182	1
LIPT2	0.182859182	1
LRPAP1	0.182859182	1
LRRC14B	0.182859182	1
LRRC49	0.182859182	1
LTB4R	0.182859182	1
LTV1	0.182859182	1
MAGI2	0.182859182	1
MAML2	0.182859182	1
MAN2A1	0.182859182	1
MAST1	0.182859182	1
MBD5	0.182859182	1
MCM5	0.182859182	1
MCOLN2	0.182859182	1
MDFIC	0.182859182	1
MED12L	0.182859182	1
MED19	0.182859182	1
MEF2A	0.182859182	1
MFSD5	0.182859182	1
MIER3	0.182859182	1
MIP	0.182859182	1
MLYCD	0.182859182	1
MMS19	0.182859182	1
MRPL11	0.182859182	1
MRPL48	0.182859182	1
MRPL52	0.182859182	1

SetID	P.value	N.Marker.Test
MTCH2	0.182859182	1
MTG1	0.182859182	1
MZB1	0.182859182	1
NAGPA	0.182859182	1
NBR1	0.182859182	1
NDUFAF4	0.182859182	1
NEBL	0.182859182	1
NEUROD4	0.182859182	1
NINL	0.182859182	1
NMNAT1	0.182859182	1
NOL10	0.182859182	1
NOSIP	0.182859182	1
NQO1	0.182859182	1
NSL1	0.182859182	1
NTHL1	0.182859182	1
NTSR1	0.182859182	1
NUP205	0.182859182	1
NUP98	0.182859182	1
NXN	0.182859182	1
NXPE1	0.182859182	1
OPN3	0.182859182	1
OR10G3	0.182859182	1
OR10G8	0.182859182	1
OR14I1	0.182859182	1
OR51D1	0.182859182	1
OR51G2	0.182859182	1
OR5F1	0.182859182	1
OR5P3	0.182859182	1
OR8D1	0.182859182	1
OTOG	0.182859182	1
PARD6G	0.182859182	1
PATL2	0.182859182	1
PC	0.182859182	1
PCDH1	0.182859182	1
PCDHA1,PCDHA2,I	0.182859182	1
PCSK1	0.182859182	1
PDCD2	0.182859182	1
PDE5A	0.182859182	1
PEMT	0.182859182	1
PES1	0.182859182	1
PHF12	0.182859182	1
PIGV	0.182859182	1
PKN1	0.182859182	1
PLEKHB1	0.182859182	1
PLPP2	0.182859182	1
PLPPR5	0.182859182	1
PNPLA6	0.182859182	1
POU3F2	0.182859182	1
POU5F1B	0.182859182	1

SetID	P.value	N.Marker.Test
PPIC	0.182859182	1
PRDX3	0.182859182	1
PREP	0.182859182	1
PREPL,SLC3A1	0.182859182	1
PRF1	0.182859182	1
PSD4	0.182859182	1
PSMC1	0.182859182	1
PTCHD4	0.182859182	1
PTGFR	0.182859182	1
PTK7	0.182859182	1
PUS7L	0.182859182	1
PXK	0.182859182	1
RAD18	0.182859182	1
RALGAPA1	0.182859182	1
RANGAP1	0.182859182	1
RASGEF1A	0.182859182	1
RBM26	0.182859182	1
REEP6	0.182859182	1
RERGL	0.182859182	1
RFPL4A	0.182859182	1
RIC8A	0.182859182	1
RIT2	0.182859182	1
RNF10	0.182859182	1
RNF157	0.182859182	1
RTN3	0.182859182	1
SCFD2	0.182859182	1
SDHAF4	0.182859182	1
SETX	0.182859182	1
SFMBT2	0.182859182	1
SH3TC2	0.182859182	1
SHLD2	0.182859182	1
SHPRH	0.182859182	1
SHROOM3	0.182859182	1
SIDT2	0.182859182	1
SLC13A2	0.182859182	1
SLC25A23	0.182859182	1
SLC26A11	0.182859182	1
SLC28A1	0.182859182	1
SLC2A5	0.182859182	1
SLC38A7	0.182859182	1
SLC9A4	0.182859182	1
SLC9A9	0.182859182	1
SLCO5A1	0.182859182	1
SNAPC3	0.182859182	1
SNX6	0.182859182	1
SORCS2	0.182859182	1
SOX13	0.182859182	1
SPARCL1	0.182859182	1
STAC	0.182859182	1

SetID	P.value	N.Marker.Test
STAC2	0.182859182	1
STARD9	0.182859182	1
STAT4	0.182859182	1
SULT1A2	0.182859182	1
SULT6B1	0.182859182	1
TAS1R1	0.182859182	1
TAS2R8	0.182859182	1
TBC1D7,TBC1D7-L(	0.182859182	1
TBX6	0.182859182	1
TEPSIN	0.182859182	1
THBS4	0.182859182	1
THSD1	0.182859182	1
TM6SF2	0.182859182	1
TMC6	0.182859182	1
TMEM163	0.182859182	1
TMEM243	0.182859182	1
TMEM62	0.182859182	1
TNRC6A	0.182859182	1
TRIM5	0.182859182	1
TRPC7	0.182859182	1
TRPV5	0.182859182	1
TSC2	0.182859182	1
TTC24	0.182859182	1
TTLL11	0.182859182	1
TUBE1	0.182859182	1
UBR1	0.182859182	1
UGT1A1,UGT1A3,U	0.182859182	1
UHRF1BP1L	0.182859182	1
VWDE	0.182859182	1
WDFY4	0.182859182	1
WDR74	0.182859182	1
XKR9	0.182859182	1
XPO4	0.182859182	1
XRRA1	0.182859182	1
XYLT2	0.182859182	1
ZC3H12A	0.182859182	1
ZDHHC23	0.182859182	1
ZNF215	0.182859182	1
ZNF540	0.182859182	1
ZNF875	0.182859182	1
ZPR1	0.182859182	1
COL15A1	0.184707464	4
TCF3	0.185189307	4
CTU2	0.185230618	4
KRT34	0.185655576	2
RNF213	0.187858117	4
TAF1L	0.189216027	4
ALPP	0.190486652	3
SPEF2	0.190530253	5

SetID	P.value	N.Marker.Test
DNAH5	0.193078744	5
GPRC6A	0.196559194	3
NCKIPSD	0.206605972	4
SLC12A1	0.206605972	4
TRIB3	0.206605972	4
CDHR2	0.215568933	4
FMNL2	0.21589828	2
HPD	0.217018556	2
MYH11,NDE1	0.217125772	4
SLC7A8	0.217304498	2
SLC16A11	0.217522829	2
MLH3	0.217736908	2
DCC	0.218290353	2
ESCO1	0.21853439	2
UNC45B	0.22059307	5
EFCAB5	0.221175993	2
MYH13	0.221562066	3
MYO7B	0.22666467	5
FUT3	0.227422495	2
KMT2A	0.230318575	3
APC2	0.232215873	3
ACLY	0.233462776	4
ADGRL2	0.234364311	4
GNAT3	0.234572744	2
AGXT	0.23478459	3
MCM4	0.234870297	2
TSR1	0.234899075	3
TJP1	0.235036184	3
TDO2	0.236465627	2
COL17A1	0.237074709	4
IFT122	0.237763847	3
FOXM1	0.237891696	3
USH2A	0.237942214	4
C5orf34	0.238382048	4
MYH4	0.238831465	4
XIRP2	0.2389704	3
CASP6	0.239647872	2
DUSP27	0.239767659	3
PLXNA2	0.239894803	4
CAPN12	0.240785387	2
TMEM255B	0.244429943	2
ZFYVE16	0.244624538	2
AATK	0.245136388	2
NALCN	0.245436883	2
PNKD,TMBIM1	0.245518024	4
TMC5	0.245558086	2
ACAD9	0.253486245	3
FAM234A	0.253656143	3
TOP3B	0.254801361	3

SetID	P.value	N.Marker.Test
PLEKHG2	0.254886392	3
PCARE	0.255001786	3
ASCC2	0.255618235	3
LMNB1	0.255672475	3
NHSL1	0.255955279	3
EP300	0.257503016	3
KIF26A	0.258221896	6
DAAM2	0.25887852	3
FGL1	0.267561239	2
MYO10	0.267981344	4
CFAP57	0.268673931	3
MEGF11	0.268673931	3
CCM2L	0.272182871	2
GJB4	0.273658739	2
UGGT1	0.274022464	2
MYBBP1A	0.274092113	2
GNA14	0.274792262	3
RNPEP	0.275044904	2
NMBR	0.275989786	2
TAF1C	0.277032719	2
PDHA2	0.277077938	2
ABCC11	0.277714916	4
EVC2	0.277963024	2
NRP1	0.278022941	3
EFS	0.278432187	2
PPL	0.280221811	4
CNGB1	0.281296034	4
BLK	0.283256204	4
CROCC	0.284601006	4
NEB	0.287267336	10
PDE6A	0.288634494	4
BCO2	0.291430669	4
SYNJ2	0.291656595	2
LAMA5	0.295839471	10
MTMR4	0.297694375	4
FAM86B2	0.301779971	1
ABHD10	0.305515092	2
ABRAXAS1	0.305515092	2
ALX1	0.305515092	2
CILP	0.305515092	2
CTNS	0.305515092	2
DNMT3A	0.305515092	2
KNDC1	0.305515092	2
MPDZ	0.305515092	2
MYOM2	0.305515092	2
NCBP1	0.305515092	2
NID2	0.305515092	2
SLC35E2A	0.305515092	2
SLC5A9	0.305515092	2

SetID	P.value	N.Marker.Test
TCF7L1	0.305515092	2
TSEN54	0.305515092	2
UBXN4	0.305515092	2
ZNF217	0.305515092	2
AARS2	0.305515092	2
ABCA3	0.305515092	2
ABCD4	0.305515092	2
ABCG2	0.305515092	2
ABLIM2	0.305515092	2
ACAA1	0.305515092	2
ACOX2	0.305515092	2
ACSM5	0.305515092	2
ADA	0.305515092	2
AMBN	0.305515092	2
B3GALNT1	0.305515092	2
BRPF3	0.305515092	2
C4orf47	0.305515092	2
CACNA1H	0.305515092	2
CLUH	0.305515092	2
COL1A2	0.305515092	2
COL9A3	0.305515092	2
DHCR24	0.305515092	2
DMBX1	0.305515092	2
DNAJC21	0.305515092	2
ECHDC1	0.305515092	2
EVPL	0.305515092	2
EVX1	0.305515092	2
GRIK1	0.305515092	2
HACD1	0.305515092	2
HAUS5	0.305515092	2
HIP1	0.305515092	2
HS3ST6	0.305515092	2
ITGA2B	0.305515092	2
LARGE2	0.305515092	2
MPZL2	0.305515092	2
NAALAD2	0.305515092	2
NCAPD2	0.305515092	2
OR13A1	0.305515092	2
PMS2	0.305515092	2
RECQL	0.305515092	2
RGL3	0.305515092	2
RNASEH2B	0.305515092	2
SLC10A2	0.305515092	2
SLC7A2	0.305515092	2
SPIRE2	0.305515092	2
STAR	0.305515092	2
TEC	0.305515092	2
TFR2	0.305515092	2
TGM3	0.305515092	2

SetID	P.value	N.Marker.Test
TUBA8	0.305515092	2
WNT10A	0.305515092	2
ZFR2	0.305515092	2
ACSM3,ERI2	0.305515092	2
ACTL7B	0.305515092	2
ADAMTS12	0.305515092	2
ADAMTS20	0.305515092	2
ARHGEF16	0.305515092	2
ATM,C11orf65	0.305515092	2
BOLA1	0.305515092	2
CFAP58	0.305515092	2
CNGA4	0.305515092	2
COL11A1	0.305515092	2
DMBT1	0.305515092	2
DNAJB1	0.305515092	2
ELN	0.305515092	2
FBLN1	0.305515092	2
FTSJ3	0.305515092	2
HCN4	0.305515092	2
HSDL2	0.305515092	2
KRT12	0.305515092	2
MAB21L1,NBEA	0.305515092	2
PHYHD1	0.305515092	2
PTPRS	0.305515092	2
RIPK4	0.305515092	2
RIPOR3	0.305515092	2
SBF2	0.305515092	2
SLC26A2	0.305515092	2
SMAD9	0.305515092	2
SPAG6	0.305515092	2
TARS3	0.305515092	2
TEX9	0.305515092	2
TMCO4	0.305515092	2
TMIGD1	0.305515092	2
ALDH1L2	0.305515092	2
ALOX12	0.305515092	2
AOX1	0.305515092	2
ATP8B4	0.305515092	2
C5	0.305515092	2
CDH12	0.305515092	2
CHD6	0.305515092	2
CHST10	0.305515092	2
CLCN2	0.305515092	2
DIAPH3	0.305515092	2
DNAAF4	0.305515092	2
DPYSL5	0.305515092	2
FAM228B,PFN4	0.305515092	2
FASN	0.305515092	2
FOXQ1	0.305515092	2

SetID	P.value	N.Marker.Test
GALC	0.305515092	2
GCM2	0.305515092	2
GTPBP4	0.305515092	2
HTT	0.305515092	2
ITGB3	0.305515092	2
LLGL1	0.305515092	2
LNX1	0.305515092	2
LYVE1	0.305515092	2
MARCHF10	0.305515092	2
MPO	0.305515092	2
MTSS2	0.305515092	2
MYL5	0.305515092	2
NCAPG	0.305515092	2
NSFL1C	0.305515092	2
PCDHB7	0.305515092	2
PDZRN4	0.305515092	2
PYGB	0.305515092	2
RAD54L2	0.305515092	2
SCARA3	0.305515092	2
SCML4	0.305515092	2
SCTR	0.305515092	2
SLC35B4	0.305515092	2
TLCD3A	0.305515092	2
WDR1	0.305515092	2
ZFYVE28	0.305515092	2
ZMYND10	0.305515092	2
ZNF451	0.305515092	2
ZNF816,ZNF816-ZI	0.305515092	2
ATP6V0D2	0.305515092	2
GALNS	0.305515092	2
MSANTD1	0.305515092	2
NLE1	0.305515092	2
SLC25A19	0.305515092	2
CCDC39,TTC14	0.307197496	1
DPP7	0.307197496	1
GALNT15	0.307197496	1
PCDHA1,PCDHA2,I	0.307197496	1
RNASEL	0.307197496	1
SDAD1	0.307197496	1
SDHD	0.307197496	1
TTLL4	0.307197496	1
ZNF846	0.307197496	1
PCDH12	0.309701213	4
SERPINI2	0.310949274	2
NEK1	0.312722716	2
ONECUT1	0.313789158	2
DCLRE1C	0.314385377	2
ATAD3A	0.314546979	2
DOCK10	0.316310749	2

SetID	P.value	N.Marker.Test
VIL1	0.316746892	2
CFAP206	0.317113128	2
FBXO36	0.317363493	2
PLEC	0.318179022	8
SCNN1A	0.320250452	4
PCDHB11	0.324187371	3
CFAP45	0.324653325	3
WDR6	0.324801524	3
EXOC1	0.325842255	3
TNS3	0.325914582	3
ATP8B3	0.326104983	3
CSMD2	0.326228852	3
EFCAB7	0.326589462	3
SYTL3	0.326777057	3
RBM20	0.326915673	3
MAML1	0.326953069	3
CLTCL1	0.327099357	5
PIGQ	0.327577343	3
NDOR1	0.327654728	3
PYGM	0.327746471	3
MRI1	0.327810628	3
SLC34A3	0.327815955	3
MKRN2	0.328098744	3
UBA7	0.328470698	3
RBM19	0.328565075	3
CPT1A	0.328896663	3
MFN1	0.329031764	3
TSTA3	0.329079254	3
NUBP1	0.329719581	3
NEFH	0.330840953	3
PLCD4	0.33110041	3
IDUA,SLC26A1	0.346571203	3
LAMA2	0.349544596	3
EP400	0.351301927	4
WFS1	0.352903254	5
DYSF	0.352968194	7
HADH	0.357448931	2
ABCA8	0.359139211	3
SLIT3	0.361128333	3
MGAM	0.361813893	3
WASHC2C	0.36211859	2
SLC4A5	0.362719654	4
FGD2	0.36290896	2
PHKB	0.362976103	2
WASHC5	0.365417757	2
WASHC4	0.365561424	2
JAG2	0.366710184	2
CLDN3	0.367323097	2
BBS12	0.367762114	2

SetID	P.value	N.Marker.Test
EGFLAM	0.368054083	2
SCNN1B	0.368068208	3
SRMS	0.36824324	2
PEX12	0.368376323	2
KRT74	0.368717111	3
DMXL1	0.369897618	2
IFT172	0.370238881	2
ABCA1	0.370654962	2
KIF16B	0.371105794	2
CHGA	0.371717574	2
PIGU	0.372138455	2
PARP4	0.372994584	2
CTC1	0.373306213	2
ADAMTS16	0.373321286	2
SPNS1	0.374000632	2
GCNT1	0.374728676	2
IL6ST	0.37483696	3
NXPE4	0.375426414	1
MXRA8	0.375979968	1
CALHM5,TRAPPC3	0.377202375	1
CHAT	0.377316712	1
SLC16A12	0.377374119	1
PLA2G2D	0.377388195	1
ACSM1	0.378227552	1
HARS1	0.378350562	1
FMO4	0.378694581	1
CAD	0.378773311	3
NDNF	0.378875207	1
STC2	0.379033291	1
OGG1	0.379413462	1
MAVS	0.379524962	1
SHISA6	0.379627803	1
TMEM126B	0.379666109	1
CENPP,OMD	0.379707909	1
SULT2B1	0.379846203	1
ANKLE2	0.380109524	1
NR3C1	0.380169385	1
ABCF2	0.380610547	1
NFX1	0.38069036	1
FGD4	0.380693122	1
GANAB	0.380727688	1
C15orf39	0.380886363	1
AZIN2	0.380960451	3
KMT2C	0.381062229	6
C1QBP	0.381063926	1
CYP2U1	0.381145012	1
CCDC87	0.381238914	1
MRPL43,SEMA4G	0.381242887	1
ACAN	0.381277235	1

SetID	P.value	N.Marker.Test
GABRA6	0.381354579	1
SLC38A11	0.381493365	1
LRRN4	0.381498957	1
NUDT22	0.381511732	1
MMRN1	0.381768809	3
OR1F1	0.381799179	1
SELENOO	0.38183369	1
FAM124A	0.382020872	1
ZZEF1	0.382233096	1
HS3ST5	0.382704556	1
OR8B12	0.382720881	1
DOK7	0.38290913	3
P2RX4	0.383047697	3
PYGL	0.383383163	3
NCKAP1	0.383511085	1
CFAP298	0.383617518	1
GBGT1	0.383772389	3
SUPT4H1	0.383776498	1
CD101	0.383829428	3
SEMA3F	0.383836282	3
OCA2	0.384166912	3
MIOX	0.384188609	1
CYP2D6	0.384279398	3
SLIT1	0.384340221	1
SCAPER	0.384394641	1
OLAH	0.384448423	1
PGAM2	0.384460505	1
CNGB3	0.384970494	3
MYO5B	0.385030239	3
LAMB4	0.385045333	1
EGFR	0.385142709	3
CAPN5	0.385232537	3
KCNH6	0.385447456	3
.	0.385652038	3
SPATA5	0.386050191	3
COL4A4	0.386141714	3
EPHA6	0.386488016	3
CTNNA3	0.386953503	3
PTPN14	0.387118836	3
ESPNL	0.387212065	3
IL20	0.387401609	1
MMEL1	0.387451327	3
PFKL	0.387755234	3
TBC1D4	0.387794947	3
MYO1D	0.387970046	2
DPY19L4	0.388220689	3
PMS1	0.388669277	3
ADGRG6	0.389525698	3
HSPG2	0.390580051	3

SetID	P.value	N.Marker.Test
GFAP	0.390890085	3
SLC26A10	0.391703066	3
LRP6	0.391851765	3
MELTF	0.3923151	3
AARS1	0.393855158	3
ENPP3	0.39508396	4
PPFIBP1	0.395241122	4
VWF	0.396048237	4
OR51B5,OR52D1	0.405157911	4
CAPN3	0.406177214	1
CATSPERB	0.407668084	1
DPP10	0.407853	1
MCM9	0.407856586	1
CFTR	0.409032853	7
BTD	0.409510431	1
MOV10L1	0.410051956	1
ECM1	0.410955212	1
CP	0.410962131	1
SSH1	0.411680849	1
PCNX2	0.411688768	4
RPL3L	0.412395374	1
OR2A14	0.413101391	1
SLC46A3	0.413636143	1
BMPR1B	0.413809021	1
PALMD	0.413982892	1
TNFAIP1	0.413986114	1
FAM83C	0.414157611	1
PLOD1	0.414684879	1
PTGIR	0.414686021	1
LRIT1	0.415035039	1
CEP104	0.415208856	1
CRAT	0.415210768	1
NARF	0.415384801	1
SLC4A11	0.415601269	2
STK36	0.415676826	2
LRRN1	0.41572585	1
ESD	0.416070184	1
ZNF684	0.416070184	1
COMMD8	0.416241215	1
CRELD1	0.416417202	1
MYG1	0.416762303	1
PCDHB2	0.416926036	1
RIC8B	0.416929301	1
TVP23C,TVP23C-C	0.416934204	1
METAP1D	0.41709496	1
FAM189A1	0.417098808	1
ACAD8	0.417101339	1
CSMD3	0.417268081	1
DEFB119	0.417276557	1

SetID	P.value	N.Marker.Test
ZP3	0.417775331	1
HEATR3	0.417780855	1
METTL11B	0.417949965	1
OCIAD2	0.417950832	1
GUCY1A1	0.41795451	1
SH3GL1	0.418120187	1
IMPG2	0.418123133	1
MEP1B	0.41829296	1
KCTD20	0.418461136	1
UBQLN1	0.418462842	1
SIM2	0.418624283	1
DDX1	0.41863106	1
KRT16	0.418797966	1
IFT46	0.418967283	1
ATRN	0.419134021	1
DMRT2	0.419299535	1
ADGRF1	0.419462234	1
KDM8	0.41946777	1
TGM7	0.419474092	1
LIMCH1	0.419632105	1
NUBPL	0.419634524	1
CYP2A6	0.419804092	1
HAVCR1	0.420131701	1
ST6GALNAC2	0.420137339	1
WNK4	0.420137819	1
CDK18	0.420297639	1
CMKLR1	0.420569209	2
IL1F10	0.420629286	1
IFT140	0.420630939	1
NEDD4	0.420631119	1
CYP2B6	0.42079798	1
HSD3B1	0.420799718	1
FCRLA	0.421125372	1
GALNTL5	0.421265324	2
VCAN	0.421266628	2
SYNE3	0.421622527	1
SPATA18	0.421788313	1
CPS1	0.421947974	1
FKBP9	0.422114391	1
KAT2B	0.422116987	1
OLFML1	0.422275226	1
MCEE	0.422435918	2
HTRA3	0.422441759	1
PACRGL	0.422444873	1
PDCL3	0.422597254	1
CCDC74B	0.422770779	1
LATS2	0.422927771	1
CHURC1-FNTB,GP)	0.423247701	1
DHRS9	0.423575587	1

SetID	P.value	N.Marker.Test
NDUFA10	0.423896953	1
ABCG5	0.424048108	2
CC2D2A	0.424048108	2
CLSTN2	0.424048108	2
ERC2	0.424048108	2
FRMD1	0.424048108	2
LPIN1	0.424048108	2
TMEM104	0.424048108	2
ODF3L2	0.424048108	2
KHK	0.424048108	2
LHX6	0.424048108	2
MYO1E	0.424048108	2
PLTP	0.424048108	2
PRMT7	0.424048108	2
ZEB1	0.424048108	2
ANKRD16	0.424378387	1
RALGAPB	0.424536619	1
ADCY6	0.424700509	1
RIC1	0.424861851	1
GAA	0.425175878	1
SIGMAR1	0.425492972	1
CA14	0.425810425	1
FN3KRP	0.427066577	1
COQ7	0.42707173	1
COL28A1	0.428302967	6
TG	0.428931261	4
ACAD10	0.430141123	7
LPA	0.431280869	3
COG1	0.435005185	3
CNDP2	0.435271586	2
KLK14	0.435750808	3
TTC21A	0.438098588	3
AGBL3	0.438282972	3
CPXM2	0.438282972	3
MCTP2	0.438282972	3
NRG2	0.438282972	3
ALDH1B1	0.438282972	3
CELSR1	0.438282972	3
CKAP2	0.438282972	3
GCDH	0.438282972	3
JMJD1C	0.438282972	3
MYO1H	0.438282972	3
OSBPL5	0.438282972	3
SEC31B	0.438282972	3
SLC8A3	0.438282972	3
TMEM184A	0.438282972	3
CPLANE1	0.438282972	3
DNA2	0.438282972	3
DOK2	0.438282972	3

SetID	P.value	N.Marker.Test
NCOR1	0.438282972	3
SMYD5	0.438282972	3
SLC6A18	0.438294898	2
RASGRP4	0.438696581	3
MYO9B	0.44063987	3
LMOD2	0.44543416	2
SLC5A10	0.445610455	2
LRBA	0.454984006	5
PFAS	0.455824439	4
NPHP4	0.458167406	5
MC1R	0.458407777	4
PADI3	0.4668607	3
CGN	0.468456998	7
TTN	0.473006663	31
DNAH6	0.473123368	6
AKAP11	0.473612538	4
OR4L1	0.483490535	1
GCFC2	0.484897511	1
KHNYN,SDR39U1	0.48551951	1
ARPP21	0.486134519	1
R3HDM1	0.486135472	1
PCDHA1,PCDHA2,I	0.486209751	1
CYP4V2	0.486898774	1
TICRR	0.487812591	1
POLG2	0.488116796	1
LZTS1	0.488120334	1
MOS	0.48826835	1
SLC3A2	0.488720406	1
MRPL51	0.488722835	1
PTBP3	0.488869023	1
TMEM43	0.489768259	1
EPHA10	0.489769314	1
HOXC12	0.489769314	1
TMEM266	0.489918494	1
GHITM	0.490361834	1
ZAR1	0.490510472	1
NOB1	0.49051342	1
IL1R2	0.490661876	1
CES1	0.490961471	1
IDH1	0.491102471	1
DRD5	0.491107136	1
OR7C2	0.491249277	1
HAO2	0.491250761	1
AIPL1	0.491550407	1
KRT82	0.491690698	1
VNN2	0.491696218	1
LTF	0.491841668	1
ME1	0.491841851	1
IFT81	0.491843307	1

SetID	P.value	N.Marker.Test
SLC39A2	0.492274818	1
TBC1D32	0.492278364	1
TRPM2	0.492278364	1
OR6C2	0.492279085	1
TMEM128	0.492571415	1
RESP18	0.492713208	1
LRIG1	0.492716394	1
PAQR7	0.492999977	1
ADAM17	0.493002865	1
NEK5	0.493148425	1
IGFN1	0.493149135	1
CHST4	0.493152275	1
DCAF6	0.493730453	1
RPF1	0.493874314	1
DGUOK	0.494016317	1
TMEM223	0.494160132	1
PIK3C2A	0.494300115	1
METTL7B	0.494301968	1
SHBG	0.494445283	1
SMARCAL1	0.494445331	1
TRIM25	0.494589869	1
OSGIN1	0.495013675	1
OR5M10	0.495158633	1
TNKS1BP1	0.495299038	1
CALCA	0.495300469	1
POGLUT3	0.495859651	1
OR52N4	0.495869416	1
ENTPD2	0.495872352	1
PLXNB2	0.496008672	1
SLC26A3	0.496008719	1
TMPRSS4	0.496430457	1
R3HCC1	0.496570967	1
CMYA5	0.497130063	1
MSI2	0.49726749	1
GATAD1	0.497412983	1
LEMD1	0.497547668	1
OR5J2	0.497690155	1
PALLD	0.49796529	1
RPL22L1	0.498104494	1
SMARCD1	0.498109572	1
LCN2	0.498242122	1
CATIP	0.499476221	1
ANXA4	0.500027674	1
37834	0.500432486	1
RUSF1,SLC5A2	0.500976975	1
ATP10A	0.502448322	1
LRP2	0.503009383	7
FAT2	0.506921733	9
SH3TC1	0.522122876	5

SetID	P.value	N.Marker.Test
ST6GAL2	0.523789085	2
LTBP2	0.524183739	3
ABCC2	0.524459076	6
ANKRD52	0.527182504	2
PCSK5	0.527657118	2
CLDN8	0.527909994	2
MCMDC2	0.528316486	2
PUM1	0.528750184	2
RPTOR	0.529080154	2
MYOF	0.529123584	4
P3H2	0.529900866	2
KCNK10	0.529988327	2
COL14A1	0.530040723	2
ALDOB	0.530176812	2
GMPR	0.530677852	2
PCM1	0.531228921	2
ABCG8	0.531294987	2
PPT1	0.53133288	2
EPRS1	0.531352606	2
PKN3	0.531352799	2
KATNAL2	0.531558746	2
ARPC4-TTLL3,TTLL	0.531667966	2
ACTRT2	0.53179576	2
POLD1	0.531961285	2
PCDHGA1,PCDHGA	0.532393348	2
GNPTAB	0.532446205	2
SCG5	0.532788348	2
RASSF4	0.532988884	2
KRT7	0.533011823	2
POLE	0.53320956	2
SLCO2A1	0.533442191	2
TBC1D31	0.533451976	2
SLC1A7	0.533650036	2
ADCY4	0.533678403	2
TRAPPC12	0.533763779	2
SLC13A4	0.533933766	2
ZP4	0.534016013	2
DOCK6	0.534025074	2
ADAMTS4	0.534025074	2
CDH17	0.53406333	2
URB1	0.534103087	2
COL24A1	0.534129841	2
CLCNKB	0.534146606	2
CEP135	0.534263352	2
VPS18	0.534278283	2
MEGF6	0.53440129	2
AKR1C2	0.534463718	2
MVP	0.53446855	2
DISP3	0.534552468	2

SetID	P.value	N.Marker.Test
CTDP1	0.534586797	2
STEAP3	0.534593583	2
ADGRE1	0.53474928	2
SCN5A	0.535005978	2
KIF7	0.53513169	2
TNS1	0.535282393	4
ADH1B	0.535412655	2
ACOX1	0.535412655	2
LRRC34	0.535451873	2
ATP2B4	0.535465199	2
EID3,TXNRD1	0.535833201	2
SLC12A7	0.535846971	2
OPRM1	0.535859675	2
AQP1	0.53613602	2
NDUFS1	0.536162749	2
GTF3C1	0.536302642	2
OR1S1	0.536379007	2
KRT84	0.53640844	2
SEMA3C	0.536433169	2
QSOX1	0.536699746	2
STAB1	0.536699746	2
KIF13B	0.536757836	4
KRT8	0.536863266	2
DDX24	0.536967811	2
NME8	0.536974293	2
ATP7B	0.537078288	4
UNC13B	0.537255003	3
PREX2	0.537555941	2
MYO9A	0.537739852	2
GAPDHS	0.537739852	2
LIPT1	0.537739852	2
ARHGAP28	0.537780125	2
NBEA	0.537872286	2
CIITA	0.537912233	2
SLC18A1	0.537951033	2
PPP6R1	0.538286458	2
DNAI2	0.538332489	2
PCK1	0.538332489	2
TMEM63A	0.538460761	2
HOXD1	0.538503881	2
KRT33A	0.538524578	2
ECEL1	0.538572846	4
RAB6C	0.538664354	2
PEPD	0.538718262	2
WDR17	0.538754147	2
PRUNE1	0.539006849	2
RAD52	0.5393595	2
CSF3R	0.539426305	2
HEPHL1	0.539443429	2

SetID	P.value	N.Marker.Test
SPATA13	0.539560416	2
ADSS1	0.539802711	2
THBS2	0.539832694	2
PAPLN	0.539868353	2
MYH9	0.540051496	2
MAP3K21	0.540053207	2
RAP1GAP2	0.540075532	2
AURKB	0.540202817	2
ATP5MF-PTCD1,PT	0.54042162	2
CEP290	0.540512982	2
ATG7	0.540613792	2
RAB11FIP1	0.541111624	2
CPT1B	0.541334926	2
ADAM2	0.541609493	2
TNRC6C	0.541629399	2
RCCD1	0.542281372	2
AOC1	0.542404851	2
SPO11	0.542627272	2
KDM1B	0.542978258	2
TMEM132A	0.544173422	2
EPB41L1	0.544680337	2
TECTA	0.545148616	2
SAPCD2	0.545306475	2
SLC4A2	0.546124847	2
CELSR2	0.546784201	4
NOD2	0.54778242	4
FBN3	0.548925461	5
TLN2	0.549464423	3
PRODH	0.551436264	2
ATP2C2	0.55205519	3
NBAS	0.552060722	3
COL12A1	0.552112474	4
CALD1	0.552238604	2
CAMSAP1	0.552238604	2
DNAJC13	0.552238604	2
HK1	0.552238604	2
HOOK2	0.552238604	2
LARGE1	0.552238604	2
LPIN3	0.552238604	2
MAN2B2	0.552238604	2
MCCC1	0.552238604	2
PLK4	0.552238604	2
PPARGC1A	0.552238604	2
SETD5	0.552238604	2
ARHGAP39	0.552238604	2
DSP	0.552238604	2
ENTPD3	0.552238604	2
GCAT	0.552238604	2
GSN	0.552238604	2

SetID	P.value	N.Marker.Test
IRS1	0.552238604	2
LMOD3	0.552238604	2
PCCB	0.552238604	2
RPAP1	0.552238604	2
TBX2	0.552238604	2
TMPRSS15	0.552238604	2
UBR4	0.552238604	2
DYTN	0.552238604	2
KLC2	0.552238604	2
CERS4	0.552238604	2
GOLGA2	0.552238604	2
PTPN13	0.552238604	2
TPCN2	0.553108725	3
TM7SF2	0.55426319	3
KLHL33	0.554530613	3
ADGRV1	0.55471724	10
UROC1	0.555083118	3
L3HYPDH	0.555437384	3
P2RX5	0.555470186	3
MYO1A	0.555689186	3
COL21A1	0.556042042	3
NEU4	0.556749978	3
FOLH1	0.556866795	3
KRT38	0.556963551	3
CLCNKA	0.557521367	3
POLQ	0.558083644	3
CES5A	0.558158114	3
COL22A1	0.558387762	3
MTCL1	0.558423434	3
CCDC40	0.558643057	3
RP1L1	0.558665339	3
CACNA1G	0.559122429	3
CORIN	0.559935317	3
GNAT2	0.560804848	3
TENM3	0.560806243	3
ITGA7	0.560806547	3
KBTBD13	0.560939593	3
AKAP9	0.561090207	3
ABCA12	0.561633137	3
EPN3	0.56239621	3
IPO4	0.563166533	3
PFKM	0.563490315	3
BHMT2	0.565372156	3
SHPK	0.566097683	3
LRGUK	0.566292396	3
RGPD4	0.566655942	6
INPP5B	0.567722964	3
FBF1	0.567722964	3
CARS1	0.570935209	3

SetID	P.value	N.Marker.Test
USP43	0.573117358	2
SLC26A4	0.576813212	3
TRIM45	0.577130819	2
SAMD9	0.577376589	2
TUBB1	0.577625261	2
ATP13A3	0.577870064	2
ATP1A4	0.577930934	6
DIP2A	0.57860644	2
RNF123	0.579814479	2
CUL7	0.580055395	2
CYP1A2	0.580541806	2
CYP4F2	0.580782214	2
TRIM37	0.580782214	2
DAAM1	0.580787327	2
RANBP2	0.581022788	2
KCNQ5	0.581026245	2
ATAD3C	0.581154723	4
CDC14A	0.581259149	2
ANKRD11	0.581264594	2
SRCAP	0.581656578	4
UNC13C	0.581737376	2
GPATCH1	0.581971034	2
ZNF607	0.582219011	2
CACNA1B	0.582221179	2
NECAB2	0.582222721	2
TMEM252	0.582222721	2
SVOPL	0.582459919	2
SLC36A3	0.582462418	2
SI	0.582929021	2
ZBBX	0.583163438	2
GPX7	0.583635306	2
OR10X1	0.583640538	2
NDST4	0.58364357	2
FES	0.583863408	2
CABIN1	0.583869523	2
FAM13B	0.583872864	2
ALDH3A2	0.583873395	2
POLR1C	0.584100535	2
ERCC2	0.584106285	2
PLA2G4D	0.584340082	2
HEATR6	0.584571188	2
SH2D3C	0.584575147	2
H6PD	0.584796924	2
HEATR1	0.584806376	2
ITGA9	0.584817654	2
DCLRE1A	0.585036053	2
ZCCHC4	0.585266654	2
ISX	0.585269431	2
HELZ2	0.585317824	5

SetID	P.value	N.Marker.Test
NME5	0.585502785	2
NAXD	0.585740236	2
EEF2K	0.585743457	2
SLC25A46	0.585960805	2
SLC24A1	0.585961821	2
AP1M2	0.585966615	2
FMO2	0.585966763	2
EXOSC6	0.586192705	2
SH3BP4	0.586193789	2
PDGFRL	0.586195228	2
GNPTG	0.586197942	2
SLC26A5	0.586420413	2
SAE1	0.586427254	2
NMUR2	0.586651866	2
DACT2	0.586653879	2
NFATC3	0.586657012	2
PTPRZ1	0.586657961	2
BTNL3	0.586889491	2
CHRND	0.586889775	2
ZADH2	0.58711236	2
PRKAR1A,WIPI1	0.58711363	2
RBBP8	0.587114258	2
COL10A1,NT5DC1	0.587567783	2
BAHCC1	0.587792071	2
UBE3B	0.587792071	2
FERMT1	0.587794784	2
RARRES1	0.58779536	2
LLGL2	0.588021543	2
TTC12	0.588022431	2
CDHR3	0.588025183	2
LAMB3	0.588025183	2
RRP12	0.588025573	2
OR10J1	0.588026723	2
FRMD4B	0.588036901	4
ALAD	0.588253633	2
LPP	0.588256669	2
ZNF74	0.588475167	2
TULP2	0.588479821	2
ADAM18	0.588703304	2
PLEKHA6	0.588705043	2
ADGRL1	0.588705812	2
SLC38A2	0.588709728	2
ATP2A3	0.588930366	2
PNMT	0.58915545	2
HYLS1,PUS3	0.589158392	2
TMEM214	0.589382259	2
ATP6V0A2	0.589602128	2
DCST2	0.59005356	2
MYADML2	0.590054711	2

SetID	P.value	N.Marker.Test
SASH1	0.590274132	2
PRICKLE1	0.59028101	2
NLRP12	0.590496329	2
SLA2	0.59049828	2
RARS1	0.590722355	2
CHD1L	0.590723696	2
IL22RA2	0.590724632	2
CENPJ	0.59094356	2
TAS1R3	0.590944112	2
BIN2	0.590945742	2
CPQ	0.590948181	2
SULT1C2	0.591160155	2
PPP1R21	0.591165201	2
AOC2	0.591167212	2
METTL8	0.591461297	2
ABCB1	0.591465857	2
LGMN	0.591601386	2
LYPLAL1	0.591607177	2
ANKZF1	0.591607555	2
PCDHGA1,PCDHGA	0.591609994	2
CEP152	0.59161064	2
CLCN7	0.591611347	2
TJP3	0.591690086	3
TIMM44	0.591822479	2
DUOXA1	0.591828939	2
KIF13A	0.591829176	2
NRDE2	0.591830738	2
APLP2	0.591835029	2
NEU3	0.591835029	2
MUC5B	0.592047087	2
MCM3	0.592050397	2
CFAP44	0.592054397	2
MAP7D1	0.592266665	2
COL3A1	0.592267959	2
CILP2	0.592270288	2
YIPF2	0.592270288	2
MYPN	0.592272424	2
ABCA6	0.592275288	2
GRM6	0.592305768	4
ALKBH7	0.592481375	2
APOB	0.592486557	2
PARD3B	0.592492538	2
BCAR1	0.592706427	2
ASL	0.592706992	2
NXNL1	0.592708401	2
PRRC2C	0.59271098	2
SPINK5	0.59271302	2
KISS1R	0.592878265	2
TMEM67	0.592923365	2

SetID	P.value	N.Marker.Test
GYS2	0.592925483	2
MYH11	0.592926143	2
FRMPD1	0.592927575	2
RELN	0.592927716	2
ANXA5	0.593145176	2
PLOD2	0.593145176	2
GDAP2	0.593151068	2
FUT2	0.593242008	5
MRPL23	0.593360397	2
CARS2	0.593364054	2
NAV1	0.593364264	2
ARHGEF10	0.593579938	2
GPLD1	0.593799045	2
SUOX	0.593799273	2
STON1,STON1-GTF	0.593801764	2
ATAD3B	0.593905101	6
FAM160A1	0.594011459	2
PBRM1	0.594011459	2
EPHX2	0.594017407	2
HEXB	0.594222386	2
HEG1	0.594443323	2
MEIS3	0.594446601	2
ALDH5A1	0.59444854	2
ABCC10	0.594651705	2
CACNA2D4	0.594663486	2
TMEM82	0.5948818	2
NUP188	0.595091625	2
GJB7	0.595525665	2
LRP1	0.595525665	2
ARHGAP21	0.595729927	2
TRPM7	0.595734115	2
CTBP2	0.595734706	2
CCR3	0.595948209	2
EVI5	0.595949101	2
RIN1	0.596161076	2
BEST2	0.596373405	2
MSH4	0.596375746	2
HMGCLL1	0.596380954	2
MYH15	0.596582083	2
GK2	0.596585262	2
SULF2	0.596587042	2
B3GNT4	0.596588578	2
FREM2	0.596795411	2
BRF1	0.596796167	2
CCDC88C	0.596796167	2
RIN2	0.597005323	2
FSCN1	0.59700814	2
TUBA3E	0.597009925	2
TENM4	0.597035749	6

SetID	P.value	N.Marker.Test
OPN4	0.597217215	2
GATAD2A	0.597225554	2
MYO7A	0.597395255	6
CACNB2	0.597850657	2
MCF2L2	0.598048353	3
MLXIPL	0.598267477	2
TTC28	0.598482915	2
SLC5A11	0.598900441	2
MYO3A	0.59890058	2
NPHS2	0.599105682	2
CHD8	0.599105756	2
HCN3	0.599107142	2
TRIOBP	0.599108694	2
CEP170B	0.599519391	2
KRT14	0.599943432	2
MYOD1	0.600471854	2
MMP13	0.600766332	2
VCL	0.600766332	2
DCHS2	0.600769282	2
ASPM	0.600803941	6
ANK3	0.600973497	2
IQCA1	0.601172735	2
RANBP17	0.602391597	2
FBP2	0.60320126	2
SOAT2	0.603201654	2
TNS2	0.60359745	2
TMEM132D	0.603601203	2
NT5E	0.604003949	2
MYO1C	0.604713342	3
TEKT5	0.60565822	3
GHR	0.606576998	3
DNAH2	0.607501985	11
ST7L	0.609183558	3
GBE1	0.610270005	3
MACF1	0.622709144	5
AMPD1	0.626646529	4
DUOX2	0.628981553	9
MYH7B	0.629866668	8
TRPM1	0.639374533	4
HIVEP1	0.640837826	4
CLUAP1	0.642300416	3
MICAL2	0.642740195	4
SDK1	0.642981447	4
ABCA4	0.644079138	5
POLG	0.644150734	4
COL9A1	0.644225656	4
SLC7A4	0.644864692	4
SERPINA1	0.64616284	3
COL7A1	0.647247401	3

SetID	P.value	N.Marker.Test
DDX54	0.647630608	4
MYH14	0.648488904	7
OTOF	0.657420504	5
GRID2	0.660971891	3
CYP1A1	0.6610764	3
MICAL1	0.661162862	5
POSTN	0.664636221	4
NCOR2	0.673543789	5
TPR	0.675256986	6
PCDHGA1,PCDHGA	0.678858578	6
SPTA1	0.691739509	4
DUSP22	0.692600192	1
ACADL	0.693539633	2
RRP7A	0.696389682	1
ACE	0.698366651	6
FER1L6	0.700820496	3
LRRFIP1	0.70107985	3
MYH8	0.702011868	7
A2M	0.70246176	1
ABTB1	0.70246176	1
ACTG2	0.70246176	1
ACTR1B	0.70246176	1
ADAM7	0.70246176	1
ADAMTS1	0.70246176	1
ADGRL4	0.70246176	1
ADPRHL1	0.70246176	1
AFAP1L1	0.70246176	1
AGMO	0.70246176	1
AHNAK2	0.70246176	1
AIMP2,EIF2AK1	0.70246176	1
AK6,TAF9	0.70246176	1
ALDH16A1	0.70246176	1
ALDH3A1	0.70246176	1
AMPD2	0.70246176	1
ANGPT1	0.70246176	1
ANKFY1	0.70246176	1
APLF	0.70246176	1
ARHGDIG	0.70246176	1
ARHGEF25	0.70246176	1
ARHGEF26	0.70246176	1
ARID4B	0.70246176	1
ARL13B,STX19	0.70246176	1
ARTN	0.70246176	1
ASB1	0.70246176	1
ASB13	0.70246176	1
ASGR1	0.70246176	1
ASPA,SPATA22	0.70246176	1
ASXL2	0.70246176	1
ATCAY	0.70246176	1

SetID	P.value	N.Marker.Test
ATG2B	0.70246176	1
ATG4D	0.70246176	1
ATP13A4	0.70246176	1
ATP1A2	0.70246176	1
ATP23	0.70246176	1
ATP4A	0.70246176	1
B3GAT2	0.70246176	1
B4GALT6	0.70246176	1
BBS2	0.70246176	1
BNIP1	0.70246176	1
BPTF	0.70246176	1
BUB1B	0.70246176	1
C17orf64	0.70246176	1
C1D	0.70246176	1
C1orf109	0.70246176	1
C1QTNF1	0.70246176	1
C1RL	0.70246176	1
C22orf23	0.70246176	1
C4orf45	0.70246176	1
C7orf50	0.70246176	1
C9orf139,FUT7	0.70246176	1
CACNB1	0.70246176	1
CARD10	0.70246176	1
CASP7	0.70246176	1
CBLN3	0.70246176	1
CCDC125	0.70246176	1
CCDC89	0.70246176	1
CCNDBP1	0.70246176	1
CDH15	0.70246176	1
CDH2	0.70246176	1
CEBPZ	0.70246176	1
CEP131	0.70246176	1
CEP44	0.70246176	1
CEP78	0.70246176	1
CERS1,GDF1	0.70246176	1
CFAP126	0.70246176	1
CFAP43	0.70246176	1
CHDH	0.70246176	1
CHEK2	0.70246176	1
CHMP1A	0.70246176	1
CHORDC1	0.70246176	1
CHRNA6	0.70246176	1
CHURC1,CHURC1-[illegible]	0.70246176	1
CIDEB,NOP9	0.70246176	1
CKAP2L	0.70246176	1
CLCA2	0.70246176	1
CLDN7	0.70246176	1
CLEC4F	0.70246176	1
CLEC7A	0.70246176	1

SetID	P.value	N.Marker.Test
CLIC5	0.70246176	1
CNTNAP1	0.70246176	1
COMP	0.70246176	1
CRB1	0.70246176	1
CRTAP	0.70246176	1
CSE1L	0.70246176	1
CSMD2,HMGB4	0.70246176	1
CSPG4	0.70246176	1
CTRC	0.70246176	1
CTU1	0.70246176	1
CYFIP2	0.70246176	1
CYP26C1	0.70246176	1
CYP3A5	0.70246176	1
DECR2	0.70246176	1
DGCR6L	0.70246176	1
DHODH	0.70246176	1
DHRS1	0.70246176	1
DKK3	0.70246176	1
DLK2	0.70246176	1
DMPK	0.70246176	1
DNAJC6	0.70246176	1
DNASE1L3	0.70246176	1
DNASE2B	0.70246176	1
DOC2A	0.70246176	1
DPH7	0.70246176	1
DTNB	0.70246176	1
DUSP15	0.70246176	1
EEA1	0.70246176	1
EEFSEC	0.70246176	1
EFL1	0.70246176	1
EHD3	0.70246176	1
EIF3L	0.70246176	1
ELP1	0.70246176	1
ELP3	0.70246176	1
ELP5	0.70246176	1
ENKD1	0.70246176	1
ENTPD7	0.70246176	1
EPAS1	0.70246176	1
EPDR1	0.70246176	1
EPHA7	0.70246176	1
ESYT2	0.70246176	1
EXD3	0.70246176	1
F12	0.70246176	1
F2RL1	0.70246176	1
FAM210A	0.70246176	1
FAM89A	0.70246176	1
FANCM	0.70246176	1
FBXO40	0.70246176	1
FGB	0.70246176	1

SetID	P.value	N.Marker.Test
FIGN	0.70246176	1
FLII	0.70246176	1
FMNL3	0.70246176	1
FOXP4	0.70246176	1
FRMD4A	0.70246176	1
FSHR	0.70246176	1
FZD10	0.70246176	2
GAD2	0.70246176	1
GATA5	0.70246176	1
GDF15	0.70246176	1
GDF9	0.70246176	1
GFPT2	0.70246176	1
GIGYF2,KCNJ13	0.70246176	1
GIMAP1-GIMAP5,(	0.70246176	1
GLB1L3	0.70246176	1
GLDC	0.70246176	1
GOLIM4	0.70246176	1
GOLM2	0.70246176	1
GORAB	0.70246176	1
GPR37L1	0.70246176	1
GRB7	0.70246176	1
GRHPR	0.70246176	1
GRIN3B	0.70246176	1
GRM8	0.70246176	1
GTPBP2	0.70246176	1
H1-1	0.70246176	1
HAS1	0.70246176	1
HDAC5	0.70246176	1
HK3	0.70246176	1
HMGCS2	0.70246176	1
HOXC11	0.70246176	1
HOXC4	0.70246176	1
HOXD10	0.70246176	1
HOXD11	0.70246176	1
HTR1A	0.70246176	1
HTRA4	0.70246176	1
HVCN1	0.70246176	1
IBA57	0.70246176	1
IFITM5	0.70246176	1
IGDCC4	0.70246176	1
IGSF9	0.70246176	1
IL17RE	0.70246176	1
INSL3	0.70246176	1
IQCH	0.70246176	1
ISM1	0.70246176	1
ITPK1	0.70246176	1
ITPR1	0.70246176	1
IYD	0.70246176	1
JADE1	0.70246176	1

SetID	P.value	N.Marker.Test
KARS1	0.70246176	1
KCNK15	0.70246176	1
KCNK16	0.70246176	1
KIAA1671	0.70246176	1
KIDINS220	0.70246176	1
KLHL17	0.70246176	1
KLHL29	0.70246176	1
KNL1	0.70246176	1
KRT19	0.70246176	1
KRT79	0.70246176	1
LARP1B	0.70246176	1
LGR5	0.70246176	1
LIMK2	0.70246176	1
LINGO1	0.70246176	1
LIPE	0.70246176	1
LIPF	0.70246176	1
LRRC45	0.70246176	1
LRRC63	0.70246176	1
LRRC71	0.70246176	1
LYST	0.70246176	1
MAMDC4	0.70246176	1
MAP3K6	0.70246176	1
MAPKAPK5	0.70246176	1
MARCHF6	0.70246176	1
MAST4	0.70246176	1
MBOAT7	0.70246176	1
MCM7	0.70246176	1
MESP1	0.70246176	1
METTL18	0.70246176	1
MFGE8	0.70246176	1
MFSD11,SRSF2	0.70246176	1
MGAT2	0.70246176	1
MMADHC	0.70246176	1
MMP14	0.70246176	1
MMP25	0.70246176	1
MOGAT2	0.70246176	1
MPZL3	0.70246176	1
MROH7	0.70246176	1
MRPL24	0.70246176	1
MRVI1	0.70246176	1
MSTN	0.70246176	1
MTNR1B	0.70246176	1
MUC4	0.70246176	1
MYBL1	0.70246176	1
MYL6B	0.70246176	1
MYO16	0.70246176	1
MYOM3	0.70246176	1
MYORG	0.70246176	1
MYSM1	0.70246176	1

SetID	P.value	N.Marker.Test
NCOA5	0.70246176	1
NELL1	0.70246176	1
NFS1	0.70246176	1
NIPAL2	0.70246176	1
NKAIN4	0.70246176	1
NKAPD1	0.70246176	1
NKD1	0.70246176	1
NPBWR2	0.70246176	1
NPTN	0.70246176	1
NR1I3	0.70246176	1
NRAP	0.70246176	1
NRG1	0.70246176	1
NT5C1A	0.70246176	1
NUAK2	0.70246176	1
NUP37	0.70246176	1
NUP93	0.70246176	1
OGDHL	0.70246176	1
OIT3	0.70246176	1
OLFML2A	0.70246176	1
OR2AG1	0.70246176	1
OR4A16	0.70246176	1
OR4S1	0.70246176	1
OR5C1	0.70246176	1
ORC4	0.70246176	1
OSBPL6	0.70246176	1
OTUD7B	0.70246176	1
PAQR5	0.70246176	1
PASK	0.70246176	1
PCDHGA1	0.70246176	1
PCDHGA1,PCDHGA	0.70246176	1
PCSK9	0.70246176	1
PCYOX1	0.70246176	1
PDLIM7	0.70246176	1
PEAK1	0.70246176	1
PEX5	0.70246176	1
PFN2	0.70246176	1
PGLYRP1	0.70246176	1
PHLDB2	0.70246176	1
PIEZO2	0.70246176	1
PIGG	0.70246176	1
PIGM	0.70246176	1
PIGP	0.70246176	1
PISD	0.70246176	1
PKD2L1	0.70246176	1
PLA2G1B	0.70246176	1
PLEK	0.70246176	1
PLEKHH1	0.70246176	1
PLIN3	0.70246176	1
PLS1	0.70246176	1

SetID	P.value	N.Marker.Test
PLXNA1	0.70246176	1
POLA2	0.70246176	1
POLR1E	0.70246176	1
POM121C	0.70246176	1
POU6F1	0.70246176	1
PPM1J	0.70246176	1
PPP1R17	0.70246176	1
PPP1R9A	0.70246176	1
PRDM2	0.70246176	1
PRPF18	0.70246176	1
PRPH	0.70246176	1
PRR14	0.70246176	1
PRR15L	0.70246176	1
PRRX2	0.70246176	1
PRSS58	0.70246176	1
PSAT1	0.70246176	1
PSMB1	0.70246176	1
PTCD3	0.70246176	1
PTF1A	0.70246176	1
PTH2R	0.70246176	1
PTK2	0.70246176	1
PTPN12	0.70246176	1
PTPRE	0.70246176	1
PTPRM	0.70246176	1
PTPRT	0.70246176	1
PYROXD1	0.70246176	1
QPCT	0.70246176	1
RABEPK	0.70246176	1
RABL2B	0.70246176	1
RALY	0.70246176	1
RAPGEF4	0.70246176	1
RAPGEF6	0.70246176	1
RCHY1	0.70246176	1
RCN2	0.70246176	1
RETSAT	0.70246176	1
REV1	0.70246176	1
RFTN1	0.70246176	1
RFWD3	0.70246176	1
RGS9	0.70246176	1
RHBDF2	0.70246176	1
RIMBP2	0.70246176	1
RITA1	0.70246176	1
RNF112	0.70246176	1
RNF167	0.70246176	1
ROR2	0.70246176	1
RPS6KA5	0.70246176	1
RRBP1	0.70246176	1
RSPH10B2	0.70246176	1
RTKN2	0.70246176	1

SetID	P.value	N.Marker.Test
RTL1	0.70246176	1
RUBCN	0.70246176	1
RUFY4	0.70246176	1
RWDD2B	0.70246176	1
RXFP2	0.70246176	1
SBNO2	0.70246176	1
SCEL	0.70246176	1
SCG2	0.70246176	1
SCP2	0.70246176	1
SDHC	0.70246176	1
SEPTIN7	0.70246176	1
SERGEF	0.70246176	1
SERPINA12	0.70246176	1
SIGLEC5	0.70246176	1
SIRT4	0.70246176	1
SLC12A3	0.70246176	1
SLC17A5	0.70246176	1
SLC19A2	0.70246176	1
SLC22A12	0.70246176	1
SLC25A20	0.70246176	1
SLC25A52	0.70246176	1
SLC26A7	0.70246176	1
SLC2A2	0.70246176	1
SLC35F4	0.70246176	1
SLC45A1	0.70246176	1
SLC45A3	0.70246176	1
SLC4A1	0.70246176	1
SLC52A1	0.70246176	1
SLC6A13	0.70246176	1
SLC6A20	0.70246176	1
SLC9A1	0.70246176	1
SMUG1	0.70246176	1
SNAP47	0.70246176	1
SND1	0.70246176	1
SOX9	0.70246176	1
SPAG7	0.70246176	1
STAP1	0.70246176	1
STAT5A	0.70246176	1
STEAP2	0.70246176	1
STK10	0.70246176	1
SWSAP1	0.70246176	1
TAF15	0.70246176	1
TAF8	0.70246176	1
TBC1D2	0.70246176	1
TCF15	0.70246176	1
TEX26	0.70246176	1
TFCP2L1	0.70246176	1
TH	0.70246176	1
THBS1	0.70246176	1

SetID	P.value	N.Marker.Test
THOC5	0.70246176	1
TIMM22	0.70246176	1
TJAP1	0.70246176	1
TLDC2	0.70246176	1
TMEM38B	0.70246176	1
TMEM39B	0.70246176	1
TMEM63C	0.70246176	1
TMEM72	0.70246176	1
TMEM86A	0.70246176	1
TMOD3	0.70246176	1
TNIK	0.70246176	1
TOM1L1	0.70246176	1
TP53BP2	0.70246176	1
TP53INP1	0.70246176	1
TPSG1	0.70246176	1
TRAF3IP1	0.70246176	1
TRAM1L1	0.70246176	1
TRDMT1	0.70246176	1
TRMT12	0.70246176	1
TRMT9B	0.70246176	1
TRPM4	0.70246176	1
TTLL2	0.70246176	1
TULP1	0.70246176	1
TYRP1	0.70246176	1
UBAC2	0.70246176	1
UBTD2	0.70246176	1
UCP1	0.70246176	1
UEVLD	0.70246176	1
UFSP1	0.70246176	1
UMODL1	0.70246176	1
UNC13D	0.70246176	1
URGCP,URGCP-MI	0.70246176	1
UTP6	0.70246176	1
VANGL2	0.70246176	1
VAX2	0.70246176	1
VIPR2	0.70246176	1
VPS33B	0.70246176	1
VPS53	0.70246176	1
VTA1	0.70246176	1
VTCN1	0.70246176	1
VWA1	0.70246176	1
WDR41	0.70246176	1
WDR66	0.70246176	1
WDSUB1	0.70246176	1
WEE2	0.70246176	1
WT1	0.70246176	1
XRCC4	0.70246176	1
XYLT1	0.70246176	1
ZHX2	0.70246176	1

SetID	P.value	N.Marker.Test
ZKSCAN2	0.70246176	1
ZNF292	0.70246176	1
ZNF423	0.70246176	1
ZNF445	0.70246176	1
ZNF609	0.70246176	1
ZNF679	0.70246176	1
ZNF689	0.70246176	1
ZNF701	0.70246176	1
ZNF784	0.70246176	1
ZSWIM2	0.70246176	1
ZSWIM4	0.70246176	1
PIEZO1	0.70289895	8
RYR3	0.704526485	4
CFAP52	0.708002469	4
ADAMTS8	0.709384089	4
DNAH12	0.71119819	4
AP5Z1	0.712365011	4
PCDHA1	0.712365011	4
OR4Q3	0.712691375	1
IQGAP2	0.713355516	4
C1QTNF9	0.714981125	1
TRPM8	0.717787827	4
PCDHA1,PCDHA2,F	0.72042791	1
GBA	0.721564947	1
PDXDC1	0.721564947	1
TRIM73	0.721564947	1
TUBA3D	0.721564947	1
ARMC4	0.721847386	1
C9	0.721915386	1
C3	0.721922302	1
NUP133	0.721942098	1
PRKD2	0.721960459	1
STRN4	0.721960459	1
IL17RB	0.722108876	1
KRT86	0.722159047	1
PRSS12	0.722191788	1
PCYOX1L	0.722200707	1
FANCC	0.72224604	1
SEC24C	0.722249166	1
ARL13B	0.722249781	1
NXF1	0.722252959	1
RRS1	0.72226071	1
GORASP1	0.722263972	1
AKAP8	0.722303757	1
KLHL41	0.722318913	1
NTN3	0.722336314	1
GPRC5C	0.722336739	1
GJD4	0.722338188	1
SAMD12	0.722338818	1

SetID	P.value	N.Marker.Test
ABCC5	0.722357872	1
TMEM139	0.722391793	1
SIGLEC1	0.722477537	1
SFR1	0.722512192	1
S100A3	0.722541359	1
ADIPOQ	0.72257996	1
NCF2	0.722610584	1
CPNE3	0.722647245	1
GAS8	0.722653257	1
ALPI	0.722659808	1
SLC5A8	0.722727508	1
ACP7	0.722758886	1
LY75,LY75-CD302	0.72279553	1
SYT6	0.722804127	1
KIAA1324L	0.7228081	1
ELP4	0.722826855	1
CYP4A22	0.722839058	1
WRAP73	0.722841678	1
OR51T1	0.72285624	1
CCDC9	0.722883899	1
RIPOR1	0.723070062	1
ATMIN	0.723143686	1
GALNT7	0.723178963	1
PLEKHA1	0.723263072	1
SUMF1	0.723266925	1
CEP55	0.723280165	1
COCH	0.723286584	1
GATB	0.72329141	1
OLFML2B	0.723304591	1
AFTPH	0.723327727	1
CAMK1,OGG1	0.723328731	1
TTC21B	0.723440876	1
CDH26	0.723494221	1
ALKBH4	0.72351141	1
KPNA2	0.723532907	1
MARCO	0.723616024	2
OR6K6	0.723616024	2
ZNF804B	0.723616024	2
AAMDC	0.723616024	1
ABCB10	0.723616024	1
ABCB4	0.723616024	1
ABCB9	0.723616024	1
ABCC4	0.723616024	1
ABHD16B	0.723616024	1
ABI3BP	0.723616024	1
ABLIM1	0.723616024	1
ABRA	0.723616024	1
ACAP2	0.723616024	1
ACAP3	0.723616024	1

SetID	P.value	N.Marker.Test
ACAT2	0.723616024	1
ACMSD	0.723616024	1
ACOX3	0.723616024	1
ACOXL	0.723616024	1
ACP2	0.723616024	1
ACP3	0.723616024	1
ACSF3	0.723616024	1
ACSL6	0.723616024	1
ACSM3	0.723616024	1
ACSS1	0.723616024	1
ACTN2	0.723616024	1
ACTR3C	0.723616024	1
ACY3	0.723616024	1
ADA2	0.723616024	1
ADAM15	0.723616024	1
ADAM33	0.723616024	1
ADAMTS13	0.723616024	1
ADAMTS3	0.723616024	1
ADAMTS6	0.723616024	1
ADAMTS7	0.723616024	1
ADAMTS9	0.723616024	1
ADAMTSL1	0.723616024	1
ADAP1	0.723616024	1
ADAP2	0.723616024	1
ADGRA1	0.723616024	1
ADGRA2	0.723616024	1
ADGRA3	0.723616024	1
ADGRB3	0.723616024	1
ADGRF4	0.723616024	1
ADI1	0.723616024	1
ADORA2A	0.723616024	1
ADORA3,TMIGD3	0.723616024	1
AFAP1	0.723616024	1
AFDN	0.723616024	1
AGAP1	0.723616024	1
AGAP2	0.723616024	1
AGBL1	0.723616024	1
AGK	0.723616024	1
AGPAT2	0.723616024	1
AGRN	0.723616024	1
AHCTF1	0.723616024	1
AHCY	0.723616024	1
AHCYL2	0.723616024	1
AIP	0.723616024	1
AIRE	0.723616024	1
AK3	0.723616024	1
AKAP13	0.723616024	1
AKAP3	0.723616024	1
AKAP6	0.723616024	1

SetID	P.value	N.Marker.Test
AKR1C1	0.723616024	1
AKR7A3	0.723616024	1
ALB	0.723616024	1
ALDH1A1	0.723616024	1
ALDH4A1	0.723616024	1
ALDH8A1	0.723616024	1
ALG1L	0.723616024	1
ALG9	0.723616024	1
ALK	0.723616024	1
ALKBH3	0.723616024	1
ALOX12B	0.723616024	1
ALOXE3	0.723616024	1
ALPL	0.723616024	1
AMBRA1	0.723616024	1
AMDHD2	0.723616024	1
AMOTL1	0.723616024	1
ANAPC15,LRTOMT	0.723616024	1
ANGPT2,MCPH1	0.723616024	1
ANKAR	0.723616024	1
ANKMY1	0.723616024	1
ANKRD54	0.723616024	1
ANKRD55	0.723616024	1
ANO10	0.723616024	1
ANXA2	0.723616024	1
AP1S3	0.723616024	1
AP4B1	0.723616024	1
APBB1	0.723616024	1
APCDD1	0.723616024	1
APH1B	0.723616024	1
APLP1	0.723616024	1
APMAP	0.723616024	1
APOA4	0.723616024	1
APOBEC2	0.723616024	1
APTX	0.723616024	1
AQR	0.723616024	1
ARFIP1	0.723616024	1
ARHGAP1	0.723616024	1
ARHGAP11B	0.723616024	1
ARHGAP15	0.723616024	1
ARHGAP19	0.723616024	1
ARHGAP24	0.723616024	1
ARHGAP8,PRR5-AI	0.723616024	1
ARHGEF15	0.723616024	1
ARHGEF17	0.723616024	1
ARHGEF2	0.723616024	1
ARID3C	0.723616024	1
ARL5C	0.723616024	1
ARNT	0.723616024	1
ARPC1B	0.723616024	1

SetID	P.value	N.Marker.Test
ARPIN,ARPIN-AP3S	0.723616024	1
ARRB2	0.723616024	1
ARRDC3	0.723616024	1
ARVCF	0.723616024	1
ASAH1	0.723616024	1
ASAP2	0.723616024	1
ASB3,CHAC2,GPR7	0.723616024	1
ASIC2	0.723616024	1
ASIC5	0.723616024	1
ASNS	0.723616024	1
ASPH	0.723616024	1
ASPHD1	0.723616024	1
ATF2	0.723616024	1
ATP11A	0.723616024	1
ATP12A	0.723616024	1
ATP1A3	0.723616024	1
ATP2B2	0.723616024	1
ATP4B	0.723616024	1
ATP6V0E2	0.723616024	1
ATP6V1B1	0.723616024	1
ATP6V1E2	0.723616024	1
ATP9B	0.723616024	1
ATRIP	0.723616024	1
ATXN7	0.723616024	1
AXDND1,NPHS2	0.723616024	1
AXL	0.723616024	1
B3GNT3	0.723616024	1
B4GALT4	0.723616024	1
BAK1	0.723616024	1
BARHL2	0.723616024	1
BBOF1	0.723616024	1
BBOX1	0.723616024	1
BCAN	0.723616024	1
BCAP29	0.723616024	1
BCHE	0.723616024	1
BCL11B	0.723616024	1
BCO1	0.723616024	1
BDH1	0.723616024	1
BDKRB2	0.723616024	1
BEST1	0.723616024	1
BEST3	0.723616024	1
BHLHE40	0.723616024	1
BICRAL	0.723616024	1
BIRC7	0.723616024	1
BIRC8	0.723616024	1
BIVM-ERCC5,ERCC	0.723616024	1
BLVRB	0.723616024	1
BLZF1	0.723616024	1
BMP10	0.723616024	1

SetID	P.value	N.Marker.Test
BMP6	0.723616024	1
BOD1L1	0.723616024	1
BPHL	0.723616024	1
BPIFB4	0.723616024	1
BPIFC	0.723616024	1
BRCA1	0.723616024	1
BRD1	0.723616024	1
BTBD11	0.723616024	1
BUB1B-PAK6,PAK6	0.723616024	1
C10orf71	0.723616024	1
C11orf49	0.723616024	1
C12orf50	0.723616024	1
C16orf89	0.723616024	1
C1QL4	0.723616024	1
C1QTNF2	0.723616024	1
C1S	0.723616024	1
C2CD4B	0.723616024	1
C2orf78	0.723616024	1
C4orf33	0.723616024	1
C5orf22	0.723616024	1
C8A	0.723616024	1
C8B	0.723616024	1
C8orf34	0.723616024	1
C8orf74	0.723616024	1
CA11	0.723616024	1
CA12	0.723616024	1
CAB39L	0.723616024	1
CACNA1A	0.723616024	1
CACNA1C	0.723616024	1
CACNA1D	0.723616024	1
CACNA1I	0.723616024	1
CACNG5	0.723616024	1
CADM4	0.723616024	1
CADPS2,RNF133	0.723616024	1
CADPS2,RNF148	0.723616024	1
CALCOCO2	0.723616024	1
CALHM1	0.723616024	1
CALHM2	0.723616024	1
CALHM3	0.723616024	1
CALHM4,TRAPPC3	0.723616024	1
CAMK1G	0.723616024	1
CAMKK2	0.723616024	1
CAMSAP2	0.723616024	1
CAND2	0.723616024	1
CANT1	0.723616024	1
CAPG	0.723616024	1
CAPN1	0.723616024	1
CAPN11	0.723616024	1
CAPN2	0.723616024	1

SetID	P.value	N.Marker.Test
CAPS2,GLIPR1L1	0.723616024	1
CAPSL	0.723616024	1
CASD1	0.723616024	1
CASKIN1	0.723616024	1
CASP8	0.723616024	1
CASQ1	0.723616024	1
CBFA2T3	0.723616024	1
CBS	0.723616024	1
CCDC146,FGL2	0.723616024	1
CCDC151	0.723616024	1
CCDC173	0.723616024	1
CCDC42	0.723616024	1
CCDC62	0.723616024	1
CCDC81	0.723616024	1
CCDC86	0.723616024	1
CCDC90B	0.723616024	1
CCKBR	0.723616024	1
CCNL2	0.723616024	1
CCPG1	0.723616024	1
CCZ1	0.723616024	1
CD207	0.723616024	1
CD276	0.723616024	1
CD3EAP,ERCC1	0.723616024	1
CD4	0.723616024	1
CD44	0.723616024	1
CD9	0.723616024	1
CDAN1	0.723616024	1
CDC37L1	0.723616024	1
CDC42EP4	0.723616024	1
CDHR4	0.723616024	1
CDK15	0.723616024	1
CDK5RAP2	0.723616024	1
CDNF	0.723616024	1
CECR2	0.723616024	1
CEL	0.723616024	1
CELA2B	0.723616024	1
CELA3B	0.723616024	1
CENPK	0.723616024	1
CENPP,ECM2	0.723616024	1
CENPT	0.723616024	1
CEP76,PSMG2	0.723616024	1
CERS5	0.723616024	1
CERS6	0.723616024	1
CERT1	0.723616024	1
CFAP61	0.723616024	1
CFAP73	0.723616024	1
CFD	0.723616024	1
CHD1	0.723616024	1
CHFR	0.723616024	1

SetID	P.value	N.Marker.Test
CHMP1B,GNAL	0.723616024	1
CHRFAM7A	0.723616024	1
CHRNA3	0.723616024	1
CHRNA7	0.723616024	1
CHRNB4	0.723616024	1
CHST14	0.723616024	1
CIAO3	0.723616024	1
CIP2A	0.723616024	1
CIT	0.723616024	1
CLCA4	0.723616024	1
CLCN1	0.723616024	1
CLCN6	0.723616024	1
CLDN14	0.723616024	1
CLDN20,TFB1M	0.723616024	1
CLDN23	0.723616024	1
CLEC18A	0.723616024	1
CLIP2	0.723616024	1
CLN5	0.723616024	1
CLOCK	0.723616024	1
CLPTM1	0.723616024	1
CLSTN1	0.723616024	1
CMTR2	0.723616024	1
CNBD1	0.723616024	1
CNDP1	0.723616024	1
CNNM2	0.723616024	1
CNOT10	0.723616024	1
CNTLN	0.723616024	1
CNTN2	0.723616024	1
CNTNAP4	0.723616024	1
COG5	0.723616024	1
COG8	0.723616024	1
COG8,PDF	0.723616024	1
COL27A1	0.723616024	1
COL4A1	0.723616024	1
COMMD1	0.723616024	1
COPA	0.723616024	1
CORO7,CORO7-PA	0.723616024	1
COX10	0.723616024	1
CPA3	0.723616024	1
CPN2	0.723616024	1
CPVL	0.723616024	1
CPZ	0.723616024	1
CRB3	0.723616024	1
CREB3L2	0.723616024	1
CREBBP	0.723616024	1
CRMP1	0.723616024	1
CRX	0.723616024	1
CRYBG1	0.723616024	1
CRYBG3	0.723616024	1

SetID	P.value	N.Marker.Test
CRYM	0.723616024	1
CSF1R	0.723616024	1
CSH1	0.723616024	1
CSH2	0.723616024	1
CSRP1	0.723616024	1
CTH	0.723616024	1
CTSB	0.723616024	1
CTTN	0.723616024	1
CUL9	0.723616024	1
CWC22	0.723616024	1
CWF19L1	0.723616024	1
CYB5R4	0.723616024	1
CYBA	0.723616024	1
CYC1	0.723616024	1
CYCS	0.723616024	1
CYP11B2	0.723616024	1
CYP20A1	0.723616024	1
CYP2W1	0.723616024	1
CYP4B1	0.723616024	1
CYP7A1	0.723616024	1
DAB2	0.723616024	1
DAG1	0.723616024	1
DAP	0.723616024	1
DAPK1	0.723616024	1
DAZL	0.723616024	1
DBX1	0.723616024	1
DCAKD	0.723616024	1
DCP1B	0.723616024	1
DCPS	0.723616024	1
DCT	0.723616024	1
DCTD	0.723616024	1
DCTN1	0.723616024	1
DCTN4	0.723616024	1
DDO	0.723616024	1
DDX11	0.723616024	1
DDX17	0.723616024	1
DDX31	0.723616024	1
DDX49	0.723616024	1
DDX51	0.723616024	1
DDX55	0.723616024	1
DEF8	0.723616024	1
DENND11	0.723616024	1
DENND2A	0.723616024	1
DENND6A,PDE12	0.723616024	1
DEPTOR	0.723616024	1
DGCR2	0.723616024	1
DGKD	0.723616024	1
DGKG	0.723616024	1
DGKI	0.723616024	1

SetID	P.value	N.Marker.Test
DHCR7	0.723616024	1
DHPS	0.723616024	1
DHRS13	0.723616024	1
DHRS7	0.723616024	1
DHX38	0.723616024	1
DHX58	0.723616024	1
DHX8	0.723616024	1
DIAPH1	0.723616024	1
DIO2	0.723616024	1
DIP2B	0.723616024	1
DIPK1B	0.723616024	1
DISP1	0.723616024	1
DKK2	0.723616024	1
DLEC1	0.723616024	1
DLG1	0.723616024	1
DLG5	0.723616024	1
DLGAP1	0.723616024	1
DLX2	0.723616024	1
DLX5	0.723616024	1
DMRTC2	0.723616024	1
DMXL2	0.723616024	1
DNAAF5	0.723616024	1
DNAH11	0.723616024	1
DNAH14	0.723616024	1
DNAI1	0.723616024	1
DNAJB11	0.723616024	1
DNAJB8	0.723616024	1
DNM1L	0.723616024	1
DOCK5	0.723616024	1
DOCK9	0.723616024	1
DONSON	0.723616024	1
DPH6	0.723616024	1
DPP3	0.723616024	1
DPYS	0.723616024	1
DPYSL4	0.723616024	1
DRC1	0.723616024	1
DRC7	0.723616024	1
DRD1	0.723616024	1
DSCAM	0.723616024	1
DSCC1	0.723616024	1
DSEL	0.723616024	1
DSG1	0.723616024	1
DTD1	0.723616024	1
DTD2	0.723616024	1
DTWD2	0.723616024	1
DTX3L	0.723616024	1
DUPD1	0.723616024	1
DUS3L	0.723616024	1
DVL1	0.723616024	1

SetID	P.value	N.Marker.Test
DYNC2LI1	0.723616024	1
EAF2	0.723616024	1
EBF1	0.723616024	1
ECPAS	0.723616024	1
EDEM2	0.723616024	1
EFEMP2	0.723616024	1
EFNB3	0.723616024	1
EGF	0.723616024	1
EHBP1L1	0.723616024	1
EHD4	0.723616024	1
EIF1AD	0.723616024	1
EIF2AK4	0.723616024	1
ELOVL3	0.723616024	1
ELP6	0.723616024	1
EMC10	0.723616024	1
EML2	0.723616024	1
EML6	0.723616024	1
ENAH	0.723616024	1
ENKUR	0.723616024	1
ENKUR,THNSL1	0.723616024	1
ENOPH1	0.723616024	1
ENPP6	0.723616024	1
ENTPD1	0.723616024	1
EPB41	0.723616024	1
EPHA2	0.723616024	1
EPHA4	0.723616024	1
EPHB1	0.723616024	1
EPHB3	0.723616024	1
EPS8L2	0.723616024	1
ERCC3	0.723616024	1
ERGIC1	0.723616024	1
ERICH6	0.723616024	1
ERN1	0.723616024	1
ERN2	0.723616024	1
ESF1	0.723616024	1
ESPN	0.723616024	1
ETAA1	0.723616024	1
ETFA	0.723616024	1
EXO1	0.723616024	1
EXOSC10	0.723616024	1
EXOSC2	0.723616024	1
EXOSC4	0.723616024	1
EXOSC9	0.723616024	1
EXPH5	0.723616024	1
F13A1	0.723616024	1
FAM104A	0.723616024	1
FAM135A	0.723616024	1
FAM13B,PKD2L2	0.723616024	1
FAM13C	0.723616024	1

SetID	P.value	N.Marker.Test
FAM149B1	0.723616024	1
FAM151B	0.723616024	1
FAM160A2	0.723616024	1
FAM160B2	0.723616024	1
FAM161A	0.723616024	1
FAM161B	0.723616024	1
FAM171A1	0.723616024	1
FAM71B	0.723616024	1
FAM71F2	0.723616024	1
FAM83B	0.723616024	1
FAM83D	0.723616024	1
FAM83F	0.723616024	1
FAM98A	0.723616024	1
FAM98B	0.723616024	1
FANK1	0.723616024	1
FARP1	0.723616024	1
FASTKD2	0.723616024	1
FBLN2	0.723616024	1
FBLN7	0.723616024	1
FBXL6	0.723616024	1
FBXO2	0.723616024	1
FBXO27	0.723616024	1
FBXO30	0.723616024	1
FBXO4	0.723616024	1
FBXW4	0.723616024	1
FCSK	0.723616024	1
FDPS	0.723616024	1
FECH	0.723616024	1
FERMT3	0.723616024	1
FGF22	0.723616024	1
FGGY	0.723616024	1
FHAD1	0.723616024	1
FHL2	0.723616024	1
FHOD1	0.723616024	1
FICD	0.723616024	1
FIGNL1	0.723616024	1
FKBP15	0.723616024	1
FLCN	0.723616024	1
FLT3	0.723616024	1
FNIP2	0.723616024	1
FOXJ2	0.723616024	1
FOXP2	0.723616024	1
FOXS1	0.723616024	1
FREM1	0.723616024	1
FRYL	0.723616024	1
FUS	0.723616024	1
FUT11	0.723616024	1
FYB1	0.723616024	1
FYTTD1	0.723616024	1

SetID	P.value	N.Marker.Test
FZD3	0.723616024	1
FZD4	0.723616024	1
FZD6	0.723616024	1
G6PC	0.723616024	1
GABRG1	0.723616024	1
GABRR1	0.723616024	1
GAL	0.723616024	1
GAL3ST1	0.723616024	1
GALK2	0.723616024	1
GALR3	0.723616024	1
GALT	0.723616024	1
GBA2	0.723616024	1
GBF1	0.723616024	1
GBP5	0.723616024	1
GBP6	0.723616024	1
GCDH,SYCE2	0.723616024	1
GCKR	0.723616024	1
GCNT3	0.723616024	1
GCNT4	0.723616024	1
GDF6	0.723616024	1
GEM	0.723616024	1
GEMIN2	0.723616024	1
GGA2	0.723616024	1
GGT1,LRRC75B	0.723616024	1
GIGYF1	0.723616024	1
GINM1	0.723616024	1
GIPR	0.723616024	1
GJA10	0.723616024	1
GJC3	0.723616024	1
GLB1L	0.723616024	1
GLDN	0.723616024	1
GLI2	0.723616024	1
GLYAT	0.723616024	1
GLYATL3	0.723616024	1
GMEB2	0.723616024	1
GMIP	0.723616024	1
GMPR2	0.723616024	1
GMPS	0.723616024	1
GNE	0.723616024	1
GOT1L1	0.723616024	1
GOT2	0.723616024	1
GPATCH2	0.723616024	1
GPD1	0.723616024	1
GPR1	0.723616024	1
GPR141	0.723616024	1
GPR31	0.723616024	1
GPR84	0.723616024	1
GPRC5B	0.723616024	1
GPSM1	0.723616024	1

SetID	P.value	N.Marker.Test
GPSM2	0.723616024	1
GPX3	0.723616024	1
GRAMD2B	0.723616024	1
GREB1L	0.723616024	1
GRHL3	0.723616024	1
GRIA4	0.723616024	1
GRID2IP	0.723616024	1
GRIK2	0.723616024	1
GRIN2C	0.723616024	1
GRK4	0.723616024	1
GRK6	0.723616024	1
GRM7	0.723616024	1
GSDME	0.723616024	1
GSS	0.723616024	1
GSTM3	0.723616024	1
GSTM5	0.723616024	1
GSX2	0.723616024	1
GTDC1	0.723616024	1
GTF2IRD1	0.723616024	1
GTF3C5	0.723616024	1
GTSE1	0.723616024	1
GUCY2C	0.723616024	1
GYG1	0.723616024	1
HABP2	0.723616024	1
HAUS2	0.723616024	1
HDAC4	0.723616024	1
HDDC3,UNC45A	0.723616024	1
HEATR5A	0.723616024	1
HELLS	0.723616024	1
HELZ	0.723616024	1
HES3	0.723616024	1
HESX1	0.723616024	1
HGD	0.723616024	1
HLTF	0.723616024	1
HM13	0.723616024	1
HMOX1	0.723616024	1
HNRNPF	0.723616024	1
HNRNPL	0.723616024	1
HOMER2	0.723616024	1
HOMER3	0.723616024	1
HOXA3	0.723616024	1
HOXA7	0.723616024	1
HOXB13	0.723616024	1
HOXB3	0.723616024	1
HOXB5	0.723616024	1
HP	0.723616024	1
HPGDS	0.723616024	1
HPR	0.723616024	1
HPS4	0.723616024	1

SetID	P.value	N.Marker.Test
HS3ST3B1	0.723616024	1
HSD17B14	0.723616024	1
HSD17B4	0.723616024	1
HSP90B1	0.723616024	1
HSPA9	0.723616024	1
HTR3D	0.723616024	1
IAH1	0.723616024	1
IBTK	0.723616024	1
ID3	0.723616024	1
IER3IP1	0.723616024	1
IFI27L1	0.723616024	1
IFIT1B	0.723616024	1
IFT80	0.723616024	1
IGF1R	0.723616024	1
IGFBPL1	0.723616024	1
IKZF3	0.723616024	1
IL11RA	0.723616024	1
IL17RC	0.723616024	1
IL20RA	0.723616024	1
IL25	0.723616024	1
IL4I1,NUP62	0.723616024	1
IL4R	0.723616024	1
IL7R	0.723616024	1
ILVBL	0.723616024	1
IMMP1L	0.723616024	1
IMPDH2	0.723616024	1
INAFM1	0.723616024	1
INCENP	0.723616024	1
INTS1	0.723616024	1
INTS2	0.723616024	1
INTS7	0.723616024	1
INTU	0.723616024	1
IQCB1	0.723616024	1
IQCE	0.723616024	1
IQGAP1	0.723616024	1
IRAK1BP1	0.723616024	1
IRAK2	0.723616024	1
IRAK4	0.723616024	1
IRS2	0.723616024	1
IRX6	0.723616024	1
ISM2	0.723616024	1
ISOC2	0.723616024	1
ITGAM	0.723616024	1
ITGAV	0.723616024	1
ITGB7	0.723616024	1
ITPKC	0.723616024	1
JPH3	0.723616024	1
JUNB	0.723616024	1
KALRN	0.723616024	1

SetID	P.value	N.Marker.Test
KCNC2	0.723616024	1
KCNC4	0.723616024	1
KCNH5	0.723616024	1
KCNJ16	0.723616024	1
KCNJ5	0.723616024	1
KCNK18	0.723616024	1
KCNN4	0.723616024	1
KCNQ3	0.723616024	1
KCNRG,TRIM13	0.723616024	1
KCNS1	0.723616024	1
KDM2B	0.723616024	1
KDM4E	0.723616024	1
KDM5A	0.723616024	1
KIAA0232	0.723616024	1
KIAA0825	0.723616024	1
KIAA0930	0.723616024	1
KIAA1324	0.723616024	1
KIAA1549	0.723616024	1
KIF21A	0.723616024	1
KIF21B	0.723616024	1
KIF25	0.723616024	1
KIF2B	0.723616024	1
KIF6	0.723616024	1
KIRREL1	0.723616024	1
KL	0.723616024	1
KLC3	0.723616024	1
KLHL22	0.723616024	1
KLHL23,PHOSPHO2	0.723616024	1
KLHL30	0.723616024	1
KLHL31	0.723616024	1
KLK5	0.723616024	1
KLK8	0.723616024	1
KMT2D	0.723616024	1
KREMEN1	0.723616024	1
KRT17	0.723616024	1
KRT18	0.723616024	1
KRT24	0.723616024	1
KRT31	0.723616024	1
KRT36	0.723616024	1
KRT37	0.723616024	1
KRT5	0.723616024	1
KRT6B	0.723616024	1
KRT83	0.723616024	1
KRTAP12-2,TSPEAR	0.723616024	1
KSR2	0.723616024	1
L3MBTL1	0.723616024	1
L3MBTL2	0.723616024	1
LAMC2	0.723616024	1
LAP3	0.723616024	1

SetID	P.value	N.Marker.Test
LBP	0.723616024	1
LCMT1	0.723616024	1
LCP2	0.723616024	1
LDHAL6B,MYO1E	0.723616024	1
LDHD	0.723616024	1
LDLR	0.723616024	1
LEF1	0.723616024	1
LGALS9B	0.723616024	1
LGI2	0.723616024	1
LGI4	0.723616024	1
LGR6	0.723616024	1
LHFPL3	0.723616024	1
LHX8	0.723616024	1
LIG4	0.723616024	1
LIN37	0.723616024	1
LIPJ	0.723616024	1
LIPK	0.723616024	1
LMAN1	0.723616024	1
LMX1A	0.723616024	1
LNPK	0.723616024	1
LOXL2	0.723616024	1
LOXL4	0.723616024	1
LPCAT2	0.723616024	1
LPCAT4	0.723616024	1
LPO	0.723616024	1
LRCH1	0.723616024	1
LRFN4,PC	0.723616024	1
LRPPRC	0.723616024	1
LRRC4C	0.723616024	1
LRRC7	0.723616024	1
LRRCC1	0.723616024	1
LRRIQ4	0.723616024	1
LTBP4	0.723616024	1
LYG1	0.723616024	1
LZTR1	0.723616024	1
LZTS2	0.723616024	1
MAB21L4	0.723616024	1
MAFA	0.723616024	1
MAIP1	0.723616024	1
MAK16	0.723616024	1
MAMDC2	0.723616024	1
MAN1C1	0.723616024	1
MANBA	0.723616024	1
MAP1A	0.723616024	1
MAP1LC3B2	0.723616024	1
MAP3K5	0.723616024	1
MAP4K4	0.723616024	1
MARCHF3	0.723616024	1
MARCHF8	0.723616024	1

SetID	P.value	N.Marker.Test
MASP1	0.723616024	1
MATN2	0.723616024	1
MCM2	0.723616024	1
MCM3AP	0.723616024	1
MCM6	0.723616024	1
MCM8	0.723616024	1
MDGA1	0.723616024	1
MDM1	0.723616024	1
MEAK7	0.723616024	1
MED1	0.723616024	1
MEGF10	0.723616024	1
MEOX1	0.723616024	1
MESD	0.723616024	1
METRNL	0.723616024	1
METTL1	0.723616024	1
METTL26	0.723616024	1
METTL2A	0.723616024	1
MFSD1	0.723616024	1
MFSD13A	0.723616024	1
MFSD6L	0.723616024	1
MFSD8	0.723616024	1
MGA	0.723616024	1
MIA2	0.723616024	1
MICALL2	0.723616024	1
MIS18BP1	0.723616024	1
MLF1	0.723616024	1
MMEL1,PRXL2B	0.723616024	1
MMP8	0.723616024	1
MOGAT3	0.723616024	1
MOGS	0.723616024	1
MOK	0.723616024	1
MORN1	0.723616024	1
MOV10	0.723616024	1
MPP6	0.723616024	1
MPP7	0.723616024	1
MPRIP	0.723616024	1
MRM2	0.723616024	1
MRO	0.723616024	1
MRPL13	0.723616024	1
MRPL17	0.723616024	1
MRPL46	0.723616024	1
MRPS10	0.723616024	1
MRPS18A	0.723616024	1
MRPS30	0.723616024	1
MRPS33	0.723616024	1
MRPS6,SLC5A3	0.723616024	1
MRPS9	0.723616024	1
MS4A2	0.723616024	1
MSH2	0.723616024	1

SetID	P.value	N.Marker.Test
MSR1	0.723616024	1
MSTO1	0.723616024	1
MTAP	0.723616024	1
MTARC2	0.723616024	1
MTERF4	0.723616024	1
MTFR1	0.723616024	1
MTHFD1L	0.723616024	1
MTHFD2L	0.723616024	1
MTIF2	0.723616024	1
MTMR7	0.723616024	1
MTMR9	0.723616024	1
MTO1	0.723616024	1
MTREX	0.723616024	1
MTRF1	0.723616024	1
MTUS2	0.723616024	1
MTX2	0.723616024	1
MUC1	0.723616024	1
MX1	0.723616024	1
MYBPC2	0.723616024	1
MYBPC3	0.723616024	1
MYBPHL	0.723616024	1
MYCL	0.723616024	1
MYEF2	0.723616024	1
MYH10	0.723616024	1
MYLIP	0.723616024	1
MYLK4,WRNIP1	0.723616024	1
MYO1G	0.723616024	1
MYO5C	0.723616024	1
MYRFL	0.723616024	1
MZF1	0.723616024	1
NAA16	0.723616024	1
NADK	0.723616024	1
NAGK	0.723616024	1
NAPEPLD	0.723616024	1
NAPRT	0.723616024	1
NAPSA	0.723616024	1
NAT14	0.723616024	1
NAT8	0.723616024	1
NBEAL2	0.723616024	1
NCOA1	0.723616024	1
NCOA3	0.723616024	1
NDUFA7	0.723616024	1
NDUFAF6	0.723616024	1
NDUFAF7	0.723616024	1
NDUFB10	0.723616024	1
NDUFB6	0.723616024	1
NECTIN3	0.723616024	1
NEIL3	0.723616024	1
NEPRO	0.723616024	1

SetID	P.value	N.Marker.Test
NET1	0.723616024	1
NFATC1	0.723616024	1
NFATC2	0.723616024	1
NFE2L3	0.723616024	1
NFKBIA	0.723616024	1
NFRKB	0.723616024	1
NFU1	0.723616024	1
NFXL1	0.723616024	1
NGEF	0.723616024	1
NHP2	0.723616024	1
NIBAN1	0.723616024	1
NIBAN3	0.723616024	1
NIM1K	0.723616024	1
NIN	0.723616024	1
NIP7	0.723616024	1
NIPBL	0.723616024	1
NKTR	0.723616024	1
NME4	0.723616024	1
NME6	0.723616024	1
NMRK2	0.723616024	1
NOL4	0.723616024	1
NOLC1	0.723616024	1
NOP2	0.723616024	1
NOP53	0.723616024	1
NOVA1	0.723616024	1
NPAS1	0.723616024	1
NPC2	0.723616024	1
NPEPL1	0.723616024	1
NPFFR1	0.723616024	1
NPHP1	0.723616024	1
NPL	0.723616024	1
NPNT	0.723616024	1
NR4A1	0.723616024	1
NRBP2	0.723616024	1
NRCAM	0.723616024	1
NRL	0.723616024	1
NRTN	0.723616024	1
NSG2	0.723616024	1
NSMCE2	0.723616024	1
NSUN2	0.723616024	1
NSUN6	0.723616024	1
NTN5	0.723616024	1
NTRK1	0.723616024	1
NUP210L	0.723616024	1
NXPE2	0.723616024	1
NXPE3	0.723616024	1
NXT1	0.723616024	1
OAS2	0.723616024	1
OCLN	0.723616024	1

SetID	P.value	N.Marker.Test
ODF3	0.723616024	1
OGFOD1	0.723616024	1
OR10G7	0.723616024	1
OR10H5	0.723616024	1
OR10K2	0.723616024	1
OR10Z1	0.723616024	1
OR11H4	0.723616024	1
OR13C3	0.723616024	1
OR1B1	0.723616024	1
OR1J1	0.723616024	1
OR2D2	0.723616024	1
OR2D3	0.723616024	1
OR2V2	0.723616024	1
OR4F17	0.723616024	1
OR4F4	0.723616024	1
OR51V1	0.723616024	1
OR52B2	0.723616024	1
OR52H1	0.723616024	1
OR52N1	0.723616024	1
OR5H1	0.723616024	1
OR5K3	0.723616024	1
OR9A4	0.723616024	1
ORC5	0.723616024	1
OSBP	0.723616024	1
OSBPL1A	0.723616024	1
OSER1	0.723616024	1
P4HB	0.723616024	1
PADI1	0.723616024	1
PAOX	0.723616024	1
PAPOLG	0.723616024	1
PAPPA	0.723616024	1
PAPSS2	0.723616024	1
PARN	0.723616024	1
PARP15	0.723616024	1
PARP16	0.723616024	1
PARPBP	0.723616024	1
PAX7	0.723616024	1
PAX9	0.723616024	1
PAXIP1	0.723616024	1
PBLD	0.723616024	1
PCBP4	0.723616024	1
PCDHA1,PCDHA2,P	0.723616024	1
PCDHB12	0.723616024	1
PCDHB6	0.723616024	1
PCDHGA1,PCDHGA	0.723616024	1
PCDHGA1,PCDHGA	0.723616024	1
PCDHGA1,PCDHGA	0.723616024	1
PCDHGA1,PCDHGA	0.723616024	1
PCDHGA1,PCDHGA	0.723616024	1

SetID	P.value	N.Marker.Test
PCDHGA1,PCDHGA	0.723616024	1
PCGF6	0.723616024	1
PCNT	0.723616024	1
PCOLCE	0.723616024	1
PCOLCE2	0.723616024	1
PDE11A	0.723616024	1
PDE6B	0.723616024	1
PDIA2	0.723616024	1
PDIA4	0.723616024	1
PDILT	0.723616024	1
PDLIM3	0.723616024	1
PDX1	0.723616024	1
PDXK	0.723616024	1
PDYN	0.723616024	1
PDZRN3	0.723616024	1
PEBP4	0.723616024	1
PEG3,ZIM2	0.723616024	1
PER2	0.723616024	1
PEX11A	0.723616024	1
PEX14	0.723616024	1
PEX7	0.723616024	1
PFKFB3	0.723616024	1
PFKP	0.723616024	1
PGAP1	0.723616024	1
PGAP3	0.723616024	1
PGGHG	0.723616024	1
PGM2	0.723616024	1
PGR	0.723616024	1
PHACTR4	0.723616024	1
PHRF1	0.723616024	1
PHYH	0.723616024	1
PI4KA,SERPIND1	0.723616024	1
PIGK	0.723616024	1
PIGN	0.723616024	1
PIGS	0.723616024	1
PIGZ	0.723616024	1
PIK3C2G	0.723616024	1
PIWIL2	0.723616024	1
PIWIL4	0.723616024	1
PKD2	0.723616024	1
PKD2L2	0.723616024	1
PLAA	0.723616024	1
PLBD2	0.723616024	1
PLCH2	0.723616024	1
PLD3	0.723616024	1
PLD4	0.723616024	1
PLEK2	0.723616024	1
PLEKHA4	0.723616024	1
PLEKHA5	0.723616024	1

SetID	P.value	N.Marker.Test
PLEKHB2	0.723616024	1
PLEKHG3	0.723616024	1
PLEKHG5	0.723616024	1
PLEKHG7	0.723616024	1
PLSCR1	0.723616024	1
PLXNC1	0.723616024	1
PMM1	0.723616024	1
PMP2	0.723616024	1
PMPCB	0.723616024	1
PNKD	0.723616024	1
PNKP	0.723616024	1
PNLDC1	0.723616024	1
PNPLA2	0.723616024	1
PNPLA8	0.723616024	1
POLD2	0.723616024	1
POLK	0.723616024	1
POLN	0.723616024	1
POMGNT1	0.723616024	1
POTEI	0.723616024	1
POU2F1	0.723616024	1
PPFIA2	0.723616024	1
PPFIA3	0.723616024	1
PPIH	0.723616024	1
PPIL2	0.723616024	1
PPM1F	0.723616024	1
PPM1M	0.723616024	1
PPP1R16A	0.723616024	1
PPP1R37	0.723616024	1
PPP1R3A	0.723616024	1
PPP2R1B	0.723616024	1
PPP2R5D	0.723616024	1
PPP2R5E	0.723616024	1
PRDM16	0.723616024	1
PREX1	0.723616024	1
PRKAR1B	0.723616024	1
PRKAR2A	0.723616024	1
PRKD1	0.723616024	1
PRKG1	0.723616024	1
PRKN	0.723616024	1
PRLHR	0.723616024	1
PRODH2	0.723616024	1
PROK1	0.723616024	1
PRORP	0.723616024	1
PRPF3	0.723616024	1
PRPSAP1	0.723616024	1
PRR5,PRR5-ARHGA	0.723616024	1
PRRC2B	0.723616024	1
PRSS42P	0.723616024	1
PSD2	0.723616024	1

SetID	P.value	N.Marker.Test
PSD3	0.723616024	1
PSEN2	0.723616024	1
PSMC3	0.723616024	1
PSMD13	0.723616024	1
PSME4	0.723616024	1
PSMF1	0.723616024	1
PSMG1	0.723616024	1
PTAFR	0.723616024	1
PTGR1,ZNF483	0.723616024	1
PTGR2	0.723616024	1
PTGS1	0.723616024	1
PTPN3	0.723616024	1
PTPRD	0.723616024	1
PTPRR	0.723616024	1
PTPRU	0.723616024	1
PVR	0.723616024	1
QARS1	0.723616024	1
QTRT2	0.723616024	1
RAB11FIP2	0.723616024	1
RAB11FIP5	0.723616024	1
RAB26	0.723616024	1
RAB28	0.723616024	1
RAB3GAP1	0.723616024	1
RAB3GAP2	0.723616024	1
RABEP1	0.723616024	1
RABGGTA	0.723616024	1
RAD50	0.723616024	1
RAD51D	0.723616024	1
RAI1	0.723616024	1
RAMP1	0.723616024	1
RANBP10	0.723616024	1
RANBP3L	0.723616024	1
RASA1	0.723616024	1
RASA3	0.723616024	1
RASAL2	0.723616024	1
RASGRP1	0.723616024	1
RASSF6	0.723616024	1
RBL1	0.723616024	1
RBM34	0.723616024	1
RBM45	0.723616024	1
RBM46	0.723616024	1
RBMXL2	0.723616024	1
RBP1	0.723616024	1
RCBTB2	0.723616024	1
RCL1	0.723616024	1
RCSD1	0.723616024	1
REEP4	0.723616024	1
RFTN2	0.723616024	1
RGMB	0.723616024	1

SetID	P.value	N.Marker.Test
RGS18	0.723616024	1
RGS22	0.723616024	1
RGS8	0.723616024	1
RHOD	0.723616024	1
RHOJ	0.723616024	1
RIC3	0.723616024	1
RINT1	0.723616024	1
RIOK2	0.723616024	1
RMDN2	0.723616024	1
RMDN3	0.723616024	1
RMI1	0.723616024	1
RMND5B	0.723616024	1
RNF125	0.723616024	1
RNF151	0.723616024	1
RNF175	0.723616024	1
RNF19A	0.723616024	1
RNF31	0.723616024	1
RNF34	0.723616024	1
RNLS	0.723616024	1
ROBO4	0.723616024	1
ROS1	0.723616024	1
RPL10L	0.723616024	1
RPP38	0.723616024	1
RPUSD1	0.723616024	1
RPUSD2	0.723616024	1
RRM1	0.723616024	1
RRNAD1	0.723616024	1
RRP9	0.723616024	1
RTP2	0.723616024	1
RUSF1	0.723616024	1
RWDD3	0.723616024	1
RXFP4	0.723616024	1
RYR2	0.723616024	1
SAMSN1	0.723616024	1
SARAF	0.723616024	1
SATB1	0.723616024	1
SBNO1	0.723616024	1
SC5D	0.723616024	1
SCLY	0.723616024	1
SCN8A	0.723616024	1
SCRIB	0.723616024	1
SCUBE3	0.723616024	1
SCYL2	0.723616024	1
SDC2	0.723616024	1
SDHA	0.723616024	1
SDK2	0.723616024	1
SDSL	0.723616024	1
SEC14L2	0.723616024	1
SEC16A	0.723616024	1

SetID	P.value	N.Marker.Test
SEC16B	0.723616024	1
SEC24D	0.723616024	1
SECISBP2	0.723616024	1
SELP	0.723616024	1
SEMA3E	0.723616024	1
SEMA3G	0.723616024	1
SEMA4A	0.723616024	1
SEMA6A	0.723616024	1
SEMA6D	0.723616024	1
SEPTIN4	0.723616024	1
SERAC1	0.723616024	1
SERINC4	0.723616024	1
SERPINA10	0.723616024	1
SERPINB1	0.723616024	1
SERPINB5	0.723616024	1
SERPINB6	0.723616024	1
SERPINE3	0.723616024	1
SERPINH1	0.723616024	1
SETBP1	0.723616024	1
SETD1B	0.723616024	1
SETD2	0.723616024	1
SETDB1	0.723616024	1
SEZ6	0.723616024	1
SFXN5	0.723616024	1
SGCG	0.723616024	1
SGK2	0.723616024	1
SGSM3	0.723616024	1
SH3PXD2B	0.723616024	1
SHCBP1L	0.723616024	1
SHE	0.723616024	1
SHQ1	0.723616024	1
SIDT1	0.723616024	1
SIGLEC15	0.723616024	1
SIK2	0.723616024	1
SIPA1L3	0.723616024	1
SIRT1	0.723616024	1
SIVA1	0.723616024	1
SLA,TG	0.723616024	1
SLAIN2	0.723616024	1
SLC11A1	0.723616024	1
SLC12A4	0.723616024	1
SLC12A8	0.723616024	1
SLC12A9	0.723616024	1
SLC14A2	0.723616024	1
SLC15A4	0.723616024	1
SLC16A8	0.723616024	1
SLC17A1	0.723616024	1
SLC18B1	0.723616024	1
SLC22A11	0.723616024	1

SetID	P.value	N.Marker.Test
SLC22A13	0.723616024	1
SLC22A18	0.723616024	1
SLC22A2	0.723616024	1
SLC22A5	0.723616024	1
SLC22A6	0.723616024	1
SLC24A2	0.723616024	1
SLC24A3	0.723616024	1
SLC25A2	0.723616024	1
SLC25A31	0.723616024	1
SLC25A32	0.723616024	1
SLC25A4	0.723616024	1
SLC26A6	0.723616024	1
SLC27A2	0.723616024	1
SLC2A11	0.723616024	1
SLC2A7	0.723616024	1
SLC30A10	0.723616024	1
SLC30A2	0.723616024	1
SLC30A8	0.723616024	1
SLC35D3	0.723616024	1
SLC36A4	0.723616024	1
SLC38A8	0.723616024	1
SLC38A9	0.723616024	1
SLC39A11	0.723616024	1
SLC44A3	0.723616024	1
SLC45A4	0.723616024	1
SLC4A4	0.723616024	1
SLC4A9	0.723616024	1
SLC6A1	0.723616024	1
SLC6A12	0.723616024	1
SLC6A3	0.723616024	1
SLC7A10	0.723616024	1
SLC7A14	0.723616024	1
SLC7A5	0.723616024	1
SLC7A9	0.723616024	1
SLC9A3R1	0.723616024	1
SLC9B1	0.723616024	1
SLCO1B7	0.723616024	1
SLCO2B1	0.723616024	1
SLCO4A1	0.723616024	1
SLCO6A1	0.723616024	1
SLIRP	0.723616024	1
SLMAP	0.723616024	1
SMC2	0.723616024	1
SMC4	0.723616024	1
SMPD1	0.723616024	1
SMPD4	0.723616024	1
SMPDL3B	0.723616024	1
SMTNL2	0.723616024	1
SNAI3	0.723616024	1

SetID	P.value	N.Marker.Test
SNTB1	0.723616024	1
SNX31	0.723616024	1
SNX5	0.723616024	1
SOD3	0.723616024	1
SOS1	0.723616024	1
SOX5	0.723616024	1
SP140L	0.723616024	1
SPAG4	0.723616024	1
SPDL1	0.723616024	1
SPECC1	0.723616024	1
SPG7	0.723616024	1
SPICE1	0.723616024	1
SPIDR	0.723616024	1
SPINK6	0.723616024	1
SPINT1	0.723616024	1
SPNS3	0.723616024	1
SPON2	0.723616024	1
SPOUT1	0.723616024	1
SPP1	0.723616024	1
SPR	0.723616024	1
SPTLC2	0.723616024	1
SRA1	0.723616024	1
SRBD1	0.723616024	1
SRGAP3	0.723616024	1
SRR,TSR1	0.723616024	1
SSB	0.723616024	1
SSBP3	0.723616024	1
SSBP4	0.723616024	1
ST13	0.723616024	1
ST14	0.723616024	1
ST6GALNAC3	0.723616024	1
STAB2	0.723616024	1
STARD4	0.723616024	1
STAU2	0.723616024	1
STEAP4	0.723616024	1
STOX1	0.723616024	1
STRIP2	0.723616024	1
STX11	0.723616024	1
STX16	0.723616024	1
STX2	0.723616024	1
STX5	0.723616024	1
STX6	0.723616024	1
STXBP3	0.723616024	1
SUCLG2	0.723616024	1
SUGCT	0.723616024	1
SUGP2	0.723616024	1
SULT4A1	0.723616024	1
SUMF2	0.723616024	1
SUN2	0.723616024	1

SetID	P.value	N.Marker.Test
SUPT3H	0.723616024	1
SUSD1	0.723616024	1
SUSD5	0.723616024	1
SVEP1	0.723616024	1
SYBU	0.723616024	1
SYNGR1	0.723616024	1
SYT2	0.723616024	1
SYT9	0.723616024	1
TAAR5	0.723616024	1
TAF1B	0.723616024	1
TAF6	0.723616024	1
TAL2	0.723616024	1
TALDO1	0.723616024	1
TAOK3	0.723616024	1
TAS1R2	0.723616024	1
TATDN3	0.723616024	1
TBC1D10A	0.723616024	1
TBL3	0.723616024	1
TBX3	0.723616024	1
TCAF1	0.723616024	1
TCF12	0.723616024	1
TCF7	0.723616024	1
TCIRG1	0.723616024	1
TDP1	0.723616024	1
TDRD6	0.723616024	1
TEAD3	0.723616024	1
TEKT2	0.723616024	1
TEP1	0.723616024	1
TEX14	0.723616024	1
TFPT	0.723616024	1
TGFBR3	0.723616024	1
TGM1	0.723616024	1
THADA	0.723616024	1
THAP3	0.723616024	1
THEM4	0.723616024	1
THOP1	0.723616024	1
THSD7B	0.723616024	1
THUMPD3	0.723616024	1
TIAM2	0.723616024	1
TIMELESS	0.723616024	1
TIMM50	0.723616024	1
TLCD5	0.723616024	1
TLE1	0.723616024	1
TLL2	0.723616024	1
TLR10	0.723616024	1
TLR2	0.723616024	1
TLR3	0.723616024	1
TLR4	0.723616024	1
TM4SF1	0.723616024	1

SetID	P.value	N.Marker.Test
TMC3	0.723616024	1
TMC8	0.723616024	1
TMCC3	0.723616024	1
TMEM119	0.723616024	1
TMEM126A	0.723616024	1
TMEM131L	0.723616024	1
TMEM161A	0.723616024	1
TMEM171	0.723616024	1
TMEM186	0.723616024	1
TMEM230	0.723616024	1
TMEM231	0.723616024	1
TMEM42	0.723616024	1
TMEM52B	0.723616024	1
TMEM87A	0.723616024	1
TMEM91	0.723616024	1
TMOD4	0.723616024	1
TMUB2	0.723616024	1
TNFRSF11B	0.723616024	1
TNNI2	0.723616024	1
TNNT3	0.723616024	1
TNRC18	0.723616024	1
TOMM40	0.723616024	1
TOP2A	0.723616024	1
TOP3A	0.723616024	1
TOR3A	0.723616024	1
TP53BP1	0.723616024	1
TPD52	0.723616024	1
TPRN	0.723616024	1
TRAF3IP2	0.723616024	1
TRAP1	0.723616024	1
TRAPPC6B	0.723616024	1
TRAPPC8	0.723616024	1
TRH	0.723616024	1
TRIM14	0.723616024	1
TRIM16	0.723616024	1
TRIM6,TRIM6-TRII	0.723616024	1
TRIM63	0.723616024	1
TRIM66	0.723616024	1
TRIP12	0.723616024	1
TRMT2A	0.723616024	1
TRMT44	0.723616024	1
TRPA1	0.723616024	1
TRPC4	0.723616024	1
TRPM5	0.723616024	1
TRPM6	0.723616024	1
TRPT1	0.723616024	1
TRPV1	0.723616024	1
TSHR	0.723616024	1
TSPOAP1	0.723616024	1

SetID	P.value	N.Marker.Test
TSSC4	0.723616024	1
TTC23	0.723616024	1
TTC26	0.723616024	1
TTC30A	0.723616024	1
TTC7B	0.723616024	1
TTF2	0.723616024	1
TTI1	0.723616024	1
TTL	0.723616024	1
TUBAL3	0.723616024	1
TUBG2	0.723616024	1
TUBGCP2	0.723616024	1
TXN	0.723616024	1
TXNDC5	0.723616024	1
TYR	0.723616024	1
TYRO3	0.723616024	1
UAP1L1	0.723616024	1
UBASH3A	0.723616024	1
UBE2Q2	0.723616024	1
UBN2	0.723616024	1
UBQLN3	0.723616024	1
UBR3	0.723616024	1
UBXN6	0.723616024	1
UFC1	0.723616024	1
UNC5B	0.723616024	1
UNC5D	0.723616024	1
UNC93A	0.723616024	1
UNCX	0.723616024	1
UPB1	0.723616024	1
UQCRB	0.723616024	1
URB2	0.723616024	1
USP13	0.723616024	1
USP28	0.723616024	1
USP29	0.723616024	1
USP44	0.723616024	1
USP45	0.723616024	1
USP47	0.723616024	1
UTP18	0.723616024	1
UTP25	0.723616024	1
VAV3	0.723616024	1
VDR	0.723616024	1
VEGFB	0.723616024	1
VEZT	0.723616024	1
VMP1	0.723616024	1
VNN1	0.723616024	1
VPREB3	0.723616024	1
VPS13B	0.723616024	1
VPS35L	0.723616024	1
VPS4B	0.723616024	1
VPS9D1	0.723616024	1

SetID	P.value	N.Marker.Test
VRK2	0.723616024	1
VSIG10	0.723616024	1
VWA3B	0.723616024	1
WARS1	0.723616024	1
WASL	0.723616024	1
WDR19	0.723616024	1
WDR20	0.723616024	1
WDR33	0.723616024	1
WDR48	0.723616024	1
WDR64	0.723616024	1
WDR7	0.723616024	1
WDR83,WDR83OS	0.723616024	1
WIPF1	0.723616024	1
WRAP53	0.723616024	1
WWC1	0.723616024	1
WWC2	0.723616024	1
XRCC1	0.723616024	1
ZBTB40	0.723616024	1
ZBTB42	0.723616024	1
ZC3H12C	0.723616024	1
ZC3HC1	0.723616024	1
ZFAND1	0.723616024	1
ZFYVE26	0.723616024	1
ZFYVE9	0.723616024	1
ZKSCAN5	0.723616024	1
ZMYM5	0.723616024	1
ZNF138	0.723616024	1
ZNF146	0.723616024	1
ZNF226	0.723616024	1
ZNF268	0.723616024	1
ZNF281	0.723616024	1
ZNF337	0.723616024	1
ZNF365	0.723616024	1
ZNF426	0.723616024	1
ZNF439	0.723616024	1
ZNF440	0.723616024	1
ZNF467	0.723616024	1
ZNF491	0.723616024	1
ZNF506	0.723616024	1
ZNF507	0.723616024	1
ZNF511	0.723616024	1
ZNF514	0.723616024	1
ZNF521	0.723616024	1
ZNF534	0.723616024	1
ZNF585A	0.723616024	1
ZNF621	0.723616024	1
ZNF641	0.723616024	1
ZNF708	0.723616024	1
ZNF780A	0.723616024	1

SetID	P.value	N.Marker.Test
ZNF799	0.723616024	1
ZRANB3	0.723616024	1
ZSCAN20	0.723616024	1
ZSCAN29	0.723616024	1
ZSCAN5B	0.723616024	1
ZW10	0.723616024	1
OR4C16	0.724084136	1
ACTL7A	0.726327637	2
CIC	0.726327637	2
DNAJC5B	0.726327637	2
LRWD1	0.726327637	2
MOCS1	0.726327637	2
MTG2	0.726327637	2
NOTCH2	0.726327637	2
PCDHA1,PCDHA2,I	0.726327637	2
PEAR1	0.726327637	2
RPL18A	0.726327637	2
SERINC2	0.726327637	2
SLC22A14	0.726327637	2
WDR60	0.726327637	2
ABCC1	0.726327637	2
ADAMTS14	0.726327637	2
AGL	0.726327637	2
BBX	0.726327637	2
BRAT1	0.726327637	2
CDRT1	0.726327637	2
CYP2C8	0.726327637	2
FSTL5	0.726327637	2
HSPB2	0.726327637	2
KDM5B	0.726327637	2
KIAA1217	0.726327637	2
KIF23	0.726327637	2
MAD1L1	0.726327637	2
MELK	0.726327637	2
MTMR3	0.726327637	2
NCAPD3	0.726327637	2
NOL6	0.726327637	2
PALB2	0.726327637	2
PCNX4	0.726327637	2
PKP3	0.726327637	2
RPGRIP1	0.726327637	2
SCN7A	0.726327637	2
SERPINB11	0.726327637	2
SHCBP1	0.726327637	2
SLC26A9	0.726327637	2
SMO	0.726327637	2
SPTB	0.726327637	2
TBC1D8	0.726327637	2
TGM5	0.726327637	2

SetID	P.value	N.Marker.Test
TIMM21	0.726327637	2
TOM1	0.726327637	2
TRNT1	0.726327637	2
UBXN1	0.726327637	2
USP6	0.726327637	2
BDP1	0.726327637	2
FAM3C	0.726327637	2
GREB1	0.726327637	2
MAN2C1	0.726327637	2
PLG	0.726327637	2
TLR1	0.726327637	2
XAB2	0.726327637	2
ARAP3	0.726327637	2
CC2D1B	0.726327637	2
COL5A2	0.726327637	2
FKBP10	0.726327637	2
RHOT2	0.726327637	2
TMC2	0.726327637	2
ACTN4	0.726327637	2
ADD3	0.726327637	2
ATAD5	0.726327637	2
CCDC13	0.726327637	2
CWH43	0.726327637	2
EFHD1	0.726327637	2
EML5	0.726327637	2
FAM135B	0.726327637	2
FAM221A	0.726327637	2
FBXL13,LRRC17	0.726327637	2
FOXD4	0.726327637	2
GPR17,LIMS2	0.726327637	2
IFIH1	0.726327637	2
INPPL1	0.726327637	2
KIF26B	0.726327637	2
KLHL25	0.726327637	2
LAMB2	0.726327637	2
LSG1	0.726327637	2
NUP85	0.726327637	2
PLA2G4E	0.726327637	2
POR	0.726327637	2
RTN4	0.726327637	2
SLC5A4	0.726327637	2
STARD3	0.726327637	2
TAPBPL	0.726327637	2
TBX10	0.726327637	2
WWOX	0.726327637	2
ZMIZ2	0.726327637	2
ZSWIM6	0.726327637	2
HAPLN3	0.726327637	2
LONP2	0.726327637	2

SetID	P.value	N.Marker.Test
GSC	0.7279228	1
VSIG2	0.729010022	1
PDPR	0.730841287	2
DNAH7	0.731994171	10
AQP12A	0.733253346	1
TENM2	0.739833509	3
TRPV4	0.742575245	2
ATPSCKMT	0.742807245	2
FAN1	0.742827136	2
RTTN	0.745267676	2
EHMT1	0.746354172	2
XDH	0.747086478	7
UTP4	0.747403751	2
SYNE1	0.754664548	8
TBC1D9B	0.756939253	5
CCDC136	0.757276522	5
LTBP1	0.760832876	3
GANC	0.762840028	3
MYO18B	0.763354325	6
CHRNG	0.777210484	2
NUP210	0.784283729	3
ACACB	0.788927896	4
PKHD1L1	0.793118937	4
BRCA2	0.795009695	3
FAT1	0.798450262	5
DNAH8	0.805192574	8
AGTR1	0.808082062	1
EIF2A	0.808082062	1
GAB2	0.808082062	1
GOLGA5	0.808082062	1
MAG	0.808082062	1
NEU2	0.808082062	1
NUDT19	0.808082062	1
PEX2	0.808082062	1
PLA2G12A	0.808082062	1
PLEKHO2	0.808082062	1
SLC22A16	0.808082062	1
SYCP2	0.808082062	1
XRN1	0.808082062	1
SCN10A	0.811539193	7
KIAA0556	0.812063831	4
FAT3	0.812627787	5
COL6A2	0.813109289	5
WDR90	0.813672948	4
ADAM28	0.815693395	3
DSC3	0.815693395	3
ZFHX4	0.815693395	3
RGS12	0.815693395	3
DNAJC10	0.818561309	4

SetID	P.value	N.Marker.Test
SVIL	0.830764951	7
CACNA1S	0.838762309	8
TLE2	0.839674974	2
EXT2	0.839852356	3
RIOX2	0.840324127	2
NWD1	0.84122194	2
XIRP1	0.841263316	2
LMO7	0.841402071	3
KCNH7	0.842732603	2
METTL21C	0.843422928	2
TP53I13	0.843921209	2
SLC7A13	0.844115574	3
SGSM2	0.844596886	2
HSD3B7	0.844852958	2
TIRAP	0.844998793	2
LRP1B	0.845053115	2
CYP3A4	0.845061077	2
ITGB6	0.845127251	2
GUSB	0.845150265	2
PCK2	0.84530409	2
DRC3	0.845311618	3
MTBP	0.845353051	2
WDR86	0.845365006	2
LMF2	0.845427846	2
APBA2	0.845461153	2
ATIC	0.845755277	2
MYH3	0.846497508	5
SHANK2	0.856583487	3
KRT26	0.857228924	3
SLC22A1	0.860462317	6
KRT25	0.860503529	3
CFAP100	0.861018412	3
CHD9	0.86107248	3
EHBP1	0.861396734	3
ABCA13	0.861451151	3
CFAP53	0.861530746	3
ERBB3	0.861530746	3
LIG1	0.861530746	3
NOTCH3	0.861530746	3
CAPN10	0.861530746	3
CAPN9	0.861530746	3
DNAH9	0.861530746	3
DOCK1	0.861530746	3
OBSL1	0.861530746	3
POTEF	0.861530746	3
SOX30	0.861530746	3
LONP1	0.864148189	3
JMJD7-PLA2G4B,P	0.866349262	5
ABCB5	0.866777219	3

SetID	P.value	N.Marker.Test
CEP162	0.870867458	5
AHNAK	0.871511525	3
FYCO1	0.882562091	4
BMP3	0.882901853	3
MASP2	0.884232801	2
THBS3	0.89118274	4
ESR1	0.891987296	3
MAST2	0.893469666	3
FAAH	0.894240221	3
OR51B5	0.901189072	2
TMPRSS9	0.901341854	2
TYK2	0.901481723	2
HYDIN	0.906802565	7
SCN9A	0.913686667	4
RYR1	0.915541364	9
BSN	0.920314646	4
KRT15	0.920314646	4
HAAO	0.921993622	2
KCNJ14	0.922014258	2
SCUBE2	0.922249009	2
POMC	0.922388053	2
WDR63	0.922618328	2
CHTF18	0.928494883	3
SLC24A4	0.928810977	3
CFI	0.934084094	4
A2ML1	1	0
A4GALT	1	0
A4GNT	1	0
AAAS	1	0
AAR2	1	0
AASDHPPT	1	0
AASS	1	0
ABCA2	1	0
ABCB11	1	0
ABCC3	1	0
ABCC9	1	0
ABCD3	1	0
ABHD14A-ACY1,AC	1	0
ABHD5	1	0
ABHD8	1	0
ABL1	1	0
ABLIM3	1	0
ABTB2	1	0
ACAD11,UBA5	1	0
ACADM	1	0
ACADS	1	0
ACADSB	1	0
ACAP1	1	0
ACAT1	1	0

SetID	P.value	N.Marker.Test
ACHE	1	0
ACKR2	1	0
ACO2,POLR3H	1	0
ACOT2	1	0
ACOT4	1	0
ACOT8	1	0
ACP5	1	0
ACSM2A	1	0
ACTA1	1	0
ACTA2	1	0
ACTB	1	0
ACTC1	1	0
ACTG1	1	0
ACTL10,NECAB3	1	0
ACTL8	1	0
ACTR10	1	0
ACTR8	1	0
ACVR1	1	0
ACVRL1	1	0
ADAD1	1	0
ADAM10	1	0
ADAM32	1	0
ADAMTSL2	1	0
ADGRB2	1	0
ADGRG1	1	0
ADGRG7	1	0
ADGRL3	1	0
ADH1A	1	0
ADHFE1	1	0
ADK	1	0
ADRA1D	1	0
ADRB1	1	0
ADRB3	1	0
ADSL	1	0
AEBP2	1	0
AFF3	1	0
AFF4	1	0
AFG3L2	1	0
AFMID	1	0
AGFG2	1	0
AGPS	1	0
AGRP	1	0
AGT	1	0
AHI1	1	0
AICDA	1	0
AIFM2	1	0
AJM1	1	0
AK1	1	0
AK2	1	0

SetID	P.value	N.Marker.Test
AK7	1	2
AK8	1	0
AKAP10	1	0
AKR1B10	1	0
AKR1B15	1	0
AKR1C3	1	0
AKR1D1	1	0
AKT1	1	0
AKT2	1	0
AKT3	1	0
ALAS1	1	0
ALDH18A1	1	0
ALDH1A3	1	0
ALDH1L1	1	0
ALDH2	1	0
ALDH6A1	1	0
ALDH6A1,BBOF1	1	0
ALDOA	1	0
ALG1	1	0
ALG10	1	0
ALG11	1	0
ALG11,UTP14C	1	0
ALG12	1	0
ALG14	1	0
ALG2	1	0
ALG3	1	0
ALG6	1	0
ALKBH1	1	0
ALKBH6	1	0
ALKBH8	1	2
ALOX5	1	0
ALPK1	1	4
ALPK3	1	0
ALS2	1	0
ALX4	1	0
AMACR	1	0
AMH	1	0
AMN	1	0
AMT	1	0
AMY2A	1	0
AMZ2	1	0
ANAPC1	1	0
ANAPC10	1	0
ANG,RNASE4	1	0
ANK1	1	0
ANKH	1	0
ANKHD1,ANKHD1-	1	0
ANKRD1	1	0
ANKRD13A	1	0

SetID	P.value	N.Marker.Test
ANKRD13C	1	0
ANKRD24	1	0
ANKRD26	1	0
ANKRD50	1	0
ANKRD65	1	0
ANKS3	1	0
ANKS6	1	0
ANO5	1	0
ANO7	1	0
ANO9	1	0
ANPEP	1	0
ANTXR2	1	0
AOPEP	1	0
AP2A2	1	0
AP2S1	1	0
AP3B1	1	0
AP3B2	1	0
AP3M2	1	0
AP4E1	1	0
AP4M1	1	0
APAF1	1	0
APBA3	1	0
APBB1IP	1	0
APC	1	0
APOA1	1	0
APOC2	1	0
APOE	1	0
APP	1	0
APPL1	1	0
APRT	1	0
AQP2	1	0
AQP5	1	0
ARAP2	1	0
ARF4,PDE12	1	0
ARFGAP3	1	0
ARFGEF1	1	0
ARFGEF2	1	0
ARG1	1	0
ARG1,MED23	1	0
ARHGAP10	1	0
ARHGAP26	1	0
ARHGAP33	1	0
ARHGEF19	1	0
ARHGEF28	1	0
ARID1B	1	0
ARID3B	1	0
ARL14EP	1	0
ARL2BP	1	0
ARL6	1	0

SetID	P.value	N.Marker.Test
ARMC2	1	0
ARMC6	1	0
ARMC9	1	0
ARSA	1	0
ARSB	1	0
ARSI	1	0
ART1	1	0
ARV1	1	0
ASAP3	1	0
ASB10	1	0
ASB5	1	0
ASH1L	1	0
ASTN2,TRIM32	1	0
ATAD2	1	0
ATF6	1	0
ATF7	1	0
ATG2A	1	0
ATG9A	1	0
ATL1	1	0
ATL3	1	0
ATM	1	0
ATN1	1	0
ATP13A1	1	0
ATP13A2	1	0
ATP1A1	1	0
ATP2A1	1	0
ATP2A2	1	0
ATP2C1	1	0
ATP5F1A	1	0
ATP5F1E	1	0
ATP5MC1	1	0
ATP6AP1L	1	0
ATP6V0A1	1	0
ATP6V0A4	1	0
ATP6V1B2	1	0
ATP8A2	1	0
ATP8B1	1	0
ATP8B1,LOC10050	1	0
ATPAF2	1	0
ATR	1	0
ATRNL1	1	0
ATXN2	1	0
ATXN3	1	0
AUH	1	0
AVP	1	0
AXDND1	1	0
AXIN2	1	0
B3GALNT2	1	0
B3GALT6	1	0

SetID	P.value	N.Marker.Test
B3GAT3	1	0
B3GLCT	1	0
B3GNT5,MCF2L2	1	0
B3GNT7	1	0
B3GNT8	1	0
B4GALT7	1	0
B4GAT1	1	0
B9D1	1	0
B9D2	1	0
BAAT	1	0
BAG2	1	0
BAG3	1	0
BAIAP2L2	1	0
BAIAP3	1	0
BAP1	1	0
BARD1	1	0
BARX1	1	0
BAX	1	0
BBS4	1	0
BBS5	1	0
BBS9	1	0
BCKDHA	1	0
BCKDK	1	0
BCL9L	1	0
BCR	1	0
BCS1L	1	0
BDH2	1	0
BEGAIN	1	0
BFAR	1	0
BFSP1	1	0
BFSP2	1	0
BHLHA9	1	0
BHLHE22	1	0
BICD2	1	0
BICRA	1	0
BIN1	1	0
BLNK	1	0
BLOC1S6	1	0
BLVRA	1	0
BMP1	1	0
BMP2	1	0
BMP2K	1	0
BMP4	1	0
BMPER	1	0
BMPR1A	1	0
BMPR2	1	0
BMS1	1	0
BNC2	1	0
BOK	1	0

SetID	P.value	N.Marker.Test
BPGM	1	0
BPNT2	1	0
BRAF	1	0
BRINP1	1	0
BRINP3	1	0
BRSK2	1	0
BSND	1	0
BTBD17	1	0
C10orf90	1	0
C12orf65	1	0
C15orf41	1	0
C17orf107,CHRNE	1	0
C19orf12	1	0
C19orf38	1	0
C19orf47	1	0
C1orf131	1	0
C1orf35	1	0
C1QL1	1	0
C1QTNF4	1	0
C1QTNF5,MFRP	1	0
C2CD2L	1	0
C2CD3	1	0
C5orf66,PITX1	1	0
C7orf31	1	0
C8orf37	1	0
C8orf82	1	0
CA1	1	0
CA2	1	0
CA4	1	0
CA9	1	0
CABP2	1	0
CACNA2D1	1	0
CACNB4	1	0
CADM3	1	0
CADPS	1	0
CALHM6	1	0
CALR3	1	0
CAMK2A	1	0
CAMK2G	1	0
CAMSAP3	1	0
CAND1	1	0
CAPN7	1	0
CAPS	1	0
CARD11	1	0
CARD14	1	0
CARD9	1	0
CARHSP1	1	0
CARTPT	1	0
CASP9	1	0

SetID	P.value	N.Marker.Test
CASQ2	1	0
CASR	1	0
CASTOR3,STAG3	1	0
CATSPERG	1	0
CAV3	1	0
CAVIN1	1	0
CBL	1	0
CCBE1	1	0
CCDC102B	1	0
CCDC103	1	0
CCDC115	1	0
CCDC122	1	0
CCDC130	1	0
CCDC148	1	0
CCDC149	1	0
CCDC174	1	0
CCDC180	1	2
CCDC186	1	0
CCDC50	1	0
CCDC85A	1	0
CCDC88A	1	0
CCDC88B	1	0
CCDC93	1	0
CCKAR	1	0
CCL27	1	0
CCN4	1	0
CCN6	1	0
CCND1	1	0
CCND2	1	0
CCND3	1	0
CCR9,LZTFL1	1	0
CCS	1	0
CCT4	1	0
CCT7	1	0
CD151	1	0
CD180	1	0
CD2	1	0
CD27	1	0
CD37	1	0
CD40	1	0
CD46	1	0
CD59	1	0
CD79A	1	0
CD79B	1	0
CD8A	1	0
CDC23	1	0
CDC27	1	0
CDC42	1	0
CDC42BPA	1	0

SetID	P.value	N.Marker.Test
CDC42EP5	1	0
CDC6	1	0
CDC73	1	0
CDCA4	1	0
CDH1	1	0
CDH13	1	0
CDH18	1	0
CDH22	1	0
CDH3	1	0
CDH4	1	0
CDK12	1	0
CDK5R1	1	0
CDK5RAP1	1	0
CDKN1A	1	0
CDKN1B	1	0
CDKN1C	1	0
CDKN2A	1	0
CDON	1	0
CDX1	1	0
CEACAM16	1	0
CELF5	1	0
CELF6	1	0
CELSR3	1	0
CENPB	1	0
CENPN	1	0
CEP120	1	0
CEP164	1	0
CEP250	1	0
CEP41	1	0
CEP57	1	0
CEP63	1	0
CEP72	1	0
CEP85L,PLN	1	0
CERKL	1	0
CFAP69	1	0
CFH	1	0
CFHR5	1	0
CFL2	1	0
CGA	1	0
CHADL	1	0
CHAT,SLC18A3	1	0
CHCHD10	1	0
CHCHD2	1	0
CHCHD4	1	0
CHD2	1	0
CHD3	1	0
CHD5	1	0
CHD7	1	0
CHML,OPN3	1	0

SetID	P.value	N.Marker.Test
CHMP4B	1	0
CHMP6	1	0
CHN1	1	0
CHPF	1	0
CHRNA1	1	0
CHRNA4	1	0
CHRNB1	1	0
CHRNB2	1	0
CHRNE	1	0
CHST3	1	0
CHST5	1	0
CHST6	1	0
CHUK	1	0
CIB1	1	0
CIB2	1	0
CIBAR2	1	0
CIDEA	1	0
CIDEB,LTB4R2	1	0
CILK1	1	0
CISD2,SLC9B1	1	0
CLCC1,GPSM2	1	0
CLDN16	1	0
CLDN18	1	0
CLDN19	1	0
CLDN22,WWC2	1	0
CLDN4	1	0
CLDN5	1	0
CLHC1	1	0
CLIP4	1	0
CLN3	1	0
CLN8	1	0
CLPB	1	0
CLPP	1	0
CLPX	1	0
CLRN1	1	0
CLSPN	1	0
CNFN	1	0
CNGA1	1	0
CNMD	1	0
CNNM1	1	0
CNNM4	1	0
CNOT4	1	0
CNPY3-GNMT,GNI	1	0
CNTN6	1	0
CNTNAP2	1	0
CNTNAP5	1	0
COA3	1	0
COASY	1	0
COBL	1	0

SetID	P.value	N.Marker.Test
COG2	1	0
COG4	1	0
COG6	1	0
COG7	1	0
COL13A1	1	0
COL4A2	1	0
COLEC11	1	0
COLQ	1	0
COMT	1	0
COPS4	1	0
COPS6	1	0
COQ2	1	0
COQ6	1	0
COQ6,ENTPD5	1	0
COQ8A	1	0
COQ8B	1	0
COQ9	1	0
CORO1A	1	0
CORO7-PAM16,PA	1	0
COX15	1	0
COX20	1	0
CPA6	1	0
CPB1	1	0
CPOX	1	0
CPPED1	1	0
CPT1C	1	0
CPT2	1	2
CRADD	1	0
CRB2	1	0
CRBN	1	0
CREB3L4	1	0
CRHBP	1	0
CRHR2	1	0
CRIP1	1	0
CRIP2	1	0
CRISPLD1	1	0
CRISPLD2	1	0
CRLF1	1	0
CROT	1	0
CRPPA	1	0
CRYAA	1	0
CRYBA4	1	0
CRYBB3	1	0
CRYBG2	1	0
CRYGC	1	0
CRYGD	1	0
CRYGS	1	0
CSF2RB	1	0
CSGALNACT1	1	0
