# Supplementary material for: Whole-genome sequencing analysis of semi-supercentenarians
Source: eLife. 2021 May 4;10:e57849. doi: 10.7554/eLife.57849 (PMC8096429; doi:10.7554/eLife.57849)
Supplement: Supplementary file 12. — Genomic position (hg19), gene name, and group in which the mutation has been identified are reported. [file elife-57849-supp12.pdf]

Table 12S. Disruptive mutations (moderate and high impact). Genomic position (hg19), gene name and group in which the mutation has been identified are reported

Genomic position	Gene	Num. of H/M somatic muations	Group
chr2_25466800	DNMT3A	1	105+
chr4_106156041	TET2	1	105+
chr2_25469922	DNMT3A	1	105+
chr2_198267483	SF3B1	1	105+
chr17_58740806	PPM1D	1	105+
chr2_198266606	SF3B1	1	105+
chr4_106190819	TET2	1	105+
chr2_25469055	DNMT3A	1	105+
chr2_25469947	DNMT3A	1	105+
