# Supplementary material for: Whole-genome sequencing analysis of semi-supercentenarians
Source: eLife. 2021 May 4;10:e57849. doi: 10.7554/eLife.57849 (PMC8096429; doi:10.7554/eLife.57849)
Supplement: Supplementary file 13. [file elife-57849-supp13.pdf]

Table 13S. List of somatic mutations identified that are reported at least seven times in hematopoietic and lymphoid malignancies using the catalogue COSMIC

**chr_posizione**	**GENE_NAME**	**GRCH**	**MUTATION_GENOME_POSITION**	**MUTATION_DESCRIPTION**	**FATHMM_PREDICTION**	**FATHMM_SCORE**	**MUTATION_SOMATIC_STATUS**	
chr2_25466800	DNMT3A	37	2:25466800-25466800	Substitution - Missense	PATHOGENIC	0.97138	Reported in another cancer sample as somatic	105+/110+
chr2_25487027	DNMT3A	37	2:25487027-25487027	Unknown	NEUTRAL	0.01668	Confirmed somatic variant	CTR
chr2_25469055	DNMT3A	37	2:25469055-25469055	Substitution - Missense	PATHOGENIC	0.987	Variant of unknown origin	105+/110+
chr2_25469922	DNMT3A	37	2:25469922-25469922	Substitution - Nonsense	PATHOGENIC	0.99473	Variant of unknown origin	105+/110+
chr20_31014361	ASXL1	37	20:31014361-31014361	Unknown	NEUTRAL	0.0001	Confirmed somatic variant	CTR
chr20_30998850	ASXL1	37	20:30998850-30998859	Unknown			Confirmed somatic variant	CTR
chr20_30965512	ASXL1	37	20:30965512-30965512	Unknown	NEUTRAL	0.00162	Confirmed somatic variant	CTR
chr2_198266606	SF3B1	37	2:198266606-198266606	Substitution - Missense	PATHOGENIC	0.99181	Confirmed somatic variant	105+/110+
chr2_198267483	SF3B1	37	2:198267483-198267483	Substitution - Missense	PATHOGENIC	0.99339	Confirmed somatic variant	105+/110+
chr4_106190819	TET2	37	4:106190819-106190819	Substitution - Missense	PATHOGENIC	0.99153	Reported in another cancer sample as somatic	105+/110+
chr4_106156041	TET2	37	4:106156041-106156041	Substitution - Nonsense		0.65893	Variant of unknown origin	105+/110+
