# Supplementary material for: Whole-genome sequencing analysis of semi-supercentenarians
Source: eLife. 2021 May 4;10:e57849. doi: 10.7554/eLife.57849 (PMC8096429; doi:10.7554/eLife.57849)
Supplement: Supplementary file 14. [file elife-57849-supp14.pdf]

Table 14S. Logistic regression calculated considering genetic risk score for each individual for Alzheimer diseases, cancer (breast, colon, lung, pancreatic, prostate), coronary disease, stroke and type 2 diabetes according to Erikson et al 2016.

**pathology**	**p-val**
alzheimer	0.997
breast	0.997
colon	0.997
coronary	0.997
lung	0.0681
pancreatic	0.997
prostate	0.11
stroke	0.998
type 2 diabetes	0.997
ALL	0.998
