# Supplementary material for: Whole-genome sequencing analysis of semi-supercentenarians
Source: eLife. 2021 May 4;10:e57849. doi: 10.7554/eLife.57849 (PMC8096429; doi:10.7554/eLife.57849)
Supplement: Supplementary file 15. [file elife-57849-supp15.pdf]

Table 15S. CAD genetic risk scores analyzed in Cohort 1

Nr.	CAD GRS Reference	SNP number	SNP effect size measure
1	(Ntalla et al., 2019)	300	Log(OR)
2	(Khera et al., 2018)	6630150	Effect weights
3	(Natarajan et al., 2017)	67	OR
4	(van der Harst & Verweij, 2018)	661	Beta values
5	(Nelson et al., 2017)	72	OR
