# Supplementary material for: Whole-genome sequencing analysis of semi-supercentenarians
Source: eLife. 2021 May 4;10:e57849. doi: 10.7554/eLife.57849 (PMC8096429; doi:10.7554/eLife.57849)
Supplement: Supplementary file 16. [file elife-57849-supp16.pdf]

Table 16S. SNPs used for PRS in Ntalla et al 2019

Reference rsID	Chr	Position (bp)	EA	OA	Proxy rsID	EAF	logOR	SE	P
rs2843152	1	2245570	C	G		0.296	0.042	0.01	0.0000134
rs35465346	1	22132518	G	A		0.838	0.055	0.012	0.00000591
rs7538207	1	22310674	C	T		0.053	0.098	0.022	0.00000573
rs11811081	1	32026584	C	A		0.938	0.077	0.019	0.0000565
rs12733730	1	37023446	A	G		0.195	0.046	0.011	0.0000179
rs11485595	1	38411350	T	C		0.427	0.04	0.009	0.000004
rs34232196	1	55489542	C	T		0.757	0.055	0.01	2.87E-08
rs11591147	1	55505647	G	T		0.984	0.221	0.035	2.84E-10
rs17111652	1	55590465	T	C		0.047	0.082	0.019	0.0000134
rs6665249	1	56890526	A	G		0.258	0.042	0.01	0.0000154
rs56170783	1	57016131	A	C		0.915	0.104	0.015	2.14E-12
rs2149821	1	68719672	A	T		0.283	0.039	0.009	0.0000317
rs10890013	1	73639751	T	C		0.495	0.035	0.008	0.0000226
rs113832197	1	109678062	T	C		0.054	0.081	0.019	0.000034
rs7528419	1	109817192	A	G		0.782	0.109	0.01	3.77E-27
rs11552449	1	114448389	T	C		0.173	0.045	0.011	0.000039
rs10305649	1	150849103	A	C		0.039	0.114	0.024	0.00000287
rs11810571	1	151762308	G	C		0.807	0.058	0.01	2.21E-08
rs6689306	1	154395946	A	G		0.435	0.05	0.008	1.46E-09
rs2789422	1	159892088	G	A		0.599	0.035	0.009	0.0000463
rs6413828	1	175329712	A	T		0.302	0.038	0.009	0.0000177
rs183692864	1	185047852	G	A		0.991	0.244	0.061	0.0000549
rs1892094	1	169094459	C	T		0.516	0.02	0.008	0.0155
rs6700559	1	200646073	C	T		0.53	0.022	0.008	0.00696
rs2820315	1	201872264	T	C		0.296	0.043	0.009	0.00000209
rs67180937	1	222823743	G	T		0.684	0.071	0.01	8.45E-14
rs17464857	1	222762709	T	G		0.856	0.06	0.012	0.00000069
rs3755549	2	1506914	C	T		0.452	0.034	0.008	0.0000264
rs2709437	2	6123492	T	C		0.505	0.034	0.008	0.0000438
rs16986953	2	19942473	A	G		0.073	0.105	0.017	4.77E-10
rs585967	2	21270554	C	A		0.844	0.066	0.012	2.76E-08
rs58560619	2	26563419	C	T		0.488	0.036	0.008	0.0000165
rs4299376	2	44072576	G	T		0.319	0.055	0.009	5.65E-10
rs4076834	2	44081627	T	G		0.936	0.1	0.018	1.23E-08
rs139591697	2	55839855	T	C		0.038	0.102	0.025	0.0000515
rs72375964	2	63130920	A	G	rs6545972	0.653	0.035	0.009	0.0000501
rs7568458	2	85788175	A	T		0.452	0.061	0.008	2.39E-13
rs149366039	2	98543945	T	C		0.001	0.731	0.178	0.0000403
rs7578433	2	100384141	T	C		0.092	0.07	0.017	0.0000387
rs79716828	2	102132484	C	A		0.038	0.111	0.025	0.00000863
rs6761276	2	113832312	T	C		0.432	0.036	0.008	0.0000266
rs7570006	2	136994969	C	T		0.851	0.048	0.012	0.0000378
rs17678683	2	145286559	G	T		0.089	0.077	0.014	0.000000115
rs35500812	2	145830570	A	C	rs12476923	0.32	0.042	0.009	0.0000032
rs12619842	2	164945044	G	C		0.818	0.048	0.011	0.00000857
rs62172372	2	188242369	A	G		0.806	0.046	0.011	0.0000361
rs114123510	2	203831212	A	T		0.116	0.118	0.013	2.88E-19

Reference rsID	Chr	Position (bp)	EA	OA	Proxy rsID	EAF	logOR	SE	P
rs2011559	2	206314671	G	A		0.145	0.054	0.013	0.0000244
rs1250229	2	216304384	T	C		0.262	0.069	0.009	1.85E-13
rs2161967	2	218680529	T	G		0.455	0.039	0.009	0.00000621
rs2972146	2	227100698	T	G		0.648	0.047	0.009	0.000000065
rs10168194	2	228986980	C	G		0.345	0.039	0.009	0.00000678
rs13003675	2	233584109	T	C		0.361	0.042	0.009	0.00000172
rs10929113	2	236172291	C	T		0.221	0.042	0.01	0.0000524
rs143803699	2	236902252	G	C		0.021	0.12	0.029	0.0000295
rs748431	3	14928077	G	T		0.39	0.041	0.008	0.000000904
rs3821396	3	21706369	G	A		0.887	0.06	0.013	0.00000657
rs7623687	3	49448566	A	C		0.859	0.072	0.012	3.72E-09
rs77622129	3	51265685	A	G		0.052	0.094	0.022	0.00002
rs62253653	3	53013267	A	G		0.722	0.038	0.009	0.0000435
rs71331765	3	134223808	G	C		0.154	0.051	0.013	0.0000447
rs6787409	3	135798738	C	T		0.312	0.039	0.009	0.0000163
rs9818870	3	138122122	T	C		0.144	0.068	0.012	7.82E-09
rs4632520	3	152324643	C	T		0.277	0.038	0.009	0.000029
rs12493885	3	153839866	C	G		0.863	0.071	0.013	3.29E-08
rs10513507	3	156983742	C	T		0.343	0.036	0.008	0.000022
rs34229028	3	172117455	G	A	rs12897	0.417	0.037	0.009	0.0000125
rs9869263	3	190030680	G	A		0.841	0.048	0.012	0.0000374
rs113148244	4	31417775	G	T		0.974	0.144	0.034	0.000022
rs16994919	4	38911205	A	G		0.902	0.06	0.014	0.00000955
rs2616407	4	54591399	C	T		0.185	0.049	0.011	0.0000056
rs13134452	4	56028425	C	T		0.631	0.037	0.009	0.0000316
rs72627509	4	57839051	G	C		0.199	0.054	0.01	0.000000081
rs10857147	4	81181072	T	A		0.277	0.054	0.009	8.96E-09
rs138495951	4	100249726	G	A		0.985	0.16	0.039	0.0000384
rs7678555	4	120909501	C	A		0.284	0.048	0.009	0.000000143
rs144059514	4	139146333	G	A	rs6830952	0.017	0.085	0.03	0.0045
rs13109172	4	146759483	C	T	rs4544728	0.644	0.038	0.008	0.00000573
rs4593108	4	148281001	C	G		0.805	0.058	0.01	1.95E-08
rs6841581	4	148401190	A	G		0.153	0.068	0.011	4.57E-10
rs7435973	4	156436507	G	A		0.139	0.059	0.012	0.000000451
rs3796587	4	156638073	C	G		0.816	0.063	0.01	1.24E-09
rs869396	4	169688000	C	A		0.522	0.039	0.008	0.00000185
rs11728590	4	184682414	G	T		0.419	0.037	0.009	0.0000197
rs71600236	5	6721700	C	G		0.691	0.039	0.009	0.00000654
rs112941079	5	9546098	A	G		0.869	0.059	0.013	0.00000386
rs5868014	5	55860907	G	A	rs3936511	0.177	0.052	0.011	0.00000104
rs111777100	5	87402102	A	G		0.034	0.093	0.023	0.0000535
rs288187	5	107344426	C	T		0.825	0.046	0.011	0.0000263
rs1800449	5	121413208	T	C		0.17	0.056	0.011	0.000000406
rs1500187	5	122824678	G	A		0.459	0.037	0.008	0.00000973
rs6883598	5	127926190	C	A		0.268	0.039	0.009	0.0000232
rs273909	5	131667353	G	A		0.116	0.053	0.016	0.000994
rs251023	5	140893410	G	A		0.361	0.038	0.009	0.00000834
rs11955380	5	141910017	C	A		0.102	0.06	0.014	0.0000128
rs3776307	5	142494165	G	A		0.431	0.038	0.009	0.0000094

Reference rsID	Chr	Position (bp)	EA	OA	Proxy rsID	EAF	logOR	SE	P
rs6860540	5	156938327	G	A		0.661	0.035	0.009	0.0000549
rs9501744	6	1617143	C	T		0.872	0.064	0.013	0.00000108
rs421329	6	5090056	C	T		0.775	0.049	0.01	0.00000155
rs742115	6	11327021	C	T		0.484	0.036	0.009	0.0000286
rs6458138	6	12232203	G	A		0.074	0.065	0.016	0.0000613
rs9349379	6	12903957	G	A		0.407	0.105	0.008	9.95E-36
rs13200993	6	22612912	T	C		0.348	0.05	0.009	5.6E-09
rs3130683	6	31888367	T	C		0.86	0.077	0.014	2.77E-08
rs9268402	6	32341353	A	G		0.483	0.014	0.009	0.128
rs4472337	6	34769765	T	C		0.155	0.055	0.012	0.00000242
rs17609940	6	35034800	G	C		0.808	0.029	0.011	0.00711
rs56015508	6	39152041	C	A		0.794	0.054	0.01	0.000000108
rs1214752	6	43396325	C	T		0.503	0.039	0.008	0.00000326
rs6905288	6	43758873	A	G		0.57	0.039	0.008	0.00000326
rs1330633	6	57148971	G	A		0.066	0.067	0.017	0.0000589
rs194937	6	82442022	A	G		0.159	0.048	0.011	0.0000126
rs11153071	6	97039741	G	A		0.819	0.05	0.011	0.000003
rs9398803	6	126683594	A	G		0.51	0.034	0.008	0.0000623
rs12202017	6	134173151	A	G		0.702	0.066	0.009	6.02E-14
rs9493752	6	134204788	A	G		0.013	0.154	0.034	0.00000705
rs2492304	6	134378151	A	T		0.485	0.033	0.008	0.0000559
rs2153219	6	149755695	A	G		0.835	0.051	0.011	0.00000259
rs55730499	6	161005610	T	C		0.067	0.268	0.018	5.64E-49
rs186696265	6	161111700	T	C		0.014	0.466	0.037	8.97E-36
rs4252198	6	161173728	G	C		0.019	0.152	0.035	0.0000166
rs79018195	6	161024291	C	T		0.005	0.304	0.063	0.00000126
rs41269133	6	161087863	T	C		0.911	0.079	0.014	4.78E-08
rs9364552	6	160782934	C	G		0.504	0.035	0.008	0.000015
rs6956990	7	5724091	C	T		0.024	0.115	0.026	0.00000911
rs11509880	7	12261911	A	G		0.365	0.036	0.009	0.0000211
rs2107595	7	19049388	A	G		0.182	0.074	0.01	3.41E-13
rs55889159	7	20289594	A	C	rs6966545	0.364	0.035	0.009	0.0000511
rs78850423	7	28914157	A	G		0.022	0.143	0.032	0.00000949
rs2971672	7	44205906	C	A		0.38	0.036	0.008	0.000019
rs1088868	7	44763122	G	A		0.383	0.04	0.01	0.0000384
rs35146811	7	99720994	C	A		0.276	0.042	0.009	0.00000536
rs112370447	7	107176780	T	C		0.28	0.045	0.009	0.000000962
rs10953541	7	107244545	C	T		0.757	0.027	0.01	0.00577
rs2024233	7	116917427	G	A		0.343	0.037	0.009	0.0000285
rs11556924	7	129663496	C	T		0.657	0.067	0.009	6.26E-13
rs2286198	7	139726207	G	A		0.784	0.051	0.01	0.000000342
rs3918226	7	150690176	T	C		0.071	0.125	0.018	1.58E-12
rs2083636	8	19865263	T	G		0.742	0.051	0.009	6.44E-08
rs28597716	8	19936687	A	G		0.819	0.051	0.011	0.00000685
rs16885577	8	36788479	G	A		0.147	0.049	0.012	0.0000256
rs10109493	8	56711713	A	G		0.888	0.061	0.015	0.0000493
rs72658939	8	65943600	G	C		0.17	0.046	0.011	0.000028
rs77211063	8	105730425	T	C		0.031	0.11	0.026	0.000023
rs10955380	8	106238111	C	A		0.732	0.04	0.009	0.0000119

Reference rsID	Chr	Position (bp)	EA	OA	Proxy rsID	EAF	logOR	SE	P
rs2954029	8	126490972	A	T		0.541	0.06	0.008	5.24E-13
rs117938894	8	132384512	G	A		0.976	0.161	0.039	0.0000373
rs75824083	8	135614908	C	T		0.001	0.907	0.224	0.0000509
rs58594043	8	142230002	A	G	rs3739239	0.87	0.051	0.012	0.0000314
rs34914400	8	145737086	T	C		0.001	0.813	0.188	0.000016
rs2891168	9	22098619	G	A		0.487	0.173	0.008	#######
rs3217992	9	22003223	T	C		0.38	0.122	0.008	5.5E-49
rs1333050	9	22125913	T	C		0.627	0.124	0.009	2.39E-41
rs4149311	9	107588777	T	C		0.146	0.052	0.012	0.00000906
rs1967604	9	110530324	A	G		0.284	0.037	0.009	0.0000501
rs111245230	9	113169775	C	T		0.036	0.109	0.022	0.000000829
rs781622	9	114927849	T	C		0.351	0.036	0.008	0.0000213
rs77275410	9	118806911	C	T		0.089	0.061	0.014	0.0000255
rs10818583	9	124422261	A	G		0.256	0.043	0.009	0.00000485
rs507666	9	136149399	A	G		0.192	0.074	0.01	1.34E-12
rs11257613	10	12284392	G	A		0.494	0.035	0.008	0.0000182
rs7094201	10	29632242	G	A		0.073	0.062	0.015	0.0000577
rs1887318	10	30321598	T	C		0.428	0.058	0.008	4.12E-12
rs1870634	10	44480811	G	T		0.648	0.062	0.009	5.51E-13
rs1657345	10	44779078	A	G		0.865	0.081	0.012	4.58E-12
rs17726488	10	73918058	T	C		0.038	0.103	0.023	0.0000101
rs4691	10	75561916	T	C		0.725	0.041	0.009	0.00000867
rs7098414	10	82214586	A	C		0.264	0.046	0.01	0.00000266
rs2246942	10	91004886	G	A		0.349	0.076	0.009	3.51E-16
rs59898454	10	92658390	A	G		0.978	0.147	0.031	0.00000209
rs11191416	10	104604916	T	G		0.89	0.073	0.012	5.58E-09
rs12252333	10	119166137	G	A		0.271	0.047	0.01	0.0000049
rs2257129	10	122898697	C	T		0.937	0.096	0.02	0.00000164
rs2281674	10	124130308	C	G		0.069	0.064	0.016	0.0000468
rs28596486	10	124274794	C	T		0.148	0.052	0.011	0.00000291
rs56210063	11	8789165	C	G		0.057	0.068	0.017	0.00004
rs10840293	11	9751196	A	G		0.552	0.049	0.008	6.88E-09
rs11042937	11	10745394	T	G		0.511	0.011	0.008	0.202
rs3993105	11	13303071	T	C		0.686	0.047	0.009	0.000000106
rs11462682	11	29210369	G	A	rs1994721	0.842	0.044	0.011	0.0000835
rs146039567	11	38140468	C	A		0.981	0.149	0.035	0.000022
rs2306029	11	46893108	T	C		0.461	0.039	0.009	0.0000157
rs2727020	11	49111407	C	G		0.686	0.042	0.009	0.0000047
rs12146487	11	64026639	G	A		0.831	0.048	0.011	0.0000228
rs12801636	11	65391317	G	A		0.766	0.043	0.01	0.00000775
rs571353	11	75152243	C	T		0.281	0.043	0.009	0.00000347
rs634552	11	75282052	G	T		0.859	0.05	0.012	0.0000471
rs3133293	11	77195100	G	T		0.676	0.041	0.009	0.00000334
rs17712139	11	100567565	G	A		0.233	0.041	0.01	0.0000362
rs2212437	11	102798691	A	G		0.289	0.04	0.009	0.00000763
rs2839812	11	103673294	T	A		0.307	0.06	0.009	1.99E-11
rs567040	11	111460678	C	T		0.325	0.037	0.009	0.0000621
rs964184	11	116648917	G	C		0.163	0.051	0.011	0.00000468
rs3782774	12	3371231	G	A		0.472	0.036	0.009	0.0000217

Reference rsID	Chr	Position (bp)	EA	OA	Proxy rsID	EAF	logOR	SE	P
rs3861086	12	20218869	C	T		0.691	0.045	0.009	0.000000634
rs11170820	12	54513915	G	C		0.07	0.089	0.017	0.000000238
rs56245751	12	56795415	T	C		0.825	0.061	0.014	0.0000113
rs11172113	12	57527283	C	T		0.408	0.036	0.008	0.0000244
rs2229357	12	57843711	G	A		0.764	0.047	0.01	0.00000339
rs6538176	12	76649823	T	C		0.197	0.047	0.01	0.00000624
rs11115214	12	82574691	C	T		0.223	0.042	0.01	0.0000371
rs2681472	12	90008959	G	A		0.189	0.066	0.01	7.63E-11
rs10774625	12	111910219	A	G		0.491	0.064	0.009	9.22E-14
rs2244608	12	121416988	G	A		0.335	0.051	0.009	2.32E-09
rs11057401	12	124427306	T	A		0.687	0.044	0.009	0.00000132
rs11057830	12	125307053	A	G		0.147	0.069	0.012	4.24E-09
rs1924981	13	29022645	T	C		0.334	0.046	0.009	0.000000186
rs9591012	13	33058333	G	A		0.663	0.038	0.009	0.0000187
rs73468973	13	42613890	A	G		0.195	0.043	0.011	0.0000568
rs75535189	13	61711000	C	T		0.008	0.262	0.059	0.0000101
rs9515203	13	111049623	T	C		0.752	0.062	0.01	6.48E-10
rs4773141	13	110954353	G	C		0.36	0.059	0.01	9.46E-10
rs9588107	13	110823340	A	G		0.522	0.033	0.008	0.000078
rs12867664	13	111066208	A	G		0.047	0.086	0.02	0.0000157
rs17102313	14	24709976	T	C		0.002	0.615	0.151	0.0000464
rs12891473	14	35452257	C	T		0.556	0.035	0.008	0.0000233
rs4506804	14	42296685	T	G		0.499	0.034	0.008	0.0000314
rs3832966	14	75614504	C	T	rs10138183	0.478	0.037	0.008	0.00000467
rs112635299	14	94838142	G	T		0.982	0.163	0.038	0.0000165
rs10139550	14	100145710	G	C		0.421	0.051	0.008	1.84E-09
rs113025579	14	103131425	C	T		0.972	0.125	0.031	0.0000494
rs147580454	15	55902144	C	T	rs491014	0.461	0.033	0.008	0.0000972
rs6494488	15	65024204	A	G		0.799	0.034	0.011	0.0018
rs72743461	15	67441750	C	A		0.783	0.071	0.01	4.81E-12
rs7164479	15	79123054	T	C		0.578	0.072	0.008	6.38E-18
rs2083460	15	89574484	T	C		0.885	0.072	0.014	0.000000141
rs2071382	15	91428197	T	C		0.464	0.062	0.009	7.14E-13
rs17581137	15	96146414	A	C		0.755	0.042	0.01	0.0000138
rs116082507	16	1837788	T	C		0.001	1.204	0.261	0.00000413
rs7185993	16	15820297	T	C		0.414	0.036	0.008	0.0000123
rs247616	16	56989590	C	T		0.678	0.044	0.009	0.00000101
rs35259348	16	72003952	C	G		0.242	0.051	0.01	0.000000115
rs1050362	16	72130815	A	C		0.368	0.029	0.008	0.000484
rs9929108	16	75243138	T	G		0.69	0.047	0.009	0.000000232
rs3851738	16	75387533	C	G		0.573	0.041	0.008	0.000000667
rs7500448	16	83045790	A	G		0.763	0.059	0.01	5.14E-09
rs1968266	16	84739212	T	C		0.371	0.037	0.009	0.0000523
rs117592425	17	2024564	A	C		0.987	0.203	0.044	0.00000414
rs113348108	17	2088848	G	A	rs57130712	0.325	0.044	0.009	0.000000202
rs8068571	17	2525175	T	C		0.748	0.042	0.01	0.0000176
rs9897596	17	17593453	T	C		0.523	0.039	0.008	0.00000313
rs13723	17	27941886	G	A		0.488	0.035	0.008	0.0000239
rs148720362	17	30018492	C	T	rs62062229	0.256	0.035	0.01	0.000506

Reference rsID	Chr	Position (bp)	EA	OA	Proxy rsID	EAF	logOR	SE	P
rs1122326	17	40274873	C	A		0.232	0.05	0.011	0.00000276
rs8068844	17	40571284	C	T		0.344	0.043	0.009	0.000000393
rs17608766	17	45013271	C	T		0.13	0.044	0.013	0.000607
rs46522	17	46988597	T	C		0.527	0.033	0.008	0.0000931
rs4643373	17	47123423	T	C		0.724	0.046	0.009	0.0000012
rs62076439	17	47404628	T	G		0.339	0.044	0.009	0.000000783
rs8068952	17	59286644	G	C		0.228	0.07	0.011	1.41E-09
rs7212798	17	59013488	C	T	rs8080784	0.152	0.063	0.012	4.37E-08
rs6504218	17	62408299	G	A		0.524	0.041	0.008	0.000000941
rs11077501	17	68526061	C	T		0.353	0.037	0.009	0.0000219
rs75589791	17	71639797	G	A		0.069	0.065	0.016	0.0000458
rs35489971	17	72700943	A	G		0.194	0.054	0.011	0.00000106
rs11654510	17	74677362	C	A		0.851	0.058	0.013	0.00000946
rs7211674	17	76899065	C	A		0.43	0.034	0.008	0.0000614
rs9951447	18	20009691	C	T		0.406	0.038	0.008	0.00000437
rs178002	18	20078962	G	A		0.369	0.042	0.009	0.00000191
rs12922	18	32722917	A	C		0.879	0.05	0.012	0.0000292
rs833509	18	46528787	C	T		0.721	0.039	0.009	0.0000258
rs948937	18	47179901	A	T		0.357	0.034	0.009	0.0000624
rs35614134	18	57832856	A	C	rs571312	0.249	0.039	0.009	0.000023
rs663129	18	57838401	A	G		0.248	0.04	0.009	0.0000182
rs116843064	19	8429323	G	A		0.98	0.159	0.031	0.000000287
rs111397563	19	11171865	T	C	rs68010235	0.714	0.052	0.009	1.28E-08
rs6511720	19	11202306	G	T		0.884	0.128	0.013	7.88E-22
rs2738448	19	11228726	G	C		0.432	0.034	0.008	0.0000504
rs167479	19	11526765	G	T		0.526	0.04	0.008	0.00000226
rs73015715	19	17855840	T	C		0.202	0.049	0.01	0.00000232
rs78030362	19	18575193	G	A		0.071	0.069	0.017	0.0000546
rs10423964	19	31764851	T	C		0.28	0.039	0.009	0.0000238
rs10417115	19	33386556	C	T		0.061	0.068	0.016	0.0000225
rs34322801	19	41824179	C	G		0.836	0.05	0.011	0.00000603
rs73045269	19	41825191	T	C		0.152	0.064	0.012	0.000000171
rs4760	19	44153100	G	A		0.155	0.054	0.013	0.0000256
rs7412	19	45412079	C	T		0.922	0.143	0.016	2.17E-19
rs56131196	19	45422846	A	G		0.174	0.082	0.012	2.71E-12
rs1964272	19	46190268	G	A		0.518	0.044	0.009	0.000000229
rs425105	19	47208481	C	T		0.162	0.047	0.011	0.0000472
rs13734	20	17594729	A	G		0.204	0.043	0.01	0.0000203
rs59909520	20	33591107	C	T	rs8121710	0.9	0.059	0.013	0.0000104
rs867186	20	33764554	A	G		0.9	0.057	0.013	0.0000147
rs117113213	20	39165692	A	G		0.034	0.131	0.027	0.00000119
rs6129767	20	39822332	G	T		0.296	0.04	0.009	0.0000104
rs56313611	20	47456856	C	T		0.866	0.058	0.012	0.00000139
rs259983	20	57735457	C	A		0.144	0.056	0.012	0.00000289
rs3813452	20	61174357	T	C		0.386	0.035	0.008	0.0000367
rs2832275	21	30602994	T	A		0.176	0.051	0.011	0.00000204
rs75187018	21	31259893	G	A		0.974	0.136	0.032	0.0000185
rs28451064	21	35593827	A	G		0.124	0.133	0.013	2.62E-23
rs743339	21	35655217	C	T		0.243	0.075	0.01	3.05E-14

Reference rsID	Chr	Position (bp)	EA	OA	Proxy rsID	EAF	logOR	SE	P
rs117696200	21	37284291	T	G		0.933	0.079	0.018	0.0000182
rs2836621	21	40054149	T	C		0.478	0.033	0.008	0.0000468
rs35219138	21	45117913	C	A	rs914198	0.608	0.034	0.008	0.0000483
rs9604969	22	19514013	A	G		0.918	0.06	0.015	0.0000569
rs71313931	22	19960184	G	C		0.284	0.039	0.009	0.0000187
rs11287675	22	24256692	C	T	rs5751771	0.426	0.035	0.008	0.0000278
rs12485143	22	24575333	C	T		0.936	0.067	0.016	0.0000272
rs468224	22	29955252	A	G		0.238	0.043	0.01	0.00000885
