# Supplementary material for: Whole-genome sequencing analysis of semi-supercentenarians
Source: eLife. 2021 May 4;10:e57849. doi: 10.7554/eLife.57849 (PMC8096429; doi:10.7554/eLife.57849)
Supplement: Supplementary file 17. [file elife-57849-supp17.pdf]

Table 17S. SNPs used for PRS in Natarajan et al 2018

Locus	Gene	Lead SNP	CHR:POS (GRCh38p12)	CHR:POS (GRCh37/hg19)	Lead Risk Allele	CHD OR
1p13.3	SORT1	rs602633	chr1:109278889	1_109821511	C	1.12
1p32.2	PPAP2B	rs17114036	chr1:56497149	1_56962821	A	1.11
1p32.3	PCSK9	rs11206510	chr1:55030366	1_55496039	T	1.06
1q21.3	IL6R	rs4845625	chr1:154449591	1_154422067	T	1.06
1q41	MIA3	rs17464857	chr1:222589367	1_222762709	T	1.05
21q22.11	KCNE2	rs9982601	chr21:34226827	21_35599128	T	1.13
22q11.23	POM121L9P, ADORA2A	rs180803	chr22:24262890	22_24658858	G	1.02
2p11.2	GGCX/VAMP8	rs1561198	chr2:85582866	2_85809989	A	1.06
2p21	ABCG8	rs6544713	chr2:43846742	2_44073881	T	1.06
2p24.1	APOB	rs515135	chr2:21063185	2_21286057	G	1.07
2q22.3	ZEB2-AC074093.1	rs2252641	chr2:145043894	2_145801461	G	1.06
2q33.1	WDR12	rs6725887	chr2:202881162	2_203745885	C	1.12
2q37.1	GIGYF2, KCNJ13	rs1801251	chr2:232768750	2_233633460	A	1.05
3q22.3	MRAS	rs9818870	chr3:138403280	3_138122122	T	1.07
4q12	REST,NOA1	rs17087335	chr4:56972417	4_57838583	T	1.06
4q31.22	EDNRA	rs1878406	chr4:147472512	4_148393664	T	1.1
4q32.1	GUCY1A3	rs7692387	chr4:155714157	4_156635309	G	1.08
5q31.1	SLC22A4/SLC22A5	rs273909	chr5:132331660	5_131667353	C	1.07
6p21.2	KCNK5	rs10947789	chr6:39207146	6_39174922	T	1.07
6p21.31	ANKS1A	rs12205331	chr6:34930678	6_34898455	C	1.04
6p21.33	C2	rs3130683	chr6:31920590	6_31888367	T	1.09
6p24.1	PHACTR1	rs9369640	chr6:12901209	6_12901441	A	1.09
6q23.2	TCF21	rs12190287	chr6:133893387	6_134214525	C	1.07
6q25.3	LPA	rs3798220	chr6:160540105	6_160961137	C	1.28
6q25.3	SLC22A3/LPAL2/LPA	rs2048327	chr6:160442500	6_160863532	G	1.06
6q26	PLG	rs4252120	chr6:160722576	6_161143608	T	1.07
7p21.1	HDAC9	rs2023938	chr7:18997152	7_19036775	G	1.08
7q22.3	NA	rs12539895	chr7:107451404	7_107091849	A	1.08
7q32.2	ZC3HC1	rs11556924	chr7:130023656	7_129663496	C	1.09
7q36.1	NOS3	rs3918226	chr7:150993088	7_150690176	T	1.14
8p21.3	LPL	rs264	chr8:19955669	8_19813180	G	1.11
8q24.13	TRIB1	rs2954029	chr8:125478730	8_126490972	A	1.06
9p21.3	CDKN2A	rs1333049	chr9:22125504	9_22125503	C	1.21
9p21.3	CDKN2A	rs3217992	chr9:22003224	9_22003223	A	1.16
9q31.3	SVEP1	rs111245230	chr9:110407495	9_113169775	C	1.14
9q34.2	ABO	rs579459	chr9:133278724	9_136154168	C	1.07
10p11.23	KIAA1462	rs2505083	chr10:30046193	10_30335122	C	1.06
10q11.21	CXCL12	rs501120	chr10:44258419	10_44753867	A	1.07
10q11.21	CXCL12	rs2047009	chr10:44044465	10_44539913	C	1.05
10q23.31	LIPA	rs2246833	chr10:89246097	10_91005854	T	1.06
10q23.31	LIPA	rs11203042	chr10:89229352	10_90989109	T	1.04
10q24.32	CYP17A1	rs12413409	chr10:102959339	10_104719096	G	1.1
11p15.3	NA	rs11042937	chr11:10723847	11_10745394	T	1.04
11p15.4	SWAP70	rs10840293	chr11:9729649	11_9751196	A	1.06
11q22.3	PDGFD	rs974819	chr11:103789839	11_103660567	A	1.07
11q23.3	APOA5,APOA1	rs9326246	chr11:116741017	11_116611733	C	1.09
12p13.3	LRP1	rs11172113	chr12:57133500	12_57527283	C	1.06
12p24.31	SCARB1	rs11057830	chr12:124822507	12_125307053	A	1.08
12q24.12	SH2B3	rs3184504	chr12:111446804	12_111884608	T	1.07
13q12.3	FLT1	rs9319428	chr13:28399484	13_28973621	A	1.06
13q34	COL4A1	rs4773144	chr13:110308365	13_110960712	T	1.08
13q34	COL4A1/COL4A2	rs9515203	chr13:110397276	13_111049623	G	1.07
14q32.2	HHIPL1	rs2895811	chr14:99667605	14_100133942	C	1.06
15q22.33	SMAD3	rs17293632	chr15:67150258	15_67442596	C	1.05
15q25.1	ADAMTS7	rs7173743	chr15:78849442	15_79141784	T	1.07
15q26.1	MFGE8,ABHD2	rs8042271	chr15:89030987	15_89574218	G	1.1
15q26.1	FURIN/FES	rs17514846	chr15:90873320	15_91416550	A	1.07
16q13	CETP	rs247616	chr16:56955678	16_56989590	C	1.05

Locus	Gene	Lead SNP	CHR:POS (GRCh38p12)	CHR:POS (GRCh37/hg19)	Lead Risk Allele	CHD OR
17p11.2	RASD1	rs12936587	chr17:17640408	17_17543722	G	1.06
17p13.3	SMG6	rs2281727	chr17:2214651	17_2117945	C	1.04
17q21.32	UBE2Z	rs15563	chr17:48927831	17_47005193	C	1.04
17q23.2	BCAS3	rs8080784	chr17:60939664	17_59017025	C	1.06
18q21.32	PMAIP1,MC4R	rs663129	chr18:60171168	18_57838401	A	1.06
19p13.2	ANGPTL4	rs116843064	chr19:8364439	19_8429323	G	1.16
19p13.2	LDLR	rs1122608	chr19:11052925	19_11163601	G	1.1
19q13.32	APOE,APOC1	rs445925	chr19:44912383	19_45415640	C	1.13
19q13.32	APOE,APOC1	rs2075650	chr19:44892362	19_45395619	G	1.11
