# Supplementary material for: Whole-genome sequencing analysis of semi-supercentenarians
Source: eLife. 2021 May 4;10:e57849. doi: 10.7554/eLife.57849 (PMC8096429; doi:10.7554/eLife.57849)
Supplement: Supplementary file 18. [file elife-57849-supp18.pdf]

Table 18S. SNPs used for PRS in van der Hars et al 2018

REGION	SNPS	CHR_ID	CHR:POS (GRCh38p12)	to lift over	CHR:POS (GRCh37/hg19)	Allele	OR or BETA
12q23.3	rs1077854	12	107358300	chr12:107358300-107358300	12_107752077	A	0.029
1p31.1	rs7271182	1	82542105	chr1:82542105-82542105	1_83007788	A	0.133
16p13.3	rs2235642	16	1534865	chr16:1534865-1534865	16_1584866	T	0.027
19p13.3	rs5630735	19	1946186	chr19:1946186-1946186	19_1946185	T	0.03
2p16.1	rs1271342	2	60399170	chr2:60399170-60399170	2_60626305	A	0.031
22q11.21	rs5993586	22	19304503	chr22:19304503-19304503	22_19292026	T	0.064
1q42.12	rs6426551	1	226354348	chr1:226354348-226354348	1_226542049	A	0.033
14q13.3	rs6072429	14	36977398	chr14:36977398-36977398	14_37446603	A	0.059
5q23.3	rs4836390	5	128675995	chr5:128675995-128675995	5_128011688	T	0.04
1p21.3	rs4394694	1	97905893	chr1:97905893-97905893	1_98371449	A	0.034
1p13.2	rs1214430	1	113772871	chr1:113772871-113772871	1_114315493	T	0.037
3q27.2	rs1309287	3	185777532	chr3:185777532-185777532	3_185495320	A	0.031
6q25.3	rs294883	6	159261174	chr6:159261174-159261174	6_159682206	T	0.032
8q12.1	rs1120015	8	55158830	chr8:55158830-55158830	8_56071390	T	0.075
6q15	rs6905628	6	89635150	chr6:89635150-89635150	6_90344869	C	0.032
2q22.1	rs1303505	2	136240494	chr2:136240494-136240494	2_136998064	T	0.033
17p13.1	rs6741489	17	9710287	chr17:9710287-9710287	17_9613604	C	0.048
2p15	rs360798	2	62723300	chr2:62723300-62723300	2_62950435	A	0.038
11q23.3	rs1089256	11	120353941	chr11:120353941-120353941	11_120224650	A	0.03
15q26.2	rs2860711	15	94204658	chr15:94204658-94204658	15_94747887	T	0.086
10q21.3	rs2671540	10	63972738	chr10:63972738-63972738	10_65732498	T	0.031
6q11.1	rs1449615	6	61506512	chr6:61506512-61506512	6_62216417	A	0.062
17q12	rs7282339	17	39267817	chr17:39267817-39267817	17_37424070	T	0.121
19q13.43	rs7305993	19	56097934	chr19:56097934-56097934	19_56609303	A	0.042
1p35.3	rs1137163	1	27602129	chr1:27602129-27602129	1_27928640	A	0.064
10q24.31	rs603424	10	100315722	chr10:100315722-100315722	10_102075479	A	0.033
7p21.2	rs2073533	7	13990114	chr7:13990114-13990114	7_14029739	T	0.032
2q13	rs6736093	2	111897665	chr2:111897665-111897665	2_112655242	A	0.033
8q21.11	rs7842194	8	73912151	chr8:73912151-73912151	8_74824386	A	0.036
3p13	rs7310409	3	70061238	chr3:70061238-70061238	3_70110389	T	0.07
20q13.33	rs7314358	20	63814349	chr20:63814349-63814349	20_62445702	A	0.056
11q13.3	rs7294513	11	70267795	chr11:70267795-70267795	11_70113901	C	0.047
2p25.2	rs7957631	2	5927875	chr2:5927875-5927875	2_6068007	A	0.109
14q32.33	rs4906332	14	103513797	chr14:103513797-103513797	14_103980134	A	0.032
10q23.33	rs4919044	10	93012138	chr10:93012138-93012138	10_94771895	T	0.053
10q23.33	rs1223583	10	93996743	chr10:93996743-93996743	10_95756500	A	0.026
13q13.3	rs9547692	13	36899106	chr13:36899106-36899106	13_37473243	A	0.036
8p21.2	rs3606901	8	25200975	chr8:25200975-25200975	8_25058491	T	0.035
6q24.2	rs4896604	6	142906275	chr6:142906275-142906275	6_143227412	A	0.031
5q35.3	rs337366	5	177503650	chr5:177503650-177503650	5_176930651	A	0.035
20q13.13	rs587648	20	49900976	chr20:49900976-49900976	20_48517513	T	0.045
10p11.21	rs4934855	10	37400135	chr10:37400135-37400135	10_37689063	A	0.121
4q28.1	rs1310267	4	124978810	chr4:124978810-124978810	4_125899965	T	0.032
2p14	rs6213908	2	65280779	chr2:65280779-65280779	2_65507913	A	0.037
1q42.13	rs1180944	1	228138791	chr1:228138791-228138791	1_228326492	T	0.038
11q23.3	rs1172041	11	118328710	chr11:118328710-118328710	11_118199425	A	0.111
11q23.3	rs1185460	11	119072723	chr11:119072723-119072723	11_118943433	A	0.032
16q24.1	rs6140307	16	86666118	chr16:86666118-86666118	16_86699724	T	0.034
15q24.1	rs2852267	15	73931375	chr15:73931375-73931375	15_74223716	C	0.039
18q11.2	rs1623003	18	23585199	chr18:23585199-23585199	18_21165163	T	0.034
3q22.2	rs1308864	3	134438421	chr3:134438421-134438421	3_134157263	A	0.033
16p13.11	rs216158	16	15823981	chr16:15823981-15823981	16_15917838	C	0.033
11q24.2	rs3509918	11	126384371	chr11:126384371-126384371	11_126254266	T	0.038
5q21.3	rs2454899	5	106929424	chr5:106929424-106929424	5_106265125	A	0.034
18q11.2	rs4800401	18	22423662	chr18:22423662-22423662	18_20003625	T	0.033
7p14.2	rs1215480	7	35217909	chr7:35217909-35217909	7_35257521	A	0.037
1q21.2	rs6780799	1	150023307	chr1:150023307-150023307	1_149995265	A	0.034
5q11.2	rs35006	5	54146895	chr5:54146895-54146895	5_53442725	C	0.035
9p23	rs1096120	9	13724052	chr9:13724052-13724052	9_13724051	A	0.057
9p22.3	rs1012114	9	14292721	chr9:14292721-14292721	9_14292720	A	0.031
5q12.3	rs1005563	5	67009610	chr5:67009610-67009610	5_66305438	A	0.035
5q22.1	rs256277	5	112071113	chr5:112071113-112071113	5_111406810	T	0.035

REGION	SNPS	CHR_ID	CHR:POS (GRCh38p12)	to lift over	CHR:POS (GRCh37/hg19)	Allele	OR or BETA
21q22.3	rs6078734	21	43590437	chr21:43590437-43590437	21_45010318	A	0.032
15q15.1	rs1116849	15	41144539	chr15:41144539-41144539	15_41436737	A	0.034
2p23.3	rs1169046	2	26367659	chr2:26367659-26367659	2_26590527	A	0.036
12q12	rs1291621	12	45947038	chr12:45947038-45947038	12_46340821	A	0.128
2q22.3	rs1301073	2	143442474	chr2:143442474-143442474	2_144200043	A	0.035
22q12.2	rs877549	22	30276431	chr22:30276431-30276431	22_30672420	A	0.047
4q31.21	rs4345206	4	145845384	chr4:145845384-145845384	4_146766536	T	0.034
11q22.3	rs4753788	11	107221129	chr11:107221129-107221129	11_107091855	A	0.034
5q23.2	rs2546343	5	123098971	chr5:123098971-123098971	5_122434666	T	0.034
12q13.3	rs1231543	12	57387153	chr12:57387153-57387153	12_57780936	A	0.04
7p13	rs2107732	7	45038379	chr7:45038379-45038379	7_45077978	A	0.061
5q23.1	rs5607929	5	121941102	chr5:121941102-121941102	5_121276797	A	0.042
4p16.3	rs5995028	4	3450618	chr4:3450618-3450618	4_3452345	A	0.033
7q22.3	rs1739857	7	106769006	chr7:106769006-106769006	7_106409452	A	0.033
7q22.3	rs7809950	7	107597362	chr7:107597362-107597362	7_107237807	T	0.039
22q13.1	rs6001960	22	40547028	chr22:40547028-40547028	22_40943032	A	0.04
11p15.2	rs3415642	11	16460403	chr11:16460403-16460403	11_16481950	A	0.048
11p15.1	rs5767783	11	17006642	chr11:17006642-17006642	11_17028189	A	0.033
5q11.2	rs3936510	5	56565039	chr5:56565039-56565039	5_55860866	T	0.039
6p25.3	rs6239061	6	1616030	chr6:1616030-1616030	6_1616265	A	0.055
1q42.13	rs6541297	1	230147759	chr1:230147759-230147759	1_230283506	A	0.035
6p21.2	rs1478020	6	36672808	chr6:36672808-36672808	6_36640585	A	0.057
1q32.2	rs1161616	1	210300277	chr1:210300277-210300277	1_210473622	A	0.05
17q11.2	rs7695479	17	31706495	chr17:31706495-31706495	17_30033514	T	0.043
7p21.3	rs6460942	7	12381363	chr7:12381363-12381363	7_12420989	T	0.047
4q32.3	rs7696431	4	168766574	chr4:168766574-168766574	4_169687725	T	0.035
2q37.3	rs4496284	2	237311587	chr2:237311587-237311587	2_238220230	A	0.033
8q23.1	rs2841665	8	105552424	chr8:105552424-105552424	8_106564652	A	0.036
7p22.1	rs1095198	7	6406396	chr7:6406396-6406396	7_6446027	A	0.038
7p21.3	rs1008898	7	7233928	chr7:7233928-7233928	7_7273559	T	0.037
1p36.33	rs1692580	1	2226509	chr1:2226509-2226509	1_2157948	T	0.034
1p36.32	rs3609619	1	2320766	chr1:2320766-2320766	1_2252205	T	0.053
3q25.31	rs4266144	3	157134803	chr3:157134803-157134803	3_156852592	C	0.038
11p11.2	rs2679053	11	43583754	chr11:43583754-43583754	11_43605304	A	0.034
2p21	rs582384	2	45669298	chr2:45669298-45669298	2_45896437	A	0.037
4q22.3	rs1434538	4	95145521	chr4:95145521-95145521	4_96066672	T	0.034
7p22.3	rs1176063	7	1813117	chr7:1813117-1813117	7_1852753	T	0.04
2q32.1	rs840611	2	187333719	chr2:187333719-187333719	2_188198446	A	0.032
8p22	rs4646248	8	18402845	chr8:18402845-18402845	8_18260355	T	0.032
21q21.3	rs7319380	21	29162981	chr21:29162981-29162981	21_30535302	A	0.049
17q23.2	rs8079951	17	61165403	chr17:61165403-61165403	17_59242764	A	0.048
6q25.1	rs6557122	6	151239600	chr6:151239600-151239600	6_151560735	T	0.036
18q21.1	rs2337157	18	49000440	chr18:49000440-49000440	18_46526810	A	0.036
18q21.1	rs9964304	18	49703347	chr18:49703347-49703347	18_47229717	A	0.043
17q21.32	rs8069437	17	46829583	chr17:46829583-46829583	17_44906949	T	0.039
17q21.32	rs1912483	17	47366128	chr17:47366128-47366128	17_45443494	A	0.029
20q13.32	rs236710	20	59118574	chr20:59118574-59118574	20_57693629	T	0.047
16q13	rs5622860	16	56953853	chr16:56953853-56953853	16_56987765	T	0.039
10q24.33	rs4918072	10	103933886	chr10:103933886-103933886	10_105693644	A	0.042
1p36.32	rs1090986	1	3020251	chr1:3020251-3020251	1_2936815	A	0.043
1p36.32	rs2493298	1	3409348	chr1:3409348-3409348	1_3325912	A	0.057
14q32.13	rs1126352	14	94371805	chr14:94371805-94371805	14_94838142	T	0.165
12p13.31	rs1183826	12	7068568	chr12:7068568-7068568	12_7175872	T	0.058
14q24.3	rs1013189	14	74980176	chr14:74980176-74980176	14_75446879	C	0.038
9q33.2	rs4131193	9	120977282	chr9:120977282-120977282	9_123739560	T	0.057
6q14.1	rs1080623	6	81993618	chr6:81993618-81993618	6_82703335	T	0.037
11q13.4	rs566818	11	75447305	chr11:75447305-75447305	11_75158350	A	0.032
11q13.5	rs659418	11	75573289	chr11:75573289-75573289	11_75284334	T	0.053
17q21.2	rs3489148	17	42111440	chr17:42111440-42111440	17_40263458	T	0.074
17q21.31	rs9912587	17	43021069	chr17:43021069-43021069	17_41173086	A	0.043
1p13.2	rs1213671	1	115212576	chr1:115212576-115212576	1_115755197	A	0.032
17q11.2	rs7220401	17	29608670	chr17:29608670-29608670	17_27935688	T	0.04
3q26.31	rs4894803	3	172082466	chr3:172082466-172082466	3_171800256	A	0.031
3p25.1	rs1077534	3	14836067	chr3:14836067-14836067	3_14877574	A	0.059

REGION	SNPS	CHR_ID	CHR:POS (GRCh38p12)	to lift over	CHR:POS (GRCh37/hg19)	Allele	OR or BETA
3p25.1	rs1488807	3	14923130	chr3:14923130-14923130	3_14964637	A	0.503
1p34.3	rs1905697	1	37407990	chr1:37407990-37407990	1_37873591	A	0.165
1p34.3	rs6177671	1	37995647	chr1:37995647-37995647	1_38461319	A	0.041
7q34	rs7795996	7	139995345	chr7:139995345-139995345	7_139695144	A	0.027
4q21.1	rs1250082	4	76495474	chr4:76495474-76495474	4_77416627	A	0.036
4q21.22	rs1109949	4	81665896	chr4:81665896-81665896	4_82587050	A	0.043
6q22.32	rs1591805	6	126395918	chr6:126395918-126395918	6_126717064	A	0.041
13q13.1	rs1184050	13	32624572	chr13:32624572-32624572	13_33198709	A	0.04
11q22.1	rs604723	11	100739815	chr11:100739815-100739815	11_100610546	T	0.04
10q26.13	rs4237540	10	122477982	chr10:122477982-122477982	10_124237498	A	0.04
11q13.1	rs3741380	11	65581592	chr11:65581592-65581592	11_65349063	A	0.041
16q22.2	rs8053891	16	71963890	chr16:71963890-71963890	16_71997789	A	0.03
2q24.3	rs1299990	2	164100741	chr2:164100741-164100741	2_164957251	A	0.056
1q21.3	rs4845570	1	151786628	chr1:151786628-151786628	1_151759104	T	0.058
13q12.3	rs9513112	13	28423565	chr13:28423565-28423565	13_28997702	A	0.04
13q12.3	rs1177096	13	28753140	chr13:28753140-28753140	13_29327277	T	0.113
3p21.31	rs1249522	3	48137688	chr3:48137688-48137688	3_48179178	T	0.035
3p21.31	rs7308236	3	49092945	chr3:49092945-49092945	3_49130378	T	0.061
19p13.2	rs1168430	19	8364439	chr19:8364439-8364439	19_8429323	A	0.161
19p13.2	rs5937229	19	8481254	chr19:8481254-8481254	19_8546138	T	0.039
6p21.31	rs9469890	6	34796205	chr6:34796205-34796205	6_34763982	T	0.054
6p21.31	rs1132444	6	35560913	chr6:35560913-35560913	6_35528690	A	0.058
10q23.1	rs1768074	10	80491758	chr10:80491758-80491758	10_82251514	T	0.047
20q11.22	rs1169899	20	34861936	chr20:34861936-34861936	20_33449739	C	0.034
20q11.22	rs6060235	20	35103849	chr20:35103849-35103849	20_33691652	A	0.055
8p21.3	rs7322584	8	22172552	chr8:22172552-22172552	8_22030065	T	0.096
13q34	rs1201954	13	113005392	chr13:113005392-113005392	13_113659706	A	0.038
6p21.2	rs1045610	6	39215694	chr6:39215694-39215694	6_39183470	T	0.044
6p22.3	rs7766436	6	22598030	chr6:22598030-22598030	6_22598259	T	0.048
6p21.1	rs1034246	6	43100632	chr6:43100632-43100632	6_43068370	T	0.04
6p21.1	rs6905288	6	43791136	chr6:43791136-43791136	6_43758873	A	0.045
9q31.2	rs2140480	9	107763828	chr9:107763828-107763828	9_110526109	T	0.047
2q35	rs6174126	2	217804502	chr2:217804502-217804502	2_218669225	T	0.062
2q35	rs1129411	2	217869242	chr2:217869242-217869242	2_218733965	A	0.108
11p15.4	rs1073464	11	9759344	chr11:9759344-9759344	11_9780891	T	0.044
2q22.3	rs6740731	2	144513025	chr2:144513025-144513025	2_145270592	A	0.051
2q22.3	rs7604735	2	145082088	chr2:145082088-145082088	2_145839655	T	0.049
2q22.3	rs7286233	2	145825059	chr2:145825059-145825059	2_146582627	A	0.046
6p21.33	rs685031	6	31913954	chr6:31913954-31913954	6_31881731	A	0.043
12q22	rs4762479	12	95101265	chr12:95101265-95101265	12_95495041	T	0.08
1q24.2	rs1275381	1	169183350	chr1:169183350-169183350	1_169152588	T	0.036
6p11.2	rs223290	6	57241914	chr6:57241914-57241914	6_57106712	T	0.089
16q23.3	rs7342694	16	81870195	chr16:81870195-81870195	16_81903800	T	0.037
11p15.4	rs1160150	11	5679844	chr11:5679844-5679844	11_5701074	A	0.084
5p15.31	rs1726391	5	9552226	chr5:9552226-9552226	5_9552338	A	0.066
3q21.2	rs4678145	3	124731234	chr3:124731234-124731234	3_124450081	C	0.068
12q13.13	rs1117082	12	54120131	chr12:54120131-54120131	12_54513915	C	0.097
19p13.11	rs1120094	19	17736035	chr19:17736035-17736035	19_17846844	A	0.06
19p13.11	rs1132300	19	18350146	chr19:18350146-18350146	19_18460956	A	0.033
19p13.11	rs1167005	19	18479133	chr19:18479133-18479133	19_18589943	T	0.088
12p12.2	rs1084144	12	20067099	chr12:20067099-20067099	12_20220033	C	0.051
8p21.3	rs7011846	8	19928145	chr8:19928145-19928145	8_19785656	A	0.113
8p21.3	rs15285	8	19967156	chr8:19967156-19967156	8_19824667	T	0.052
8p21.3	rs1010665	8	20070649	chr8:20070649-20070649	8_19928160	A	0.048
3q25.2	rs701145	3	154337010	chr3:154337010-154337010	3_154054799	T	0.072
4q21.21	rs1085714	4	80259918	chr4:80259918-80259918	4_81181072	A	0.055
11q23.3	rs964184	11	116778201	chr11:116778201-116778201	11_116648917	C	0.071
10q24.32	rs1241477	10	102938024	chr10:102938024-102938024	10_104697781	T	0.055
3q22.3	rs655720	3	136384425	chr3:136384425-136384425	3_136103267	T	0.049
4q27	rs7678555	4	119988346	chr4:119988346-119988346	4_120909501	A	0.056
12q21.33	rs5748106	12	89625401	chr12:89625401-89625401	12_90019178	C	0.061
2p24.1	rs1698695	2	19742712	chr2:19742712-19742712	2_19942473	A	0.086
16q23.1	rs1293045	16	75428157	chr16:75428157-75428157	16_75462055	A	0.052
1q21.3	rs1213364	1	154455807	chr1:154455807-154455807	1_154428283	A	0.043

REGION	SNPS	CHR_ID	CHR:POS (GRCh38p12)	to lift over	CHR:POS (GRCh37/hg19)	Allele	OR or BETA
14q32.2	rs8003602	14	99682624	chr14:99682624-99682624	14_100148961	T	0.061
14q32.2	rs1013151	14	99892957	chr14:99892957-99892957	14_100359294	T	0.043
17q21.32	rs7214663	17	48960409	chr17:48960409-48960409	17_47037771	A	0.053
17q21.32	rs1918965	17	49148374	chr17:49148374-49148374	17_47225736	T	0.162
17q21.33	rs7502499	17	49412740	chr17:49412740-49412740	17_47490102	A	0.043
16q23.3	rs7500448	16	83012185	chr16:83012185-83012185	16_83045790	A	0.063
17p13.3	rs6206803	17	2129018	chr17:2129018-2129018	17_2032312	A	0.118
17p13.3	rs7209460	17	2145419	chr17:2145419-2145419	17_2048713	T	0.053
19q13.2	rs7304526	19	41319286	chr19:41319286-41319286	19_41825191	T	0.07
19q13.2	rs4803455	19	41345604	chr19:41345604-41345604	19_41851509	A	0.054
15q22.33	rs1722805	15	67157967	chr15:67157967-67157967	15_67450305	A	0.066
5q31.3	rs246600	5	143137332	chr5:143137332-143137332	5_142516897	T	0.048
2p24.1	rs1022174	2	21196778	chr2:21196778-21196778	2_21419650	A	0.054
10p11.23	rs9337951	10	30028144	chr10:30028144-30028144	10_30317073	A	0.062
3q22.3	rs3732837	3	138403078	chr3:138403078-138403078	3_138121920	A	0.063
12q24.31	rs1169288	12	120978847	chr12:120978847-120978847	12_121416650	A	0.054
2p21	rs6740322	2	43334790	chr2:43334790-43334790	2_43561929	A	0.04
2p21	rs4299376	2	43845437	chr2:43845437-43845437	2_44072576	T	0.057
2q35	rs1250259	2	215435759	chr2:215435759-215435759	2_216300482	A	0.048
9q34.2	rs495828	9	133279294	chr9:133279294-133279294	9_136154867	T	0.06
12q24.31	rs1105784	12	124831509	chr12:124831509-124831509	12_125316055	A	0.067
12q24.31	rs1012895	12	124855901	chr12:124855901-124855901	12_125340447	A	0.039
7q36.1	rs3918226	7	150993088	chr7:150993088-150993088	7_150690176	T	0.122
4q32.1	rs2880099	4	155511216	chr4:155511216-155511216	4_156432368	A	0.06
4q32.1	rs1313193	4	155693032	chr4:155693032-155693032	4_156614184	T	0.065
2p11.2	rs2166529	2	85515052	chr2:85515052-85515052	2_85742175	T	0.063
4q31.22	rs6810798	4	147369666	chr4:147369666-147369666	4_148290818	A	0.06
4q31.22	rs1761269	4	147444187	chr4:147444187-147444187	4_148365339	A	0.079
13q34	rs1161795	13	110165755	chr13:110165755-110165755	13_110818102	A	0.081
13q34	rs4773141	13	110302006	chr13:110302006-110302006	13_110954353	C	0.06
13q34	rs9515203	13	110397276	chr13:110397276-110397276	13_111049623	T	0.068
7p21.1	rs2107595	7	19009765	chr7:19009765-19009765	7_19049388	A	0.082
10q23.31	rs1412444	10	89243170	chr10:89243170-89243170	10_91002927	T	0.064
10q11.21	rs1079351	10	44001523	chr10:44001523-44001523	10_44496971	T	0.067
10q11.21	rs2457480	10	44244562	chr10:44244562-44244562	10_44740010	A	0.106
7q32.2	rs1155692	7	130023656	chr7:130023656-130023656	7_129663496	T	0.062
1p32.3	rs1159114	1	55039974	chr1:55039974-55039974	1_55505647	T	0.282
8q24.13	rs2860176	8	125487789	chr8:125487789-125487789	8_126500031	C	0.06
15q26.1	rs4932373	15	90886057	chr15:90886057-90886057	15_91429287	A	0.073
1p32.2	rs7267553	1	56111674	chr1:56111674-56111674	1_56577346	A	0.031
1p32.2	rs1711404	1	56500678	chr1:56500678-56500678	1_56966350	A	0.107
1p32.2	rs1470556	1	56520631	chr1:56520631-56520631	1_56986303	A	0.088
11q22.3	rs2128739	11	103802549	chr11:103802549-103802549	11_103673277	A	0.074
12q24.12	rs4766578	12	111466567	chr12:111466567-111466567	12_111904371	A	0.059
15q25.1	rs1163778	15	78846658	chr15:78846658-78846658	15_79139000	T	0.074
1q41	rs1746598	1	222664597	chr1:222664597-222664597	1_222837939	A	0.079
6q23.2	rs6919211	6	133678730	chr6:133678730-133678730	6_133999868	C	0.042
6q23.2	rs1966248	6	133838484	chr6:133838484-133838484	6_134159622	A	0.076
6q23.2	rs965652	6	134047815	chr6:134047815-134047815	6_134368953	A	0.034
2q33.2	rs7293453	2	203104250	chr2:203104250-203104250	2_203968973	T	0.128
21q22.11	rs2845106	21	34221526	chr21:34221526-34221526	21_35593827	A	0.119
21q22.11	rs1494871	21	34233562	chr21:34233562-34233562	21_35605863	T	0.101
19q13.32	rs7412	19	44908822	chr19:44908822-44908822	19_45412079	T	0.16
19q13.32	rs157595	19	44922203	chr19:44922203-44922203	19_45425460	A	0.032
19q13.32	rs1167309	19	45238836	chr19:45238836-45238836	19_45742094	A	0.047
19p13.2	rs6511720	19	11091630	chr19:11091630-11091630	19_11202306	T	0.136
19p13.2	rs2738447	19	11116804	chr19:11116804-11116804	19_11227480	A	0.036
19p13.2	rs318719	19	11386305	chr19:11386305-11386305	19_11496981	T	0.054
1p13.3	rs1274037	1	109274968	chr1:109274968-109274968	1_109817590	T	0.124
6p24.1	rs1629862	6	12295643	chr6:12295643-12295643	6_12295876	A	0.054
6p24.1	rs9349379	6	12903725	chr6:12903725-12903725	6_12903957	A	0.121
6q25.3	rs1221441	6	160489485	chr6:160489485-160489485	6_160910517	A	0.069
6q25.3	rs1475555	6	160490564	chr6:160490564-160490564	6_160911596	A	0.398
6q25.3	rs5573049	6	160584578	chr6:160584578-160584578	6_161005610	T	0.337

REGION	SNPS	CHR_ID	CHR:POS (GRCh38p12)	to lift over	CHR:POS (GRCh37/hg19)	Allele	OR or BETA
6q25.3	rs1405708	6	160591981	chr6:160591981-160591981	6_161013013	T	0.481
6q25.3	rs7359681	6	160596331	chr6:160596331-160596331	6_161017363	A	0.118
6q26	rs1450990	6	160871806	chr6:160871806-160871806	6_161292838	A	0.204
9p21.3	rs1455424	9	21953100	chr9:21953100-21953100	9_21953099	A	0.158
9p21.3	rs2891168	9	22098620	chr9:22098620-22098620	9_22098619	A	0.189
1p36.32	rs2843152	1	2314131	chr1:2314131-2314131	1_2245570	C	0.0406
1p36.32	rs2493298	1	3409348	chr1:3409348-3409348	1_3325912	A	0.0567
1p36.22	rs1703739	1	11800786	chr1:11800786-11800786	1_11860843	A	0.0395
1p36.12	rs3546534	1	21806025	chr1:21806025-21806025	1_22132518	A	0.0401
1p34.3	rs4072980	1	37990434	chr1:37990434-37990434	1_38456106	A	0.0353
1p32.3	rs1159114	1	55039974	chr1:55039974-55039974	1_55505647	T	0.2821
1p32.2	rs4634932	1	56530519	chr1:56530519-56530519	1_56996191	T	0.1286
1p32.2	rs1741628	1	56554977	chr1:56554977-56554977	1_57020650	C	0.0669
1p22.2	rs950037	1	88685915	chr1:88685915-88685915	1_89151598	T	0.0274
1p13.3	rs602633	1	109278889	chr1:109278889-109278889	1_109821511	T	0.112
1p13.2	rs1180631	1	115210861	chr1:115210861-115210861	1_115753482	A	0.0369
1q21.2	rs1120466	1	150570652	chr1:150570652-150570652	1_150543128	T	0.0389
1q21.3	rs1181057	1	151789832	chr1:151789832-151789832	1_151762308	C	0.0553
1q21.3	rs4845625	1	154449591	chr1:154449591-154449591	1_154422067	T	0.0448
1q24.2	rs1892094	1	169125221	chr1:169125221-169125221	1_169094459	T	0.0413
1q32.1	rs6700559	1	200676945	chr1:200676945-200676945	1_200646073	T	0.0325
1q32.1	rs2820315	1	201903136	chr1:201903136-201903136	1_201872264	T	0.0405
1q32.3	rs1157153	1	212613683	chr1:212613683-212613683	1_212787025	T	0.0631
1q41	rs1746563	1	222650187	chr1:222650187-222650187	1_222823529	A	0.0817
1q42.13	rs2281719	1	230161913	chr1:230161913-230161913	1_230297659	T	0.0289
1q42.2	rs699	1	230710048	chr1:230710048-230710048	1_230845794	A	0.036
2p24.1	rs1698695	2	19742712	chr2:19742712-19742712	2_19942473	A	0.0855
2p24.1	rs668948	2	21068657	chr2:21068657-21068657	2_21291529	A	0.063
2p23.3	rs1550115	2	24818751	chr2:24818751-24818751	2_25041620	T	0.0321
2p21	rs2052923	2	43184681	chr2:43184681-43184681	2_43411820	T	0.0316
2p21	rs1049590	2	43771587	chr2:43771587-43771587	2_43998726	A	0.041
2p21	rs6544713	2	43846742	chr2:43846742-43846742	2_44073881	T	0.0549
2p13.3	rs898123	2	71152220	chr2:71152220-71152220	2_71379350	C	0.0287
2p11.2	rs1561198	2	85582866	chr2:85582866-85582866	2_85809989	T	0.0549
2q14.1	rs1018613	2	113079367	chr2:113079367-113079367	2_113836944	T	0.0281
2q14.3	rs2923	2	127706044	chr2:127706044-127706044	2_128463618	C	0.0743
2q22.3	rs7849041	2	143405256	chr2:143405256-143405256	2_144162825	T	0.0449
2q22.3	rs1767868	2	144528992	chr2:144528992-144528992	2_145286559	T	0.056
2q22.3	rs2252641	2	145043894	chr2:145043894-145043894	2_145801461	T	0.0401
2q24.2	rs3399898	2	162427557	chr2:162427557-162427557	2_163284067	T	0.0374
2q24.3	rs1261984	2	164088534	chr2:164088534-164088534	2_164945044	C	0.0549
2q32.1	rs840616	2	187331742	chr2:187331742-187331742	2_188196469	T	0.0402
2q33.2	rs1156546	2	203029276	chr2:203029276-203029276	2_203893999	A	0.1179
2q35	rs1250229	2	215439661	chr2:215439661-215439661	2_216304384	T	0.0644
2q35	rs2571445	2	217818431	chr2:217818431-217818431	2_218683154	A	0.041
2q36.3	rs2943634	2	226203364	chr2:226203364-226203364	2_227068080	A	0.0409
2q36.3	rs4566357	2	227057299	chr2:227057299-227057299	2_227922015	A	0.026
2q37.1	rs1801251	2	232768750	chr2:232768750-232768750	2_233633460	A	0.0416
2q37.1	rs1405130	2	234655048	chr2:234655048-234655048	2_235563692	T	0.0325
3p25.1	rs1307922	3	14860018	chr3:14860018-14860018	3_14901525	C	0.0405
3p21.33	rs1171845	3	44015406	chr3:44015406-44015406	3_44056898	T	0.0296
3p21.31	rs7617773	3	48152025	chr3:48152025-48152025	3_48193515	T	0.0393
3p21.31	rs7623687	3	49411133	chr3:49411133-49411133	3_49448566	A	0.0529
3p14.1	rs4411878	3	64717989	chr3:64717989-64717989	3_64703665	T	0.0287
3q13.31	rs9840892	3	115175249	chr3:115175249-115175249	3_114894096	A	0.1086
3q21.2	rs1784379	3	124734175	chr3:124734175-124734175	3_124453022	T	0.0679
3q22.1	rs1051286	3	132539117	chr3:132539117-132539117	3_132257961	T	0.0454
3q22.3	rs7322223	3	136169800	chr3:136169800-136169800	3_135888642	A	0.0385
3q22.3	rs185244	3	138374047	chr3:138374047-138374047	3_138092889	T	0.0737
3q25.2	rs1249388	3	154122077	chr3:154122077-154122077	3_153839866	C	0.074
3q26.31	rs12897	3	172398112	chr3:172398112-172398112	3_172115902	A	0.042
4p16.3	rs1684440	4	3447925	chr4:3447925-3447925	4_3449652	A	0.0683
4q12	rs2616407	4	53725232	chr4:53725232-53725232	4_54591399	T	0.0381
4q12	rs5726525	4	56973114	chr4:56973114-56973114	4_57839280	A	0.0468

REGION	SNPS	CHR_ID	CHR:POS (GRCh38p12)	to lift over	CHR:POS (GRCh37/hg19)	Allele	OR or BETA
4q21.1	rs1250082	4	76495474	chr4:76495474-76495474	4_77416627	A	0.0359
4q21.21	rs1085714	4	80259918	chr4:80259918-80259918	4_81181072	A	0.0553
4q21.22	rs1109949	4	81665896	chr4:81665896-81665896	4_82587050	A	0.0431
4q22.3	rs2452600	4	94575731	chr4:94575731-94575731	4_95496882	T	0.0365
4q24	rs1703527	4	105115388	chr4:105115388-105115388	4_106036545	T	0.036
4q27	rs1172343	4	119980181	chr4:119980181-119980181	4_120901336	A	0.0519
4q31.21	rs3587980	4	145861685	chr4:145861685-145861685	4_146782837	A	0.0333
4q31.22	rs4593108	4	147359849	chr4:147359849-147359849	4_148281001	C	0.0627
4q31.22	rs6842241	4	147479667	chr4:147479667-147479667	4_148400819	A	0.081
4q32.1	rs1311882	4	155515365	chr4:155515365-155515365	4_156436517	A	0.042
4q32.1	rs7692387	4	155714157	chr4:155714157-155714157	4_156635309	A	0.0687
4q32.1	rs1451867	4	159118343	chr4:159118343-159118343	4_160039495	A	0.1419
4q32.3	rs7696431	4	168766574	chr4:168766574-168766574	4_169687725	T	0.0351
5p15.31	rs4389656	5	6753615	chr5:6753615-6753615	5_6753728	A	0.0328
5p15.31	rs1508798	5	9556582	chr5:9556582-9556582	5_9556694	T	0.0516
5q11.2	rs459193	5	56510924	chr5:56510924-56510924	5_55806751	A	0.0399
5q11.2	rs832540	5	56903375	chr5:56903375-56903375	5_56199202	A	0.0322
5q12.3	rs9283706	5	66987692	chr5:66987692-66987692	5_66283520	C	0.0345
5q13.3	rs1005681	5	75309395	chr5:75309395-75309395	5_74605220	A	0.0274
5q23.1	rs1130752	5	122026055	chr5:122026055-122026055	5_121361750	A	0.0448
5q23.2	rs2081914	5	123337928	chr5:123337928-123337928	5_122673622	C	0.0346
5q31.1	rs273909	5	132331660	chr5:132331660-132331660	5_131667353	A	0.0534
5q31.3	rs246600	5	143137332	chr5:143137332-143137332	5_142516897	T	0.048
6p25.3	rs9501744	6	1616908	chr6:1616908-1616908	6_1617143	T	0.0553
6p24.2	rs742115	6	11326788	chr6:11326788-11326788	6_11327021	T	0.0331
6p24.1	rs9349379	6	12903725	chr6:12903725-12903725	6_12903957	A	0.1205
6p22.3	rs2876643	6	22591296	chr6:22591296-22591296	6_22591525	A	0.0458
6p22.2	rs9379774	6	25596815	chr6:25596815-25596815	6_25597043	A	0.0384
6p21.33	rs2072633	6	31951801	chr6:31951801-31951801	6_31919578	A	0.0411
6p21.31	rs2814993	6	34651116	chr6:34651116-34651116	6_34618893	A	0.061
6p21.2	rs6930083	6	36666379	chr6:36666379-36666379	6_36634156	A	0.029
6p21.2	rs1094778	6	39207146	chr6:39207146-39207146	6_39174922	T	0.0462
6p21.2	rs1094778	6	39207146	chr6:39207146-39207146	6_39174922	T	0.0413
6p21.1	rs1214752	6	43428587	chr6:43428587-43428587	6_43396325	T	0.0305
6p21.1	rs6905288	6	43791136	chr6:43791136-43791136	6_43758873	A	0.045
6p12.3	rs4644046	6	51520211	chr6:51520211-51520211	6_51385009	A	0.0275
6p11.2	rs9367716	6	57295774	chr6:57295774-57295774	6_57160572	T	0.0375
6q14.1	rs4613862	6	81902554	chr6:81902554-81902554	6_82612271	A	0.0345
6q16.1	rs9486719	6	96612248	chr6:96612248-96612248	6_97060124	A	0.0413
6q22.32	rs1591805	6	126395918	chr6:126395918-126395918	6_126717064	A	0.0412
6q23.2	rs9493740	6	133839756	chr6:133839756-133839756	6_134160894	T	0.1197
6q23.2	rs2327429	6	133888699	chr6:133888699-133888699	6_134209837	T	0.0723
6q23.2	rs2811686	6	134048286	chr6:134048286-134048286	6_134369424	T	0.0312
6q25.1	rs2153219	6	149434559	chr6:149434559-149434559	6_149755695	A	0.0429
6q25.1	rs1266349	6	150682704	chr6:150682704-150682704	6_151003840	T	0.0591
6q25.3	rs688359	6	160044259	chr6:160044259-160044259	6_160465291	A	0.0526
6q25.3	rs1475555	6	160490564	chr6:160490564-160490564	6_160911596	A	0.3977
6q25.3	rs5573049	6	160584578	chr6:160584578-160584578	6_161005610	T	0.3368
6q25.3	rs4127211	6	160585045	chr6:160585045-160585045	6_161006077	T	0.1153
6q26	rs4126913	6	160666831	chr6:160666831-160666831	6_161087863	T	0.0637
6q26	rs1866962	6	160690668	chr6:160690668-160690668	6_161111700	T	0.5467
7p22.3	rs1026759	7	1897625	chr7:1897625-1897625	7_1937261	A	0.0412
7p21.3	rs1150988	7	12222285	chr7:12222285-12222285	7_12261911	A	0.0367
7p21.1	rs2107595	7	19009765	chr7:19009765-19009765	7_19049388	A	0.0815
7p21.1	rs4719608	7	20252511	chr7:20252511-20252511	7_20292134	A	0.0329
7q22.1	rs3514681	7	100123371	chr7:100123371-100123371	7_99720994	A	0.0337
7q22.3	rs6817081	7	107619276	chr7:107619276-107619276	7_107259721	T	0.0343
7q31.2	rs1024965	7	117424571	chr7:117424571-117424571	7_117064625	T	0.0311
7q32.2	rs1155692	7	130023656	chr7:130023656-130023656	7_129663496	T	0.0618
7q32.2	rs1155692	7	130023656	chr7:130023656-130023656	7_129663496	T	0.0575
7q32.2	rs7783857	7	130754299	chr7:130754299-130754299	7_130439058	C	0.0297
7q34	rs1023737	7	140057336	chr7:140057336-140057336	7_139757136	T	0.0423
7q36.1	rs3918226	7	150993088	chr7:150993088-150993088	7_150690176	T	0.1219
8p22	rs6997340	8	18429487	chr8:18429487-18429487	8_18286997	T	0.036

REGION	SNPS	CHR_ID	CHR:POS (GRCh38p12)	to lift over	CHR:POS (GRCh37/hg19)	Allele	OR or BETA
8p21.3	rs6997330	8	19943018	chr8:19943018-19943018	8_19800529	C	0.1291
8p21.3	rs264	8	19955669	chr8:19955669-19955669	8_19813180	A	0.0632
8p21.3	rs1120408	8	20083285	chr8:20083285-20083285	8_19940796	T	0.0432
8p21.3	rs6984210	8	22176102	chr8:22176102-22176102	8_22033615	C	0.0846
8p21.2	rs1137563	8	26245256	chr8:26245256-26245256	8_26102772	T	0.0398
8q22.1	rs1267525	8	94267412	chr8:94267412-94267412	8_95279640	T	0.0438
8q23.1	rs2342781	8	105253492	chr8:105253492-105253492	8_106265720	C	0.0297
8q24.13	rs2954029	8	125478730	chr8:125478730-125478730	8_126490972	A	0.0666
8q24.13	rs2954029	8	125478730	chr8:125478730-125478730	8_126490972	A	0.0546
9p22.3	rs475957	9	15229624	chr9:15229624-15229624	9_15229622	T	0.0437
9p21.3	rs896655	9	21706572	chr9:21706572-21706572	9_21706571	A	0.04
9p21.3	rs3217978	9	22007331	chr9:22007331-22007331	9_22007330	A	0.1546
9p21.3	rs1333049	9	22125504	chr9:22125504-22125504	9_22125503	C	0.1879
9p21.2	rs1322052	9	27179246	chr9:27179246-27179246	9_27179244	T	0.0337
9q22.33	rs4743150	9	97977842	chr9:97977842-97977842	9_100740124	T	0.0355
9q31.1	rs4149309	9	104827299	chr9:104827299-104827299	9_107589580	A	0.0418
9q31.1	rs1800978	9	104903697	chr9:104903697-104903697	9_107665978	C	0.0438
9q31.2	rs944172	9	107755513	chr9:107755513-107755513	9_110517794	T	0.0422
9q31.3	rs1112452	9	110407495	chr9:110407495-110407495	9_113169775	T	0.098
9q33.2	rs1081857	9	121650669	chr9:121650669-121650669	9_124412948	T	0.0429
	rs507666	9	133273983	chr9:133273983-133273983	9_136149399	A	0.0734
10p12.1	rs1888657	10	24544685	chr10:24544685-24544685	10_24833614	T	0.0349
10p11.23	rs2487928	10	30034963	chr10:30034963-30034963	10_30323892	A	0.0546
10p11.22	rs1555321	10	33200961	chr10:33200961-33200961	10_33489889	A	0.0297
10q11.21	rs1254531	10	44083614	chr10:44083614-44083614	10_44579062	T	0.0399
10q11.21	rs1657346	10	44282112	chr10:44282112-44282112	10_44777560	C	0.0918
10q21.1	rs2879627	10	51923587	chr10:51923587-51923587	10_53683347	A	0.0348
10q22.1	rs4403732	10	71878397	chr10:71878397-71878397	10_73638155	C	0.0346
10q23.1	rs1226425	10	80517585	chr10:80517585-80517585	10_82277341	A	0.0435
10q23.31	rs2246942	10	89245129	chr10:89245129-89245129	10_91004886	A	0.0706
10q24.32	rs3740390	10	102878723	chr10:102878723-102878723	10_104638480	T	0.0719
10q24.33	rs1276587	10	103909864	chr10:103909864-103909864	10_105669622	T	0.0327
10q25.2	rs7901695	10	112994329	chr10:112994329-112994329	10_114754088	T	0.0304
10q26.12	rs2257129	10	121139183	chr10:121139183-121139183	10_122898697	T	0.0835
10q26.13	rs2268344	10	122485453	chr10:122485453-122485453	10_124244969	T	0.0333
11p15.4	rs7762870	11	5662842	chr11:5662842-5662842	11_5684072	T	0.0682
11p15.4	rs1160150	11	5679844	chr11:5679844-5679844	11_5701074	A	0.0838
11p15.4	rs7941600	11	9258737	chr11:9258737-9258737	11_9280284	A	0.0517
11p15.4	rs1084029	11	9729649	chr11:9729649-9729649	11_9751196	A	0.046
11p15.4	rs1104293	11	10723847	chr11:10723847-10723847	11_10745394	T	0.0403
11p15.3	rs1102273	11	13271635	chr11:13271635-13271635	11_13293182	T	0.038
11p15.2	rs1102407	11	16895672	chr11:16895672-16895672	11_16917219	T	0.0328
11p14.1	rs1691723	11	27680836	chr11:27680836-27680836	11_27702383	T	0.0319
11p11.12	rs2727020	11	49089855	chr11:49089855-49089855	11_49111407	C	0.031
11q13.1	rs1280163	11	65623846	chr11:65623846-65623846	11_65391317	A	0.0441
11q13.1	rs1280163	11	65623846	chr11:65623846-65623846	11_65391317	A	0.0418
11q13.5	rs590121	11	75563105	chr11:75563105-75563105	11_75274150	T	0.0386
11q22.1	rs7947761	11	100753868	chr11:100753868-100753868	11_100624599	A	0.042
11q22.2	rs1278892	11	102931118	chr11:102931118-102931118	11_102801847	A	0.0324
11q22.3	rs974819	11	103789839	chr11:103789839-103789839	11_103660567	T	0.0674
11q22.3	rs3758911	11	107326914	chr11:107326914-107326914	11_107197640	T	0.0356
11q22.3	rs7356201	11	110401537	chr11:110401537-110401537	11_110272261	A	0.0458
11q23.3	rs964184	11	116778201	chr11:116778201-116778201	11_116648917	C	0.0705
11q24.2	rs7933887	11	126405869	chr11:126405869-126405869	11_126275764	A	0.0344
12p13.32	rs3782774	12	3262065	chr12:3262065-3262065	12_3371231	A	0.0317
12p12.2	rs1084144	12	20067099	chr12:20067099-20067099	12_20220033	C	0.051
12p11.21	rs1161042	12	31259735	chr12:31259735-31259735	12_31412669	A	0.0361
12q12	rs1282694	12	42402498	chr12:42402498-42402498	12_42796300	A	0.0347
12q13.13	rs1117082	12	54120131	chr12:54120131-54120131	12_54513915	C	0.0969
12q13.3	rs1117211	12	57133500	chr12:57133500-57133500	12_57527283	T	0.0349
12q13.3	rs1117211	12	57133500	chr12:57133500-57133500	12_57527283	T	0.0257
12q21.33	rs2681492	12	89619312	chr12:89619312-89619312	12_90013089	T	0.0629
12q22	rs7306455	12	94961765	chr12:94961765-94961765	12_95355541	A	0.0549
12q24.12	rs3184504	12	111446804	chr12:111446804-111446804	12_111884608	T	0.0742

REGION	SNPS	CHR_ID	CHR:POS (GRCh38p12)	to lift over	CHR:POS (GRCh37/hg19)	Allele	OR or BETA
12q24.31	rs2244608	12	120979185	chr12:120979185-120979185	12_121416988	A	0.0518
12q24.31	rs1105740	12	123942759	chr12:123942759-123942759	12_124427306	A	0.0383
12q24.31	rs1105783	12	124822507	chr12:124822507-124822507	12_125307053	A	0.0821
13q12.3	rs9319428	13	28399484	chr13:28399484-28399484	13_28973621	A	0.0404
13q13.1	rs9591012	13	32484196	chr13:32484196-32484196	13_33058333	A	0.0441
13q34	rs1286657	13	110182619	chr13:110182619-110182619	13_110834966	T	0.0828
13q34	rs9521678	13	110263771	chr13:110263771-110263771	13_110916118	T	0.0408
13q34	rs9515203	13	110397276	chr13:110397276-110397276	13_111049623	T	0.0675
13q34	rs9583531	13	110732814	chr13:110732814-110732814	13_111385161	T	0.0448
13q34	rs1317507	13	112977466	chr13:112977466-112977466	13_113631780	A	0.0434
14q24.3	rs7145159	14	75116565	chr14:75116565-75116565	14_75583268	T	0.0348
14q32.2	rs2895811	14	99667605	chr14:99667605-99667605	14_100133942	T	0.0442
15q15.1	rs1244004	15	41490486	chr15:41490486-41490486	15_41782684	A	0.0345
15q22.31	rs6494488	15	64732005	chr15:64732005-64732005	15_65024204	A	0.0431
15q22.33	rs5606213	15	67163292	chr15:67163292-67163292	15_67455630	T	0.0655
15q24.1	rs2507	15	73983335	chr15:73983335-73983335	15_74275676	A	0.0303
15q25.1	rs6495335	15	78824791	chr15:78824791-78824791	15_79117133	T	0.0694
15q25.3	rs2880765	15	85513231	chr15:85513231-85513231	15_86056462	A	0.0284
15q26.1	rs8042271	15	89030987	chr15:89030987-89030987	15_89574218	A	0.0746
15q26.1	rs1751484	15	90873320	chr15:90873320-90873320	15_91416550	A	0.0613
15q26.2	rs1758113	15	95603185	chr15:95603185-95603185	15_96146414	A	0.0419
16p13.11	rs9972711	16	15808733	chr16:15808733-15808733	16_15902590	A	0.0351
16q13	rs1800775	16	56961324	chr16:56961324-56961324	16_56995236	A	0.0405
16q22.2	rs1050362	16	72096916	chr16:72096916-72096916	16_72130815	A	0.0386
16q23.1	rs4888378	16	75298143	chr16:75298143-75298143	16_75332041	A	0.0439
16q23.3	rs7199941	16	81872818	chr16:81872818-81872818	16_81906423	A	0.0401
16q23.3	rs7500448	16	83012185	chr16:83012185-83012185	16_83045790	A	0.0633
17p11.2	rs1293658	17	17640408	chr17:17640408-17640408	17_17543722	A	0.0361
17q11.2	rs13723	17	29614868	chr17:29614868-29614868	17_27941886	A	0.0395
17q21.2	rs2074158	17	42105145	chr17:42105145-42105145	17_40257163	T	0.0543
17q21.32	rs1760876	17	46935905	chr17:46935905-46935905	17_45013271	T	0.0519
17q21.32	rs3895874	17	48970506	chr17:48970506-48970506	17_47047868	A	0.0414
17q21.33	rs1694804	17	49363104	chr17:49363104-49363104	17_47440466	A	0.0534
17q23.2	rs8080784	17	60939664	chr17:60939664-60939664	17_59017025	T	0.053
17q23.2	rs2286526	17	61394696	chr17:61394696-61394696	17_59472057	T	0.0282
17q23.3	rs1867624	17	64309731	chr17:64309731-64309731	17_62387091	T	0.0369
17q24.2	rs1801689	17	66214462	chr17:66214462-66214462	17_64210580	A	0.0813
17q24.2	rs4148005	17	68886325	chr17:68886325-68886325	17_66882466	T	0.0286
17q25.1	rs3548997	17	74704804	chr17:74704804-74704804	17_72700943	A	0.0438
17q25.1	rs1135889	17	75930040	chr17:75930040-75930040	17_73926121	A	0.0403
18q11.2	rs3813127	18	22457634	chr18:22457634-22457634	18_20037597	A	0.0337
18q21.1	rs833509	18	49002417	chr18:49002417-49002417	18_46528787	T	0.0399
18q21.1	rs2000813	18	49567494	chr18:49567494-49567494	18_47093864	T	0.033
18q21.32	rs3463341	18	60187461	chr18:60187461-60187461	18_57854694	T	0.0423
19p13.2	rs1168430	19	8364439	chr19:8364439-8364439	19_8429323	A	0.1609
19p13.2	rs6511720	19	11091630	chr19:11091630-11091630	19_11202306	T	0.1359
19p13.2	rs1761666	19	11192878	chr19:11192878-11192878	19_11303554	A	0.0567
19p13.11	rs3745348	19	17101600	chr19:17101600-17101600	19_17212410	T	0.0325
19p13.11	rs7301571	19	17744954	chr19:17744954-17744954	19_17855763	C	0.0593
19q13.2	rs1298094	19	41326326	chr19:41326326-41326326	19_41832231	A	0.0609
19q13.2	rs2288874	19	41346870	chr19:41346870-41346870	19_41852775	T	0.0531
19q13.32	rs7412	19	44908822	chr19:44908822-44908822	19_45412079	T	0.1603
19q13.32	rs8103278	19	45867123	chr19:45867123-45867123	19_46370381	A	0.0331
19q13.32	rs425105	19	46705224	chr19:46705224-46705224	19_47208481	T	0.0385
20p12.1	rs13734	20	17614084	chr20:17614084-17614084	20_17594729	A	0.0384
20q11.22	rs6088590	20	34725762	chr20:34725762-34725762	20_33313566	T	0.0338
20q11.22	rs867186	20	35176751	chr20:35176751-35176751	20_33764554	A	0.063
20q12	rs6129767	20	41193692	chr20:41193692-41193692	20_39822332	T	0.0385
20q13.12	rs3827066	20	45957384	chr20:45957384-45957384	20_44586023	T	0.0467
20q13.13	rs5631361	20	48840319	chr20:48840319-48840319	20_47456856	T	0.0524
21q21.3	rs2832227	21	29160755	chr21:29160755-29160755	21_30533076	A	0.0438
21q22.11	rs2845106	21	34221526	chr21:34221526-34221526	21_35593827	A	0.1193
21q22.2	rs2836633	21	38695521	chr21:38695521-38695521	21_40067445	A	0.0285
21q22.3	rs2838344	21	43669996	chr21:43669996-43669996	21_45089877	A	0.0299

REGION	SNPS	CHR_ID	CHR:POS (GRCh38p12)	to lift over	CHR:POS (GRCh37/hg19)	Allele	OR or BETA
22q11.23	rs8141797	22	24186073	chr22:24186073-24186073	22_24582041	A	0.0655
22q11.23	rs5760293	22	24266203	chr22:24266203-24266203	22_24662171	T	0.1275
22q12.2	rs6006426	22	30273894	chr22:30273894-30273894	22_30669883	A	0.03
1p36.32	rs3609619	1	2320766	chr1:2320766-2320766	1_2252205	T	0.0469
17q11.2	rs7695479	17	31706495	chr17:31706495-31706495	17_30033514	T	0.0393
7p22.1	rs7797644	7	6446436	chr7:6446436-6446436	7_6486067	T	0.0386
10q26.13	rs4752700	10	122478096	chr10:122478096-122478096	10_124237612	A	0.0332
7q31.2	rs975722	7	117692860	chr7:117692860-117692860	7_117332914	A	0.0283
20q13.32	rs260020	20	59138970	chr20:59138970-59138970	20_57714025	T	0.0518
3q22.3	rs667920	3	136350630	chr3:136350630-136350630	3_136069472	T	0.0489
5q11.2	rs3936511	5	56564954	chr5:56564954-56564954	5_55860781	A	0.0366
20q12	rs6102343	20	41295639	chr20:41295639-41295639	20_39924279	A	0.0372
3p21.31	rs7633770	3	46647072	chr3:46647072-46647072	3_46688562	A	0.0295
14q23.1	rs2145598	14	58327283	chr14:58327283-58327283	14_58794001	A	0.0283
11p11.2	rs7116641	11	43675367	chr11:43675367-43675367	11_43696917	T	0.0314
4q22.3	rs3775058	4	95196220	chr4:95196220-95196220	4_96117371	A	0.0386
10p13	rs6184834	10	12261814	chr10:12261814-12261814	10_12303813	T	0.0363
2p21	rs582384	2	45669298	chr2:45669298-45669298	2_45896437	A	0.0332
6q25.1	rs1708009	6	150676265	chr6:150676265-150676265	6_150997401	T	0.0537
18q21.1	rs9964304	18	49703347	chr18:49703347-49703347	18_47229717	A	0.0382
14q32.13	rs1126352	14	94371805	chr14:94371805-94371805	14_94838142	T	0.1363
8q23.1	rs1009311	8	105553186	chr8:105553186-105553186	8_106565414	A	0.0319
3q25.31	rs4266144	3	157134803	chr3:157134803-157134803	3_156852592	C	0.0346
15q26.2	rs1758113	15	95603185	chr15:95603185-95603185	15_96146414	A	0.0373
6p21.2	rs1321309	6	36670859	chr6:36670859-36670859	6_36638636	A	0.028
12p13.31	rs1183826	12	7068568	chr12:7068568-7068568	12_7175872	T	0.0514
7p13	rs2107732	7	45038379	chr7:45038379-45038379	7_45077978	A	0.0567
2q37.3	rs1167793	2	237315312	chr2:237315312-237315312	2_238223955	A	0.0339
1q32.2	rs6015412	1	210295654	chr1:210295654-210295654	1_210468999	T	0.0444
20q13.12	rs3827066	20	45957384	chr20:45957384-45957384	20_44586023	T	0.0424
9q33.2	rs885150	9	121657894	chr9:121657894-121657894	9_124420173	T	0.0355
6q14.1	rs4613862	6	81902554	chr6:81902554-81902554	6_82612271	A	0.0319
7p22.3	rs1026759	7	1897625	chr7:1897625-1897625	7_1937261	A	0.036
4q21.1	rs1250082	4	76495474	chr4:76495474-76495474	4_77416627	A	0.0336
6q22.32	rs1591805	6	126395918	chr6:126395918-126395918	6_126717064	A	0.037
10q23.1	rs1768074	10	80491758	chr10:80491758-80491758	10_82251514	T	0.042
11q22.1	rs4754698	11	100761177	chr11:100761177-100761177	11_100631908	C	0.0368
1p36.32	rs2493298	1	3409348	chr1:3409348-3409348	1_3325912	A	0.0514
1p13.2	rs1180631	1	115210861	chr1:115210861-115210861	1_115753482	A	0.0337
2q24.3	rs1299990	2	164100741	chr2:164100741-164100741	2_164957251	A	0.0482
6p21.1	rs6905288	6	43791136	chr6:43791136-43791136	6_43758873	A	0.0386
4q32.3	rs7696431	4	168766574	chr4:168766574-168766574	4_169687725	T	0.0311
10q24.33	rs4918072	10	103933886	chr10:103933886-103933886	10_105693644	A	0.0386
3p21.31	rs7617773	3	48152025	chr3:48152025-48152025	3_48193515	T	0.0355
11p15.4	rs1160150	11	5679844	chr11:5679844-5679844	11_5701074	A	0.0782
7p21.3	rs1150988	7	12222285	chr7:12222285-12222285	7_12261911	A	0.0327
13q34	rs1317507	13	112977466	chr13:112977466-112977466	13_113631780	A	0.0398
3q26.31	rs12897	3	172398112	chr3:172398112-172398112	3_172115902	A	0.0358
12q22	rs1110790	12	95114195	chr12:95114195-95114195	12_95507971	A	0.0747
16q23.3	rs7199941	16	81872818	chr16:81872818-81872818	16_81906423	A	0.0367
5p15.31	rs1129410	5	9545986	chr5:9545986-9545986	5_9546098	A	0.0615
6p11.2	rs7156684	6	57283373	chr6:57283373-57283373	6_57148171	T	0.0829
19p13.11	rs7251815	19	17734133	chr19:17734133-17734133	19_17844942	T	0.0511
21q21.3	rs2832227	21	29160755	chr21:29160755-29160755	21_30533076	A	0.0393
3q22.1	rs1051286	3	132539117	chr3:132539117-132539117	3_132257961	T	0.0431
13q13.1	rs7998440	13	32551069	chr13:32551069-32551069	13_33125206	A	0.0383
8p21.3	rs6984210	8	22176102	chr8:22176102-22176102	8_22033615	C	0.0784
1p34.3	rs4072980	1	37990434	chr1:37990434-37990434	1_38456106	A	0.0326
4q21.22	rs1109949	4	81665896	chr4:81665896-81665896	4_82587050	A	0.039
8p22	rs6997340	8	18429487	chr8:18429487-18429487	8_18286997	T	0.0331
9q31.2	rs944172	9	107755513	chr9:107755513-107755513	9_110517794	T	0.0395
17q21.2	rs2074164	17	42118220	chr17:42118220-42118220	17_40270238	C	0.0423
17q11.2	rs1108010	17	29611406	chr17:29611406-29611406	17_27938424	T	0.0358
1p32.3	rs1159114	1	55039974	chr1:55039974-55039974	1_55505647	T	0.2406

REGION	SNPS	CHR_ID	CHR:POS (GRCh38p12)	to lift over	CHR:POS (GRCh37/hg19)	Allele	OR or BETA
5q31.1	rs273909	5	132331660	chr5:132331660-132331660	5_131667353	A	0.0488
2q22.3	rs2252641	2	145043894	chr2:145043894-145043894	2_145801461	T	0.0368
2q35	rs1250229	2	215439661	chr2:215439661-215439661	2_216304384	T	0.0435
4q32.1	rs7692387	4	155714157	chr4:155714157-155714157	4_156635309	A	0.0643
10q11.21	rs1870634	10	43985363	chr10:43985363-43985363	10_44480811	T	0.06
19p13.2	rs1168430	19	8364439	chr19:8364439-8364439	19_8429323	A	0.1402
16q13	rs1214954	16	56959249	chr16:56959249-56959249	16_56993161	A	0.0374
6q25.3	rs5573049	6	160584578	chr6:160584578-160584578	6_161005610	T	0.3122
11p15.4	rs472109	11	9748771	chr11:9748771-9748771	11_9770318	C	0.0391
2q35	rs2571445	2	217818431	chr2:217818431-217818431	2_218683154	A	0.037
8p21.3	rs1709189	8	19985660	chr8:19985660-19985660	8_19843171	T	0.0585
	rs2519093	9	133266456	chr9:133266456-133266456	9_136141870	T	0.0554
5q31.3	rs246600	5	143137332	chr5:143137332-143137332	5_142516897	T	0.043
15q22.31	rs6494488	15	64732005	chr15:64732005-64732005	15_65024204	A	0.0382
19p13.2	rs5579137	19	11077477	chr19:11077477-11077477	19_11188153	A	0.1157
6p24.1	rs9349379	6	12903725	chr6:12903725-12903725	6_12903957	A	0.1072
15q26.1	rs1751484	15	90873320	chr15:90873320-90873320	15_91416550	A	0.0559
12q24.12	rs7137828	12	111494996	chr12:111494996-111494996	12_111932800	T	0.0643
1p32.2	rs1711404	1	56500678	chr1:56500678-56500678	1_56966350	A	0.096
6p21.31	rs1334894	6	35647353	chr6:35647353-35647353	6_35615130	T	0.0482
1q32.1	rs6700559	1	200676945	chr1:200676945-200676945	1_200646073	T	0.0284
15q22.33	rs7274346	15	67149412	chr15:67149412-67149412	15_67441750	A	0.0576
2p11.2	rs6743030	2	85536397	chr2:85536397-85536397	2_85763520	T	0.0565
17p11.2	rs1293658	17	17640408	chr17:17640408-17640408	17_17543722	A	0.032
12q13.13	rs1117082	12	54120131	chr12:54120131-54120131	12_54513915	C	0.0832
3q21.2	rs4678145	3	124731234	chr3:124731234-124731234	3_124450081	C	0.0639
4q21.21	rs1085714	4	80259918	chr4:80259918-80259918	4_81181072	A	0.0491
4q27	rs7678555	4	119988346	chr4:119988346-119988346	4_120909501	A	0.0486
12q21.33	rs2681492	12	89619312	chr12:89619312-89619312	12_90013089	T	0.0558
1q21.3	rs4845625	1	154449591	chr1:154449591-154449591	1_154422067	T	0.0395
10q24.32	rs3740390	10	102878723	chr10:102878723-102878723	10_104638480	T	0.0662
14q32.2	rs8003602	14	99682624	chr14:99682624-99682624	14_100148961	T	0.0539
16q23.1	rs8046696	16	75408245	chr16:75408245-75408245	16_75442143	T	0.0479
2p24.1	rs515135	2	21063185	chr2:21063185-21063185	2_21286057	T	0.0555
19q13.32	rs7412	19	44908822	chr19:44908822-44908822	19_45412079	T	0.1368
11q13.5	rs606452	11	75565133	chr11:75565133-75565133	11_75276178	A	0.0466
12q24.31	rs1169288	12	120978847	chr12:120978847-120978847	12_121416650	A	0.049
7q36.1	rs3918226	7	150993088	chr7:150993088-150993088	7_150690176	T	0.1071
10q23.31	rs1412444	10	89243170	chr10:89243170-89243170	10_91002927	T	0.0559
8q24.13	rs6982502	8	125467120	chr8:125467120-125467120	8_126479362	T	0.0498
15q25.1	rs7173743	15	78849442	chr15:78849442-78849442	15_79141784	T	0.0636
4q31.22	rs6841581	4	147480038	chr4:147480038-147480038	4_148401190	A	0.0751
7p21.1	rs2107595	7	19009765	chr7:19009765-19009765	7_19049388	A	0.0752
21q22.11	rs2845106	21	34221526	chr21:34221526-34221526	21_35593827	A	0.1083
20q11.22	rs867186	20	35176751	chr20:35176751-35176751	20_33764554	A	0.0573
6p21.33	rs644045	6	31916180	chr6:31916180-31916180	6_31883957	A	0.0413
6p22.3	rs6909752	6	22612400	chr6:22612400-22612400	6_22612629	A	0.0442
3q25.2	rs789294	3	154339952	chr3:154339952-154339952	3_154057741	C	0.0642
16q23.3	rs7500448	16	83012185	chr16:83012185-83012185	16_83045790	A	0.0557
7q22.3	rs2189839	7	107589581	chr7:107589581-107589581	7_107230026	A	0.0345
18q21.32	rs663640	18	60178844	chr18:60178844-60178844	18_57846077	T	0.0383
11p15.3	rs7926712	11	13281538	chr11:13281538-13281538	11_13303085	A	0.0351
14q24.3	rs7145159	14	75116565	chr14:75116565-75116565	14_75583268	T	0.0319
1q32.1	rs2820315	1	201903136	chr1:201903136-201903136	1_201872264	T	0.0359
7q34	rs1023737	7	140057336	chr7:140057336-140057336	7_139757136	T	0.0338
17q23.2	rs1476098	17	61159652	chr17:61159652-61159652	17_59237013	A	0.0443
17q21.32	rs1760876	17	46935905	chr17:46935905-46935905	17_45013271	T	0.0469
17q23.3	rs9892152	17	64324605	chr17:64324605-64324605	17_62401965	T	0.033
13q12.3	rs9319428	13	28399484	chr13:28399484-28399484	13_28973621	A	0.036
3p25.1	rs3499191	3	14884844	chr3:14884844-14884844	3_14926351	T	0.0379
9p21.3	rs4977574	9	22098575	chr9:22098575-22098575	9_22098574	A	0.1788
11q23.3	rs651821	11	116791863	chr11:116791863-116791863	11_116662579	T	0.0692
17p13.3	rs170041	17	2266922	chr17:2266922-2266922	17_2170216	T	0.0469
3q22.3	rs185244	3	138374047	chr3:138374047-138374047	3_138092889	T	0.0644

REGION	SNPS	CHR_ID	CHR:POS (GRCh38p12)	to lift over	CHR:POS (GRCh37/hg19)	Allele	OR or BETA
19q13.2	rs4803455	19	41345604	chr19:41345604-41345604	19_41851509	A	0.0484
10p11.23	rs9337951	10	30028144	chr10:30028144-30028144	10_30317073	A	0.0543
12q24.31	rs1105783	12	124822507	chr12:124822507-124822507	12_125307053	A	0.0655
2p21	rs6544713	2	43846742	chr2:43846742-43846742	2_44073881	T	0.0492
13q34	rs9515203	13	110397276	chr13:110397276-110397276	13_111049623	T	0.0596
6q23.2	rs2327429	6	133888699	chr6:133888699-133888699	6_134209837	T	0.0655
2q33.2	rs6728861	2	203009020	chr2:203009020-203009020	2_203873743	A	0.1054
12q13.3	rs1161335	12	57398797	chr12:57398797-57398797	12_57792580	T	0.036
11q13.1	rs1280163	11	65623846	chr11:65623846-65623846	11_65391317	A	0.0403
6p21.2	rs1094778	6	39207146	chr6:39207146-39207146	6_39174922	T	0.0413
1q24.2	rs1892094	1	169125221	chr1:169125221-169125221	1_169094459	T	0.0362
2p24.1	rs1698695	2	19742712	chr2:19742712-19742712	2_19942473	A	0.0812
1q21.3	rs1120489	1	151795572	chr1:151795572-151795572	1_151768048	A	0.0487
12p12.2	rs1084144	12	20067099	chr12:20067099-20067099	12_20220033	C	0.046
7q32.2	rs1155692	7	130023656	chr7:130023656-130023656	7_129663496	T	0.0548
17q21.33	rs6207643	17	49327266	chr17:49327266-49327266	17_47404628	T	0.048
15q26.1	rs1807214	15	89022026	chr15:89022026-89022026	15_89565257	A	0.0639
16q22.2	rs1050362	16	72096916	chr16:72096916-72096916	16_72130815	A	0.0352
6p21.31	rs2814944	6	34585020	chr6:34585020-34585020	6_34552797	A	0.0479
1q41	rs3515867	1	222656208	chr1:222656208-222656208	1_222829550	A	0.0699
1p13.3	rs602633	1	109278889	chr1:109278889-109278889	1_109821511	T	0.0999
11q22.3	rs974819	11	103789839	chr11:103789839-103789839	11_103660567	T	0.0614
4q12	rs1708193	4	56956703	chr4:56956703-56956703	4_57822869	A	0.0399
