# Supplementary material for: Whole-genome sequencing analysis of semi-supercentenarians
Source: eLife. 2021 May 4;10:e57849. doi: 10.7554/eLife.57849 (PMC8096429; doi:10.7554/eLife.57849)
Supplement: Supplementary file 19. [file elife-57849-supp19.pdf]

Table 19S. SNPs used for PRS in van der Nelson et al 2017

REGION	SNPS	CHR_ID	CHR:POS (GRCh38p12)	to lift over	CHR:POS (GRCh37/hg19)	Allele	OR or BETA
1p32.3	rs11591147	1	1:55039974	chr1:55505647-55505647	1_55505647	G	1.25
1p32.2	rs56170783	1	1:56550459	chr1:57016131-57016131	1_57016131	A	1.11
1p13.3	rs7528419	1	1:109274570	chr1:109817192-109817192	1_109817192	A	1.11
1q21.3	rs6689306	1	1:154423470	chr1:154395946-154395946	1_154395946	A	1.05
1q41	rs67180937	1	1:222650401	chr1:222823743-222823743	1_222823743	G	1.07
2p24.1	rs16986953	2	2:19742712	chr2:19942473-19942473	2_19942473	A	1.11
2p24.1	rs585967	2	2:21047682	chr2:21270554-21270554	2_21270554	C	1.07
2p21	rs4299376	2	2:43845437	chr2:44072576-44072576	2_44072576	G	1.06
2p11.2	rs7568458	2	2:85561052	chr2:85788175-85788175	2_85788175	A	1.06
2q22.3	rs17678683	2	2:144528992	chr2:145286559-145286559	2_145286559	G	1.08
2q33.2	rs114123510	2	2:202966489	chr2:203831212-203831212	2_203831212	A	1.13
2q37.1	rs13003675	2	2:232719399	chr2:233584109-233584109	2_233584109	T	1.04
3q22.3	rs139016349	3	3:138380320	chr3:138099162-138099162	3_138099162	I	1.08
4q12	rs72627509	4	4:56972885	chr4:57839051-57839051	4_57839051	G	1.06
4q31.22	rs6841581	4	4:147480038	chr4:148401190-148401190	4_148401190	A	1.07
4q32.1	rs2306556	4	4:155717421	chr4:156638573-156638573	4_156638573	A	1.07
6p24.1	rs9349379	6	6:12903725	chr6:12903957-12903957	6_12903957	G	1.11
6p21.33	rs3130683	6	6:31920590	chr6:31888367-31888367	6_31888367	T	1.08
6p21.31	rs4472337	6	6:34801988	chr6:34769765-34769765	6_34769765	T	1.06
6p21.2	rs56015508	6	6:39184265	chr6:39152041-39152041	6_39152041	C	1.06
6q23.2	rs12202017	6	6:133852013	chr6:134173151-134173151	6_134173151	A	1.07
6q25.3	rs10455872	6	6:160589086	chr6:161010118-161010118	6_161010118	G	1.31
7p21.1	rs2107595	7	7:19009765	chr7:19049388-19049388	7_19049388	A	1.08
7q22.3	rs112370447	7	7:107536335	chr7:107176780-107176780	7_107176780	T	1.05
7q32.2	rs11556924	7	7:130023656	chr7:129663496-129663496	7_129663496	C	1.07
7q36.1	rs3918226	7	7:150993088	chr7:150690176-150690176	7_150690176	T	1.13
8p21.3	rs2083636	8	8:20007752	chr8:19865263-19865263	8_19865263	T	1.05
8q24.13	rs2954029	8	8:125478730	chr8:126490972-126490972	8_126490972	A	1.06
9p21.3	rs2891168	9	9:22098620	chr9:22098619-22098619	9_22098619	G	1.19
9q31.3	rs111245230	9	9:110407495	chr9:113169775-113169775	9_113169775	C	1.12
	rs507666	9	9:133273983	chr9:136149399-136149399	9_136149399	A	1.08
10p11.23	rs1887318	10	10:30032669	chr10:30321598-30321598	10_30321598	T	1.06
10q11.21	rs1870634	10	10:43985363	chr10:44480811-44480811	10_44480811	G	1.06
10q23.31	rs2246942	10	10:89245129	chr10:91004886-91004886	10_91004886	G	1.08
10q24.32	rs11191416	10	10:102845159	chr10:104604916-104604916	10_104604916	T	1.08
11p15.4	rs10840293	11	11:9729649	chr11:9751196-9751196	11_9751196	A	1.05
11q22.3	rs2839812	11	11:103802566	chr11:103673294-103673294	11_103673294	T	1.06
11q23.3	rs964184	11	11:116778201	chr11:116648917-116648917	11_116648917	G	1.05
12q13.3	rs2229357	12	12:57449928	chr12:57843711-57843711	12_57843711	G	1.05
12q21.33	rs2681472	12	12:89615182	chr12:90008959-90008959	12_90008959	G	1.07
12q24.12	rs10774625	12	12:111472415	chr12:111910219-111910219	12_111910219	A	1.07
12q24.31	rs11057830	12	12:124822507	chr12:125307053-125307053	12_125307053	A	1.07
13q12.3	rs1924981	13	13:28448508	chr13:29022645-29022645	13_29022645	T	1.05
13q34	rs11617955	13	13:110165755	chr13:110818102-110818102	13_110818102	T	1.09
14q32.2	rs10139550	14	14:99679373	chr14:100145710-100145710	14_100145710	G	1.05
15q22.33	rs72743461	15	15:67149412	chr15:67441750-67441750	15_67441750	C	1.07
15q25.1	rs7164479	15	15:78830712	chr15:79123054-79123054	15_79123054	T	1.07
15q26.1	rs2083460	15	15:89031253	chr15:89574484-89574484	15_89574484	T	1.07
15q26.1	rs2071382	15	15:90884967	chr15:91428197-91428197	15_91428197	T	1.06
16q13	rs247616	16	16:56955678	chr16:56989590-56989590	16_56989590	C	1.04
17p13.3	rs113348108	17	17:2185555	chr17:2088849-2088849	17_2088849	D	1.05
17p11.2	rs9897596	17	17:17690139	chr17:17593453-17593453	17_17593453	T	1.04
17q21.32	rs4643373	17	17:49046061	chr17:47123423-47123423	17_47123423	T	1.05
17q23.2	rs8068952	17	17:61209283	chr17:59286644-59286644	17_59286644	G	1.07
19p13.2	rs116843064	19	19:8364439	chr19:8429323-8429323	19_8429323	G	1.17
19p13.2	rs6511720	19	19:11091630	chr19:11202306-11202306	19_11202306	G	1.14
19q13.32	rs7412	19	19:44908822	chr19:45412079-45412079	19_45412079	C	1.15
21q22.11	rs28451064	21	21:34221526	chr21:35593827-35593827	21_35593827	A	1.14
22q11.23	rs180803	22	22:24262890	chr22:24658858-24658858	22_24658858	G	1.18
1q21.3	rs11810571	1	1:151789832	chr1:151762308-151762308	1_151762308	G	1.057
2q35	rs1250229	2	2:215439661	chr2:216304384-216304384	2_216304384	T	1.071
3p21.31	rs7623687	3	3:49411133	chr3:49448566-49448566	3_49448566	A	1.076

REGION	SNPS	CHR_ID	CHR:POS (GRCh38p12)	to lift over	CHR:POS (GRCh37/hg19)	Allele	OR or BETA
3q21.2	rs142695226	3	3:124756354	chr3:124475201-124475201	3_124475201	G	1.071
3q25.2	rs12493885	3	3:154122077	chr3:153839866-153839866	3_153839866	C	1.073
4q21.21	rs10857147	4	4:80259918	chr4:81181072-81181072	4_81181072	T	1.054
4q27	rs7678555	4	4:119988346	chr4:120909501-120909501	4_120909501	C	1.052
6p22.3	rs6909752	6	6:22612400	chr6:22612629-22612629	6_22612629	A	1.051
11p15.3	rs3993105	11	11:13281524	chr11:13303071-13303071	11_13303071	T	1.048
12q24.31	rs2244608	12	12:120979185	chr12:121416988-121416988	12_121416988	G	1.053
16q23.3	rs7500448	16	16:83012185	chr16:83045790-83045790	16_83045790	A	1.063
19q13.2	rs8108632	19	19:41348629	chr19:41854534-41854534	19_41854534	T	1.048
19q13.32	rs1964272	19	19:45687010	chr19:46190268-46190268	19_46190268	G	1.047
